# The global, regional, and national patterns of change in the burden of congenital birth defects, 1990–2021: an analysis of the global burden of disease study 2021 and forecast to 2040

**DOI:** 10.1016/j.eclinm.2024.102873

**Published:** 2024-10-04

**Authors:** Zihao Bai, Jingru Han, Jia An, Hao Wang, Xueying Du, Zhaocong Yang, Xuming Mo

**Affiliations:** a Nanjing Children's Hospital, Clinical Teaching Hospital of Medical School, Nanjing University, Nanjing, 210008, China; b Guangzhou Key Laboratory of Basic and Applied Research of Oral Regenerative Medicine, Guangdong Engineering Research Center of Oral Restoration and Reconstruction, Affiliated Stomatology Hospital of Guangzhou Medical University, Guangzhou, 510182, China; c Department of Cardiothoracic Surgery, Children's Hospital of Nanjing Medical University, Nanjing, 210008, China

**Keywords:** Disability-adjusted life-years, Health inequality, Congenital birth defects, Sociodemographic index, Global burden of disease

## Abstract

**Background:**

Congenital birth defects (CBDs) present enormous challenges to global healthcare systems. These conditions severely impact patients' health and underscore issues related to socioeconomic development and healthcare accessibility and efficiency. Previous studies have been geographically limited and lacked comprehensive global analysis. This study provides global, regional, and national disability-adjusted life years (DALYs) data for four major congenital birth defects—congenital heart defects (CHD), neural tube defects (NTDs), digestive congenital anomalies (DCAs), and Down syndrome (DS) from 1990 to 2021, emphasizing health inequalities. The goal is to offer scientific evidence for optimizing resource allocation, focusing on high-burden populations, and reducing disease burden.

**Methods:**

This study systematically evaluated the global, regional, and national burden of CBDs and their changes from 1990 to 2021 using the Global Burden of Diseases, Injuries, and Risk Factors Study (GBD) 2021. To conduct a more focused analysis, four specific CBDs were selected: CHD, NTDs, DCAs, and DS. DALYs were used as the metric, combined with the sociodemographic index (SDI). Analyses included the slope index of inequality and concentration index to measure health inequalities, frontier analysis to estimate achievable outcomes based on development levels, decomposition analysis to identify drivers of disease burden changes, Joinpoint regression analysis to assess temporal trends, and the Bayesian age-period-cohort (BAPC) model to predict future disease burden trends.

**Findings:**

Compared to 1990, the global burden of the CBDs in 2021 showed a downward trend. Males had a higher burden than females, with the highest burden observed in low-SDI regions. When examining CHD, NTDs, DCAs, and DS specifically, trends in burden changes varied across different CBDs at the global, regional, and national levels. Frontier analysis revealed potential for burden improvement in various countries and territories. Decomposition analysis highlighted differences in disease burden drivers across SDI regions, showing the greatest improvement observed in low-SDI regions. Joinpoint regression analysis indicated a downward trend in DALYs burden across SDI regions, and BAPC model predictions suggested that the burden of CBDs will continue to decline in the future.

**Interpretation:**

CBDs pose a major challenge to global public health. Despite an overall decline in disease burden, health inequalities remain prominent, particularly in countries and territories with lower levels of development. Future public health interventions should focus on countries and territories with low levels of development by optimizing healthcare resource allocation, improving basic health infrastructure, enhancing health education, and reducing disease burden inequalities. Global collaboration and data sharing are essential to promote a lifecycle management model for CBDs research and treatment, advancing global health development.

**Funding:**

This study was supported by the 10.13039/501100001809National Natural Science Foundation of China (No. 82270310) and the 10.13039/501100013058Jiangsu Provincial Key Research and Development Program (No. BE2023662).

**Research in context**

**Evidence before this study**

Prior research on congenital birth defects (CBDs), particularly focusing on congenital heart defects (CHD), neural tube defects (NTDs), digestive congenital anomalies (DCAs), and Down syndrome (DS), has been limited and lacking in comprehensiveness. To gather existing evidence, we conducted a search of the PubMed database up to September 2024 using the terms “global burden”, “congenital birth defects”, “GBD”, and “DALYs”. This study leverages the latest Global Burden of Diseases, Injuries, and Risk Factors Study (GBD) 2021 data to analyze the global, regional, and national burden of CBDs from 1990 to 2021, with an emphasis on health inequalities and changes in disease burden, particularly for these four specific CBDs.

**Added value of this study**

This is the first study to systematically evaluate the global burden of CHD, NTDs, DCAs, and DS from 1990 to 2021, using GBD 2021 data. It identifies inequalities in the burden of CBDs across different sociodemographic index (SDI) regions, uncovers the driving factors behind changes in disease burden through decomposition analysis, and explores potential improvements through frontier analysis. Additionally, Joinpoint regression analysis was used to identify temporal trends in the burden, and the Bayesian age-period-cohort (BAPC) model was applied to predict future trends up to 2040, further highlighting the urgent need for interventions in low-SDI regions where the burden remains concentrated.

**Implications of all the available evidence**

This study provides the most up-to-date and comprehensive global assessment of the burden of CHD, NTDs, DCAs, and DS, offering critical insights for clinicians, public health practitioners, and policymakers. The findings emphasize the necessity for targeted public health strategies to address health inequalities, improve resource allocation, and enhance prenatal and postnatal screening and care in low-SDI regions. Additionally, the study calls for strengthened international collaboration to reduce the global burden of congenital anomalies and ensure equitable access to healthcare services for affected populations.

## Introduction

Congenital birth defects (CBDs) primarily refer to structural abnormalities that arise during embryonic development. These defects are typically detectable before birth and may also be associated with functional impairments. These defects can severely impact physical capabilities, cognitive development, and may even threaten life. The occurrence of congenital birth defects typically results from a complex interplay between genetic factors (such as single-gene mutations and chromosomal abnormalities) and environmental influences (such as maternal nutrition, drug exposure, and infections). Recent studies have also highlighted the critical role of epigenetic regulation in this process, which, by altering gene expression, further increases the risk of these defects.^1^^,^^2^ According to the World Health Organization's 2023 report, approximately 6% of newborns worldwide are affected by congenital birth defects, with about 240,000 newborns dying within 28 days of birth due to these conditions, underscoring their substantial impact on the global disease burden.^3^^,^^4^ Although some research has addressed the global burden of congenital birth defects, these studies often focus on specific periods and regions, lacking a comprehensive analysis of major congenital birth defects, particularly those based on the latest Global Burden of Diseases, Injuries, and Risk Factors Study (GBD) 2021 data.

The GBD 2021 systematically analyzes and integrates global disease and health data, making it a valuable tool for studying the global, regional, and national burden of CBDs.^5^ Sociodemographic index (SDI) regions, categorized based on levels of social development, provide a method to detect health inequalities, analyze absolute and relative inequalities, and reveal the temporal impact of socioeconomic development levels on disease burden. Further research, policy-making, international cooperation, and technological improvements can more effectively address the challenges of congenital birth defects and promote global health development.^6^

This study systematically assessed the burden of CBDs from 1990 to 2021, encompassing all 11 major structural defects as defined in GBD 2021. For a more detailed and focused analysis, we selected four representative types of CBDs—congenital heart defects (CHD), neural tube defects (NTDs), digestive congenital anomalies (DCAs), and Down syndrome (DS)—for secondary, phenotype-specific, and primary analyses. These representative diseases were chosen because of their substantial contribution to the overall burden of congenital birth defects, enabling us to conduct an in-depth study of their trends and determinants. These four defects were selected not only due to their high prevalence and considerable global public health impact but also because they encompass both structural defects and chromosomal abnormalities, offering a comprehensive reflection of the burden of various types of congenital birth defects. Analyzing these defects provides insights into their association with socioeconomic development levels and offers scientific evidence for developing more targeted public health strategies.

This study comprehensively analyzed data using various methods to address multiple aspects of the burden of CHD, NTDs, DCAs, and DS. The slope index of inequality and concentration index were employed to assess absolute and relative health inequalities between different countries and territories. Frontier analysis was used to estimate the optimal achievable health outcomes based on SDI levels. Decomposition analysis was utilized to break down the drivers of changes in disease burden, identifying the contributions of key factors. Joinpoint regression analysis was applied to detect critical shifts in temporal trends. Based on predictions from the BAPC model, this study aims to highlight public health challenges that may emerge by 2040, offering a timeline to help policymakers optimize global health resource allocation and develop proactive interventions to reduce the burden of congenital birth defects. It revealed trends in disease burden changes across different SDI levels, sexes, and geographical regions. Unlike previous studies that primarily focused on incidence and mortality, this study employed disability-adjusted life years (DALYs) as a comprehensive metric to assess the disease burden. DALYs combine years of life lost (YLLs) and years lived with disability (YLDs), thereby providing a holistic view of the health burden of congenital birth defects across the entire life cycle of patients, from onset to death, rather than being limited to specific points in time. These findings provide valuable epidemiological information for disease treatment research, helping clinicians, epidemiologists, and health policymakers optimize healthcare resource allocation and develop more effective public health strategies.

## Methods

### Data source

All data in this study were retrieved from the GBD 2021 using the GBD results tool on the Institute for Health Metrics and Evaluation (IHME) website (http://ghdx.healthdata.org/). The GBD 2021 study analyzed 100,983 data sources, including vital registration systems, verbal autopsies, censuses, household surveys, specific disease registries, health service contact data, and other sources, to estimate YLDs, YLLs, and DALYs for 371 diseases and injuries. This analysis systematically adjusted epidemiological data to account for biases arising from differences in data sources, definitions, and measurement methods. These adjustments were achieved through complex statistical models such as MR-BRT and DisMod-MR 2.1, ensuring internal consistency in estimates across different regions, ages, sexes, and years. The process aims to minimize the impact of heterogeneity on study results through standardization and calibration steps. Since GBD 2019, the GBD location hierarchy has included all of the World Health Organization's (WHO) Member States. Unless otherwise specified, data from 204 countries and territories are not duplicated. For detailed procedures, refer to Appendix 1.

Despite the various methods available for defining birth defects, GBD 2021 integrates International Classification of Diseases (ICD) codes with disease burden analysis results to provide burden estimates for 11 major birth defects (https://ghdx.healthdata.org/record/ihme-data/gbd-2021-cause-icd-code-mappings). These include congenital heart anomalies, neural tube defects, digestive congenital anomalies, Down syndrome, orofacial clefts, Turner syndrome, Klinefelter syndrome, other chromosomal abnormalities, congenital musculoskeletal and limb anomalies, urogenital congenital anomalies, and other congenital birth defects. A detailed description of the ICD code classifications for these 11 birth defects in GBD 2021 can be found in Appendix 2.

We selected DALYs data for CBDs from GBD 2021 as the analysis metric. DALYs, which combine YLDs and YLLs, reflect early death and loss of quality of life, providing a comprehensive assessment of disease burden. This indicator is crucial for directly comparing different health conditions and populations, offering a reference point for assessing health inequalities. While DALYs alone may not directly reveal health inequalities, when combined with other inequality indicators, they serve as a comprehensive and effective measure that can more accurately reflect the relationship between CBDs and health disparities. This allows for an assessment of how health inequalities impact CBD patients throughout their lifecycle. Additionally, we employed the SDI as a comprehensive measure of social development. The SDI is a composite index calculated as the geometric mean of three standardized indicators: total fertility rate under age 25, mean years of schooling for those aged 15 and older, and lag-distributed income per capita. This index, calculated at the national level, accurately reflects the level of social development across different countries and territories.^5^ Based on SDI, the 204 countries and territories in GBD 2021 were classified into five development levels according to their SDI values, ranging from low to high. Specifically, the SDI value ranges from 0 to 1, with higher values indicating higher socioeconomic status. To ensure accuracy and clarity in presentation, we rounded the SDI threshold values. The countries and territories were categorized into different development levels based on the following SDI thresholds: low-SDI region [0–0.4658), low-middle-SDI region [0.4658–0.6188), middle-SDI region [0.6188–0.7120), high-middle-SDI region [0.7120–0.8103), and high-SDI region [0.8103–1.0000]. This classification enables a more systematic analysis of the impact of socioeconomic development levels on health outcomes.

### Statistics

Both the rate and the number of DALYs cases are used to assess the burden of CBDs. The DALYs rate is expressed as estimates per 100,000 population, while the number of cases reflects the absolute burden in terms of total cases. Both metrics are presented with their 95% uncertainty intervals (UI). All analyses were conducted using appropriate statistical models, and *p* < 0.05 was considered statistically significant. Detailed descriptions of each specific analysis method, including the slope index of inequality, concentration index, frontier analysis, decomposition analysis, Joinpoint regression analysis, and BAPC model, are provided in subsequent sections. All analyses and visualizations were completed using the World Health Organization's Health Equity Assessment Toolkit and R Software (version 4.3.2).

### Cross-country inequality analysis

This study employed the slope index of inequality (SII) and concentration index, as defined by the WHO, to measure absolute and relative inequality in disease burden.^7^ These indices were used to quantify disparities in the distribution of CHD, NTDs, DCAs, and DS across countries and territories, offering a comprehensive evaluation of health inequalities. The SII is calculated by regressing the DALYs rate on the SDI, using the midpoint of the cumulative population distribution sorted by SDI. To analyze changes in health inequality, we compared data from 204 countries and territories between 1990 and 2021. To better control for bias and heterogeneity, we employed a robust regression model (rlm) instead of the ordinary linear regression model (lm) in our health inequality analysis. The robust regression model reduces sensitivity to outliers, minimizing bias caused by data heterogeneity or extreme values and leading to a more accurate representation of health inequality. Additionally, the concentration index is calculated by matching the cumulative proportion of DALYs with the cumulative population distribution ranked by SDI and numerically integrating the area under the Lorenz curve.

### Frontier analysis and decomposition analysis

To assess the relationship between the burden of CHD, NTDs, DCAs, and DS and sociodemographic development levels, we employed frontier analysis to construct an age-standardized DALYs rate (ASDR)-based frontier model using the SDI. Unlike traditional regression models that describe relationships between variables or predict outcomes, complex statistical methods such as frontier analysis were employed to account for the non-linear relationship between SDI and disease burden and to capture the multidimensional drivers behind the burden of CHD, NTDs, DCAs, and DS. Frontier analysis focuses on determining the theoretically lowest ASDR value each country or territory could achieve based on its current development level, serving as a benchmark for optimal performance. This method quantifies the gap between a country's or territory's current burden and its potential minimum burden, thereby identifying areas where improvements can be made. We applied locally weighted regression (LOESS) combined with local polynomial regression, using different smoothing spans (0.3, 0.4, 0.5) to generate smooth frontier lines, thereby capturing the nonlinear relationship between SDI and ASDR. To ensure the robustness of the analysis, we conducted 1000 bootstrap samples and calculated the average ASDR for each SDI value. By measuring the absolute distance between each country's or territory's 2021 ASDR and the frontier line (i.e., effective difference), we assessed the improvement potential of each country or territory.^8^

Additionally, we utilized the Das Gupta decomposition method to break down the changes in the burden of CHD, NTDs, DCAs, and DS from 1990 to 2021 into contributions from aging, population growth, and epidemiological changes. This approach enabled us to dissect the overall changes in burden into these key factors, offering clearer insights into how demographic and epidemiological shifts have shaped the trends over time. Unlike traditional methods such as linear regression, which primarily focus on establishing relationships between variables, decomposition analysis enables a detailed assessment of the independent contributions of each factor to the overall changes in disease burden. By dissecting these trends, we gained clearer insights into the underlying drivers of changes in the global burden of congenital birth defects.

Specifically, we calculated DALYs for each region using the following formula:

$$DALY_{a_{y},p_{y},e_{y}}=\sum_{i=1}^{20}\left(a_{i,y}\times p_{y}\times e_{i,y}\right)$$

where $DALY_{a_{y},p_{y},e_{y}}$ represents the DALYs based on the age structure, population size, and DALYs rate for a specific year *y*; $a_{i,y}$ represents the proportion of the population in age group *i* out of 20 age groups in year *y*; $p_{y}$ represents the total population in year *y*; and $e_{i,y}$ represents the DALYs rate in age group *i* in year *y*. By isolating other variables, we quantified the unique impact of a single factor on the changes in DALYs.^9^

### Joinpoint regression analysis

To evaluate the temporal trend of CHD, NTDs, DCAs, and DS from 1990 to 2021, we performed Joinpoint regression analysis to understand the temporal trend in disease burden.^10^ This model employs segmented regression on a log-linear regression model, represented as ln(y)=β×x+constant, to identify inflection points in the trend. We utilized the grid search method (GSM) to calculate all possible join points, selecting the one with the smallest mean squared error (MSE) as the optimal inflection point. Building on this, the optimal number of join points was determined using the Monte Carlo permutation test, allowing for a maximum of 5 join points and a minimum of 0. The final model calculated the annual percentage change (APC), average annual percentage change (AAPC), and the corresponding 95% confidence intervals (CI) for the world and each SDI region to quantify trend changes from 1990 to 2021.

The calculation formula for APC is:

$$APC=\left(e^{\beta }-1\right)\times 100\%$$

where β is the regression coefficient from the log-linear model ln(y)=β×x+constant. AAPC reflects the overall trend change by weighting each segment's APC according to the time span. This analytical method not only enhances the precision in identifying temporal trends but also improves the robustness of the model.

### BAPC model projection

This study employed the BAPC model to predict future disease burdens due to its capacity to manage complex, high-dimensional, and sparse data frequently encountered in large-scale epidemiological studies like GBD 2021.^11^ The BAPC model builds on the traditional generalized linear model (GLM) framework within a Bayesian context, allowing the dynamic integration of age, period, and cohort effects. These effects are assumed to evolve continuously over time and are smoothed using a second-order random walk, resulting in more accurate posterior probability predictions. A notable strength of the BAPC model is its use of the Integrated Nested Laplace Approximation (INLA) method for approximating the marginal posterior distribution. This approach effectively bypasses challenges such as mixing and convergence issues often associated with Markov Chain Monte Carlo techniques, while maintaining computational efficiency. The model's flexibility and robustness in handling time series data make it particularly suitable for long-term disease burden predictions. Given its comprehensive coverage and ability to capture temporal trends, the BAPC model has been widely validated and applied in epidemiological research, especially in studies involving age-structured population data and complex cohort effects. In this study, we utilized the “BAPC” R package to forecast the global burden of CHD, NTDs, DCAs, and DS, leveraging GBD 2021 data and demographic projections from the IHME. This approach enables nuanced predictions of future disease burdens while considering the intricate interactions of age, period, and cohort effects.

### Ethics statement

For GBD studies, the Institutional Review Board of the University of Washington reviewed and approved a waiver of informed consent (https://www.healthdata.org/research-analysis/gbd).

### Role of funding source

The funders of the study had no role in study design, data collection, data analysis, data interpretation, or writing of the report. All authors had full access to all the data in the study and accepted responsibility to submit for publication.

## Results

### Congenital birth defects

**Fig. 1:**
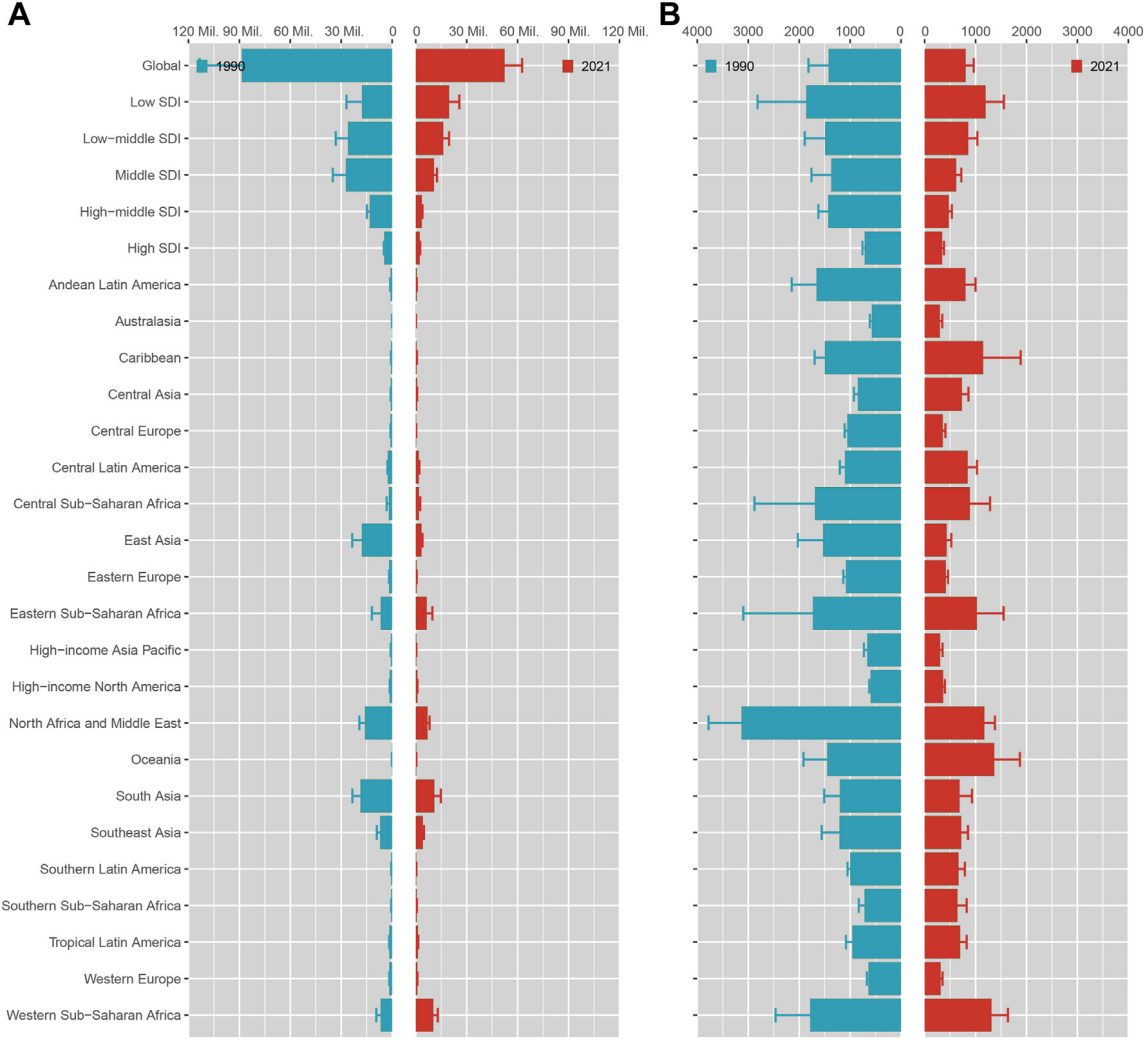
All-age number of CBDs DALYs cases (A) and age-standardized rates (B) in the world, various SDI regions, and 21 GBD regions in 1990 and 2021. CBDs, congenital birth defects; DALYs, disability-adjusted life-years; SDI, sociodemographic index.

In 1990, the disease burden of CBDs showed differences in various aspects (Fig. 1A and B and Table S1). Globally, the number of all-age cases of CBDs was 88,529,007.36 (95% UI: 56,817,570.65–113,394,608.92), and the ASDR was 1420.72 (95% UI: 915.54–1818.12). The ASDR for males was higher than for females (male: 1490.32; 95% UI: 961.4–1967.08; female: 1346.1; 95% UI: 771.89–1846.34). The disease burden is highest in low-SDI regions (1858.89; 95% UI: 875.31–2816.95) and progressively decreases as the SDI level rises. Specifically, among the 204 countries and territories, Afghanistan exhibited the highest ASDR (5202.14; 95% UI: 1467.69–7188.97), while China recorded the highest number of all-age CBD cases (17,420,113.08; 95% UI: 11,803,475.94–23,131,702.34). In contrast, the Northern Mariana Islands had the lowest ASDR (361.67; 95% UI: 283.31–457.82), and Tokelau recorded the lowest number of all-age CBD cases (15.12; 95% UI: 9.9–19.44).

In 2021, the disease burden of CBDs showed improvements but still exhibited notable differences. Globally, the number of all-age cases of CBDs was 52,325,332.69 (95% UI: 45,186,944.83–62,682,127.14), and the ASDR was 802.17 (95% UI: 692.41–962.51). The ASDR for males remained higher than for females, although the gap decreased (male: 825.17; 95% UI: 690.99–1025.15; female: 777.67; 95% UI: 653.66–954.87). The disease burden remains highest in low-SDI regions (1193.38; 95% UI: 943.47–1554.37), with ASDR gradually decreasing as the SDI level increases. Among countries and territories, Afghanistan recorded the highest ASDR (2591.86; 95% UI: 1505.58–3414.02), while India had the highest number of all-age CBDs cases (7,814,429.92; 95% UI: 6,037,520.94–10,361,178.2). On the other hand, San Marino had the lowest ASDR (161.38; 95% UI: 125.48–208.13), and Tokelau showed the lowest number of all-age CBDs cases (22.9; 95% UI: 18.65–30.88).

**Fig. 2:**
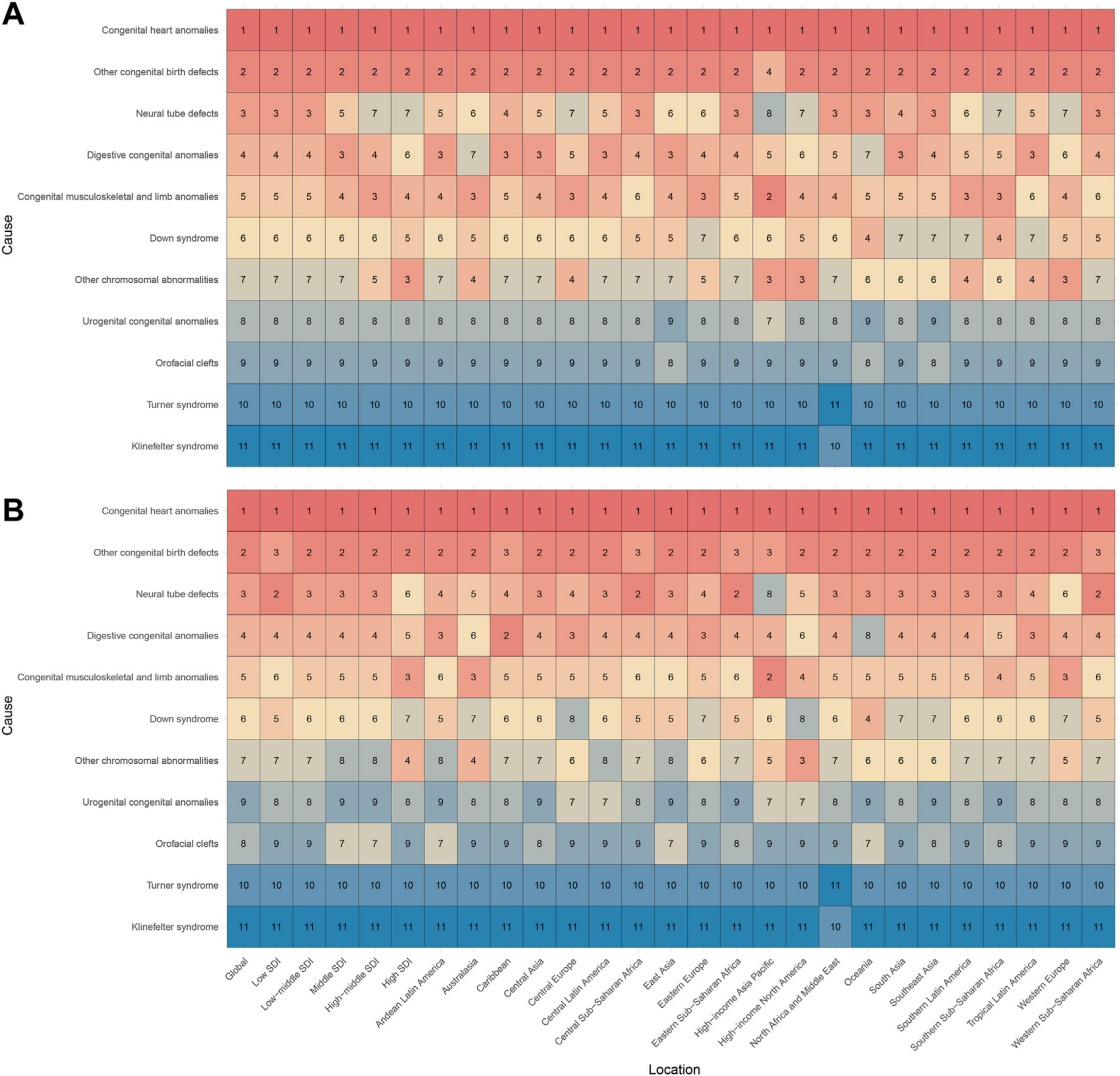
Ranking of age-standardized disability-adjusted life years rates for all congenital birth defects by location, 2021 (A) and 1990 (B). The colors in the figure represent rankings from high (red) to low (blue), with the numbers representing the specific rankings of 11 types of CBDs globally, in various SDI regions, and 21 GBD regions. CBDs, congenital birth defects; SDI, sociodemographic index.

When ranking the ASDR of 11 types of CBDs globally, across 5 SDI regions, and 21 GBD regions, differences were observed between 2021 and 1990 (Table S2). As shown in Fig. 2A, in 2021, CHD was the CBD disease with the highest burden in all regions. Other congenital birth defects ranked second in all GBD regions, except in the High-income Asia Pacific region, where they ranked differently. It is important to note that “Other congenital birth defects” refers to conditions that are not directly categorized within the specific subgroups of CBDs as defined by GBD 2021. These conditions include disorders such as Arnold-Chiari syndrome, Dandy-Walker syndrome, and other specified or unspecified congenital malformations of the central nervous system. This category serves to encompass congenital anomalies that have not been clearly classified under other CBD subtypes, ensuring that a broad range of less common or less well-defined congenital conditions are included in disease burden assessments. Neural tube defects, digestive congenital anomalies, congenital musculoskeletal and limb anomalies, Down syndrome, and other chromosomal abnormalities had varying rankings in different regions but generally ranked as the third to seventh highest disease burdens in each region. Additionally, except for North Africa and the Middle East, Klinefelter syndrome was the CBDs disease with the lowest burden in all regions. In 1990, CHD was also the CBDs disease with the highest burden in all regions (Fig. 2B). However, neural tube defects were the second highest disease burden after CHD in multiple regions, including low-SDI, Central Sub-Saharan Africa, and Eastern Europe-Saharan Africa, while digestive congenital anomalies ranked second in the Caribbean.

### Congenital heart defects

In 1990, the ASDR of CHD showed differences in various aspects (Table S3). The ASDR for males was higher than for females (male: 840.45; 95% UI: 452.13–1165.71; female: 653.77; 95% UI: 361.07–843.73). When categorized by SDI regions, low-SDI regions exhibited the highest disease burden (860.62; 95% UI: 324.42–1252.88). Among GBD regions, North Africa and the Middle East had the heaviest burden (1670.33; 95% UI: 755.19–2355.8). Specifically, among the 204 countries and territories, Afghanistan exhibited the highest ASDR (3002.73; 95% UI: 705.33–4981.45), while China recorded the highest number of all-age CHD cases (10,962,853.36; 95% UI: 6,949,618.27–15,100,267.73). Conversely, San Marino recorded the lowest ASDR (110.05; 95% UI: 79.12–150.31), and Tokelau had the lowest number of all-age CHD cases (8.14; 95% UI: 4.95–10.72). These results highlight the sex and socioeconomic disparities in the CHD burden.

In 2021, the ASDR for males was still higher than for females, although the gap decreased (male: 373.74; 95% UI: 294.98–485.29; female: 314.77; 95% UI: 246–381.74). Low-SDI regions still had the heaviest burden among SDI regions, but the GBD region with the heaviest burden shifted to Oceania (low-SDI: 488.54; 95% UI: 349.65–658.02; Oceania: 786.51; 95% UI: 364.8–1169.36). Among countries and territories, Afghanistan continued to exhibit the highest ASDR (1395.89; 95% UI: 691.74–1982.48), while India recorded the highest number of all-age CHD cases (3,557,478.65; 95% UI: 2,666,545.8–4,881,692). San Marino had the lowest ASDR (34.86; 95% UI: 23.01–49.72) and also recorded the lowest number of all-age CHD cases (6.98; 95% UI: 4.38–9.93). In 2021, although the overall CHD burden had decreased, notable sex and regional disparities persisted, with low-SDI regions still facing considerable inequalities compared to high-income areas.

**Fig. 3:**
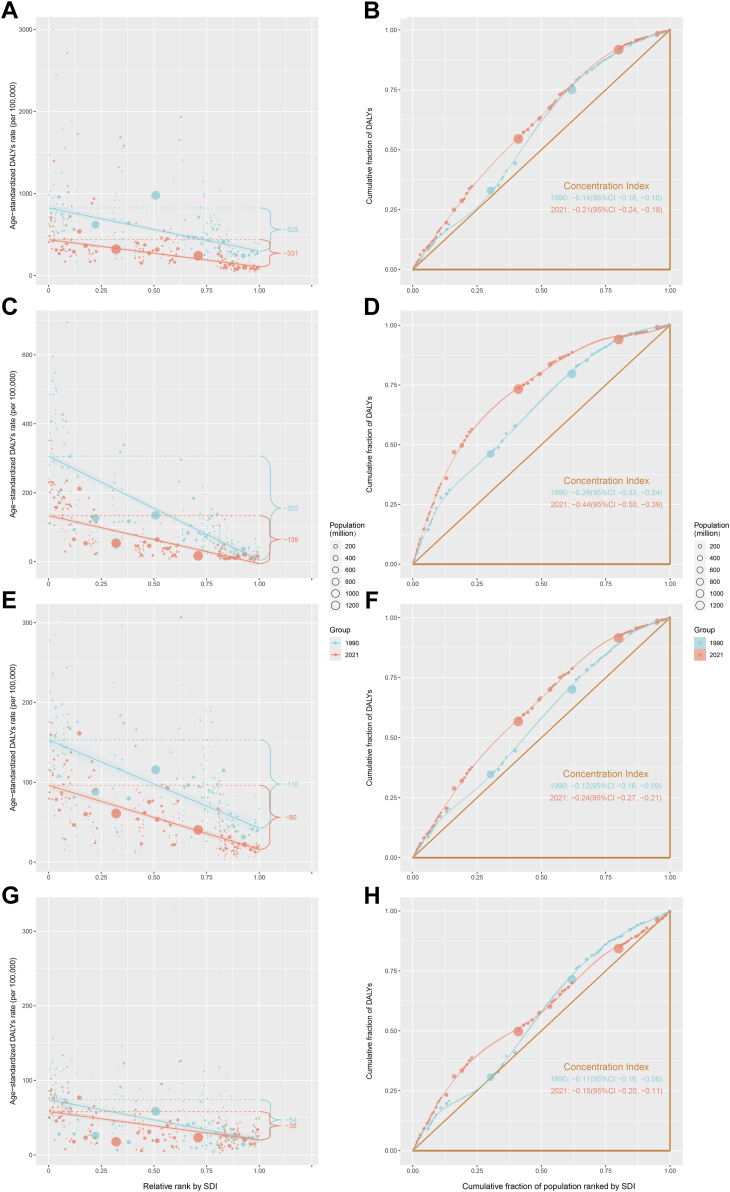
Health inequality regression curves and concentration curves for the DALYs of CHD (A and B), NTDs (C and D), DCAs (E and F), and DS (G and H) worldwide, 1990 and 2021. Panels A, C, E, and G illustrate the slope index of inequality, depicting the relationship between SDI and age-standardized DALYs rates for each condition, with points representing individual countries and territories sized by population. Panels B, D, F, and H present the concentration index, which quantifies relative inequalities by integrating the area under the Lorenz curve, aligning DALYs distribution with population distribution by SDI. Blue represents data from 1990, and red represents data from 2021. DALYs, disability-adjusted life-years; CHD, congenital heart defects; NTDs, neural tube defects; DCAs, digestive congenital anomalies; DS, Down syndrome; SDI, sociodemographic index.

In terms of the burden of CHD, we observed significant absolute and relative inequalities associated with the SDI, with countries and territories having lower SDI disproportionately bearing a higher burden (Fig. 3A and B and Tables S4 and S5, *p* < 0.05). As shown by the slope index of inequality, the gap in the DALYs rate between the highest and lowest SDI countries and territories decreased from −524.61 (95% CI: −614.14 to −435.08) in 1990 to −330.56 (95% CI: −377.48 to −283.63) in 2021. The concentration index was −0.14 (95% CI: −0.18 to −0.10) in 1990, but increased to −0.21 (95% CI: −0.24 to −0.18) in 2021. These results suggest that while absolute health inequalities in CHD burden decreased from 1990 to 2021, relative inequalities have increased.

**Fig. 4:**
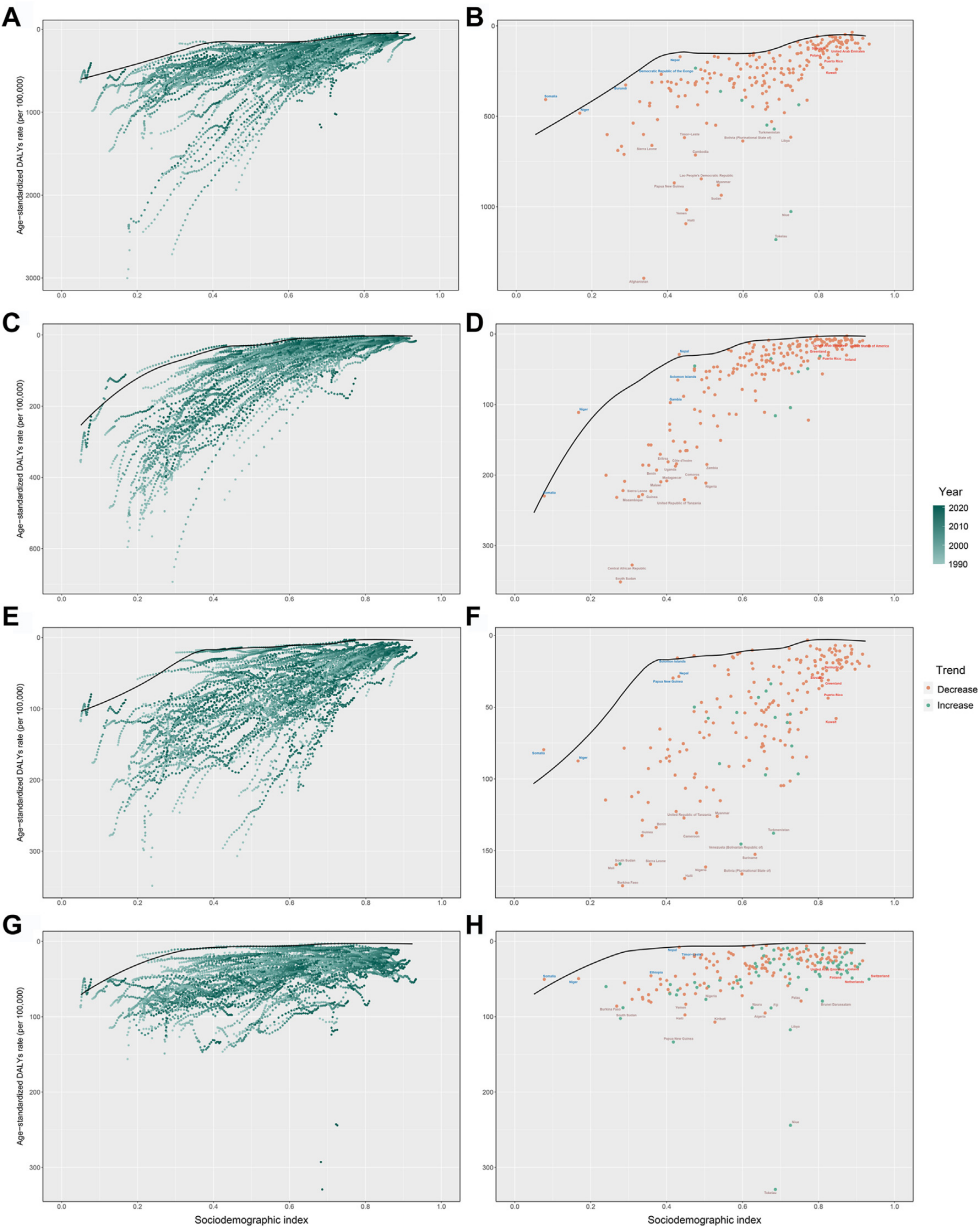
Frontier analysis exploring the relationship between SDI and ASDR for CHD (A and B), NTDs (C and D), DCAs (E and F), and DS (G and H) in 204 countries and territories. In Figures A, C, E, and G, the color change from light green (1990) to dark green (2021) represents the change in years. In Figures B, D, F, and H, each point represents a specific country or territory in 2021, the frontier line is shown in black, and the top 15 countries and territories with the largest differences from the frontier are marked in brown. Blue represents low-SDI with the smallest differences from the frontier, red represents high-SDI with the largest differences from the frontier. The direction of ASDR change from 1990 to 2021 is indicated by the color of the dots, with orange dots representing decreases and green dots representing increases. CHD, congenital heart defects; NTDs, neural tube defects; DCAs, digestive congenital anomalies; DS, Down syndrome; SDI, sociodemographic index; ASDR, age-standardized disability-adjusted life years rate.

Using data from 1990 to 2021 and based on ASDR and SDI, frontier analysis was conducted to explore the potential improvement space of the ASDR for CHD considering national and regional development levels (Fig. 4A and B and Table S6). The 15 countries and territories with the largest actual differences in potential improvement (effective difference range: 1225.01–449.98) include Afghanistan, Tokelau, Haiti, Niue, Yemen, Sudan, Myanmar, Papua New Guinea, Lao People's Democratic Republic, Cambodia, Libya, Sierra Leone, Bolivia (Plurinational State of), Timor-Leste, and Turkmenistan. Frontier countries and territories with low-SDI include Somalia, Niger, Burundi, Democratic Republic of the Congo, and Nepal. High-SDI countries and territories with relatively high improvement potential considering their development level include Slovakia, Poland, Kuwait, Puerto Rico, and United Arab Emirates. Frontier analysis revealed the potential improvement space in reducing CHD burden across countries and territories. Despite limited resources, some low-SDI countries and territories, such as Nepal, have demonstrated remarkable capabilities in controlling diseases.

**Fig. 5:**
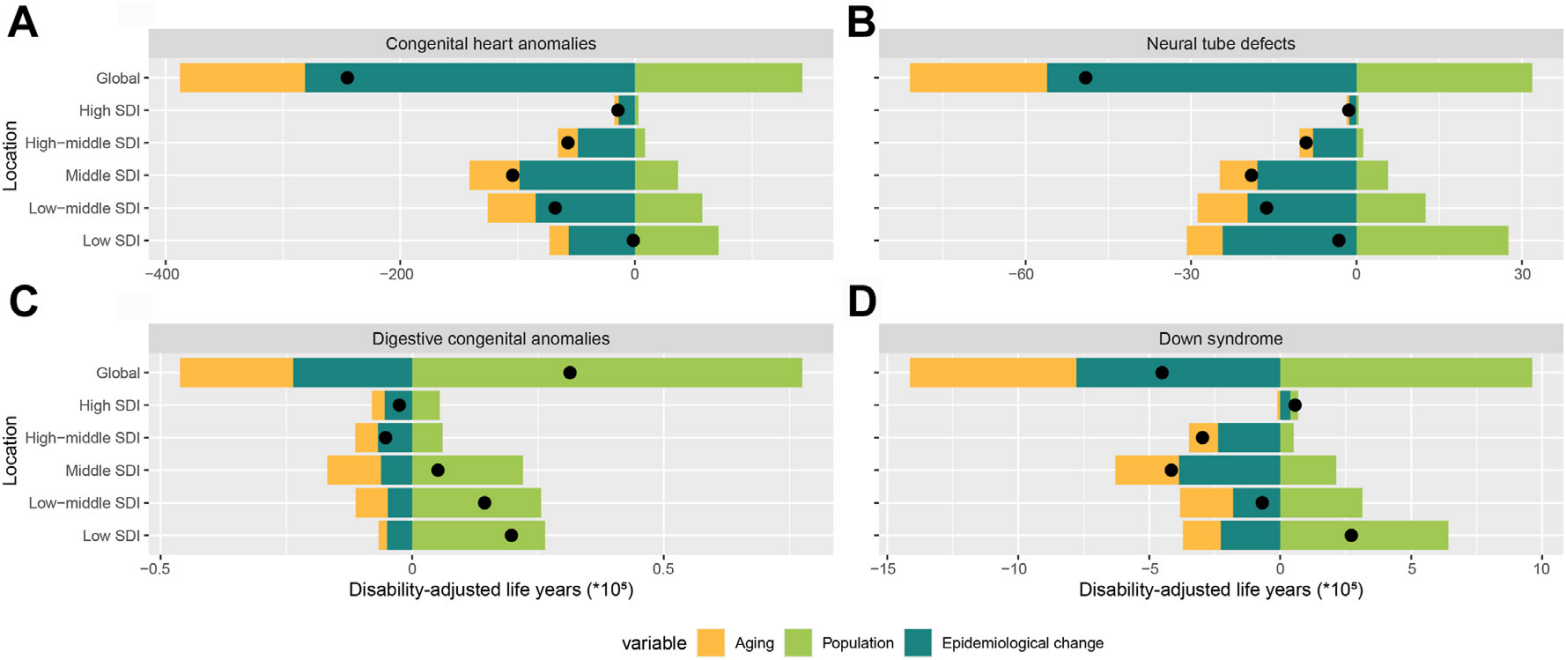
Population-level determinant changes in aging, population growth, and epidemiological changes for CHD (A), NTDs (B), DCAs (C), and DS (D) DALYs globally and in various SDI regions from 1990 to 2021. Black dots represent the total change contributed by all three components. A positive value for each component indicates a corresponding positive contribution in DALYs, and a negative value indicates a corresponding negative contribution in DALYs. CHD, congenital heart defects; NTDs, neural tube defects; DCAs, digestive congenital anomalies; DS, Down syndrome; SDI, sociodemographic index; DALYs, disability-adjusted life-years.

By performing decomposition analysis on the DALYs of CHD, this study evaluated the impact of factors such as aging, population growth, and epidemiological changes on CHD epidemiology from 1990 to 2021 (Fig. 5A and Table S7). Overall, the CHD DALYs overall difference showed a downward trend globally and in all SDI regions, with the most prominent decline observed in middle-SDI regions. Globally, aging and epidemiological changes contributed 43.51% and 114.69% respectively to the reduction in disease burden. The impact of population growth varied across different SDI regions, being −5231.72% in low-SDI regions; −84.62%, −35.36%, −15.11%, and −21.06% in low-middle, middle, middle–high, and high-SDI regions respectively. The contribution of aging was more pronounced in lower-SDI regions, specifically low-SDI (1206.67%), low-middle-SDI (60.07%), middle-SDI (40.97%), middle-high-SDI (30.01%), and high-SDI (25.87%). Epidemiological changes contributed to the reduction in global disease burden, especially in low-SDI regions, at 4125.06%. Globally, epidemiological changes were associated with a reduction in CHD burden, while population growth was linked to an increase in burden, especially in low-SDI regions.

**Fig. 6:**
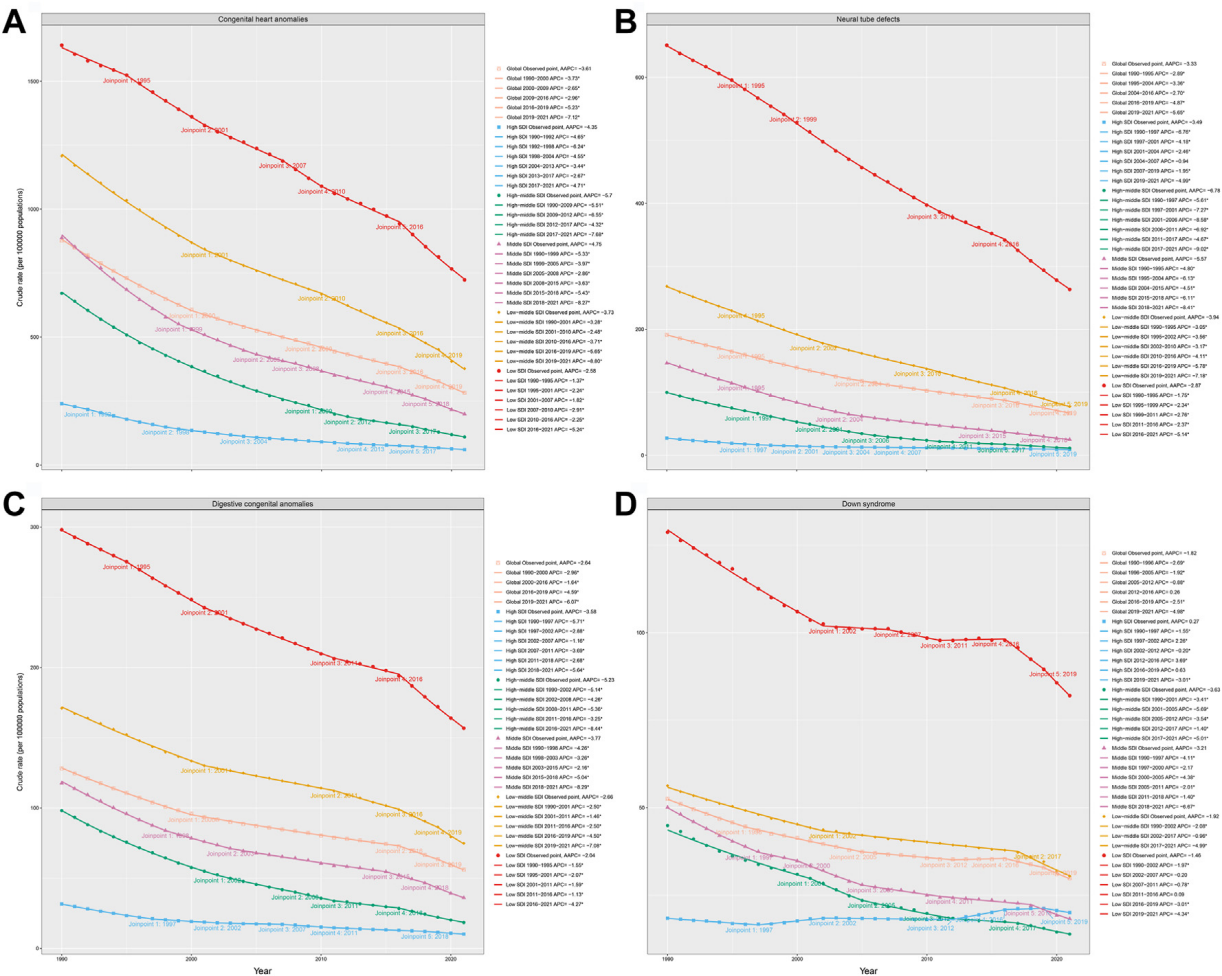
Temporal trend changes in ASDR for CHD (A), NTDs (B), DCAs (C), and DS (D) globally and in various SDI regions from 1990 to 2021 based on the Joinpoint regression model. ∗*p* < 0.05; CHD, congenital heart defects; NTDs, neural tube defects; DCAs, digestive congenital anomalies; DS, Down syndrome; SDI, sociodemographic index; ASDR, age-standardized disability-adjusted life years rate.

We used the Joinpoint regression model to analyze the temporal trend of CHD DALYs burden globally and across different SDI regions, finding differences in DALYs burden trends among SDI regions (Fig. 6A, Table S8). Globally, the CHD DALYs burden showed a downward trend (AAPC = −3.61), with a relatively gradual decline from 1990–2000 (APC = −3.73), 2000–2009 (APC = −2.65), to 2009–2016 (APC = −2.96), but an accelerated decline from 2016–2019 (APC = −5.23) to 2019–2021 (APC = −7.12). Except for low-SDI regions, all other regions generally showed a trend of an initially slowing and then accelerating decline. The trend in low-SDI regions was more complex, and the AAPC was the lowest among SDI regions (AAPC = −2.58). It showed a slow declining trend from 1990 to 1995 (APC = −1.37), followed by different phases of decline, namely 1995 to 2001 (APC = −2.24), 2001 to 2007 (APC = −1.82), 2007 to 2010 (APC = −2.91), 2010 to 2016 (APC = −2.25), and 2016 to 2021 (APC = −5.24). For the 95% CI of APC and specific *p*-values, please refer to Table S8.

**Fig. 7:**
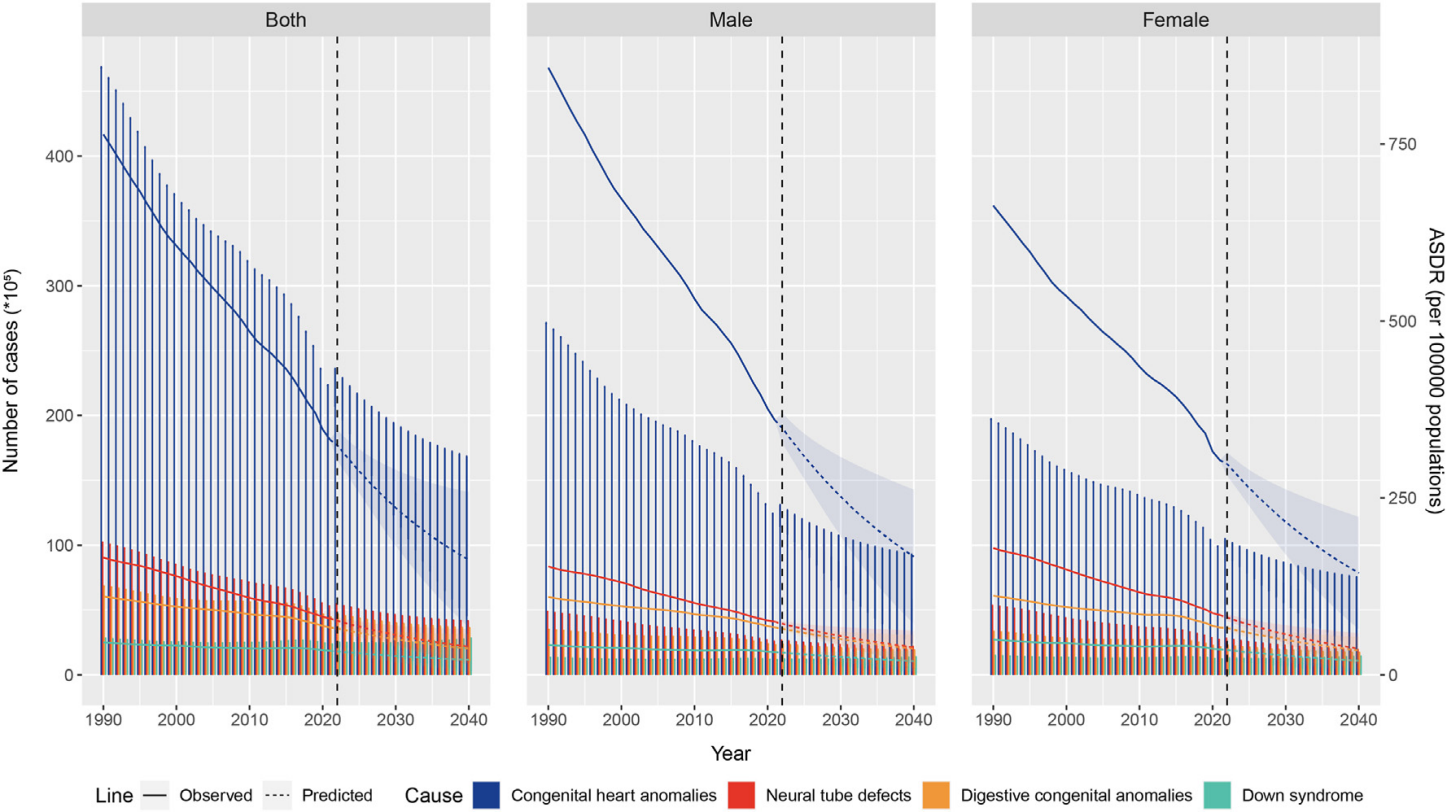
Temporal trend in the number of DALYs cases and ASDR for CHD, NTDs, DCAs, and DS from 1990 to 2040 for both males and females. Solid lines represent observed ASDR, and dashed lines represent ASDR predicted by the BAPC model. ASDR, age-standardized disability-adjusted life years rate; CHD, congenital heart defects; NTDs, neural tube defects; DCAs, digestive congenital anomalies; DS, Down syndrome; BAPC, Bayesian age-period-cohort.

When we used the BAPC model to predict the disease burden of CHD from 2021 to 2040, the DALYs predictions for different diseases showed heterogeneity (Fig. 7 and Table S9). The global CHD disease burden will continue to decline, with the number of all-age cases dropping to 10,682,983.45 (95% CI: 4,509,261.76–16,856,705.13) by 2040, and ASDR dropping to 162.8 (95% CI: 67.27–258.34). However, CHD will still be the most burdensome CBDs. Compared to 2021, the number of all-age cases will decrease by 52.12%, and the ASDR will decrease by 52.84%. Specifically, by sex, the number of all-age cases in males will decrease by 52.78%, and ASDR will decrease by 55.29%; the decline in females will be less, with the number of all-age cases decreasing by 51.28% and ASDR by 54.23%. These predictions indicate that while the global CHD burden will decline, notable sex and regional disparities will persist.

### Neural tube defects

In 1990, the ASDR of NTDs showed differences across various aspects (Table S3). The ASDR for females was higher than for males, indicating a heavier disease burden (males: 148.62; 95% UI: 111.02–210.81; females: 175.8; 95% UI: 91.77–244.92). When categorized by SDI regions, low-SDI regions exhibited the highest disease burden (331.38; 95% UI: 185.79–477.62). Among GBD regions, Central Sub-Saharan Africa had the heaviest burden (403.34; 95% UI: 213.8–577.52). Specifically, among the 204 countries and territories, Sudan exhibited the highest ASDR (693.61; 95% UI: 271.21–1271.81), while China recorded the highest number of all-age NTD cases (1,492,541.85; 95% UI: 1,081,129.64–1,887,283.03). Taiwan (Province of China) had the lowest ASDR (7.89; 95% UI: 6.90–9.02), and Tokelau demonstrated the lowest number of all-age NTD cases (1.15; 95% UI: 0.64–1.77). In 1990, the burden of NTDs showed notable differences across sexes and SDI regions.

In 2021, the ASDR for females remained higher than for males, although the gap had decreased (males: 78.6; 95% UI: 61.42–103.6; females: 88.07; 95% UI: 64.32–117.48). Low-SDI regions continued to have the heaviest burden among SDI regions, but the GBD region with the heaviest burden shifted to Eastern Sub-Saharan Africa (low-SDI: 175.35; 95% UI: 131.63–224.19; Eastern Sub-Saharan Africa: 192.6; 95% UI: 126.36–272.07). Among countries and territories, South Sudan exhibited the highest ASDR (351.62; 95% UI: 194.14–579.97), while Nigeria recorded the highest number of all-age NTD cases (798,459.56; 95% UI: 467,807.31–1,168,733.07). Taiwan (Province of China) continued to have the lowest ASDR (2.67; 95% UI: 2.11–3.3), and the Cook Islands demonstrated the lowest number of all-age NTD cases (0.82; 95% UI: 0.38–1.55), showing improvement. In 2021, although the burden of NTDs had decreased and the sex gap had narrowed, low-SDI regions remained the most affected.

In terms of the burden of NTDs, we observed significant absolute and relative inequalities associated with the SDI, with countries and territories having lower SDI disproportionately bearing a higher burden (Fig. 3C and D and Tables S4 and S5, *p* < 0.05). As shown by the slope index of inequality, the gap in the DALYs rate between the highest and lowest SDI countries and territories decreased from −302.28 (95% CI: −333.67 to −270.89) in 1990 to −139.19 (95% CI: −155.51 to −122.88) in 2021. The concentration index was −0.29 (95% CI: −0.33 to −0.24) in 1990, but increased to −0.44 (95% CI: −0.50 to −0.39) in 2021. The absolute health inequality in NTDs has decreased over the past 30 years, but relative inequality has worsened. This reflects the unequal distribution of the NTDs burden between low and high SDI regions.

Using data from 1990 to 2021, and based on ASDR and SDI, frontier analysis was conducted to explore the potential improvement space of ASDR for NTDs, considering national and regional development levels (Fig. 4C and D and Table S6). The 15 countries and territories with the largest actual differences in potential improvement (effective difference range: 267.11–142.18) include South Sudan, Central African Republic, United Republic of Tanzania, Nigeria, Comoros, Sierra Leone, Madagascar, Malawi, Zambia, Uganda, Mozambique, Côte d'Ivoire, Benin, Guinea, and Eritrea. Frontier countries and territories with low-SDI include Somalia, Niger, Nepal, Solomon Islands, and Gambia. High-SDI countries and territories with relatively high improvement potential considering their development level include Puerto Rico, Greenland, United States of America, United Arab Emirates, and Ireland. The analysis suggests that low-SDI countries and territories, such as Somalia and Niger, have performed relatively well in controlling the disease burden despite limited resources. Conversely, high-SDI countries and territories, such as Puerto Rico and Ireland, still have room for further improvement despite their development levels.

By performing decomposition analysis on the DALYs of NTDs, this study evaluated the impact of factors such as aging, population growth, and epidemiological changes on NTDs epidemiology from 1990 to 2021 (Fig. 5B and Table S7). Overall, the NTDs DALYs overall difference showed a downward trend globally and in all SDI regions, with the most prominent decline observed in middle-SDI regions. Globally, aging and epidemiological changes contributed 50.65% and 114.24%, respectively, to the reduction in disease burden. The impact of population growth varied across different SDI regions, being −861.14% in low-SDI regions; −76.66%, −30.05%, −13.24%, and −26.47% in low-middle, middle, middle–high, and high-SDI regions, respectively. The contribution of aging was more pronounced in lower-SDI regions, specifically low-SDI (203.29%), low-middle-SDI (55.53%), middle-SDI (35.75%), middle-high-SDI (27.06%), and high-SDI (33.90%). Epidemiological changes contributed to the reduction in global disease burden, especially in low-SDI regions, at 757.85%. The decomposition analysis shows that epidemiological changes are the primary factor driving the reduction in global NTDs burden, especially in low-SDI regions. In contrast, population growth is a key factor hindering the reduction in disease burden in low-income regions.

We used the Joinpoint regression model to analyze the temporal trend of NTDs DALYs burden globally and across different SDI regions, finding differences in DALYs burden trends among SDI regions (Fig. 6B, Table S8). Globally, the NTDs DALYs burden showed a downward trend (AAPC = −3.33), with a relatively gradual decline from 1990–1995 (APC = −2.89), 1995–2004 (APC = −3.36), to 2004–2016 (APC = −2.70), but an accelerated decline from 2016–2019 (APC = −4.87) to 2019–2021 (APC = −5.65). High-middle-SDI regions showed the most pronounced decline (AAPC = −6.78). Specifically, the decline accelerated from 1990–1997 (APC = −5.61), 1997–2001 (APC = −7.27), to 2001–2006 (APC = −8.58), then slowed from 2006–2011 (APC = −6.92) to 2011–2017 (APC = −4.67), but accelerated again from 2017 to 2021 (APC = −9.02). Low-SDI regions showed the slowest decline, generally displaying a trend of initially slowing and then accelerating decline (AAPC = −2.87). Specifically, the decline accelerated from 1990–1995 (APC = −1.75), 1995–1999 (APC = −2.34), to 1999–2011 (APC = −2.76), then slowed briefly from 2011 to 2016 (APC = −2.37), but accelerated again from 2016 to 2021 (APC = −5.14). The burden in low-SDI regions has declined more slowly.

We used the BAPC model to predict the disease burden of NTDs from 2021 to 2040 (Fig. 7 and Table S9). The global NTDs disease burden will continue to decline, with the number of all-age cases dropping to 2,570,987.29 (95% CI: 990,614.91–4,151,359.67) by 2040, and ASDR dropping to 39.95 (95% CI: 15.1–64.81). Compared to 2021, the number of all-age cases will decrease by 51.44%, and the ASDR will decrease by 51.97%. Specifically, by sex, the number of all-age cases in males will decrease by 47.42%, and ASDR will decrease by 50.31%; the decline in females will be much greater, with the number of all-age cases decreasing by 55.26% and ASDR by 57.95%. The number of all-age cases and ASDR in males will surpass those in females by 2040, indicating a greater disease burden in males. The global burden of NTDs will continue to decline, with the burden in males expected to surpass that of females by 2040.

### Digestive congenital anomalies

In 1990, the ASDR of DCAs showed differences across various aspects (Table S3). The ASDR for females was higher than for males, indicating a heavier disease burden (males: 106.8; 95% UI: 59.93–180.53; females: 109.83; 95% UI: 50.58–182.67). When categorized by SDI regions, low-SDI regions exhibited the highest disease burden (149.39; 95% UI: 50.91–259.22). Among GBD regions, North Africa and the Middle East had the heaviest burden (180.23; 95% UI: 64.81–352.17). Specifically, among the 204 countries and territories, Liberia had the highest ASDR (308.3; 95% UI: 59.34–580.52), while China recorded the highest number of all-age DCA cases (1,281,060.14; 95% UI: 688,871.8–2,322,525.41). American Samoa had the lowest ASDR (11.03; 95% UI: 5.3–31.95), and Tokelau demonstrated the lowest number of all-age DCA cases (0.33; 95% UI: 0.16–1.02). In 1990, the burden of DCAs showed notable differences across sexes, regions, and SDI levels, with the burden being the heaviest in low-SDI regions. Females had a slightly higher ASDR than males.

In 2021, although the disease burden decreased, the ASDR for males became higher than for females (males: 70.66; 95% UI: 48.99–96.31; females: 70.2; 95% UI: 45.77–94.1). Low-SDI regions still had the heaviest burden among SDI regions, but the GBD region with the heaviest burden shifted to Western Sub-Saharan Africa (low-SDI: 103.58; 95% UI: 69.24–141.71; Western Sub-Saharan Africa: 140.99; 95% UI: 88.96–192.78). Among countries and territories, Burkina Faso had the highest ASDR (174.49; 95% UI: 94.51–265.06), while India recorded the highest number of all-age DCA cases (649,338.01; 95% UI: 376,852.44–1,091,616.22). The Northern Mariana Islands had the lowest ASDR (3.27; 95% UI: 1.68–8.53), and Tokelau had the lowest number of all-age DCA cases (0.5; 95% UI: 0.18–1.89). In 2021, although the global burden of DCAs had decreased, the ASDR for males surpassed that of females, and low-SDI regions remained the hardest hit.

In terms of the burden of DCAs, we observed significant absolute and relative inequalities associated with SDI, with countries and territories having lower SDI disproportionately bearing a higher burden (Fig. 3E and F and Tables S4–S5, *p* < 0.05). As shown by the slope index of inequality, the gap in the DALYs rate between the highest and lowest SDI countries and territories decreased from −110.48 (95% CI: −130.25 to −90.72) in 1990 to −80.37 (95% CI: −91.12 to −69.61) in 2021. The concentration index was −0.12 (95% CI: −0.16 to −0.09) in 1990, but increased to −0.24 (95% CI: −0.27 to −0.21) in 2021. While absolute health inequalities for DCAs have decreased, relative inequalities have worsened.

Using data from 1990 to 2021, and based on ASDR and SDI, frontier analysis was conducted to explore the potential improvement space of ASDR for DCAs considering national and regional development levels (Fig. 4E and F and Table S6). The 15 countries and territories with the largest actual differences in potential improvement (effective difference range: 155.38–108.4) include Bolivia (Plurinational State of), Haiti, Nigeria, Suriname, Sierra Leone, Venezuela (Bolivarian Republic of), Turkmenistan, Burkina Faso, Cameroon, Guinea, Benin, Myanmar, United Republic of Tanzania, South Sudan, and Mali. Frontier countries and territories with low-SDI include Somalia, Niger, Papua New Guinea, Solomon Islands, and Nepal. High-SDI countries and territories with relatively high improvement potential considering their development level include Bermuda, Slovakia, Greenland, Puerto Rico, and Kuwait. Low-SDI countries and territories such as Somalia and Niger have shown impressive results in controlling the burden of DCAs despite limited resources, while high-SDI countries and territories, such as Bermuda and Puerto Rico, still have room for improvement.

By performing decomposition analysis on the DALYs of DCAs, this study evaluated the impact of factors such as aging, population growth, and epidemiological changes on DCAs epidemiology from 1990 to 2021 (Fig. 5C and Table S7). Overall, the DCAs DALYs overall difference showed an upward trend globally and in low-SDI, low-middle-SDI, and middle-SDI regions, with the most prominent increase observed in low-SDI regions. Globally, aging and epidemiological changes contributed −71.78% and −75.41%, respectively, to the increase in disease burden. The impact of population growth varied across different SDI regions, being 429.33% in middle-SDI regions; 133.95%, 178.02%, −113.98%, and −213.76% in low, low-middle, middle–high, and high-SDI regions, respectively. The contribution of aging was more pronounced in lower-SDI regions, specifically low-SDI (−8.61%), low-middle-SDI (−44.24%), middle-SDI (−208.55%), middle-high-SDI (85.56%), and high-SDI (100.39%). Epidemiological changes contributed to the decrease in the global disease burden, especially in high-SDI regions, at 213.37%.

We used the Joinpoint regression model to analyze the temporal trend of DCAs DALYs burden globally and across different SDI regions, finding differences in DALYs burden trends among SDI regions (Fig. 6C, Table S8). Globally, the DCAs DALYs burden showed a downward trend (AAPC = −2.64), with a relatively gradual decline from 1990–2000 (APC = −2.96) to 2000–2016 (APC = −1.64), but an accelerated decline from 2016–2019 (APC = −4.59) to 2019–2021 (APC = −6.07). High-middle-SDI regions showed the most pronounced decline (AAPC = −5.23). The decline trend in this region fluctuated over different periods. Specifically, the APC was −5.14 from 1990 to 2002, −4.26 from 2002 to 2008, −5.36 from 2008 to 2011, −3.25 from 2011 to 2016, and accelerated to −8.44 from 2016 to 2021. Low-SDI regions showed the slowest decline, generally displaying a trend of initially slowing and then accelerating decline (AAPC = −2.04). Specifically, the decline accelerated from 1990–1995 (APC = −1.55) to 1995–2001 (APC = −2.07), then slowed from 2001–2011 (APC = −1.59) to 2011–2016 (APC = −1.13), but accelerated again from 2016 to 2021 (APC = −4.27). Joinpoint regression analysis indicates that DCAs burden decreased most notably in middle-SDI regions, while low-SDI regions exhibited the slowest decline.

We used the BAPC model to predict the disease burden of DCAs from 2021 to 2040 (Fig. 7 and Table S9). The global DCAs disease burden will continue to decline, with the number of all-age cases dropping to 2,347,464.16 (95% CI: 1,059,505.67–3,635,422.65) by 2040, and ASDR dropping to 37.17 (95% CI: 16.68–57.66). Compared to 2021, the number of all-age cases will decrease by 47.02%, and the ASDR will decrease by 47.23%. Specifically, by sex, the number of all-age cases in males will decrease by 45.68%, and ASDR will decrease by 47.78%; the decline in females will be greater, with the number of all-age cases decreasing by 48.45% and ASDR by 50.73%. These predictions suggest that by 2040, the global DCAs burden will decline notably, with males experiencing a slightly slower reduction than females.

### Down syndrome

In 1990, the ASDR of DS showed differences across various aspects (Table S3). The ASDR for females was higher than for males, indicating a heavier disease burden (males: 41.66; 95% UI: 25.97–89.26; females: 49.56; 95% UI: 20.79–122.99). When categorized by SDI regions, low-SDI regions exhibited the highest disease burden (71.55; 95% UI: 20.85–231.96). Among GBD regions, North Africa and the Middle East had the heaviest burden (100.46; 95% UI: 41.55–245.37). Specifically, among the 204 countries and territories, Afghanistan had the highest ASDR (156.03; 95% UI: 23.56–554.38), while China recorded the highest number of all-age DS cases (654,145.15; 95% UI: 436,029.99–1,028,444.24). Taiwan (Province of China) had the lowest ASDR (2.72; 95% UI: 1.97–3.71), and Niue demonstrated the lowest number of all-age DS cases (1.47; 95% UI: 0.82–2.31). In 1990, the DS burden varied notably across sexes, regions, and SDI levels, with females having a slightly higher ASDR than males.

In 2021, the ASDR for females was still higher than for males, although the gap decreased (males: 33.35; 95% UI: 24.54–51.63; females: 37.19; 95% UI: 26.18–69.84). Low-SDI regions still had the heaviest burden among SDI regions, but the GBD region with the heaviest burden shifted to Oceania (low-SDI: 57.85; 95% UI: 34.1–120.9; Oceania: 122.16; 95% UI: 42.87–217.51). Among countries and territories, Tokelau showed the highest ASDR (329.15; 95% UI: 171.44–500.56), while Nigeria recorded the highest number of all-age DS cases (268,795.7; 95% UI: 135,861.6–652,061.05). Uzbekistan had the lowest ASDR (5.52; 95% UI: 3.66–10.88), and the Cook Islands demonstrated the lowest number of all-age DS cases (2.38; 95% UI: 0.52–5.08). In 2021, despite the overall reduction in the global DS burden, low-SDI regions still had the highest burden.

In terms of the burden of DS, we observed significant absolute and relative inequalities associated with SDI, with countries and territories having lower SDI disproportionately bearing a higher burden (Fig. 3A and B and Tables S4 and S5, *p* < 0.05). As shown by the slope index of inequality, the gap in the DALYs rate between the highest and lowest SDI countries and territories decreased from −54.15 (95% CI: −64.14 to −44.16) in 1990 to −37.89 (95% CI: −45.72 to −30.05) in 2021. The concentration index was −0.11 (95% CI: −0.16 to −0.06) in 1990, but increased to −0.15 (95% CI: −0.20 to −0.11) in 2021. While absolute inequalities in DS burden have improved, relative inequalities have worsened.

Using data from 1990 to 2021, and based on ASDR and SDI, frontier analysis was conducted to explore the potential improvement space of ASDR for DS, considering national and regional development levels (Fig. 4G and H and Table S6). The 15 countries and territories with the largest actual differences in potential improvement (effective difference range: 325.42–70.51) include Tokelau, Niue, Papua New Guinea, Libya, Kiribati, Haiti, Algeria, South Sudan, Fiji, Nauru, Yemen, Brunei Darussalam, Palau, Burkina Faso, and Nigeria. Frontier countries and territories with low-SDI include Somalia, Niger, Ethiopia, Nepal, and Timor-Leste. High-SDI countries and territories with relatively high improvement potential include the United Arab Emirates, Finland, Iceland, Netherlands, and Switzerland. Frontier analysis shows that low-SDI countries such as Somalia and Niger performed well in managing the DS burden despite limited resources, while high-SDI countries like Finland and Switzerland still have potential for further improvement.

By performing decomposition analysis on the DALYs of DS, this study evaluated the impact of factors such as aging, population growth, and epidemiological changes on DS epidemiology from 1990 to 2021 (Fig. 5D and Table S7). Overall, the DS DALYs overall difference showed a downward trend globally and in low-middle-SDI, middle-SDI, and high-middle-SDI regions, with the most notable decline observed in middle-SDI regions. Globally, aging and epidemiological changes contributed 140.84% and 172.56%, respectively, to the reduction in disease burden. The impact of population growth varied across different SDI regions, being −459.97% in low-middle-SDI regions; 236.47%, −51.41%, −17.43%, and 51.69% in low, middle, middle–high, and high-SDI regions, respectively. The contribution of aging was prominent in low-SDI (−53.13%), low-middle-SDI (296.82%), middle-SDI (58.48%), middle-high-SDI (37.53%), and high-SDI (−19.88%) regions. Epidemiological changes contributed to the reduction in global disease burden, especially in low-middle-SDI regions, at 263.15%. Decomposition analysis highlights aging and epidemiological changes as the main drivers of global DS burden reduction, especially in low-middle-SDI regions.

We used the Joinpoint regression model to analyze the temporal trend of DS DALYs burden globally and across different SDI regions, finding differences in DALYs burden trends among SDI regions (Fig. 6D, Table S8). Globally, the DS DALYs burden showed a downward trend (AAPC = −1.82), with a gradually slowing decline from 1990–1996 (APC = −2.69), 1996–2005 (APC = −1.92), to 2005–2012 (APC = −0.88), but an increase in disease burden from 2012 to 2016 (APC = 0.26). However, the burden decreased again and accelerated from 2016–2019 (APC = −2.51) to 2019–2021 (APC = −4.98). Except for high-SDI regions (AAPC = 0.27), the DS burden decreased to varying degrees across all SDI regions. The DS burden in high-SDI regions showed a fluctuating trend, initially decreasing from 1990 to 1997 (APC = −1.55), then temporarily increasing from 1997 to 2002 (APC = 2.26). The burden decreased again from 2002 to 2012 (APC = −0.20), increased between 2012 and 2016 (APC = 3.69), followed by a slight increase from 2016 to 2019 (APC = 0.63). The burden then decreased again from 2019 to 2021 (APC = −3.01). Low-SDI regions also experienced a phase of increase (AAPC = −1.46). Specifically, the burden fluctuated and declined from 1990–2002 (APC = −1.97), 2002–2007 (APC = −0.20), to 2007–2011 (APC = −0.78). The burden briefly increased from 2011 to 2016 (APC = 0.09), but further declined from 2016 to 2019 (APC = −3.01) and from 2019 to 2021 (APC = −4.34). Joinpoint regression analysis suggests that while the global DS burden generally declined, high-SDI and low-SDI regions exhibited fluctuations.

We used the BAPC model to predict the disease burden of DS from 2021 to 2040 (Fig. 7 and Table S9). The global DS disease burden will continue to decline, with the number of all-age cases dropping to 1,540,474.46 (95% CI: 263,345.42–2,822,648.83) by 2040, and ASDR dropping to 21.52 (95% CI: 3.04–40.08). Compared to 2021, the number of all-age cases will decrease by 34.53%, and the ASDR will decrease by 38.86%, showing the smallest decline among the four types of CBDs. Specifically, by sex, the number of all-age cases in males will decrease by 32.73%, and ASDR will decrease by 41.56%; the decline in females will be greater, with the number of all-age cases decreasing by 36.24% and ASDR by 46.46%. The number of all-age cases in males will surpass that of females. The predictions suggest that while the global DS burden will continue to decline, the reduction will be relatively modest, with males projected to carry a higher burden than females by 2040.

## Discussion

CBDs are a major challenge to global public health, with their lifelong impact on patients causing a tremendous disease burden and attracting substantial research attention. Our study spans thirty years and predicts the disease burden up to 2040. It includes data from all age groups across five SDI regions, 21 GBD regions, and 204 countries and territories, using various analytical methods to describe the disease burden across different populations, time periods, and geographic spaces. The study innovatively uses GBD 2021 data to assess, for the first time, the temporal trends and health inequalities in the selected CBDs of interest. It identifies key years of substantial changes in disease indicators and evaluates the impact of socioeconomic development levels on cross-national differences in disease burden. Traditional analytical methods face limitations when dealing with the nonlinear, multivariable, and high-dimensional nature of GBD 2021 data. Therefore, this study adopted more advanced and suitable analytical approaches to ensure a thorough analysis and accurate interpretation of the data. This study not only provides a new perspective on understanding the global burden of congenital anomalies but also offers valuable references for public health policymakers to address this challenge.

This study selected specific CBDs for analysis—CHD, NTDs, DCAs, and DS—primarily due to their substantial public health importance globally, especially in low-SDI regions. By extending the study period to 2040, we aim to forecast future disease burden trends, thereby offering long-term strategic guidance for policymakers. The selection of these diseases reflects the ongoing global public health imperative to reduce the incidence of congenital birth defects and underscores the urgent need to optimize healthcare resource allocation and develop effective interventions on a worldwide scale.

Based on our research findings, it is clear that from 1990 to 2021 and into the foreseeable future, the global burden of CBDs has decreased. This reduction is attributed not only to advancements in diagnostic technology and improvements in treatment plans but also to the crucial management of CBD risk factors and the screening of high-risk individuals, consistent with previous studies.^12^, ^13^, ^14^, ^15^, ^16^ Due to the high mortality rate, surgical complexity, and lifelong impact on patients, health inequalities resulting from imbalanced distribution of medical resources and lack of health education are particularly prominent in the global disease burden of CBDs.^17^, ^18^, ^19^, ^20^ Multiple reasons such as nutritional deficiencies, intrauterine infections, teratogen exposure, limited access to prenatal diagnosis, and low rates of pregnancy termination after prenatal diagnosis of congenital anomalies in low-development-level countries contribute to the heavy burden of congenital anomalies, with health inequalities increasing over the past thirty years.^21^

The global incidence of CHD is influenced by a complex interplay of genetic and environmental factors. Studies have shown that certain genetic syndromes, such as DiGeorge syndrome and Williams-Beuren syndrome, are closely associated with specific types of CHD, while familial CHD may follow inheritance patterns that increase the risk in offspring.^22^^,^^23^ The complexity of these genetic factors, especially in low-income regions with limited access to genetic screening, complicates prevention efforts. Additionally, maternal metabolic conditions during pregnancy, such as diabetes and obesity, or exposure to environmental toxins like tobacco and organic solvents, increase the risk of CHD, particularly in low- and middle-income countries.^24^ In these areas, inadequate prenatal health education and limited screening capabilities hinder early diagnosis and treatment, further exacerbating the CHD burden. While high-income countries have notably reduced CHD mortality through advanced fetal ultrasound and genetic screening, the severe shortage of screening and surgical resources in low-SDI countries leaves a substantial burden.^25^ The gap in postoperative management and long-term care is also notable in these regions, where multidisciplinary collaboration systems—common in high-income countries, encompassing cardiac surgery, pediatric cardiology, rehabilitation, and psychological support—are lacking, which negatively impacts patient survival and recovery. Addressing this global health challenge, particularly in resource-limited areas, requires strengthening early screening, improving surgical access, enhancing postoperative care, and promoting the equitable distribution of medical resources through international cooperation.

NTDs are closely associated with folic acid deficiency, and high-income countries have notably reduced the incidence of NTDs through folic acid fortification programs. However, the burden remains high in low-SDI regions due to insufficient implementation of these preventive measures. Consistent with previous reports, inadequate folic acid intake in low-income countries is a major cause of NTDs, with the disease having a mortality rate as high as 100% in rural areas, compared to only 10% in developed regions.^26^ The disparity in NTDs burden between low- and high-SDI regions emphasizes the critical importance of promoting folic acid supplementation and fortification in low-income regions to address the root cause of these anomalies. In our study, regions with higher socioeconomic development saw dramatic declines in NTDs due to improved maternal nutrition and awareness programs, while low-SDI regions exhibited a much slower decline, indicating the need for sustained public health efforts.

DCAs represent a complex interplay of genetic and environmental factors. Our findings indicated a slower rate of improvement in low-SDI regions compared to high-SDI regions, where advanced medical care and early intervention strategies have successfully reduced the burden. In low-income areas, the lack of access to prenatal screening and postnatal care has compounded the disease burden of DCAs, with higher rates of preventable complications and long-term disabilities. The concentration index for DCAs also showed a worsening trend in health inequalities, underscoring the need for resource allocation to provide comprehensive screening and postnatal services in low-SDI regions. It is noteworthy that in 1990, the ASDR for DCAs was lower in males than in females. However, this trend reversed in 2021, with the ASDR for males exceeding that for females. The reduction in disease burden for males was slower than for females, and this change may be related to multiple factors. First, the genetic susceptibility of males to congenital diseases may have changed over the past thirty years.^27^ With economic improvement, males may be more exposed to factors that increase disease risk, such as unhealthy diets, lack of exercise, and high stress, in their dietary habits and lifestyles.^28^, ^29^, ^30^ These findings highlight the need to understand the specific factors contributing to the disease burden in both males and females. Public health interventions should be inclusive, promoting healthy lifestyle education, early screening, and timely interventions for all individuals, regardless of sex. By addressing the unique needs and risk factors of both sexes, we can more effectively reduce the global burden of DCAs.^31^^,^^32^ Moreover, affected families often are at the core of patient care, bearing long-term caregiving responsibilities. Supporting these families through education, counseling, and access to healthcare resources is essential in managing the long-term impact of CBDs on both patients and their families.

The burden of DS is closely linked to advanced maternal age, a risk factor more prevalent in high-income countries where family planning tends to occur later in life. While early prenatal screening and selective abortion have contributed to the reduction of DS births in high-SDI regions, low-SDI regions continue to face notable challenges due to inadequate screening infrastructure and limited prenatal counseling services. Our analysis highlighted that in regions with insufficient prenatal screening, the ASDR for DS remains high, accentuating the need for improving early diagnostic and counseling capabilities in low-resource settings. Additionally, the concentration of health inequalities in DS was evident from concentration index trends, signaling that despite some advancements, substantial disparities persist in care access and outcomes for individuals with DS.

As global health continues to evolve, assessing the complexity of health inequalities across countries and territories becomes increasingly important. Traditional linear regression models have limitations in capturing this complexity. While they can assess disparities between groups, they often fall short of capturing the intricate distribution of inequality across various sociodemographic strata. In contrast, the SII and concentration index, as defined by the WHO, provide more robust measures by accounting for both population size and the relative positioning of subpopulations. These methods offer a more nuanced analysis of inequality, making them particularly suitable for GBD 2021 data, where pronounced regional and developmental differences require refined assessments to accurately capture disparities. Our study results indicate that, compared to 1990, the 2021 health inequality analysis for CHD, NTDs, DCAs, and DS shows that while absolute health inequality in the global burden of these conditions has decreased, relative inequality has widened. Particularly for DCAs, the concentration index, already high at −0.29 in 1990 (95% CI: −0.33 to −0.24), increased to −0.44 in 2021 (95% CI: −0.50 to −0.39). This indicates that although advancements in medical standards have generally improved disease burden, these improvements are likely concentrated in high-development-level countries.^33^ In these countries, advanced medical technology and high-quality health services noticeably reduce the disease burden, while countries and territories with low levels of development, due to resource shortages and inadequate medical services, have not achieved the same level of improvement.^34^ Therefore, it is crucial to promote the equitable distribution of medical resources globally, strengthen support for low-development-level countries and territories, and improve basic health infrastructure and medical services. By identifying trends in disease burden and health inequalities, policymakers can more effectively optimize resource allocation, enhance health education, and formulate targeted public health interventions, especially in areas with a high disease burden.

This study also conducted decomposition analysis based on three driving factors for CHD, NTDs, DCAs, and DS. Decomposition analysis offers a more detailed understanding of changes in disease burden by dividing them into distinct contributing factors such as aging, population growth, and epidemiological shifts. This approach allows for the identification of the key drivers behind these overall trends. In contrast, traditional linear models provide a broader view of associations but lack the ability to separate and quantify the individual contributions of these components to changes in disease burden. The results showed that low-SDI regions exhibited the most pronounced observation for improvement in disease burden. These regions substantially influenced by aging, population growth, and epidemiological changes, indicating that despite the high current disease burden, improvements can be achieved through effective public health interventions and resource investment. Combined with the temporal trend analysis of CHD, NTDs, DCAs, and DS, the disease burden in low-SDI regions declined slowly and showed fluctuations. First, low-SDI regions have a large baseline and high initial disease burden, so the absolute reduction is relatively small. Secondly, these regions have less health investment, lack trained professionals to identify or handle these birth anomalies, leading to treatment delays, difficulty accessing surgical facilities, and limited awareness of treatment. The number of pediatric cardiac surgeons per million people in high- and low-development-level countries may differ by more than 50 times, and the enormous disparity in medical resources leads to persistent health inequalities.^35^^,^^36^ Future research should prioritize investigating the specific causes and influencing factors of health inequalities, particularly in low-SDI regions. A deeper exploration of the CBDs burden across different sexes, age groups, and socioeconomic backgrounds will provide a clearer understanding of the mechanisms driving these disparities, thereby facilitating the development of more targeted public health strategies. Furthermore, research findings can inform policy decisions. Governments, in collaboration with international organizations, can promote data sharing, optimize resource allocation, and implement public health interventions, thereby more effectively controlling and reducing the global burden of congenital birth defects.

In addition, frontier analysis was applied to analyze the potential improvement space for CHD, NTDs, DCAs, and DS of interest in 204 countries and territories. Frontier analysis, unlike traditional regression models, does not simply assess associations between variables but aims to identify the theoretical minimum disease burden a country or territory could achieve based on its SDI level. This approach helps pinpoint underperforming countries by quantifying the gap between their current burden and the “frontier” of optimal performance. In contrast, traditional linear models focus primarily on existing relationships between variables and may overlook this latent improvement potential, as they do not account for the achievable benchmarks tied to SDI levels. Nepal, Somalia, and Papua New Guinea repeatedly appeared as frontier countries and territories with low-SDI, showing outstanding performance in improving disease burden. As reported in previous studies, Nepal has implemented several CBDs monitoring programs, prospectively collecting and analyzing the incidence of birth defects and related risk factors, providing a basis for birth defect prevention and management policies for Nepal and low-to middle-income populations.^37^^,^^38^ These countries and territories, despite limited resources, have performed exceptionally well in controlling multiple CBD burdens, and their policies and practices deserve in-depth study. Conversely, high-development-level countries and territories such as Finland, Iceland, and the Netherlands did not perform as expected in controlling CBDs burden. Although these countries lead in newborn screening and early intervention, they still need to improve multidisciplinary collaboration and long-term follow-up to ensure children with congenital defects receive ongoing medical support and improved quality of life.^39^^,^^40^ Despite differences in environmental pollution, nutritional status, and access to healthcare due to geographical location, and the potential impact of ethnic genetic susceptibility on disease burden, these countries and territories still need to further optimize and improve their health policy formulation and implementation.^41^

This study has several limitations that should be acknowledged. While GBD 2021 offers extensive coverage, issues related to incomplete or inconsistent data reporting across different countries and territories could impact the accuracy of our findings. The variability in data collection and reporting methods introduces additional uncertainties. Furthermore, the research analysis methods employed, such as Joinpoint regression analysis, which depends on the preset number of connection points, may introduce bias and influence the interpretation of trends. Although we employed multiple analytical approaches to capture global epidemiological changes in CBDs, each method has inherent limitations that could affect the results. Moreover, according to GBD 2021, the definition and classification standards of congenital birth defects require further refinement to enhance data accuracy and consistency, which will provide a solid foundation for developing more effective public health strategies.

In GBD 2021, the precision of disease burden estimates relies heavily on the sources and quality of data. Despite the use of advanced statistical models like DisMod-MR 2.1 to manage data heterogeneity, substantial differences in coverage, reporting mechanisms, classification standards, data collection periods, and quality control of birth defect surveillance systems across countries present ongoing challenges. In low- and middle-income countries, resource constraints often result in incomplete surveillance systems, leading to underreporting or misreporting of certain congenital defects. In contrast, developed countries may report lower disease burdens due to higher coverage and better data quality. These discrepancies can introduce systematic biases in comprehensive analyses, influencing overall global or regional disease burden estimates. Additionally, data heterogeneity might cause selection bias, particularly in regions with insufficient data coverage, potentially leading to either underestimation or overestimation of disease burdens and affecting the analysis of health inequalities. To mitigate these issues, we employed methods such as a rlm for the slope index of inequality analysis, aiming to control for data heterogeneity and extreme values. However, despite these adjustments, the heterogeneity of data sources might still impact the reliability of the analysis. Moreover, Simpson's paradox could lead to inconsistent results in stratified versus aggregated analyses. In the context of heterogeneous data sources and varying surveillance systems, this phenomenon could be amplified, affecting the robustness of the study's conclusions. Future research should aim to further standardize data collection and analysis methods, particularly in countries and territories with varying levels of development and surveillance capacity, and apply more precise corrective measures to address specific disparities in surveillance. Addressing these challenges requires a global effort to standardize birth defect surveillance systems, especially in low- and middle-income countries, to improve data collection and quality control, thereby enhancing the accuracy and reliability of disease burden estimates.

Moreover, although our data analysis may conflict somewhat with the BAPC model's assumption of temporal consistency in age, period, and cohort effects, the BAPC model remains advantageous in managing complex data and capturing long-term trends. The Bayesian framework of the BAPC model allows for the incorporation of prior information, providing flexible and robust estimates, particularly excelling in handling high-dimensional data and complex interactions between different time series. Compared to other methods, the BAPC model has distinct advantages in managing sparse data, providing uncertainty estimates, and addressing heterogeneity and complexity. Even with potential changes in period effects, the BAPC model still offers valuable insights into overall health trends. Therefore, we maintain that the BAPC model is a suitable tool for studying long-term trends in the disease burden of CBDs. Future research might consider introducing models that account for dynamic changes in period effects to further validate and extend our findings.

Based on the results of this study, future research could further explore the exact causes of CBDs burden and its influencing factors across different sexes, age groups, and socioeconomic backgrounds, revealing more details and mechanisms. The results of this study can provide scientific evidence for public health policy-making, especially in low-SDI regions, where it is necessary to increase health resource investment and health education, improve public health infrastructure, and reduce health inequalities in disease burden. Global cooperation and data sharing are crucial to reducing the burden of CBDs. With the improvement of screening methods and treatment approaches, the global burden of CBDs has improved. However, strengthened cooperation among countries to jointly address the challenge of congenital anomalies remains necessary. By sharing experiences and resources, studying comprehensive help for CBDs patients, and establishing a full lifecycle management model, the development of global health can be promoted. Additionally, future research could delve into the application of emerging technologies, such as artificial intelligence, telemedicine, and electronic medical record systems, in the prevention and control of congenital birth defects. These innovations hold tremendous potential, particularly in low- and middle-income countries, by enhancing the accuracy of disease screening and diagnosis, optimizing the allocation of medical resources, and ultimately reducing health disparities.^42^^,^^43^

The profound impact of early life origins on lifelong health is particularly evident in the study of CBDs. These defects typically manifest early in life and notably affect an individual's long-term development and health outcomes. Research indicates that adverse conditions during early life can contribute to the development of congenital diseases, resulting in lasting negative effects on quality of life.^44^ Therefore, improving early life health management, especially in low-SDI regions, through enhanced prenatal care, improved antenatal screening, and increased early diagnosis and intervention for birth defects, can effectively reduce the long-term burden of disease and promote lifelong health. The findings of this study further underscore this: the disease burden of CHD, NTDs, DCAs, and DS is more severe in low-SDI regions due to the lack of effective early interventions, leading to notable long-term health consequences. Implementing targeted public health measures during early life in these regions, particularly by improving nutritional supplementation and early screening efforts, has the potential to notably reduce future disease burden and mitigate health inequalities.^45^

Congenital birth defects have long been and will continue to be a crucial component of the global disease burden. While the global burden of CBDs has declined from 1990 to 2021, notable regional disparities persist, particularly in countries with lower development levels, where the burden remains substantial and necessitates prioritized intervention. These regional inequalities, alongside differences in disease drivers and sex-related variations, highlight the importance of developing targeted prevention and treatment strategies.

Beyond prevention and treatment, effective management of congenital birth defects is crucial for enhancing patient outcomes and quality of life. Early diagnosis, timely surgical interventions, and comprehensive rehabilitation programs are vital for managing these conditions, especially in resource-limited environments.^46^ Additionally, the integration of multidisciplinary care, including psychological support and long-term follow-up, can noticeably improve the quality of life for individuals affected by congenital birth defects. Future public health strategies should not only aim to reduce incidence rates but also emphasize the long-term care and management of affected individuals to ensure lasting health improvements. This holistic approach—combining prevention, treatment, and comprehensive management—will be essential in addressing the ongoing challenges of CBDs and enhancing the well-being of affected populations.

## Contributors

All authors made substantial contributions to the development of this manuscript. ZHB was responsible for conceptualization, methodology, data analysis, and drafting the original manuscript. JRH contributed to conceptualization, methodology, and drafting the original manuscript. JA was involved in visualization, investigation, and drafting the original manuscript. HW and XYD contributed to visualization and investigation. ZCY and XMM provided critical revisions and were responsible for reviewing and editing the manuscript. ZHB and JRH verified the underlying data. All authors have read and approved the final version of the manuscript.

## Data sharing statement

Data used for the analyses are publicly available from the Institute of Health Metrics and Evaluation, as described in the Methods section.

## Editor note

The Lancet Group takes a neutral position with respect to territorial claims in published maps and institutional affiliations.

## Declaration of interests

All authors hereby attest that they do not have any conflicts of interest related to this article.
