# Supplementary material for: The global, regional, and national patterns of change in the burden of congenital birth defects, 1990–2021: an analysis of the global burden of disease study 2021 and forecast to 2040
Source: eClinicalMedicine. 2024 Oct 4;77:102873. doi: 10.1016/j.eclinm.2024.102873 (PMC11474384; doi:10.1016/j.eclinm.2024.102873)
Supplement: Supplemental Data [file mmc1.docx]

**Supplementary Appendix**

**The Global, Regional, and National Patterns of Change in the Burden of Congenital Birth Defects, 1990 to 2021: An Analysis of the Global Burden of Disease study 2021 and forecast to 2040**

Zihao Bai^a,d^, Jingru Han^b,d^, Jia An^a,d^, Hao Wang^c^, Xueying Du^c^, Zhaocong Yang^c,*^, Xuming Mo^a,*^

^a^Nanjing Children’s Hospital, Clinical Teaching Hospital of Medical School, Nanjing University, Nanjing, 210008, China

^b^Guangzhou Key Laboratory of Basic and Applied Research of Oral Regenerative Medicine, Guangdong Engineering Research Center of Oral Restoration and Reconstruction, Affiliated Stomatology Hospital of Guangzhou Medical University, Guangzhou, 510182, China

^c^Department of Cardiothoracic Surgery, Children’s Hospital of Nanjing Medical University, Nanjing, 210008, China

^d^These authors contribute equally to this work.

***Corresponding Authors:**

Prof. Xuming Mo

Address: Nanjing Children’s Hospital, Clinical Teaching Hospital of Medical School, Nanjing University, Nanjing, 210008, China

E-mail: mohsuming15@njmu.edu.cn

Prof. Zhaocong Yang

Address: Department of Cardiothoracic Surgery, Children’s Hospital of Nanjing Medical University, Nanjing, 210008, China

E-mail: yangzhaocong34@126.com

**Contents**

[Appendix 1 Overview of data processing workflow and methodological enhancements in GBD 2021 3](#_Toc177978744)

[Appendix 2 List of International Classification of Diseases (ICD) codes mapped to CBDs in GBD 2021 7](#_Toc177978745)

[Table S1 DALYs of CBDs in 1990 and 2021 by different characteristics 8](#_Toc177978746)

[Table S2 Ranking of 11 types of DALYs of CBDs globally, in various SDI regions, and GBD regions 18](#_Toc177978747)

[Table S3 DALYs of CHD, NTDs, DCAs, and DS in 1990 and 2021 by different characteristics 31](#_Toc177978748)

[Table S4 Age-standardized DALYs rates of CHD, NTDs, DCAs, and DS with slope index of inequality analysis, categorized by global, SDI, and GBD regions 78](#_Toc177978749)

[Table S5 Age-standardized DALYs rates of CHD, NTDs, DCAs, and DS with concentration index analysis, categorized by global, SDI, and GBD regions 89](#_Toc177978750)

[Table S6 Age-standardized DALYs rates of CHD, NTDs, DCAs, and DS with frontier analysis across all countries and territories 100](#_Toc177978751)

[Table S7 Age-standardized DALYs rates of CHD, NTDs, DCAs, and DS with decomposition analysis, categorized by global and SDI regions 142](#_Toc177978752)

[Table S8 Joinpoint regression analysis results for CHD, NTDs, DCAs, and DS, categorized by global and SDI regions 144](#_Toc177978753)

[Table S9 Forecast of DALYs all-age cases and ASDR for CHD, NTDs, DCAs, and DS based on disease burden from 1990 to 2021, grouped by sex, projected to 2040 155](#_Toc177978754)

## Appendix 1 Overview of data processing workflow and methodological enhancements in GBD 2021

In GBD 2021, the data processing workflow is a crucial step to ensure the accuracy and consistency of global disease burden estimates. This process includes systematic adjustments to epidemiological data to correct for biases arising from differences in data sources, definitions, and measurement methods. These adjustments are implemented using complex statistical models such as MR-BRT and DisMod-MR 2.1, ensuring internal consistency in estimates of incidence, prevalence, years lived with disability (YLDs), years of life lost (YLLs), and disability-adjusted life years (DALYs) across different regions, ages, genders, and years. This process aims to minimize heterogeneity in the study results through standardization and correction steps.

**1. Identification and extraction of data**

In GBD 2021, estimating the nonfatal disease burden begins with the systematic identification and extraction of diverse data sources. This process includes not only systematic reviews of 77 diseases and risk factors but also the extraction of key data from surveys, disease registries, case reports, and hospitalization data. Particularly crucial are the survey data extracted from the Global Health Data Exchange (GHDx), such as the Demographic and Health Surveys (DHS), which capture a wide range of health outcomes across different regions and populations. The estimation of DALYs in GBD 2021 is based on 100,983 data sources, of which 19,189 were newly added in 2021. These sources include 75,459 data sources for nonfatal causes, covering incidence, prevalence, and other epidemiological indicators. Data sources for fatality estimation include death registrations, verbal autopsies, registries, surveys, and police or surveillance data, covering all countries and regions globally. GBD research efforts incorporated systematic reviews, proactive data collection, and contributions from a global network of collaborators to include as much data as possible from around the world.

**2. Data processing and heterogeneity control**

In GBD 2021, adjustments were made to epidemiological data known to have biases, such as those using alternative case definitions or measurement methods. These adjustments were made using correction factors estimated by MR-BRT (Meta-regression—Bayesian, regularized, trimmed), a collection of statistical models including linear and nonlinear mixed-effects models. The input data included paired estimates of two case definitions or measurement methods for the same age, sex, region, and year. MR-BRT also controlled for heterogeneity through network meta-regression and performed sex-splitting for inputs not reported by sex, and age-sex splitting for data not reported by either. These processes ensured data standardization and consistency, reducing heterogeneity issues caused by varying data sources and definitions. Additionally, input data spanning more than 25 years were disaggregated into finer age-specific estimates using alternative age patterns estimated from other available data sources.

**3. Epidemiological estimation and YLD calculation**

The prevalence and incidence estimates for most diseases and injuries were derived using DisMod-MR 2.1 (Disease Modeling Meta-Regression Tool, version 2.1). This Bayesian disease modeling tool generates internally consistent prevalence, incidence, remission, and mortality estimates stratified by sex, region, year, and age group. For areas lacking original epidemiological data, DisMod-MR 2.1 utilizes data from higher hierarchical levels as prior information to estimate parameters for lower levels. For certain causes, the Space-Time Gaussian Process Regression (ST-GPR) model was used as an alternative estimation method. For nonfatal causes, prevalence and incidence were further divided into specific sequelae estimates based on their severity. Sequela categories for nonfatal causes can range from asymptomatic to severe, depending on the disease. For most nonfatal causes, the proportion of cases in each sequela category was calculated using Medical Expenditure Panel Survey (MEPS) analysis. Crude YLD rates were estimated by multiplying the sequela-specific prevalence by the corresponding disability weights.

**4. Comorbidity adjustment**

GBD 2021 adjusted YLDs for comorbidities to account for the coexistence of nonfatal causes in the population, allowing YLDs to be additive within the GBD 2021 cause hierarchy. The coexistence of comorbidities was estimated through simulations involving 20,000 hypothetical individuals for each age, sex, region, and year. Each simulated individual was assigned the probability of having each sequela based on its prevalence. Subsequently, a cumulative disability weight was assigned to each individual by multiplying the disability weights of all assigned sequelae, with appropriate adjustments made to the sequela-specific disability weights.

**5. YLLs and DALYs estimation process**

In GBD 2021, YLLs were calculated by multiplying the estimated number of deaths by the standard life expectancy at the age of death, stratified by age, sex, region, and year. To ensure accurate attribution of causes of death, GBD 2021 employed the principles of the 11th edition of the International Classification of Diseases (ICD-11), assigning each death to the underlying cause that initiated the chain of events leading to death. For deaths recorded with non-specific, unreliable, or intermediate cause codes, reallocation algorithms were applied to reassign these "garbage codes" to the most probable causes of death. These algorithms were derived from published studies, expert consultations, or regression-based adjustments using data from sources reporting multiple causes of death. The cause of death for most diseases and injuries is estimated using the Cause of Death Ensemble model (CODEm). CODEm employs a set of statistical models, systematically testing the predictive validity of different covariate combinations, then combining the results to estimate the number of deaths for specific causes by location, age, sex, and year. For a small number of causes with sparse data or significant changes in reporting practices, GBD 2021 adopted customized modeling strategies, including the use of prevalence, incidence, case fatality data, or data related to sub-causes to infer causes of death. Through this process, GBD 2021 achieved progress in controlling data heterogeneity and reducing uncertainty. To estimate DALYs in GBD 2021, specific cause mortality rates and YLDs were first estimated. DALYs for each year were then calculated by adding YLLs to YLDs. The uncertainty of YLLs was assumed to be independent of the uncertainty of YLDs. By summing the first set of YLLs and YLDs across 500 simulations, and repeating the process for subsequent simulations, the 95% uncertainty interval for DALYs was ultimately calculated. The estimation of DALYs covered every cause, location, age group, sex, and year, providing a comprehensive assessment of the global health burden.

**6. Uncertainty and heterogeneity analysis**

GBD 2021 included extensive uncertainty analysis to ensure the robustness of the estimates. By generating multiple simulations (usually 500) for each population group, accounting for uncertainties in prevalence and DWs, and reporting 95% uncertainty intervals, GBD 2021 provided reliable ranges for disease burden estimates. Additionally, GBD 2021 addressed heterogeneity by adjusting for known biases in the data, ensuring that changes in estimates reflected actual differences in disease burden rather than inconsistencies in data sources. This rigorous approach to uncertainty and heterogeneity analysis is crucial for making health policy decisions based on GBD results.

**7. Data release and application**

GBD 2021's findings were disseminated through various channels, including scientific publications, interactive visualization tools, and the GHDx. These tools enable researchers, policymakers, and the public to explore and compare health data across different regions and time periods. The ultimate goal of GBD 2021 is to provide a comprehensive framework for understanding global and local health trends, thereby supporting evidence-based decision-making and effective allocation of public health resources.

**8. New additions in GBD 2021**

In GBD 2021, estimates for all age groups under 5 years were further subdivided into 0-6 days, 7-27 days, 1-5 months, 6-11 months, 12-23 months, and 2-4 years. Additionally, GBD 2021 for the first time reported five additional Level 3 causes and five additional Level 4 causes. The modeling and reporting of new causes considered data availability, policy concerns, research priorities, and methodological improvements. COVID-19-related causes were included for the first time, encompassing long COVID burden and other pandemic-related outcomes. To better capture the impact of the pandemic, prevalence estimates for severe depression and anxiety were adjusted accordingly.

Through this comprehensive process, GBD 2021 ensured the scientific rigor and global comparability of disease burden estimates, providing a reliable foundation for global health policy and planning by controlling heterogeneity and uncertainty. The rigorous methods and advanced modeling techniques employed by GBD 2021 reflect the high standards and precision necessary to understand and address global health challenges.

## Appendix 2 List of International Classification of Diseases (ICD) codes mapped to CBDs in GBD 2021

| **Cause** | **ICD-10** | **ICD-9** |
| --- | --- | --- |
| Congenital heart anomalies | Q20-Q27, Q27.1-Q28.9 | 745-747.9 |
| Neural tube defects | Q00-Q01.9, Q05-Q05.9, Q07.01, Q07.03 | 740-741.93, 742.0 |
| Digestive congenital anomalies | Q38-Q38.0, Q38.3-Q38.4, Q38.6-Q43, Q43.1-Q45.8, Q79.0-Q79.59 | 750-751, 751.1-751.9, 756.6-756.79 |
| Down syndrome | Q90-Q90.9 | 758.0 |
| Orofacial clefts | Q35-Q37.9 | 749-749.9 |
| Turner syndrome | Q96-Q96.9 | 758.6 |
| Klinefelter syndrome | Q98-Q98.9 | 758.7 |
| Other chromosomal abnormalities | Q74.8, Q75.1, Q75.4, Q75.8, Q79.6, Q87-Q87.89, Q91-Q93.9, Q95, Q95.2-Q95.9, Q97-Q97.9, Q99- Q99.9 | 758, 758.1-758.5, 758.8-758.9, 759.7-759.89 |
| Congenital musculoskeletal and limb anomalies | Q65-Q65.2, Q65.8-Q66.1, Q68, Q68.1-Q68.2, Q68.6  Q74, Q74.1-Q74.3, Q74.9-Q75.0, Q75.5, Q75.9- Q76, Q76.1-Q76.49, Q76.8-Q79, Q79.8-Q79.9 | 754-756.19, 756.4-756.59, 756.8-756.9 |
| Urogenital congenital anomalies | P96.0, Q50-Q52.2, Q52.4, Q52.6-Q52.9, Q54-Q55.2, Q55.22-Q57, Q64-Q64.19 | 752.0-752.9, 753.5 |
| Other congenital birth defects | G71.2, Q02-Q04.9, Q06-Q07.00, Q07.02, Q07.8- Q07.9, Q10-Q15.9, Q17-Q18.9, Q27.0, Q30-Q34.9,  Q38.1-Q38.2, Q38.5, Q43.0, Q45.9, Q52.3, Q52.5,  Q53-Q53.9, Q55.20-Q55.21, Q63.3, Q65.3-Q65.6,  Q66.2-Q67.8, Q68.0, Q68.3-Q68.5, Q74.0, Q75.2-  Q75.3, Q76.0, Q76.5-Q76.7, Q80-Q86.8, Q89- Q89.8, Q95.0-Q95.1 | 237.7, 237.73-237.79, 742, 742.1-744, 744.00-744.9, 748-  748.9, 751.0, 752, 753, 756.2-756.3, 757-757.9, 759-759.6,  759.9 |

## Table S1 DALYs of CBDs in 1990 and 2021 by different characteristics

| **Characteristics** | **1990** | | **2021** | |
| --- | --- | --- | --- | --- |
|  | **All ages number (95% UI)** | **ASDR (95% UI)** | **All ages number (95% UI)** | **ASDR (95% UI)** |
| Overall | 88529007.36(56817570.65 to 113394608.92) | 1420.72(915.54 to 1818.12) | 52325332.69(45186944.83 to 62682127.14) | 802.17(692.41 to 962.51) |
| Sex |  |  |  |  |
| Male | 47974788.13(30819193.9 to 63427358.68) | 1490.32(961.4 to 1967.08) | 27696709.76(23330922.35 to 34278906.41) | 825.17(690.99 to 1025.15) |
| Female | 40554219.23(23166265.05 to 55728578.95) | 1346.1(771.89 to 1846.34) | 24628622.93(20749667.87 to 30074370.48) | 777.67(653.66 to 954.87) |
| Sociodemographic index |  |  |  |  |
| Low SDI | 17694087.25(8151875.12 to 26905335.29) | 1858.89(875.31 to 2816.95) | 19552865.7(15275567.95 to 25660393.27) | 1193.38(943.47 to 1554.37) |
| Low-middle SDI | 25987200.47(15839006.22 to 33171092.55) | 1488.73(917.46 to 1892.38) | 16189429.78(13599286.49 to 19597856.5) | 853.32(716.33 to 1033.35) |
| Middle SDI | 27110439.99(18476819.88 to 34924378.69) | 1366.33(933.34 to 1758.96) | 10752030.84(9298318.88 to 12489247.21) | 617.34(529.81 to 719.52) |
| High-middle SDI | 13034567.59(9890047.58 to 14796401.42) | 1428.28(1080.31 to 1624.03) | 3494551.89(3078944.28 to 3987574.27) | 472.26(412.27 to 534.29) |
| High SDI | 4631957.29(4325954.7 to 4945609.43) | 711.1(665.96 to 757.07) | 2287178.3(2042949.97 to 2561952.63) | 341.83(310.41 to 379.38) |
| GBD regions |  |  |  |  |
| Andean Latin America | 897589.53(521366.65 to 1162506.47) | 1659.31(968.66 to 2146.3) | 487211.5(385516.73 to 606939.44) | 798.51(629.57 to 997.19) |
| Australasia | 93684.52(87459.89 to 100675.1) | 570.7(534.86 to 611.05) | 65049.32(57267.71 to 75474.73) | 296.34(259.81 to 345.45) |
| Caribbean | 625705.61(526813.36 to 710957.58) | 1494.68(1257.66 to 1697.29) | 451864.86(328920.1 to 745851.26) | 1146.93(831.49 to 1884.83) |
| Central Asia | 772638.43(715744.92 to 847252.77) | 845.33(780.74 to 927.26) | 719425.12(611675.07 to 846787.9) | 730.05(621.18 to 858.91) |
| Central Europe | 938180.32(882262.81 to 987970.9) | 1052.75(990.39 to 1107.95) | 229657.16(200974.72 to 265358.73) | 354.66(309.46 to 407.05) |
| Central Latin America | 2535787.67(2306259.17 to 2760327.33) | 1103.04(1004.93 to 1202.94) | 1709386.83(1413010.62 to 2070867.95) | 841.53(689.03 to 1026.81) |
| Central Sub-Saharan Africa | 1878316.71(850854.88 to 3209689.85) | 1688.53(781.25 to 2877.09) | 1798952.61(1286436.05 to 2634021.14) | 885.42(644.56 to 1283.82) |
| East Asia | 17760865.84(12108470.68 to 23523402.77) | 1528.45(1037.67 to 2026.18) | 3341945.12(2796000.42 to 3999703.08) | 430.02(358.52 to 523.58) |
| Eastern Europe | 1725111.01(1632731.78 to 1817684.63) | 1083.5(1032.51 to 1135.15) | 501898.97(445167.78 to 568330.64) | 414.62(376.87 to 459.12) |
| Eastern Sub-Saharan Africa | 6648855.11(2760128.49 to 11952096.48) | 1729.76(747.94 to 3097.91) | 6415184.98(4657852.64 to 9791448.43) | 1022.34(745.81 to 1549.36) |
| High-income Asia Pacific | 744268.11(667430.21 to 832914.36) | 660.15(592.53 to 728.87) | 274357.15(229776.51 to 321130.59) | 302.3(259.02 to 350.6) |
| High-income North America | 1372209.35(1301212.07 to 1451558.69) | 594.36(566.15 to 624.72) | 887562.88(801023.72 to 975617.18) | 360.88(328.89 to 396.28) |
| North Africa and Middle East | 15934750.07(7685402.16 to 19273999.17) | 3127.92(1526.63 to 3778.81) | 6933413.73(5881521.99 to 8156583.05) | 1170.84(990.11 to 1378.46) |
| Oceania | 146866.84(62437.38 to 196082.21) | 1447.14(640.19 to 1915.59) | 266502.95(130257.62 to 368622.03) | 1363.63(687.9 to 1870.63) |
| South Asia | 18587332.06(13208766.34 to 23397339.77) | 1198.69(860.46 to 1509.46) | 10947444.69(8718010.25 to 14763391.46) | 684.15(542.83 to 928.37) |
| Southeast Asia | 6997840.67(4000123.61 to 9041207.17) | 1206.22(693.45 to 1559.3) | 4101312.85(3509382.37 to 4836244.23) | 718.7(614.2 to 851.09) |
| Southern Latin America | 506506.91(481949.68 to 534144.93) | 997.8(949.34 to 1052.34) | 287768.21(248613.92 to 337831.75) | 663.67(563.03 to 788.68) |
| Southern Sub-Saharan Africa | 525336.99(448216.19 to 611828.77) | 714.51(611.17 to 830.23) | 510432.18(372857.2 to 653373.96) | 642.92(467.92 to 824.52) |
| Tropical Latin America | 1552785.88(1331100.38 to 1763080.74) | 954(817.36 to 1082.76) | 1221247.41(1030590.08 to 1441812.32) | 696.59(581.94 to 824.07) |
| Western Europe | 1615373.18(1516945.97 to 1721285.59) | 636.95(605.13 to 670.4) | 856519.27(754118.38 to 966789.79) | 311.32(278.06 to 350.17) |
| Western Sub-Saharan Africa | 6669002.55(2549556.96 to 9282806.23) | 1784.22(721 to 2464.56) | 10318194.9(7428248.67 to 12959094.38) | 1311(964.96 to 1634.63) |
| Countries and territories |  |  |  |  |
| Afghanistan | 990521.93(275190.47 to 1369960.87) | 5202.14(1467.69 to 7188.97) | 1420329.89(820936.16 to 1881908.88) | 2591.86(1505.58 to 3414.02) |
| Albania | 32758.17(25741.37 to 43076.8) | 844.41(664.26 to 1107.18) | 7659.17(5562.45 to 10463.97) | 505.2(356.75 to 701.01) |
| Algeria | 1134699.78(587707.79 to 1442038.84) | 3119.51(1628.75 to 3959.43) | 531428.02(428352.74 to 661172.9) | 1195.31(962.79 to 1488.5) |
| American Samoa | 348.52(284.22 to 422.73) | 465.49(381.27 to 555.87) | 171.87(129.35 to 224.78) | 443.23(329.23 to 591.72) |
| Andorra | 153.54(118.16 to 190) | 471.82(344.01 to 588.11) | 92.55(73.94 to 114.29) | 177.98(142.44 to 217.14) |
| Angola | 383526.69(134339.18 to 728015.37) | 1816.43(657.83 to 3414.09) | 504694.6(349741.21 to 757255.37) | 929.98(652.24 to 1380.38) |
| Antigua and Barbuda | 317.49(275.88 to 366.48) | 530.52(460.58 to 612.49) | 283.19(250.47 to 321.49) | 470.43(413.59 to 533.42) |
| Argentina | 343727.5(326275.79 to 363628.31) | 1027.07(975.11 to 1086.35) | 211854.32(178900.16 to 251636.31) | 717.59(598.32 to 862.54) |
| Armenia | 44764.56(39493.39 to 50930.79) | 1226.16(1081.6 to 1394.69) | 12378.14(10488.79 to 14516.46) | 639.63(544.51 to 758.2) |
| Australia | 75415.03(70200.35 to 81185.02) | 563.08(526.39 to 602.74) | 51798.05(45441.65 to 60572.41) | 283.43(246.7 to 334.41) |
| Austria | 33061.6(30855.16 to 35392.12) | 662.93(622.09 to 708.43) | 17643.21(15279.37 to 20096.78) | 313.61(274.36 to 355.27) |
| Azerbaijan | 102060.06(83332.34 to 124918.88) | 1168.79(955.46 to 1427.01) | 51082.71(40344.91 to 69209.1) | 715.61(554.19 to 989.46) |
| Bahamas | 1793.15(1527.14 to 2068.81) | 675.24(572.93 to 779.37) | 1301.51(1076.51 to 1590.6) | 516.3(417.32 to 642.76) |
| Bahrain | 8940.07(7341 to 10047.41) | 1438.67(1186.38 to 1615.56) | 5290.02(4539.18 to 6145.56) | 503.51(430.74 to 588.5) |
| Bangladesh | 3077405.87(1840333.78 to 4435910.19) | 1636.46(989.15 to 2334.84) | 909696.14(524079.8 to 1509666.99) | 629.58(358.63 to 1054.32) |
| Barbados | 1545.57(1328.62 to 1768.99) | 732.44(626.07 to 839.53) | 1078.12(833.56 to 1403.81) | 681.59(513.28 to 910.05) |
| Belarus | 92114.62(79524.89 to 105888.17) | 1234.93(1059.32 to 1420.27) | 20250.33(16989.36 to 24229.06) | 348.09(293.49 to 423.6) |
| Belgium | 37886.93(35116.06 to 40539.02) | 567.69(529.99 to 603.78) | 22583.88(19645.57 to 26196.49) | 312.44(269.77 to 366.56) |
| Belize | 2563.2(2272.82 to 2902.87) | 887.49(789.16 to 1004.48) | 2120.45(1811.52 to 2518.36) | 543.51(462.14 to 650.22) |
| Benin | 197988.27(73014.07 to 284444.73) | 1967.91(759.4 to 2814.53) | 294790.82(218079.43 to 386583.68) | 1282.79(960.38 to 1665.26) |
| Bermuda | 230.86(182.61 to 307.22) | 491.87(385.61 to 663.98) | 111.86(79.34 to 154.26) | 313.98(211.37 to 460.78) |
| Bhutan | 12705.29(6546.71 to 19160.68) | 1288.09(671.24 to 1921.59) | 4107.22(2391.49 to 6239) | 650.22(371.54 to 999) |
| Bolivia (Plurinational State of) | 293828.24(129608.12 to 401100.36) | 2824.49(1256.89 to 3850.64) | 159744.47(122913.74 to 201655.59) | 1352.8(1040.96 to 1708.72) |
| Bosnia and Herzegovina | 20002.72(17091.52 to 23689.72) | 568.08(485.4 to 680.08) | 5617.64(4601.07 to 6896.67) | 309.96(257.27 to 381.55) |
| Botswana | 13078.98(9609.58 to 16746.06) | 634.41(471.76 to 798.13) | 13989.18(9757.75 to 18786.52) | 592.43(412.25 to 796.7) |
| Brazil | 1503207.39(1285974.81 to 1713558.83) | 959.06(819.88 to 1093.86) | 1175263.31(991878.65 to 1380979.03) | 695.86(582.65 to 820.69) |
| Brunei Darussalam | 2871.71(2477.85 to 3263.34) | 874.78(754.9 to 991.67) | 2530.43(2102.47 to 3049.65) | 776.84(639.18 to 944.6) |
| Bulgaria | 61393.67(57202.7 to 65774.53) | 1153.74(1074.88 to 1232.47) | 16614.3(14257.15 to 19356.71) | 468.99(401.29 to 548.59) |
| Burkina Faso | 431153.33(133997.02 to 626655.3) | 2192.51(707.91 to 3182.13) | 682291.09(382312.99 to 945681.78) | 1646.45(943.61 to 2260.15) |
| Burundi | 204073.6(82386.29 to 342138.61) | 1824.32(775.71 to 3031.27) | 211072.41(146770.97 to 296431.99) | 1000.77(700.07 to 1391.84) |
| Cabo Verde | 6630.92(3998.59 to 8703.65) | 1168.91(727.99 to 1519.33) | 2270.37(1720.29 to 3030.92) | 489.6(362.93 to 662.99) |
| Cambodia | 461234.44(152008.66 to 664710.2) | 2442.54(842.97 to 3483.07) | 214844.29(158433.79 to 284133.83) | 1242.63(914.61 to 1643.97) |
| Cameroon | 321858.89(138727.32 to 441103.48) | 1595.21(720.08 to 2166.38) | 538518.71(382430.66 to 687923.31) | 1153(825.72 to 1456.94) |
| Canada | 130744.61(121886.03 to 140898.99) | 622.15(582.51 to 666.01) | 79776.11(70299.59 to 91298.1) | 328.21(288.5 to 376.5) |
| Central African Republic | 108700.76(45934.29 to 190487.74) | 1996.01(855.87 to 3488.6) | 125808.12(70565.57 to 200208.16) | 1476.51(842.74 to 2337.05) |
| Chad | 197747.73(66711.66 to 302388.24) | 1522.3(547.61 to 2307.7) | 480593.05(240205.25 to 713874.28) | 1336.9(698.53 to 1965.89) |
| Chile | 134932.55(127125.53 to 143157.81) | 929.01(874.87 to 986.99) | 62769.58(54505.35 to 71139.94) | 526.29(457.37 to 601.67) |
| China | 17420113.08(11803475.94 to 23131702.34) | 1554.38(1048.63 to 2065.27) | 3224746.42(2699595.33 to 3856097.69) | 430.61(358.48 to 523.47) |
| Colombia | 393992.74(349062.34 to 435636.82) | 925.11(819.39 to 1023.61) | 232610.84(178642 to 303269.43) | 649.12(491.51 to 863.26) |
| Comoros | 14796.47(6686.85 to 26180.41) | 1697.18(799.18 to 2984.66) | 8524.36(5624.54 to 13022.06) | 1050.45(693.97 to 1603.31) |
| Congo | 51308.49(27470.76 to 85012.8) | 1270.36(690.49 to 2092.27) | 44332.88(32433.31 to 63162.35) | 725.09(531.95 to 1032.02) |
| Cook Islands | 84.28(59.13 to 112.18) | 397.02(279.71 to 527.28) | 28.54(20.62 to 43.74) | 215.65(153.26 to 347.67) |
| Costa Rica | 38245.43(35615.85 to 40589.97) | 989.27(921.28 to 1049.68) | 24501.38(21177.16 to 27947.33) | 771.56(660.02 to 894.67) |
| Côte d'Ivoire | 379336.19(160984.48 to 516135.75) | 1604.17(723.51 to 2166.27) | 504858.83(390085.22 to 648195.96) | 1195.82(930.97 to 1515.62) |
| Croatia | 21961.09(20298.89 to 23699.15) | 719.99(668.15 to 773.42) | 7673.37(6484.72 to 9154.31) | 344.6(291.99 to 409.08) |
| Cuba | 77147.68(73057.12 to 81934.35) | 832.04(787.99 to 882.98) | 26680.18(23132.17 to 30522.93) | 374.42(326.82 to 430.49) |
| Cyprus | 4051.41(2633.88 to 4945.64) | 595.44(379.9 to 730.96) | 1974.72(1627.7 to 2367.59) | 207.13(172.78 to 248.39) |
| Czechia | 48127.98(43922.36 to 52248.8) | 697.68(643.19 to 749.69) | 14665.15(11846.14 to 17870.05) | 204.67(168.86 to 247.4) |
| Democratic People's Republic of Korea | 257866.82(183878.93 to 351214.57) | 1046.8(747.48 to 1424.91) | 84828.74(62372.23 to 119902.51) | 497.76(357.79 to 718.27) |
| Democratic Republic of the Congo | 1306138.07(614843.43 to 2175733.09) | 1671.84(807.14 to 2759.7) | 1098353.46(749797.9 to 1622254.44) | 842.34(580.06 to 1229.45) |
| Denmark | 28925.9(26985.64 to 30980.39) | 856.19(801.88 to 917.93) | 12249.96(10772.74 to 14020.51) | 313.89(274.96 to 359.38) |
| Djibouti | 8351.34(4135.47 to 14637.57) | 1220.16(617.58 to 2127.15) | 11216.96(7332.06 to 17781.84) | 782.65(513.48 to 1236.94) |
| Dominica | 527.52(433.31 to 636.83) | 609.77(503.69 to 733.45) | 396.27(298.23 to 514.43) | 1076.81(791.25 to 1435.84) |
| Dominican Republic | 111112.7(86452.34 to 152544.99) | 1082.62(849.52 to 1477.29) | 57408.28(39737.27 to 97629.72) | 550.47(379.59 to 941.37) |
| Ecuador | 119127.34(104195.77 to 132759.19) | 868.91(762.11 to 964.63) | 125424.46(101008.57 to 156306.96) | 777.57(623.43 to 975.06) |
| Egypt | 2774403.69(1073974.64 to 3643298.46) | 3204.23(1259.95 to 4194.56) | 1094052.21(883069.85 to 1367499.25) | 882.7(714.09 to 1102.21) |
| El Salvador | 124190.75(74829.82 to 158104.26) | 1556.27(949.48 to 1980.59) | 35637.8(25454.09 to 48238.66) | 600.57(424.58 to 818.4) |
| Equatorial Guinea | 12397.39(6201.7 to 21601.04) | 1421.94(734.94 to 2472.73) | 12692.52(8333.35 to 19614.23) | 706.23(463.12 to 1092.74) |
| Eritrea | 89695.04(37158.23 to 164704.58) | 1406.96(608.14 to 2575.75) | 91369.1(58699.76 to 150746.65) | 1017.26(656.4 to 1667.89) |
| Estonia | 10220.84(9378.73 to 11189.26) | 884.23(815.71 to 966.18) | 1836.28(1507.2 to 2228.66) | 207.61(174.73 to 245.85) |
| Eswatini | 10183.67(7229.53 to 12592.05) | 719.42(520.72 to 883.78) | 8822.9(6119.93 to 12066.64) | 647.71(450.21 to 882.61) |
| Ethiopia | 1910045(716558.26 to 3581054.49) | 1859.26(728.05 to 3470.82) | 1439400.05(994034.44 to 2324168.34) | 906.61(636.4 to 1452.96) |
| Fiji | 7745.66(6191.26 to 9727.05) | 855.96(686.07 to 1071.84) | 7735.9(5795.83 to 9961.68) | 860.07(642.43 to 1112.9) |
| Finland | 23194.86(21664.74 to 24848.04) | 650.78(609.01 to 697.51) | 10910.96(9562.42 to 12383.56) | 313.12(277.82 to 355.73) |
| France | 215498.33(200982.15 to 231214.79) | 523.37(492.43 to 558.41) | 122858.51(105600.39 to 141675.73) | 274.65(235.42 to 316.38) |
| Gabon | 16245.31(10196.28 to 26181.23) | 1026.02(663.52 to 1637.49) | 13071.03(9151.89 to 20393.94) | 633.98(443.18 to 989.15) |
| Gambia | 22313.83(9857.39 to 32016.87) | 1197.51(556.42 to 1706.12) | 28237.24(21675.68 to 38061.37) | 831.29(642.63 to 1105.31) |
| Georgia | 25589.17(22515.77 to 29115.94) | 574.3(505.6 to 653.55) | 11496.47(9789.92 to 13600.84) | 464.21(391.11 to 554.63) |
| Germany | 299988.65(279099.35 to 321954.09) | 612.64(575.88 to 650.28) | 170768.61(151421.1 to 192374.27) | 327.44(293.97 to 370.33) |
| Ghana | 339316.31(151401.21 to 468115.6) | 1304.82(609.04 to 1770.81) | 379548.06(284341.75 to 505519.77) | 857.86(646.6 to 1129.84) |
| Greece | 55070.61(50708.38 to 59631) | 921.3(859.12 to 986.71) | 22751.56(19745.71 to 26367.6) | 388.71(337.93 to 449.14) |
| Greenland | 562.96(449.68 to 668.85) | 984.19(788.16 to 1171.46) | 174.69(137.58 to 239.77) | 410.07(318.67 to 579.67) |
| Grenada | 825.83(692.03 to 977.6) | 740.72(624.38 to 869.46) | 429.04(372.16 to 498.16) | 570.54(484.49 to 670.24) |
| Guam | 746.03(628.5 to 882.93) | 442.74(373.25 to 525.04) | 596.92(481.6 to 733.03) | 448.92(358.58 to 552.98) |
| Guatemala | 90752.86(82131.52 to 100973.57) | 599.78(544.15 to 667.39) | 125349.82(100527.69 to 158879.59) | 840.46(669.81 to 1068.87) |
| Guinea | 300592.9(98946.9 to 450571.71) | 2445.13(834.26 to 3647.74) | 321669.54(228045.89 to 427823.04) | 1455.88(1039.32 to 1927.99) |
| Guinea-Bissau | 41689.9(14074.99 to 61568.92) | 2178.18(762.56 to 3210.73) | 36044.18(25905.47 to 48432.82) | 1150.39(829.74 to 1514.33) |
| Guyana | 9836.31(8600.2 to 11395.77) | 842.4(737.77 to 972.73) | 5067.81(3801.19 to 6578.61) | 688.05(513.84 to 894.64) |
| Haiti | 334334.84(249143.02 to 406848.18) | 3043.95(2236.14 to 3762.11) | 311327.8(204898.96 to 544726.84) | 1966.3(1289.7 to 3486.01) |
| Honduras | 121722.6(84640.84 to 155789.62) | 1501.91(1062.21 to 1913.2) | 73864.17(56054.79 to 100623.75) | 686.17(518.65 to 933.15) |
| Hungary | 61784.66(57884.45 to 65953.59) | 930.94(874.61 to 990.79) | 17634.14(14987.67 to 20737.23) | 306.74(259.33 to 359.75) |
| Iceland | 1154.47(1039.82 to 1277.14) | 500.63(450.96 to 553.89) | 744.95(633.5 to 870.75) | 276.01(235.92 to 323.89) |
| India | 13273215.75(8888503.39 to 18222195.88) | 1162.02(788.7 to 1598.71) | 7814429.92(6037520.94 to 10361178.2) | 687.48(526.84 to 921.19) |
| Indonesia | 2455587.64(1574028.37 to 3052216.66) | 1105.61(709.33 to 1373.45) | 1465261.22(1147036.87 to 1907556.46) | 656.68(507.97 to 861.5) |
| Iran (Islamic Republic of) | 2547321.82(1479824.26 to 3136805.98) | 3331.68(1947.03 to 4101) | 289307.89(242898.54 to 344150.3) | 452.71(376.98 to 545.19) |
| Iraq | 716717.48(406764.59 to 922175.06) | 2280.74(1331.34 to 2915.17) | 391187.18(308117.09 to 508661.4) | 943.97(741.59 to 1231.25) |
| Ireland | 21717.67(20220.39 to 23311.31) | 750.38(698.61 to 801.83) | 12877.63(11204.2 to 14697.4) | 381.31(333 to 435.36) |
| Israel | 38025.18(35549.72 to 40816.12) | 749.29(700.6 to 803.73) | 24617.53(21309.26 to 28816.36) | 270.53(234.15 to 316.6) |
| Italy | 226302.26(207993.86 to 246930.55) | 691.51(648.76 to 740.65) | 101753.34(86643.93 to 119027.16) | 305.34(263.14 to 361.22) |
| Jamaica | 21541.73(17910.5 to 25368.31) | 785.99(655.41 to 924.22) | 10905.67(8636.79 to 13887.09) | 589.7(457.84 to 764.01) |
| Japan | 460986.46(419448.52 to 509043.02) | 615.18(571.82 to 667.66) | 200945.49(168255.28 to 237357.54) | 320.58(276.78 to 373.42) |
| Jordan | 121625.49(90311.45 to 145963.22) | 1999.04(1494.54 to 2392.81) | 92325.75(76754.16 to 113345.47) | 837.54(690.45 to 1033) |
| Kazakhstan | 173189.72(152548.53 to 195109.53) | 966.37(851.32 to 1086.86) | 133779.76(113303.54 to 158902.06) | 682.61(578.88 to 809.26) |
| Kenya | 409628.6(262312.12 to 695068.72) | 956.28(630.62 to 1597.74) | 375472.07(257933.77 to 542564.45) | 659.54(453.43 to 951.39) |
| Kiribati | 1826.27(620.78 to 2576.72) | 1531.87(548.81 to 2138.05) | 1427.86(715.31 to 1924.28) | 1021.54(515.15 to 1372.51) |
| Kuwait | 24141.46(20838 to 27273.45) | 1390.32(1199.48 to 1572.17) | 21477.98(18429.72 to 24983.25) | 768.84(661.4 to 912.3) |
| Kyrgyzstan | 53848.14(47505.39 to 59914.84) | 879.19(777.8 to 979.64) | 51526.29(43420.08 to 60062.78) | 683.49(576.3 to 796.23) |
| Lao People's Democratic Republic | 213135.09(57735.12 to 335086.09) | 2822.26(803.25 to 4392.85) | 120014.11(76045.41 to 170862.39) | 1442.29(918.65 to 2049.22) |
| Latvia | 23434.27(22000.95 to 24829.21) | 1216.91(1143.89 to 1285.15) | 3265.85(2788.18 to 3855.16) | 282.3(244.42 to 331.01) |
| Lebanon | 40048.57(20153.52 to 53059.36) | 1018.33(529.99 to 1339.5) | 16943.43(13055.96 to 21624.64) | 391.15(298.86 to 508.32) |
| Lesotho | 19484.09(12106.22 to 26629.9) | 802.85(519.13 to 1082.7) | 16784.62(11401.37 to 23424.82) | 830.99(564.32 to 1161.76) |
| Liberia | 132427.84(36149.97 to 194804.68) | 2737.29(786.66 to 4009.08) | 84789.87(61239.25 to 117095.59) | 1139.91(832.33 to 1567.31) |
| Libya | 149814.14(103547.07 to 187402.36) | 2431.44(1698.54 to 3031.44) | 71230.92(53554.23 to 90455.75) | 1610.35(1186.93 to 2070.65) |
| Lithuania | 31852.63(29578.16 to 34101.63) | 1109.35(1026.99 to 1185.26) | 5413.08(4698.69 to 6302.74) | 340.74(300.74 to 391.54) |
| Luxembourg | 1236.44(1113.68 to 1381.87) | 459.89(414.58 to 512.99) | 975.64(804.75 to 1174.08) | 225.36(188.99 to 267.29) |
| Madagascar | 348503.38(155822.32 to 644235.47) | 1558.87(728.91 to 2845.83) | 405028.68(276577.34 to 603794.15) | 1037.41(722.04 to 1527) |
| Malawi | 469556.87(185368.71 to 820292.7) | 2202.98(904.72 to 3852.6) | 269899.21(185457.87 to 385854.95) | 1012.01(699.48 to 1441.42) |
| Malaysia | 168053.99(119330.99 to 202703.82) | 732.5(525.96 to 879.96) | 117375.88(96677.35 to 138455.11) | 453.24(370.84 to 538.79) |
| Maldives | 5718.36(2130.67 to 8000.57) | 1432.28(555.24 to 1983.44) | 1968.82(1587.99 to 2470.31) | 586.96(467.13 to 751.3) |
| Mali | 529918.68(313522.03 to 700361.7) | 2957.27(1871.63 to 3888.97) | 733586.5(517996.44 to 997013.25) | 1626.33(1131.63 to 2319.9) |
| Malta | 2420.73(2217.28 to 2650.65) | 844.82(772.3 to 928.02) | 1419.61(1207.62 to 1673.67) | 569.99(477.05 to 676.33) |
| Marshall Islands | 529.56(333.6 to 663.77) | 781.63(502.14 to 977.18) | 386.75(282.98 to 510.13) | 677.51(494.49 to 896.87) |
| Mauritania | 44678.47(17420.94 to 64433.53) | 1214.69(497 to 1742.11) | 44846.74(33849.05 to 59334.6) | 731.71(558.14 to 964.04) |
| Mauritius | 9604.82(8429.15 to 10545.78) | 859.29(752.78 to 945.1) | 5727.81(5021.12 to 6407.41) | 780.08(676.87 to 880.16) |
| Mexico | 1418640.22(1284537.55 to 1556308.67) | 1208.78(1096 to 1325.53) | 925267.45(773460.25 to 1117624.37) | 925.34(764.98 to 1120.51) |
| Micronesia (Federated States of) | 1558.77(815.05 to 2070.27) | 1066.05(569.46 to 1408.58) | 554.52(432.81 to 688.76) | 569.84(442.05 to 712.43) |
| Monaco | 76.82(59.56 to 96.14) | 474.2(357.07 to 601.85) | 73.82(63.52 to 86.34) | 339.92(296.47 to 390.7) |
| Mongolia | 50234.95(28067.97 to 68126.48) | 1508.94(853.03 to 2039.83) | 23565.93(17884.95 to 29776.04) | 642.68(488.95 to 809.44) |
| Montenegro | 2627.52(2151.42 to 3204.8) | 511.48(417.02 to 628.83) | 790.08(614.07 to 1042.07) | 177.21(138.45 to 235.54) |
| Morocco | 479740.02(377240.04 to 609973.52) | 1322.94(1040.29 to 1670.67) | 151131.41(103758.72 to 278228.51) | 464.08(316.98 to 856.66) |
| Mozambique | 615864.14(207299.25 to 1169398.78) | 2360.75(820.1 to 4441.37) | 605704.94(408728.92 to 978972.58) | 1203.82(828.78 to 1918.09) |
| Myanmar | 1402897.57(475259.5 to 2088921.91) | 2722.27(928.62 to 4053.85) | 811728.75(525877.61 to 1110212.26) | 1552.47(998.55 to 2126.97) |
| Namibia | 15322.53(10497.02 to 19848.5) | 677.12(478.93 to 867) | 14773.11(10171.19 to 20297.14) | 542.15(373.52 to 743.37) |
| Nauru | 147.91(86.25 to 195.95) | 953.97(565.74 to 1251.37) | 113.81(77.35 to 156.6) | 838.77(572.54 to 1150.48) |
| Nepal | 306617.3(241970.81 to 379818.11) | 909.87(727.32 to 1114.35) | 119266.48(77843.7 to 227761.58) | 381.52(248.84 to 731.47) |
| Netherlands | 72774.06(68291.35 to 77404.21) | 693.09(652.9 to 734.11) | 38802.93(34707.81 to 43521.49) | 340.26(304.8 to 380.41) |
| New Zealand | 18269.49(16861.77 to 19776.24) | 606.54(559.31 to 657.34) | 13251.26(11781.18 to 14991.87) | 358.62(321.42 to 403.89) |
| Nicaragua | 104107(62814.61 to 139554.89) | 1584.68(971.6 to 2115.28) | 43789.84(32052.87 to 57846.72) | 689.01(501.94 to 914.57) |
| Niger | 338555.82(83052.65 to 512898.46) | 1894.36(480.7 to 2862.7) | 515164.6(299300.26 to 700209.62) | 1035.39(615.67 to 1389.7) |
| Nigeria | 2807106.78(978678.12 to 3942117.1) | 1657.68(610.9 to 2319.61) | 5142156.52(3205586.78 to 6634628.48) | 1395.78(902.45 to 1780.87) |
| Niue | 18.67(13.78 to 24.09) | 794.17(587.2 to 1027.48) | 23.29(19.63 to 27.4) | 1949.81(1636.57 to 2301.03) |
| North Macedonia | 22802.86(16571.47 to 28459.13) | 1363.25(984.33 to 1706.69) | 3606.66(2940.01 to 4474.41) | 293(241 to 369.62) |
| Northern Mariana Islands | 193.67(149.87 to 246.34) | 361.67(283.31 to 457.82) | 110.35(90.11 to 133.32) | 302.67(243.41 to 370.83) |
| Norway | 17908.83(16599.37 to 19384.32) | 567.92(529.56 to 611.8) | 10623.15(9245.99 to 12164.53) | 282.3(248.01 to 323.08) |
| Oman | 52721.23(38541.14 to 66998.3) | 1607.76(1183.34 to 2036.54) | 24885.8(20910.59 to 30908.23) | 610.86(510.05 to 766.4) |
| Pakistan | 1917387.85(1481084.46 to 2280585.44) | 1021.96(796.21 to 1210.33) | 2099944.94(1593229.5 to 3016958.05) | 723.86(551.75 to 1037.49) |
| Palau | 140.14(79.38 to 194.85) | 921.05(519.35 to 1283.19) | 66.67(48.44 to 83.37) | 624.36(444.74 to 791.73) |
| Palestine | 70754.41(47643.25 to 86821.13) | 1796.4(1235 to 2191.26) | 38275.86(30163.11 to 49842.9) | 651.2(513.02 to 846.33) |
| Panama | 36340.27(31626.32 to 41454.93) | 1287.55(1121.52 to 1468.55) | 35691.75(29026.67 to 43313.39) | 992.21(805.2 to 1209.94) |
| Papua New Guinea | 112662.97(39845.63 to 156048.85) | 1700.27(632.7 to 2341.39) | 232128.67(102550.68 to 327005.54) | 1497.52(688.77 to 2088.6) |
| Paraguay | 49578.49(37727.69 to 68723.77) | 828.11(634.21 to 1139.39) | 45984.1(30532.88 to 64712) | 713.77(470.2 to 1010.69) |
| Peru | 484633.96(275490.28 to 647000.41) | 1622.3(928.47 to 2159.76) | 202042.56(140798.26 to 264142.26) | 608.66(422.66 to 800.62) |
| Philippines | 1097098.56(728048.34 to 1481609.22) | 1197.51(806.11 to 1600.83) | 836714.79(697454.49 to 1019281.56) | 745.63(619.72 to 910.46) |
| Poland | 311240.91(296597.13 to 325188.68) | 1110.7(1061.77 to 1156.44) | 79044.57(68058.9 to 92071.43) | 378.02(322.17 to 442.86) |
| Portugal | 56333.18(52676.35 to 60190.98) | 894.9(837.22 to 954.21) | 16770.08(14505.72 to 19311.23) | 281.96(246.98 to 325.19) |
| Puerto Rico | 22099.39(20637.44 to 23673.05) | 675.41(630.1 to 723.72) | 6854.81(5857.12 to 7859.48) | 455.18(392.88 to 527.38) |
| Qatar | 6271.98(4583.78 to 7755.01) | 1201.65(886.94 to 1481.01) | 9480.46(7777.77 to 11465.57) | 429.06(353.16 to 524.54) |
| Republic of Korea | 259014.37(200728.73 to 306736.71) | 725.64(560.04 to 868.15) | 61897.22(48949.7 to 77054.56) | 248.7(200.62 to 306.58) |
| Republic of Moldova | 57656.89(50907.59 to 64909.1) | 1454.65(1283.11 to 1640.33) | 11333.59(9279.44 to 13938.57) | 679.47(547.16 to 868.27) |
| Romania | 210398.79(195696.26 to 226257.38) | 1301.46(1207.83 to 1403.03) | 42006.75(36637.65 to 47838.92) | 399.81(351.1 to 453.62) |
| Russian Federation | 1100933.69(1047457.65 to 1163667.85) | 1031.83(987.89 to 1083.15) | 354423.51(311771.1 to 403774.31) | 395.64(353.4 to 440.4) |
| Rwanda | 261014.92(100323.96 to 481987.52) | 1902.82(766.77 to 3479.34) | 162463.98(112536.7 to 238487.3) | 944.18(658.46 to 1381.01) |
| Saint Kitts and Nevis | 365.46(328.19 to 405.99) | 816.22(733.98 to 905.52) | 201.41(162.45 to 250.53) | 585.42(467.69 to 740.53) |
| Saint Lucia | 1180.58(1002.18 to 1370.03) | 705.06(600.03 to 814.84) | 614(490.47 to 761.35) | 605.15(468.45 to 773.18) |
| Saint Vincent and the Grenadines | 1084.86(916.18 to 1279.41) | 873.71(739 to 1029.53) | 439.96(363.4 to 538.18) | 567.26(459.29 to 709.43) |
| Samoa | 1912.78(1241.53 to 2555.66) | 774.21(512.4 to 1022.5) | 1371.11(1003.02 to 1806.75) | 496(367.28 to 648.57) |
| San Marino | 53.18(43.53 to 65.53) | 368.28(296.6 to 456.3) | 32.19(24.87 to 40.68) | 161.38(125.48 to 208.13) |
| Sao Tome and Principe | 3104.97(1412.92 to 4347.21) | 1519.04(716.54 to 2112.16) | 1380.18(960.78 to 2232.03) | 578.57(404.12 to 935.57) |
| Saudi Arabia | 512338.48(316507.71 to 689525.6) | 2167.81(1358.83 to 2899.72) | 125635.67(100333.94 to 158199.08) | 417.01(328.1 to 539.7) |
| Senegal | 241136.98(89787.18 to 348460.56) | 1644.27(647.69 to 2359.21) | 210881.93(162794.76 to 271614.57) | 966.87(748.5 to 1242.85) |
| Serbia | 89857.4(64079.72 to 106826.59) | 1292.36(908.35 to 1542.03) | 15243.66(12520.64 to 18448.15) | 316.03(263.11 to 388.47) |
| Seychelles | 655.13(560.88 to 766.67) | 826.78(708.44 to 967.95) | 625.42(518.04 to 748.71) | 769.54(634.06 to 931.05) |
| Sierra Leone | 229492.13(63391.6 to 327554.83) | 2706.83(787.59 to 3890.09) | 208109.65(139662.36 to 282707.64) | 1547.5(1058.86 to 2085.87) |
| Singapore | 21395.57(19586.67 to 23153.74) | 862.81(788.6 to 931.31) | 8984.01(7455.19 to 10879.18) | 259.72(216.86 to 313.66) |
| Slovakia | 31357(27318.12 to 35141.16) | 764.71(665.78 to 856.06) | 12898.59(10845.71 to 15502.44) | 400.56(338.74 to 477.18) |
| Slovenia | 8864.3(8090.27 to 9718.15) | 704.32(646.42 to 770.36) | 2860.37(2402.15 to 3412.27) | 221.1(191.54 to 259.46) |
| Solomon Islands | 6413.84(3820.42 to 8573.23) | 1075.51(661.73 to 1417.98) | 6789.28(5234.81 to 8785.81) | 740.12(572.14 to 944.93) |
| Somalia | 260919.18(105382.03 to 498088.81) | 1584.68(670.5 to 3011.53) | 460893.24(267278.92 to 794688.4) | 1101.51(659.99 to 1883.03) |
| South Africa | 365360.18(314429.97 to 436399.8) | 758.93(656.15 to 905.59) | 301439.38(225121.25 to 391611.97) | 609.02(450.53 to 795.75) |
| South Sudan | 202219.12(72705.74 to 386949.17) | 1830.14(673 to 3488.04) | 297085.72(149359.62 to 519220.91) | 1769.65(911.05 to 3068.46) |
| Spain | 162031.96(151781.12 to 172202.59) | 702.55(664.32 to 739.66) | 71914.23(61965.97 to 82262.7) | 279.35(245.19 to 317.17) |
| Sri Lanka | 144800.8(102832.73 to 195135.32) | 808.48(574.6 to 1092.47) | 77791.23(62416.4 to 98328.8) | 454.25(361.96 to 579.21) |
| Sudan | 1871344.44(648268.88 to 2542589.55) | 4919.35(1729.79 to 6669.1) | 1080353.14(786006.01 to 1395705.17) | 1964.25(1432.99 to 2532.41) |
| Suriname | 4822.04(3915.49 to 5812.55) | 1104.43(897.68 to 1330.42) | 3521.96(2591.37 to 4583.13) | 771.44(558.55 to 1014.52) |
| Sweden | 38377.88(35307.13 to 41605.71) | 604.57(558.9 to 655.02) | 20810.85(17958.04 to 24265.4) | 284.16(247.3 to 328.11) |
| Switzerland | 38373.35(35972.76 to 40964.43) | 867(814.71 to 918.74) | 20558.67(17998.86 to 23391.76) | 380.5(335.58 to 436.03) |
| Syrian Arab Republic | 418919.82(246820.94 to 543241.8) | 1972.72(1177.56 to 2545.08) | 75451.3(61790.08 to 92590.4) | 691.54(560.64 to 856.88) |
| Taiwan (Province of China) | 82885.95(77878.62 to 88124.25) | 479.34(452.33 to 509.79) | 32369.97(28300.41 to 37084.5) | 274.3(241.99 to 306.07) |
| Tajikistan | 66298.22(53512.48 to 93866.25) | 718.89(582.39 to 995.74) | 87616.48(60376.39 to 147528.63) | 666.38(463.24 to 1111.72) |
| Thailand | 467890.29(373356.09 to 571100.1) | 897.08(709.14 to 1098.24) | 156625.62(129250.87 to 186370.13) | 433.41(353.68 to 513.55) |
| Timor-Leste | 29602.27(9801.45 to 46098.79) | 1964.37(680.32 to 3023.87) | 20145.19(14791.7 to 27590.23) | 1072.13(800.35 to 1456.28) |
| Togo | 103732.51(45374.24 to 143456.56) | 1513.25(695.02 to 2075.35) | 108336.62(82134.32 to 141900.06) | 976.54(741.37 to 1274.68) |
| Tokelau | 15.12(9.9 to 19.44) | 829.57(547.06 to 1060.55) | 22.9(18.65 to 30.88) | 2340.52(1896.31 to 3197.09) |
| Tonga | 751.45(551.2 to 980.45) | 512.33(385.71 to 661.73) | 484.93(371.89 to 656.4) | 360.94(278.34 to 485.8) |
| Trinidad and Tobago | 12406.37(10861.73 to 14148.47) | 1031.6(900.34 to 1176.21) | 7645.22(6057.79 to 9562.21) | 892.82(692.22 to 1140.35) |
| Tunisia | 270818.83(144606.14 to 342446.28) | 2590.04(1389.95 to 3269.66) | 66290.56(54575.69 to 81365.65) | 745.69(607.83 to 913.74) |
| Turkey | 2459026.75(1145901.15 to 3167576.12) | 3513.86(1642.36 to 4524.97) | 445744.27(372231.58 to 528052.47) | 805.41(662.44 to 963.03) |
| Turkmenistan | 51504.58(45138.55 to 58417.59) | 902.27(796.25 to 1019.89) | 61207.33(49971.69 to 75294.25) | 1150.28(939.15 to 1414.81) |
| Tuvalu | 247.65(88.81 to 356.03) | 1621.23(614.91 to 2307.76) | 79.35(59.23 to 105.32) | 618.35(461.24 to 820.66) |
| Uganda | 529187.07(220885.28 to 969355.47) | 1340.18(583.88 to 2434.04) | 705574.13(490219.96 to 1026787.98) | 980.95(693.28 to 1419.6) |
| Ukraine | 408898.08(362861.12 to 457434.19) | 1157.96(1023.21 to 1307.22) | 105376.32(91477.35 to 120175.04) | 521.96(453.13 to 590.38) |
| United Arab Emirates | 31318.85(22800.87 to 39795.6) | 1390.9(1017.64 to 1764.17) | 26781.49(22103.11 to 31414.43) | 526.68(433.73 to 618.24) |
| United Kingdom | 239427.71(224991.69 to 255288.16) | 566.61(535.03 to 599.38) | 151956.58(134616.85 to 172095.11) | 338.06(299.9 to 379.53) |
| United Republic of Tanzania | 1025549.03(417022.5 to 1789389.16) | 1964.36(826.67 to 3412.38) | 1069806.5(740367.31 to 1591892.86) | 1224.43(858.39 to 1808.66) |
| United States of America | 1240870.32(1175399.35 to 1311999.03) | 591.45(562.49 to 621.96) | 807598.16(730781.3 to 887323.2) | 364.08(332.4 to 399.81) |
| United States Virgin Islands | 782.87(653.63 to 918.1) | 705.63(589.11 to 827.68) | 185.69(146.14 to 242.66) | 371.75(286.28 to 508.55) |
| Uruguay | 27822.79(25837.91 to 29854.05) | 1012.86(941 to 1086.86) | 13128.43(11243.31 to 15582.58) | 630.93(534.08 to 760.23) |
| Uzbekistan | 205149.04(175990.71 to 234832.94) | 639.46(550.66 to 732.37) | 286772.01(237345.71 to 355432.6) | 758.84(628.97 to 937.61) |
| Vanuatu | 2154.2(1110.04 to 2948.37) | 817.59(447.03 to 1101.83) | 2444.99(1764.02 to 3180.57) | 611.44(447.38 to 787.73) |
| Venezuela (Bolivarian Republic of) | 207795.79(194327.13 to 223013.09) | 821.47(768.93 to 881.14) | 212673.79(157926.51 to 273349.47) | 943.95(696.61 to 1218.58) |
| Viet Nam | 531438.68(274828.21 to 689824.6) | 592.34(316.11 to 766.53) | 266769.26(201267.35 to 346629.12) | 323.47(239.88 to 424.89) |
| Yemen | 1244543.84(436761.58 to 1707663.71) | 4246.93(1533.27 to 5845.36) | 949343.68(660530.24 to 1232865.68) | 2064.64(1443.71 to 2674.43) |
| Zambia | 294699.62(116650.44 to 548442.87) | 1814.25(747.62 to 3356.42) | 296088.66(202873.62 to 454799.3) | 1051.3(725.2 to 1600.69) |
| Zimbabwe | 101907.53(72252.18 to 128887.89) | 591.27(423.1 to 744.63) | 154623(103088.52 to 209823.86) | 724.97(486.23 to 978.38) |

## Table S2 Ranking of 11 types of DALYs of CBDs globally, in various SDI regions, and GBD regions

| **Location** | **Cause** | **2021** | | **1990** | |
| --- | --- | --- | --- | --- | --- |
|  |  | **ASDR (95% UI)** | **Rank** | **ASDR (95% UI)** | **Rank** |
| Global | Congenital heart anomalies | 345.24(288.34 to 422.16) | 1 | 750.3(440.94 to 960.11) | 1 |
| Global | Other congenital birth defects | 160(126.05 to 214.09) | 2 | 218.78(146.46 to 389.86) | 2 |
| Global | Neural tube defects | 83.18(67.94 to 102.1) | 3 | 161.8(111.67 to 210.07) | 3 |
| Global | Digestive congenital anomalies | 70.44(53.14 to 87.6) | 4 | 108.3(60.88 to 166.69) | 4 |
| Global | Congenital musculoskeletal and limb anomalies | 54.32(40.69 to 71.09) | 5 | 65.87(46.75 to 89.22) | 5 |
| Global | Down syndrome | 35.2(26 to 57.17) | 6 | 45.49(26.17 to 102.27) | 6 |
| Global | Other chromosomal abnormalities | 34.23(28.3 to 48.86) | 7 | 32.55(23.18 to 59.3) | 7 |
| Global | Urogenital congenital anomalies | 13.56(10.08 to 19.57) | 8 | 16.63(12.38 to 24.89) | 9 |
| Global | Orofacial clefts | 5.78(3.48 to 9.82) | 9 | 20.78(9.71 to 35.78) | 8 |
| Global | Turner syndrome | 0.12(0.06 to 0.21) | 10 | 0.12(0.06 to 0.22) | 10 |
| Global | Klinefelter syndrome | 0.09(0.04 to 0.17) | 11 | 0.08(0.04 to 0.17) | 11 |
| Low SDI | Congenital heart anomalies | 488.54(349.65 to 658.02) | 1 | 860.62(324.42 to 1252.88) | 1 |
| Low SDI | Other congenital birth defects | 239.13(173.58 to 366.67) | 2 | 297.88(163.92 to 657.83) | 3 |
| Low SDI | Neural tube defects | 175.35(131.63 to 224.19) | 3 | 331.38(185.79 to 477.62) | 2 |
| Low SDI | Digestive congenital anomalies | 103.58(69.24 to 141.71) | 4 | 149.39(50.91 to 259.22) | 4 |
| Low SDI | Congenital musculoskeletal and limb anomalies | 59.91(45.66 to 79.61) | 5 | 67.79(45.35 to 96.99) | 6 |
| Low SDI | Down syndrome | 57.85(34.1 to 120.9) | 6 | 71.55(20.85 to 231.96) | 5 |
| Low SDI | Other chromosomal abnormalities | 45.91(33.59 to 85.59) | 7 | 48.04(24.34 to 121.79) | 7 |
| Low SDI | Urogenital congenital anomalies | 14.26(9.52 to 24.57) | 8 | 16.88(10.85 to 33.81) | 8 |
| Low SDI | Orofacial clefts | 8.65(3.88 to 21.66) | 9 | 15.15(4.67 to 46.74) | 9 |
| Low SDI | Turner syndrome | 0.12(0.05 to 0.21) | 10 | 0.12(0.06 to 0.22) | 10 |
| Low SDI | Klinefelter syndrome | 0.09(0.04 to 0.17) | 11 | 0.09(0.04 to 0.18) | 11 |
| Low-middle SDI | Congenital heart anomalies | 381.65(302.67 to 476.97) | 1 | 796.79(430.97 to 1074.7) | 1 |
| Low-middle SDI | Other congenital birth defects | 177.24(133.96 to 238.23) | 2 | 228.61(137.57 to 445.1) | 2 |
| Low-middle SDI | Neural tube defects | 78.79(61.81 to 103.33) | 3 | 173.69(118.08 to 218.61) | 3 |
| Low-middle SDI | Digestive congenital anomalies | 76.78(54.74 to 100.1) | 4 | 110.01(56.35 to 186.25) | 4 |
| Low-middle SDI | Congenital musculoskeletal and limb anomalies | 54.17(40.63 to 72.62) | 5 | 67.39(44.17 to 96.7) | 5 |
| Low-middle SDI | Down syndrome | 30.86(22.28 to 46.27) | 6 | 38.82(18.85 to 101.07) | 6 |
| Low-middle SDI | Other chromosomal abnormalities | 29.56(23.5 to 40.84) | 7 | 33.08(20.42 to 72.07) | 7 |
| Low-middle SDI | Urogenital congenital anomalies | 17.03(11.07 to 27.49) | 8 | 20.6(12.88 to 31.9) | 8 |
| Low-middle SDI | Orofacial clefts | 7.05(4.61 to 10.57) | 9 | 19.54(8.7 to 42.21) | 9 |
| Low-middle SDI | Turner syndrome | 0.11(0.05 to 0.2) | 10 | 0.11(0.05 to 0.2) | 10 |
| Low-middle SDI | Klinefelter syndrome | 0.08(0.04 to 0.17) | 11 | 0.08(0.04 to 0.17) | 11 |
| Middle SDI | Congenital heart anomalies | 286.65(241.63 to 342.81) | 1 | 768.69(487.96 to 1009.29) | 1 |
| Middle SDI | Other congenital birth defects | 124.49(101.76 to 148.77) | 2 | 203.96(140.89 to 335.86) | 2 |
| Middle SDI | Digestive congenital anomalies | 55.53(41.77 to 71.29) | 3 | 101.75(62.68 to 163.81) | 4 |
| Middle SDI | Congenital musculoskeletal and limb anomalies | 47.91(35.62 to 63.17) | 4 | 60.79(44.35 to 80.04) | 5 |
| Middle SDI | Neural tube defects | 37.3(30.57 to 46.22) | 5 | 126.16(97.12 to 158.66) | 3 |
| Middle SDI | Down syndrome | 25.03(21.16 to 30.43) | 6 | 43.52(29.15 to 76.69) | 6 |
| Middle SDI | Other chromosomal abnormalities | 24.26(20.38 to 30.6) | 7 | 21.58(16.15 to 34.35) | 8 |
| Middle SDI | Urogenital congenital anomalies | 11.63(8.61 to 14.65) | 8 | 13.33(10.24 to 18.42) | 9 |
| Middle SDI | Orofacial clefts | 4.37(3.11 to 6.1) | 9 | 26.39(13.43 to 42.85) | 7 |
| Middle SDI | Turner syndrome | 0.1(0.04 to 0.17) | 10 | 0.1(0.04 to 0.17) | 10 |
| Middle SDI | Klinefelter syndrome | 0.08(0.04 to 0.15) | 11 | 0.07(0.03 to 0.14) | 11 |
| High-middle SDI | Congenital heart anomalies | 204.05(171.77 to 239.89) | 1 | 783.96(558.45 to 962.93) | 1 |
| High-middle SDI | Other congenital birth defects | 92.33(78.03 to 107.52) | 2 | 214.61(147.31 to 301.28) | 2 |
| High-middle SDI | Congenital musculoskeletal and limb anomalies | 53.14(37.86 to 73.53) | 3 | 69.19(48.73 to 90.7) | 5 |
| High-middle SDI | Digestive congenital anomalies | 39.58(29.49 to 50.79) | 4 | 117.35(74.59 to 170.65) | 4 |
| High-middle SDI | Other chromosomal abnormalities | 25.2(21.66 to 28.68) | 5 | 25.55(21.55 to 31.66) | 8 |
| High-middle SDI | Down syndrome | 23.92(19.56 to 28.38) | 6 | 51.97(37.94 to 75.7) | 6 |
| High-middle SDI | Neural tube defects | 21.7(17.6 to 25.41) | 7 | 118.35(92.43 to 141.64) | 3 |
| High-middle SDI | Urogenital congenital anomalies | 8.99(6.65 to 11.83) | 8 | 15.95(11.23 to 22.94) | 9 |
| High-middle SDI | Orofacial clefts | 3.17(2.19 to 4.46) | 9 | 31.14(13.55 to 59.28) | 7 |
| High-middle SDI | Turner syndrome | 0.11(0.05 to 0.19) | 10 | 0.12(0.05 to 0.2) | 10 |
| High-middle SDI | Klinefelter syndrome | 0.08(0.04 to 0.17) | 11 | 0.08(0.04 to 0.16) | 11 |
| High SDI | Congenital heart anomalies | 101.7(87.06 to 118.34) | 1 | 326.39(283.76 to 357.66) | 1 |
| High SDI | Other congenital birth defects | 70.42(59.76 to 91.82) | 2 | 152.86(129.6 to 174.33) | 2 |
| High SDI | Other chromosomal abnormalities | 50.97(43.81 to 57.39) | 3 | 47.89(43.7 to 53.88) | 4 |
| High SDI | Congenital musculoskeletal and limb anomalies | 45.71(32.93 to 62.17) | 4 | 56.37(41.47 to 74.61) | 3 |
| High SDI | Down syndrome | 23.86(21.42 to 26.52) | 5 | 22.75(19.21 to 28.27) | 7 |
| High SDI | Digestive congenital anomalies | 20.26(16.29 to 24.18) | 6 | 45.17(39.17 to 53.8) | 5 |
| High SDI | Neural tube defects | 15.5(12.97 to 17.62) | 7 | 37.79(35.29 to 42.11) | 6 |
| High SDI | Urogenital congenital anomalies | 10.88(7.73 to 13.95) | 8 | 17.35(13.64 to 23.33) | 8 |
| High SDI | Orofacial clefts | 2.23(1.41 to 3.36) | 9 | 4.25(2.55 to 6.32) | 9 |
| High SDI | Turner syndrome | 0.19(0.09 to 0.33) | 10 | 0.2(0.09 to 0.33) | 10 |
| High SDI | Klinefelter syndrome | 0.11(0.05 to 0.22) | 11 | 0.1(0.05 to 0.21) | 11 |
| Andean Latin America | Congenital heart anomalies | 360.59(278.13 to 454.8) | 1 | 871.67(478.13 to 1128.92) | 1 |
| Andean Latin America | Other congenital birth defects | 166.38(122.84 to 211.19) | 2 | 278.4(143.48 to 503.44) | 2 |
| Andean Latin America | Digestive congenital anomalies | 92.32(65.51 to 122.41) | 3 | 175.65(92.22 to 281.96) | 3 |
| Andean Latin America | Congenital musculoskeletal and limb anomalies | 49.96(37.31 to 67.82) | 4 | 53.19(36.96 to 71.8) | 6 |
| Andean Latin America | Neural tube defects | 43.67(33.06 to 57.75) | 5 | 128.26(83.28 to 171.55) | 4 |
| Andean Latin America | Down syndrome | 38.31(29.29 to 51.35) | 6 | 85.74(43.78 to 168.59) | 5 |
| Andean Latin America | Other chromosomal abnormalities | 28.28(22.34 to 36.01) | 7 | 19.37(12.54 to 32.52) | 8 |
| Andean Latin America | Urogenital congenital anomalies | 13.78(8.3 to 23.53) | 8 | 14.57(8.6 to 25.6) | 9 |
| Andean Latin America | Orofacial clefts | 5.05(3.69 to 7.11) | 9 | 32.29(17.12 to 52.04) | 7 |
| Andean Latin America | Turner syndrome | 0.09(0.04 to 0.16) | 10 | 0.1(0.05 to 0.17) | 10 |
| Andean Latin America | Klinefelter syndrome | 0.07(0.04 to 0.15) | 11 | 0.08(0.04 to 0.15) | 11 |
| Australasia | Congenital heart anomalies | 75.26(60.82 to 90.15) | 1 | 180.95(165.96 to 199.09) | 1 |
| Australasia | Other congenital birth defects | 57.6(45.84 to 79.78) | 2 | 126.59(96.28 to 140.8) | 2 |
| Australasia | Congenital musculoskeletal and limb anomalies | 50.52(36.05 to 69.2) | 3 | 62.65(48 to 82.07) | 3 |
| Australasia | Other chromosomal abnormalities | 45.01(35.83 to 53.99) | 4 | 60.05(53.5 to 66.62) | 4 |
| Australasia | Down syndrome | 27.03(22.82 to 31.24) | 5 | 22.56(19.24 to 26.36) | 7 |
| Australasia | Neural tube defects | 16.04(12.33 to 20.22) | 6 | 52.29(46.64 to 58.42) | 5 |
| Australasia | Digestive congenital anomalies | 14.24(11.25 to 21.14) | 7 | 47.1(41.62 to 57.62) | 6 |
| Australasia | Urogenital congenital anomalies | 8.49(5.94 to 15.64) | 8 | 16.12(10.58 to 31.66) | 8 |
| Australasia | Orofacial clefts | 1.93(1.24 to 2.92) | 9 | 2.19(1.44 to 3.25) | 9 |
| Australasia | Turner syndrome | 0.13(0.06 to 0.23) | 10 | 0.13(0.06 to 0.23) | 10 |
| Australasia | Klinefelter syndrome | 0.07(0.03 to 0.14) | 11 | 0.07(0.03 to 0.14) | 11 |
| Caribbean | Congenital heart anomalies | 594.06(374.75 to 982.65) | 1 | 860.63(654.68 to 1067.15) | 1 |
| Caribbean | Other congenital birth defects | 174.03(100.04 to 362.12) | 2 | 157.99(97.23 to 262.55) | 3 |
| Caribbean | Digestive congenital anomalies | 116.09(70.06 to 184.27) | 3 | 168.55(97.53 to 275.7) | 2 |
| Caribbean | Neural tube defects | 79.62(48.19 to 136.32) | 4 | 127.64(89.03 to 186.11) | 4 |
| Caribbean | Congenital musculoskeletal and limb anomalies | 65.72(44.78 to 91.98) | 5 | 64.84(44.46 to 90.09) | 5 |
| Caribbean | Down syndrome | 58.54(32.03 to 108.79) | 6 | 61.12(31.88 to 142.72) | 6 |
| Caribbean | Other chromosomal abnormalities | 40.39(25.2 to 75.27) | 7 | 31.46(22.33 to 61.1) | 7 |
| Caribbean | Urogenital congenital anomalies | 13.56(8.12 to 27.83) | 8 | 16.05(10.53 to 25.19) | 8 |
| Caribbean | Orofacial clefts | 4.75(2.74 to 7.97) | 9 | 6.24(2.77 to 12.85) | 9 |
| Caribbean | Turner syndrome | 0.1(0.05 to 0.18) | 10 | 0.1(0.05 to 0.18) | 10 |
| Caribbean | Klinefelter syndrome | 0.06(0.03 to 0.12) | 11 | 0.06(0.03 to 0.13) | 11 |
| Central Asia | Congenital heart anomalies | 423.52(336.81 to 519.76) | 1 | 417.57(363.7 to 471.6) | 1 |
| Central Asia | Other congenital birth defects | 122.49(97.98 to 160.45) | 2 | 144.84(111.34 to 206.95) | 2 |
| Central Asia | Digestive congenital anomalies | 57.85(46.1 to 73.44) | 3 | 72.63(57.18 to 91.48) | 4 |
| Central Asia | Congenital musculoskeletal and limb anomalies | 56.71(39.81 to 78.09) | 4 | 60.88(43.66 to 81.34) | 5 |
| Central Asia | Neural tube defects | 29.35(22.66 to 39.77) | 5 | 98.13(80.42 to 114.51) | 3 |
| Central Asia | Down syndrome | 15.72(11.71 to 22.81) | 6 | 23(16.14 to 37.66) | 6 |
| Central Asia | Other chromosomal abnormalities | 10.79(8.63 to 13.15) | 7 | 11.69(9.23 to 14.2) | 7 |
| Central Asia | Urogenital congenital anomalies | 7.56(5.6 to 10.52) | 8 | 8.13(5.92 to 10.85) | 9 |
| Central Asia | Orofacial clefts | 5.79(3.9 to 8.64) | 9 | 8.22(5.77 to 11.78) | 8 |
| Central Asia | Turner syndrome | 0.15(0.07 to 0.26) | 10 | 0.15(0.07 to 0.26) | 10 |
| Central Asia | Klinefelter syndrome | 0.11(0.05 to 0.22) | 11 | 0.1(0.05 to 0.2) | 11 |
| Central Europe | Congenital heart anomalies | 143.86(120.48 to 167.77) | 1 | 535.78(461.97 to 595.51) | 1 |
| Central Europe | Other congenital birth defects | 69.75(56.53 to 94.18) | 2 | 196.96(142.55 to 244.85) | 2 |
| Central Europe | Congenital musculoskeletal and limb anomalies | 41.35(28.76 to 56.2) | 3 | 54.66(41.63 to 69.06) | 5 |
| Central Europe | Other chromosomal abnormalities | 36.08(29.45 to 42.87) | 4 | 41.79(37.2 to 49.57) | 6 |
| Central Europe | Digestive congenital anomalies | 26.49(20.89 to 32.86) | 5 | 98.06(73.74 to 126.35) | 3 |
| Central Europe | Down syndrome | 13.42(11.12 to 15.94) | 6 | 16.18(13.51 to 20.75) | 8 |
| Central Europe | Neural tube defects | 12.37(10.01 to 14.96) | 7 | 88.17(77.11 to 106.46) | 4 |
| Central Europe | Urogenital congenital anomalies | 9.27(5.7 to 12.77) | 8 | 16.73(10.39 to 23.91) | 7 |
| Central Europe | Orofacial clefts | 1.85(1.16 to 2.76) | 9 | 4.23(3.04 to 5.78) | 9 |
| Central Europe | Turner syndrome | 0.13(0.06 to 0.22) | 10 | 0.13(0.06 to 0.22) | 10 |
| Central Europe | Klinefelter syndrome | 0.08(0.04 to 0.18) | 11 | 0.08(0.04 to 0.16) | 11 |
| Central Latin America | Congenital heart anomalies | 390.66(308.43 to 492.21) | 1 | 476.47(421.91 to 537.81) | 1 |
| Central Latin America | Other congenital birth defects | 148.79(117.94 to 204.72) | 2 | 205.2(174.63 to 262.11) | 2 |
| Central Latin America | Digestive congenital anomalies | 93.06(69.56 to 120.37) | 3 | 101.79(87.82 to 125.78) | 4 |
| Central Latin America | Congenital musculoskeletal and limb anomalies | 74.99(51.55 to 106.56) | 4 | 87.6(62.67 to 123.54) | 5 |
| Central Latin America | Neural tube defects | 38.71(30.64 to 48.3) | 5 | 144.14(115.59 to 162.04) | 3 |
| Central Latin America | Down syndrome | 36.91(29.03 to 46.58) | 6 | 35.51(30.85 to 39.91) | 6 |
| Central Latin America | Other chromosomal abnormalities | 29.27(23.6 to 36.38) | 7 | 16.77(14.44 to 19.33) | 8 |
| Central Latin America | Urogenital congenital anomalies | 24.61(17.78 to 31.68) | 8 | 21.66(16.74 to 34.78) | 7 |
| Central Latin America | Orofacial clefts | 4.38(3.19 to 5.93) | 9 | 13.74(8.98 to 17.13) | 9 |
| Central Latin America | Turner syndrome | 0.09(0.04 to 0.17) | 10 | 0.1(0.05 to 0.17) | 10 |
| Central Latin America | Klinefelter syndrome | 0.06(0.03 to 0.12) | 11 | 0.06(0.03 to 0.13) | 11 |
| Central Sub-Saharan Africa | Congenital heart anomalies | 288.04(186.1 to 458.16) | 1 | 628.73(203.49 to 1126.97) | 1 |
| Central Sub-Saharan Africa | Other congenital birth defects | 189.71(130.83 to 307.39) | 2 | 302.1(159.6 to 628.25) | 3 |
| Central Sub-Saharan Africa | Neural tube defects | 172.78(106.72 to 248.54) | 3 | 403.34(213.8 to 577.52) | 2 |
| Central Sub-Saharan Africa | Digestive congenital anomalies | 71.28(39.19 to 122.63) | 4 | 142.84(36.58 to 275.3) | 4 |
| Central Sub-Saharan Africa | Down syndrome | 52.87(27.52 to 114.52) | 5 | 75.46(21.66 to 240.73) | 5 |
| Central Sub-Saharan Africa | Congenital musculoskeletal and limb anomalies | 52.1(37.25 to 74.65) | 6 | 60.96(42.9 to 86.62) | 6 |
| Central Sub-Saharan Africa | Other chromosomal abnormalities | 42.07(27.44 to 80.27) | 7 | 52.28(26.59 to 144.59) | 7 |
| Central Sub-Saharan Africa | Urogenital congenital anomalies | 11.57(6.24 to 23.19) | 8 | 14.06(7.87 to 32.35) | 8 |
| Central Sub-Saharan Africa | Orofacial clefts | 4.8(2.22 to 12.11) | 9 | 8.55(2.81 to 29.62) | 9 |
| Central Sub-Saharan Africa | Turner syndrome | 0.12(0.05 to 0.22) | 10 | 0.13(0.06 to 0.22) | 10 |
| Central Sub-Saharan Africa | Klinefelter syndrome | 0.09(0.04 to 0.18) | 11 | 0.09(0.04 to 0.18) | 11 |
| East Asia | Congenital heart anomalies | 241.17(191.48 to 305.04) | 1 | 962.04(614.3 to 1322.94) | 1 |
| East Asia | Other congenital birth defects | 52.89(39.04 to 67.8) | 2 | 145.87(90.29 to 254.78) | 2 |
| East Asia | Digestive congenital anomalies | 40.15(25.59 to 59.03) | 3 | 114.24(61.8 to 206.16) | 4 |
| East Asia | Congenital musculoskeletal and limb anomalies | 40.1(27.18 to 57.79) | 4 | 54.66(37.91 to 74.61) | 6 |
| East Asia | Down syndrome | 23.02(13.17 to 31.61) | 5 | 57.11(37.92 to 89.69) | 5 |
| East Asia | Neural tube defects | 16.81(11.59 to 22.32) | 6 | 131.35(95.15 to 165.69) | 3 |
| East Asia | Other chromosomal abnormalities | 9.36(7.44 to 11.71) | 7 | 9.83(7.66 to 12.87) | 8 |
| East Asia | Orofacial clefts | 3.61(2.44 to 5.17) | 8 | 49.35(18.2 to 93.33) | 7 |
| East Asia | Urogenital congenital anomalies | 2.77(1.65 to 4.35) | 9 | 3.87(2.16 to 7.15) | 9 |
| East Asia | Turner syndrome | 0.08(0.04 to 0.14) | 10 | 0.08(0.04 to 0.14) | 10 |
| East Asia | Klinefelter syndrome | 0.07(0.03 to 0.14) | 11 | 0.06(0.03 to 0.13) | 11 |
| Eastern Europe | Congenital heart anomalies | 150.6(126.77 to 179.24) | 1 | 463.92(410.99 to 551.64) | 1 |
| Eastern Europe | Other congenital birth defects | 110.31(83.79 to 141.42) | 2 | 280.09(179.01 to 323.77) | 2 |
| Eastern Europe | Congenital musculoskeletal and limb anomalies | 56.35(38.61 to 78.39) | 3 | 61.29(44.03 to 81.53) | 5 |
| Eastern Europe | Digestive congenital anomalies | 31.88(24.29 to 41.44) | 4 | 110.05(88.02 to 129.44) | 3 |
| Eastern Europe | Other chromosomal abnormalities | 26.64(22.76 to 30.82) | 5 | 36.97(32.48 to 43.22) | 6 |
| Eastern Europe | Neural tube defects | 15.18(12.65 to 18.2) | 6 | 77.66(68.82 to 90.62) | 4 |
| Eastern Europe | Down syndrome | 13.36(11.17 to 16.11) | 7 | 33.54(29.93 to 39.73) | 7 |
| Eastern Europe | Urogenital congenital anomalies | 8.08(5.55 to 11.55) | 8 | 15.85(12.06 to 20.29) | 8 |
| Eastern Europe | Orofacial clefts | 1.93(1.26 to 2.87) | 9 | 3.83(2.65 to 5.26) | 9 |
| Eastern Europe | Turner syndrome | 0.17(0.08 to 0.3) | 10 | 0.18(0.08 to 0.3) | 10 |
| Eastern Europe | Klinefelter syndrome | 0.13(0.07 to 0.27) | 11 | 0.13(0.06 to 0.26) | 11 |
| Eastern Sub-Saharan Africa | Congenital heart anomalies | 334.69(214.9 to 585.93) | 1 | 668.98(200.64 to 1291.08) | 1 |
| Eastern Sub-Saharan Africa | Other congenital birth defects | 227.32(158.37 to 384.62) | 2 | 307.92(151.11 to 755.12) | 3 |
| Eastern Sub-Saharan Africa | Neural tube defects | 192.6(126.36 to 272.07) | 3 | 382.12(222.41 to 580.12) | 2 |
| Eastern Sub-Saharan Africa | Digestive congenital anomalies | 92.69(52.8 to 148.72) | 4 | 152.5(37.46 to 312.24) | 4 |
| Eastern Sub-Saharan Africa | Congenital musculoskeletal and limb anomalies | 54.83(39.35 to 77.32) | 5 | 61.16(42.54 to 90.89) | 6 |
| Eastern Sub-Saharan Africa | Down syndrome | 53.79(30.29 to 109.87) | 6 | 80.8(18.32 to 275.11) | 5 |
| Eastern Sub-Saharan Africa | Other chromosomal abnormalities | 49.02(33.41 to 91.08) | 7 | 49.97(23.92 to 134.96) | 7 |
| Eastern Sub-Saharan Africa | Urogenital congenital anomalies | 10.62(6.08 to 19.79) | 8 | 12.92(7.45 to 32.75) | 9 |
| Eastern Sub-Saharan Africa | Orofacial clefts | 6.58(2.87 to 18.4) | 9 | 13.18(3.79 to 50.41) | 8 |
| Eastern Sub-Saharan Africa | Turner syndrome | 0.13(0.06 to 0.22) | 10 | 0.13(0.06 to 0.23) | 10 |
| Eastern Sub-Saharan Africa | Klinefelter syndrome | 0.09(0.04 to 0.18) | 11 | 0.09(0.04 to 0.18) | 11 |
| High-income Asia Pacific | Congenital heart anomalies | 82.88(68.11 to 103.25) | 1 | 344.78(279.67 to 392.25) | 1 |
| High-income Asia Pacific | Congenital musculoskeletal and limb anomalies | 77.55(51.19 to 112.7) | 2 | 89.35(61.15 to 128.1) | 2 |
| High-income Asia Pacific | Other chromosomal abnormalities | 54.66(44.71 to 62.88) | 3 | 40.01(34.92 to 46.92) | 5 |
| High-income Asia Pacific | Other congenital birth defects | 36.24(28.07 to 63.53) | 4 | 85.45(69.79 to 110.79) | 3 |
| High-income Asia Pacific | Digestive congenital anomalies | 14.75(11.46 to 19.35) | 5 | 42.86(33.87 to 53.88) | 4 |
| High-income Asia Pacific | Down syndrome | 14.66(12.05 to 17.59) | 6 | 22.06(18.4 to 26.75) | 6 |
| High-income Asia Pacific | Urogenital congenital anomalies | 9.06(6.25 to 12.41) | 7 | 16.5(12.19 to 20.88) | 7 |
| High-income Asia Pacific | Neural tube defects | 8.98(7.27 to 10.96) | 8 | 13.89(11.71 to 16.54) | 8 |
| High-income Asia Pacific | Orofacial clefts | 3.29(2 to 5.06) | 9 | 5.02(3.22 to 7.57) | 9 |
| High-income Asia Pacific | Turner syndrome | 0.16(0.07 to 0.27) | 10 | 0.17(0.08 to 0.28) | 10 |
| High-income Asia Pacific | Klinefelter syndrome | 0.08(0.04 to 0.17) | 11 | 0.08(0.04 to 0.16) | 11 |
| High-income North America | Congenital heart anomalies | 100.75(88.02 to 118.62) | 1 | 243.19(212.07 to 263.27) | 1 |
| High-income North America | Other congenital birth defects | 79.57(68.7 to 103.21) | 2 | 141.53(112.03 to 158.63) | 2 |
| High-income North America | Other chromosomal abnormalities | 59.51(49.74 to 67.42) | 3 | 55.39(50.66 to 63.19) | 3 |
| High-income North America | Congenital musculoskeletal and limb anomalies | 41.55(31.25 to 55.41) | 4 | 48.04(34.45 to 63.55) | 4 |
| High-income North America | Down syndrome | 22.15(19.84 to 24.41) | 5 | 15.27(13.22 to 17.89) | 8 |
| High-income North America | Digestive congenital anomalies | 21.68(17.25 to 25.44) | 6 | 31.79(29.04 to 40.1) | 6 |
| High-income North America | Neural tube defects | 20.91(17.34 to 23.93) | 7 | 36.95(34.22 to 42.85) | 5 |
| High-income North America | Urogenital congenital anomalies | 13.1(9.36 to 16.59) | 8 | 20.04(14.77 to 27.11) | 7 |
| High-income North America | Orofacial clefts | 1.3(0.79 to 2.01) | 9 | 1.8(1.28 to 2.5) | 9 |
| High-income North America | Turner syndrome | 0.26(0.12 to 0.44) | 10 | 0.26(0.12 to 0.44) | 10 |
| High-income North America | Klinefelter syndrome | 0.11(0.05 to 0.22) | 11 | 0.11(0.05 to 0.22) | 11 |
| North Africa and Middle East | Congenital heart anomalies | 539.99(431.98 to 661.97) | 1 | 1670.33(755.19 to 2355.8) | 1 |
| North Africa and Middle East | Other congenital birth defects | 296.73(222.76 to 372.71) | 2 | 620(316.15 to 1107.57) | 2 |
| North Africa and Middle East | Neural tube defects | 72.57(54.89 to 95.42) | 3 | 298.93(145.34 to 422) | 3 |
| North Africa and Middle East | Congenital musculoskeletal and limb anomalies | 69.71(53.67 to 89.4) | 4 | 115.25(70.51 to 165.24) | 5 |
| North Africa and Middle East | Digestive congenital anomalies | 67.85(47.56 to 88.75) | 5 | 180.23(64.81 to 352.17) | 4 |
| North Africa and Middle East | Down syndrome | 48.56(35.55 to 75.13) | 6 | 100.46(41.55 to 245.37) | 6 |
| North Africa and Middle East | Other chromosomal abnormalities | 40.88(30.94 to 62.63) | 7 | 61.15(32.22 to 138.52) | 7 |
| North Africa and Middle East | Urogenital congenital anomalies | 24.73(16.63 to 36.79) | 8 | 49.58(27.49 to 86.44) | 8 |
| North Africa and Middle East | Orofacial clefts | 9.67(5.72 to 21.17) | 9 | 31.83(14.54 to 67.42) | 9 |
| North Africa and Middle East | Klinefelter syndrome | 0.09(0.04 to 0.18) | 10 | 0.09(0.04 to 0.17) | 10 |
| North Africa and Middle East | Turner syndrome | 0.07(0.03 to 0.11) | 11 | 0.07(0.03 to 0.12) | 11 |
| Oceania | Congenital heart anomalies | 786.51(364.8 to 1169.36) | 1 | 895.31(342.09 to 1311.21) | 1 |
| Oceania | Other congenital birth defects | 182.31(109.65 to 307.72) | 2 | 160.87(85.95 to 297.72) | 2 |
| Oceania | Neural tube defects | 134.7(69.17 to 217.35) | 3 | 134.97(66.7 to 227.06) | 3 |
| Oceania | Down syndrome | 122.16(42.87 to 217.51) | 4 | 92.8(26.59 to 222.69) | 4 |
| Oceania | Congenital musculoskeletal and limb anomalies | 50.23(34.11 to 72.31) | 5 | 49.74(32.82 to 73.01) | 5 |
| Oceania | Other chromosomal abnormalities | 29.85(10.85 to 58.06) | 6 | 44.35(14.75 to 106.17) | 6 |
| Oceania | Digestive congenital anomalies | 28.02(11.77 to 65.82) | 7 | 29.2(11.67 to 63.05) | 8 |
| Oceania | Orofacial clefts | 17.52(4.82 to 42.2) | 8 | 33.07(6.93 to 87.91) | 7 |
| Oceania | Urogenital congenital anomalies | 12.15(4.68 to 26.94) | 9 | 6.64(3.14 to 18.22) | 9 |
| Oceania | Turner syndrome | 0.11(0.05 to 0.19) | 10 | 0.11(0.05 to 0.19) | 10 |
| Oceania | Klinefelter syndrome | 0.08(0.04 to 0.17) | 11 | 0.08(0.04 to 0.17) | 11 |
| South Asia | Congenital heart anomalies | 324.04(234.07 to 448.15) | 1 | 652.5(414.83 to 870.95) | 1 |
| South Asia | Other congenital birth defects | 131.66(85.6 to 193.22) | 2 | 168.21(93.58 to 326.54) | 2 |
| South Asia | Digestive congenital anomalies | 58.84(33.86 to 97.43) | 3 | 89.01(39.82 to 161.72) | 4 |
| South Asia | Neural tube defects | 53.83(37.65 to 77.48) | 4 | 131.36(97.92 to 164.5) | 3 |
| South Asia | Congenital musculoskeletal and limb anomalies | 48.79(34.3 to 68.01) | 5 | 62.39(39.5 to 95.85) | 5 |
| South Asia | Other chromosomal abnormalities | 22.79(15.98 to 35.74) | 6 | 29.69(16.37 to 65.84) | 6 |
| South Asia | Down syndrome | 18.62(11.86 to 32.91) | 7 | 26.42(11.39 to 78.13) | 7 |
| South Asia | Urogenital congenital anomalies | 18.42(9.73 to 33.33) | 8 | 21.3(11.51 to 33.66) | 8 |
| South Asia | Orofacial clefts | 6.93(4.24 to 11.04) | 9 | 17.61(6.59 to 41.37) | 9 |
| South Asia | Turner syndrome | 0.11(0.05 to 0.2) | 10 | 0.11(0.05 to 0.21) | 10 |
| South Asia | Klinefelter syndrome | 0.09(0.04 to 0.17) | 11 | 0.09(0.04 to 0.18) | 11 |
| Southeast Asia | Congenital heart anomalies | 364.01(298.07 to 447.05) | 1 | 708.51(354.57 to 951.89) | 1 |
| Southeast Asia | Other congenital birth defects | 138.96(109.54 to 190.06) | 2 | 180.09(96.13 to 348.32) | 2 |
| Southeast Asia | Neural tube defects | 59.98(46.16 to 74.66) | 3 | 109.84(74.49 to 152.17) | 3 |
| Southeast Asia | Digestive congenital anomalies | 56.7(38.26 to 77.26) | 4 | 81.75(38.99 to 146.18) | 4 |
| Southeast Asia | Congenital musculoskeletal and limb anomalies | 51.02(38.21 to 67.02) | 5 | 59.36(40.86 to 82) | 5 |
| Southeast Asia | Other chromosomal abnormalities | 18.91(15.29 to 27.95) | 6 | 24.51(16 to 48.58) | 6 |
| Southeast Asia | Down syndrome | 18.85(14.89 to 24.8) | 7 | 19.63(10.51 to 44.71) | 7 |
| Southeast Asia | Orofacial clefts | 5.81(3.73 to 8.99) | 8 | 16.71(8.7 to 31.29) | 8 |
| Southeast Asia | Urogenital congenital anomalies | 4.27(2.79 to 6.85) | 9 | 5.64(3.7 to 11.22) | 9 |
| Southeast Asia | Turner syndrome | 0.11(0.05 to 0.19) | 10 | 0.12(0.05 to 0.21) | 10 |
| Southeast Asia | Klinefelter syndrome | 0.08(0.04 to 0.15) | 11 | 0.07(0.04 to 0.15) | 11 |
| Southern Latin America | Congenital heart anomalies | 224.37(186.05 to 272.12) | 1 | 386.08(324.42 to 452.41) | 1 |
| Southern Latin America | Other congenital birth defects | 133.97(108.82 to 169.94) | 2 | 217.62(136.06 to 279.71) | 2 |
| Southern Latin America | Congenital musculoskeletal and limb anomalies | 70.57(50.65 to 96.5) | 3 | 71.67(51.01 to 95.81) | 5 |
| Southern Latin America | Other chromosomal abnormalities | 61.03(50.79 to 72.83) | 4 | 34.18(29.63 to 40.2) | 7 |
| Southern Latin America | Digestive congenital anomalies | 59.39(45.76 to 74.21) | 5 | 87.99(72.25 to 111.77) | 4 |
| Southern Latin America | Neural tube defects | 44.71(35.9 to 55.26) | 6 | 121.32(101.53 to 147.97) | 3 |
| Southern Latin America | Down syndrome | 41.39(34.59 to 50.11) | 7 | 48.71(42.35 to 57.34) | 6 |
| Southern Latin America | Urogenital congenital anomalies | 26.21(15.84 to 35.22) | 8 | 27.57(19.93 to 43.1) | 8 |
| Southern Latin America | Orofacial clefts | 1.81(1.2 to 2.74) | 9 | 2.45(1.64 to 3.59) | 9 |
| Southern Latin America | Turner syndrome | 0.18(0.08 to 0.32) | 10 | 0.18(0.08 to 0.32) | 10 |
| Southern Latin America | Klinefelter syndrome | 0.04(0.02 to 0.08) | 11 | 0.04(0.02 to 0.08) | 11 |
| Southern Sub-Saharan Africa | Congenital heart anomalies | 195.26(135.75 to 257.04) | 1 | 239.92(195.26 to 304.84) | 1 |
| Southern Sub-Saharan Africa | Other congenital birth defects | 176.81(117.48 to 232.53) | 2 | 187.36(145.86 to 225.98) | 2 |
| Southern Sub-Saharan Africa | Congenital musculoskeletal and limb anomalies | 65.77(48.2 to 85.99) | 3 | 59.91(43.58 to 76.65) | 4 |
| Southern Sub-Saharan Africa | Down syndrome | 48.12(31.26 to 64.19) | 4 | 43.56(34.96 to 59.07) | 6 |
| Southern Sub-Saharan Africa | Digestive congenital anomalies | 47.51(27.27 to 74.32) | 5 | 58.03(40.7 to 73.74) | 5 |
| Southern Sub-Saharan Africa | Other chromosomal abnormalities | 47.45(32.88 to 61.89) | 6 | 41.01(32.58 to 52.23) | 7 |
| Southern Sub-Saharan Africa | Neural tube defects | 43.13(30.68 to 57.36) | 7 | 63.68(47.96 to 78.02) | 3 |
| Southern Sub-Saharan Africa | Urogenital congenital anomalies | 10.97(7.51 to 15.64) | 8 | 9.82(6.82 to 13.85) | 9 |
| Southern Sub-Saharan Africa | Orofacial clefts | 7.68(4.89 to 11.37) | 9 | 11(6.91 to 15.22) | 8 |
| Southern Sub-Saharan Africa | Turner syndrome | 0.13(0.06 to 0.23) | 10 | 0.13(0.06 to 0.23) | 10 |
| Southern Sub-Saharan Africa | Klinefelter syndrome | 0.09(0.05 to 0.19) | 11 | 0.09(0.04 to 0.18) | 11 |
| Tropical Latin America | Congenital heart anomalies | 275.39(224.81 to 331.72) | 1 | 470.57(403.47 to 544.03) | 1 |
| Tropical Latin America | Other congenital birth defects | 145.3(102.12 to 179.37) | 2 | 169.45(136.55 to 230.28) | 2 |
| Tropical Latin America | Digestive congenital anomalies | 74.54(59.73 to 92.91) | 3 | 95.09(78.62 to 119.03) | 3 |
| Tropical Latin America | Other chromosomal abnormalities | 52.98(43.04 to 67.39) | 4 | 20.92(15.74 to 26.34) | 7 |
| Tropical Latin America | Neural tube defects | 46.36(36.16 to 59.87) | 5 | 83.24(70.63 to 97.82) | 4 |
| Tropical Latin America | Congenital musculoskeletal and limb anomalies | 46.32(34.64 to 62.24) | 6 | 44.37(33.95 to 56.65) | 5 |
| Tropical Latin America | Down syndrome | 31.6(26.29 to 38.77) | 7 | 40.54(31.96 to 48.84) | 6 |
| Tropical Latin America | Urogenital congenital anomalies | 21.41(11.62 to 28.43) | 8 | 18.8(13.16 to 31.76) | 8 |
| Tropical Latin America | Orofacial clefts | 2.5(1.8 to 3.42) | 9 | 10.84(7.9 to 13.14) | 9 |
| Tropical Latin America | Turner syndrome | 0.13(0.06 to 0.22) | 10 | 0.12(0.06 to 0.22) | 10 |
| Tropical Latin America | Klinefelter syndrome | 0.05(0.02 to 0.1) | 11 | 0.05(0.02 to 0.1) | 11 |
| Western Europe | Congenital heart anomalies | 90.56(76.37 to 106.5) | 1 | 285.35(247.62 to 308.59) | 1 |
| Western Europe | Other congenital birth defects | 59.06(47.69 to 81.21) | 2 | 127.65(96.59 to 147.59) | 2 |
| Western Europe | Other chromosomal abnormalities | 44.24(38.11 to 50.7) | 3 | 45.53(40.85 to 50.69) | 5 |
| Western Europe | Congenital musculoskeletal and limb anomalies | 44.07(31.27 to 60.92) | 4 | 53.42(40.26 to 70.05) | 3 |
| Western Europe | Down syndrome | 30.54(27.26 to 33.85) | 5 | 24.74(21.67 to 28.1) | 7 |
| Western Europe | Digestive congenital anomalies | 18.35(15.45 to 22.59) | 6 | 46.64(39.27 to 59.78) | 4 |
| Western Europe | Neural tube defects | 13.11(10.95 to 15.4) | 7 | 34.84(32.23 to 39.5) | 6 |
| Western Europe | Urogenital congenital anomalies | 9.32(6.49 to 12.15) | 8 | 15.44(11.11 to 23.44) | 8 |
| Western Europe | Orofacial clefts | 1.74(1.09 to 2.59) | 9 | 3.03(2.25 to 4.05) | 9 |
| Western Europe | Turner syndrome | 0.19(0.09 to 0.32) | 10 | 0.19(0.09 to 0.32) | 10 |
| Western Europe | Klinefelter syndrome | 0.13(0.06 to 0.28) | 11 | 0.12(0.06 to 0.26) | 11 |
| Western Sub-Saharan Africa | Congenital heart anomalies | 520.37(326.58 to 724.49) | 1 | 802.14(235.79 to 1218.58) | 1 |
| Western Sub-Saharan Africa | Other congenital birth defects | 254.16(182.94 to 392.68) | 2 | 287.67(151.04 to 588.81) | 3 |
| Western Sub-Saharan Africa | Neural tube defects | 190.43(138.72 to 255.08) | 3 | 317.45(141.12 to 483.04) | 2 |
| Western Sub-Saharan Africa | Digestive congenital anomalies | 140.99(88.96 to 192.78) | 4 | 176.35(57.61 to 279.05) | 4 |
| Western Sub-Saharan Africa | Down syndrome | 72.09(42.7 to 150.68) | 5 | 69.52(22.74 to 196.86) | 5 |
| Western Sub-Saharan Africa | Congenital musculoskeletal and limb anomalies | 59.8(43.49 to 81.58) | 6 | 60.37(44.06 to 83.17) | 6 |
| Western Sub-Saharan Africa | Other chromosomal abnormalities | 52.75(38.54 to 92.5) | 7 | 46.94(24.42 to 108.9) | 7 |
| Western Sub-Saharan Africa | Urogenital congenital anomalies | 12.94(7.97 to 25.49) | 8 | 12.68(6.98 to 29.64) | 8 |
| Western Sub-Saharan Africa | Orofacial clefts | 7.26(3.02 to 23.27) | 9 | 10.88(3.22 to 47.22) | 9 |
| Western Sub-Saharan Africa | Turner syndrome | 0.12(0.06 to 0.22) | 10 | 0.12(0.06 to 0.22) | 10 |
| Western Sub-Saharan Africa | Klinefelter syndrome | 0.09(0.04 to 0.17) | 11 | 0.09(0.04 to 0.17) | 11 |

## Table S3 DALYs of CHD, NTDs, DCAs, and DS in 1990 and 2021 by different characteristics

| **Characteristics** | **Congenital heart anomalies** | | | | **Neural tube defects** | | | | **Digestive congenital anomalies** | | | | **Down syndrome** | | | |
| --- | --- | --- | --- | --- | --- | --- | --- | --- | --- | --- | --- | --- | --- | --- | --- | --- |
|  | **1990** | | **2021** | | **1990** | | **2021** | | **1990** | | **2021** | | **1990** | | **2021** | |
|  | **All ages number (95% UI)** | **ASDR (95% UI)** | **All ages number (95% UI)** | **ASDR (95% UI)** | **All ages number (95% UI)** | **ASDR (95% UI)** | **All ages number (95% UI)** | **ASDR (95% UI)** | **All ages number (95% UI)** | **ASDR (95% UI)** | **All ages number (95% UI)** | **ASDR (95% UI)** | **All ages number (95% UI)** | **ASDR (95% UI)** | **All ages number (95% UI)** | **ASDR (95% UI)** |
| Overall | 46825479.57(27458859.18 to 60026261.1) | 750.3(440.94 to 960.11) | 22310093.48(18696085.02 to 27213661.74) | 345.24(288.34 to 422.16) | 10205310.18(7055129.84 to 13250974.23) | 161.8(111.67 to 210.07) | 5294602.55(4326463.67 to 6485533.17) | 83.18(67.94 to 102.1) | 6835818.74(3845407.14 to 10517048.31) | 108.3(60.88 to 166.69) | 4430696.4(3345178.04 to 5508669.1) | 70.44(53.14 to 87.6) | 2803958.38(1601880.66 to 6346043.23) | 45.49(26.17 to 102.27) | 2353047.59(1759096.25 to 3754933.89) | 35.2(26 to 57.17) |
| Sex |  |  |  |  |  |  |  |  |  |  |  |  |  |  |  |  |
| Male | 27117571.24(14525909.83 to 37614375.36) | 840.45(452.13 to 1165.71) | 12424474.8(9861186.81 to 16087943.37) | 373.74(294.98 to 485.29) | 4856660.78(3627475.79 to 6897280.19) | 148.62(111.02 to 210.81) | 2578571.22(2019473.2 to 3389917.73) | 78.6(61.42 to 103.6) | 3493055.21(1958320.35 to 5898837.85) | 106.8(59.93 to 180.53) | 2291390.16(1592493.36 to 3116181.51) | 70.66(48.99 to 96.31) | 1324244.08(813869.91 to 2865628.78) | 41.66(25.97 to 89.26) | 1145520.66(852275.31 to 1747686.24) | 33.35(24.54 to 51.63) |
| Female | 19707908.33(10877580.62 to 25436356.78) | 653.77(361.07 to 843.73) | 9885618.68(7761680.36 to 11938135.68) | 314.77(246 to 381.74) | 5348649.41(2798468.87 to 7444733.28) | 175.8(91.77 to 244.92) | 2716031.34(1987758.71 to 3610076.05) | 88.07(64.32 to 117.48) | 3342763.53(1541004.08 to 5556764.23) | 109.83(50.58 to 182.67) | 2139306.24(1393461.05 to 2866335.67) | 70.2(45.77 to 94.1) | 1479714.3(610075.65 to 3692467.61) | 49.56(20.79 to 122.99) | 1207526.93(863327.71 to 2216221.74) | 37.19(26.18 to 69.84) |
| Sociodemographic index |  |  |  |  |  |  |  |  |  |  |  |  |  |  |  |  |
| Low SDI | 8223595.17(3037463.45 to 11965775.27) | 860.62(324.42 to 1252.88) | 8087036.82(5745670.88 to 10888494.76) | 488.54(349.65 to 658.02) | 3262553.82(1838091.52 to 4677429.85) | 331.38(185.79 to 477.62) | 2942594.13(2213181.95 to 3768424.07) | 175.35(131.63 to 224.19) | 1493963.82(509889.15 to 2571785.8) | 149.39(50.91 to 259.22) | 1751664.84(1166025.2 to 2400528.48) | 103.58(69.24 to 141.71) | 644912.8(172740.6 to 2158601.82) | 71.55(20.85 to 231.96) | 916555.16(523339.99 to 1970519.71) | 57.85(34.1 to 120.9) |
| Low-middle SDI | 14028159.95(7577696.38 to 18951964.11) | 796.79(430.97 to 1074.7) | 7235428.34(5743374.11 to 9023954.37) | 381.65(302.67 to 476.97) | 3117286.39(2121211.76 to 3914216.23) | 173.69(118.08 to 218.61) | 1487255.39(1168088.2 to 1946912.36) | 78.79(61.81 to 103.33) | 1986746.41(1015593.26 to 3366578.26) | 110.01(56.35 to 186.25) | 1438299.37(1025491.55 to 1873475.64) | 76.78(54.74 to 100.1) | 653765.72(309950.66 to 1740536.46) | 38.82(18.85 to 101.07) | 585559.62(424142.18 to 876816.42) | 30.86(22.28 to 46.27) |
| Middle SDI | 15291640.89(9702218.63 to 20084169.61) | 768.69(487.96 to 1009.29) | 4877621.26(4144778.88 to 5756294.86) | 286.65(241.63 to 342.81) | 2517981.27(1939051.91 to 3166662.55) | 126.16(97.12 to 158.66) | 613504.62(506810.92 to 753645.06) | 37.3(30.57 to 46.22) | 2026788.02(1248360.64 to 3260953.91) | 101.75(62.68 to 163.81) | 885326.82(668275.71 to 1131375.47) | 55.53(41.77 to 71.29) | 863235.37(576363.14 to 1523578.09) | 43.52(29.15 to 76.69) | 447594.08(382705.03 to 540762.33) | 25.03(21.16 to 30.43) |
| High-middle SDI | 7137006.4(5102925.14 to 8743351.62) | 783.96(558.45 to 962.93) | 1429192.44(1222822.25 to 1661698.36) | 204.05(171.77 to 239.89) | 1060409.35(827907.47 to 1267523.64) | 118.35(92.43 to 141.64) | 147667.84(122995.66 to 173166.05) | 21.7(17.6 to 25.41) | 1043426.21(663553.03 to 1517513.94) | 117.35(74.59 to 170.65) | 240251.52(181063.42 to 306892.64) | 39.58(29.49 to 50.79) | 477492.73(350161.81 to 691150.6) | 51.97(37.94 to 75.7) | 181197.14(154092.99 to 211552.16) | 23.92(19.56 to 28.38) |
| High SDI | 2106263.64(1838806.92 to 2305809.6) | 326.39(283.76 to 357.66) | 656868.92(564953.74 to 765382.65) | 101.7(87.06 to 118.34) | 240152.97(224136.94 to 266778.4) | 37.79(35.29 to 42.11) | 99200.91(84187.82 to 114005.35) | 15.5(12.97 to 17.62) | 279181.51(242195.44 to 331968.02) | 45.17(39.17 to 53.8) | 111813.1(90825.02 to 132028.35) | 20.26(16.29 to 24.18) | 162159.92(137328.23 to 196305.47) | 22.75(19.21 to 28.27) | 219274.13(199625.07 to 240549.63) | 23.86(21.42 to 26.52) |
| GBD regions |  |  |  |  |  |  |  |  |  |  |  |  |  |  |  |  |
| Andean Latin America | 471002.75(257527.36 to 611575.33) | 871.67(478.13 to 1128.92) | 220004.12(170363.08 to 277181.68) | 360.59(278.13 to 454.8) | 70461.62(45584.29 to 94500.68) | 128.26(83.28 to 171.55) | 26464.42(20110.25 to 34876.77) | 43.67(33.06 to 57.75) | 96932.41(50921.84 to 155543.19) | 175.65(92.22 to 281.96) | 55492.11(39409.61 to 73498.25) | 92.32(65.51 to 122.41) | 45933.79(23268.55 to 90866.08) | 85.74(43.78 to 168.59) | 23494.58(18011.6 to 31456.79) | 38.31(29.29 to 51.35) |
| Australasia | 29619.59(27175.13 to 32648.95) | 180.95(165.96 to 199.09) | 16014.88(13206.2 to 18893.23) | 75.26(60.82 to 90.15) | 8365.81(7474.01 to 9316.66) | 52.29(46.64 to 58.42) | 3300.17(2633.02 to 4115.37) | 16.04(12.33 to 20.22) | 7294.35(6447.03 to 8917.15) | 47.1(41.62 to 57.62) | 2605.4(2063.23 to 3846.37) | 14.24(11.25 to 21.14) | 4052.06(3440.86 to 4744.59) | 22.56(19.24 to 26.36) | 7235.53(6373.37 to 8184.17) | 27.03(22.82 to 31.24) |
| Caribbean | 361441.42(275554.96 to 448629.41) | 860.63(654.68 to 1067.15) | 231279.77(146052.98 to 385331.97) | 594.06(374.75 to 982.65) | 53970.09(37610.93 to 78721.67) | 127.64(89.03 to 186.11) | 31017.78(18759.46 to 53265.47) | 79.62(48.19 to 136.32) | 71840.4(41627.87 to 117411.25) | 168.55(97.53 to 275.7) | 44573.26(26949.31 to 70630.36) | 116.09(70.06 to 184.27) | 24485.56(12363.53 to 58581.49) | 61.12(31.88 to 142.72) | 24550.38(13948.63 to 44912.21) | 58.54(32.03 to 108.79) |
| Central Asia | 385254.35(335781.22 to 435341.14) | 417.57(363.7 to 471.6) | 418324.54(332291.07 to 513640.2) | 423.52(336.81 to 519.76) | 91514.58(74661.11 to 107058.53) | 98.13(80.42 to 114.51) | 28845.33(22224.62 to 39193.66) | 29.35(22.66 to 39.77) | 68019.23(53451.43 to 85756.39) | 72.63(57.18 to 91.48) | 57128.94(45514.35 to 72575.7) | 57.85(46.1 to 73.44) | 21182.27(14720.37 to 34992.98) | 23(16.14 to 37.66) | 15508.44(11523.68 to 22537.3) | 15.72(11.71 to 22.81) |
| Central Europe | 471753.27(407794.81 to 522236.58) | 535.78(461.97 to 595.51) | 85405.28(71840.92 to 98566.25) | 143.86(120.48 to 167.77) | 76209.49(66589.06 to 91999.69) | 88.17(77.11 to 106.46) | 8101.54(6542.42 to 9821.3) | 12.37(10.01 to 14.96) | 83049.71(62523.87 to 106807.15) | 98.06(73.74 to 126.35) | 14159.35(11190.3 to 17380.45) | 26.49(20.89 to 32.86) | 15854.45(13190.45 to 20036.9) | 16.18(13.51 to 20.75) | 10447.45(8709.48 to 12194.67) | 13.42(11.12 to 15.94) |
| Central Latin America | 1100103.84(973615.14 to 1244777.02) | 476.47(421.91 to 537.81) | 779514.37(623841.59 to 975887.01) | 390.66(308.43 to 492.21) | 338991.44(271085.52 to 381497.2) | 144.14(115.59 to 162.04) | 77542.09(62362.71 to 95898.03) | 38.71(30.64 to 48.3) | 240446.17(207303.9 to 297493.85) | 101.79(87.82 to 125.78) | 178876.82(133911.87 to 231108.39) | 93.06(69.56 to 120.37) | 79562.64(68970.28 to 89851.96) | 35.51(30.85 to 39.91) | 79490.49(63426.29 to 99926.59) | 36.91(29.03 to 46.58) |
| Central Sub-Saharan Africa | 698101.37(218649.58 to 1266655.62) | 628.73(203.49 to 1126.97) | 589407.29(378883.04 to 939737.11) | 288.04(186.1 to 458.16) | 464530.06(248896.16 to 661296.49) | 403.34(213.8 to 577.52) | 360869.01(222118.41 to 518959.16) | 172.78(106.72 to 248.54) | 166056.83(41902.22 to 321243.84) | 142.84(36.58 to 275.3) | 149840.42(81774.93 to 258601.37) | 71.28(39.19 to 122.63) | 80798.03(22444.01 to 260846.86) | 75.46(21.66 to 240.73) | 104838.92(53415.12 to 229941.07) | 52.87(27.52 to 114.52) |
| East Asia | 11180599.18(7172266.41 to 15360041.67) | 962.04(614.3 to 1322.94) | 1782703.46(1440585.41 to 2196782.62) | 241.17(191.48 to 305.04) | 1510844.55(1094764.46 to 1905359.78) | 131.35(95.15 to 165.69) | 109495.64(78660.24 to 141894.21) | 16.81(11.59 to 22.32) | 1311196.5(710433.84 to 2364307.24) | 114.24(61.8 to 206.16) | 242575.25(156670.24 to 353726.87) | 40.15(25.59 to 59.03) | 661081.66(441546.87 to 1035385.05) | 57.11(37.92 to 89.69) | 153626.36(92733.66 to 208057.2) | 23.02(13.17 to 31.61) |
| Eastern Europe | 729767.68(650811.4 to 862950.48) | 463.92(410.99 to 551.64) | 170666.7(147400.52 to 199596.82) | 150.6(126.77 to 179.24) | 119562.95(106305.46 to 139366.93) | 77.66(68.82 to 90.62) | 18662.57(15516.81 to 22760.7) | 15.18(12.65 to 18.2) | 163262.38(130917.35 to 191568.16) | 110.05(88.02 to 129.44) | 30346.35(23492.84 to 39494.41) | 31.88(24.29 to 41.44) | 55978.38(49758.64 to 65755.61) | 33.54(29.93 to 39.73) | 16820.51(14181.31 to 20208.52) | 13.36(11.17 to 16.11) |
| Eastern Sub-Saharan Africa | 2579080.31(741067.21 to 4968769.09) | 668.98(200.64 to 1291.08) | 2119016.15(1337315.83 to 3731588.62) | 334.69(214.9 to 585.93) | 1520104.51(887583.73 to 2294501.07) | 382.12(222.41 to 580.12) | 1238691.35(812641.27 to 1746476.58) | 192.6(126.36 to 272.07) | 614031.27(147699.97 to 1258571.82) | 152.5(37.46 to 312.24) | 598171.53(335578.01 to 963135.06) | 92.69(52.8 to 148.72) | 297571.19(61928.08 to 1027321.96) | 80.8(18.32 to 275.11) | 330733.64(182882.41 to 687355.9) | 53.79(30.29 to 109.87) |
| High-income Asia Pacific | 374999.27(307490.74 to 424326.83) | 344.78(279.67 to 392.25) | 76963.49(61679.27 to 96224.34) | 82.88(68.11 to 103.25) | 15892.39(13236.81 to 18896.64) | 13.89(11.71 to 16.54) | 8872.36(7013.86 to 11229.68) | 8.98(7.27 to 10.96) | 43279.76(34230.22 to 54473.39) | 42.86(33.87 to 53.88) | 10602.33(8387.08 to 13503.32) | 14.75(11.46 to 19.35) | 27088.54(22292.61 to 32985.57) | 22.06(18.4 to 26.75) | 15168.22(12628.85 to 18103.46) | 14.66(12.05 to 17.59) |
| High-income North America | 560357.09(492240.32 to 604895.58) | 243.19(212.07 to 263.27) | 241407.24(211314.33 to 283431.47) | 100.75(88.02 to 118.62) | 83112.1(76911.81 to 96219.56) | 36.95(34.22 to 42.85) | 48441.84(41041.05 to 55331.33) | 20.91(17.34 to 23.93) | 71110.62(64983.58 to 89568.82) | 31.79(29.04 to 40.1) | 46118.1(37195.12 to 53547.38) | 21.68(17.25 to 25.44) | 38550.42(33197.06 to 45188.52) | 15.27(13.22 to 17.89) | 79705.65(70631.09 to 87983.27) | 22.15(19.84 to 24.41) |
| North Africa and Middle East | 8560419.3(3849442.58 to 12096037.34) | 1670.33(755.19 to 2355.8) | 3181543.55(2544863.87 to 3899408.43) | 539.99(431.98 to 661.97) | 1525816.18(734580.69 to 2156726.58) | 298.93(145.34 to 422) | 430798.26(325049.77 to 564788.42) | 72.57(54.89 to 95.42) | 932231.88(333934.49 to 1820621.85) | 180.23(64.81 to 352.17) | 394779.86(276837.24 to 516010.83) | 67.85(47.56 to 88.75) | 505412.43(206005.25 to 1247306.23) | 100.46(41.55 to 245.37) | 290927.56(214000.18 to 447222.31) | 48.56(35.55 to 75.13) |
| Oceania | 91257.9(33649.1 to 134508.73) | 895.31(342.09 to 1311.21) | 154424.81(69112.93 to 231035.78) | 786.51(364.8 to 1169.36) | 14230.54(6964.66 to 24000.93) | 134.97(66.7 to 227.06) | 27228.04(13893.49 to 44061.67) | 134.7(69.17 to 217.35) | 3047.32(1184.83 to 6608) | 29.2(11.67 to 63.05) | 5607.48(2287.77 to 13294.65) | 28.02(11.77 to 65.82) | 9168.24(2461.89 to 22569.99) | 92.8(26.59 to 222.69) | 23578.05(7866.6 to 42722.63) | 122.16(42.87 to 217.51) |
| South Asia | 10254280.66(6526703.03 to 13707205.29) | 652.5(414.83 to 870.95) | 5102105.76(3703401.2 to 7010943.54) | 324.04(234.07 to 448.15) | 2106690.81(1568442.55 to 2636289.81) | 131.36(97.92 to 164.5) | 836429.64(587873.71 to 1195752.21) | 53.83(37.65 to 77.48) | 1433467.96(636837.36 to 2611667.06) | 89.01(39.82 to 161.72) | 899742.49(519492.18 to 1485571.61) | 58.84(33.86 to 97.43) | 389881.64(161346.76 to 1198604.04) | 26.42(11.39 to 78.13) | 301470.02(195337.35 to 523591.97) | 18.62(11.86 to 32.91) |
| Southeast Asia | 4133016.41(2062756.05 to 5556043.56) | 708.51(354.57 to 951.89) | 2053889.57(1683426.46 to 2518299.4) | 364.01(298.07 to 447.05) | 646474.89(438154 to 895508.84) | 109.84(74.49 to 152.17) | 329367.38(254424.69 to 409580.75) | 59.98(46.16 to 74.66) | 479014.51(228438.9 to 856110.83) | 81.75(38.99 to 146.18) | 311254.85(210787.96 to 422330.73) | 56.7(38.26 to 77.26) | 113192.46(60019.11 to 259283.64) | 19.63(10.51 to 44.71) | 110407.36(87951.8 to 143462.91) | 18.85(14.89 to 24.8) |
| Southern Latin America | 196046.23(164726.46 to 229771.32) | 386.08(324.42 to 452.41) | 93222.87(78307.88 to 111106.53) | 224.37(186.05 to 272.12) | 61670.04(51616.73 to 75187.54) | 121.32(101.53 to 147.97) | 19254.88(15815.98 to 23362.34) | 44.71(35.9 to 55.26) | 44651.92(36665.48 to 56715.99) | 87.99(72.25 to 111.77) | 22985.88(17892.63 to 28662.66) | 59.39(45.76 to 74.21) | 24635.26(21409.69 to 29018.02) | 48.71(42.35 to 57.34) | 19796.95(16993.28 to 23398.26) | 41.39(34.59 to 50.11) |
| Southern Sub-Saharan Africa | 176341.24(143181.75 to 225381.51) | 239.92(195.26 to 304.84) | 155311.2(108192.73 to 203959.59) | 195.26(135.75 to 257.04) | 47296.48(35494.93 to 58477.27) | 63.68(47.96 to 78.02) | 34318.58(24510.37 to 45563.71) | 43.13(30.68 to 57.36) | 43781.87(30606.17 to 55629.24) | 58.03(40.7 to 73.74) | 37386.7(21500.42 to 58359.55) | 47.51(27.27 to 74.32) | 31763.87(25340.28 to 43393.86) | 43.56(34.96 to 59.07) | 38146.53(24825.26 to 50829.82) | 48.12(31.26 to 64.19) |
| Tropical Latin America | 766836.45(658138.06 to 885927.31) | 470.57(403.47 to 544.03) | 476225.39(390102.7 to 570458.6) | 275.39(224.81 to 331.72) | 134672.54(114281.29 to 158225.42) | 83.24(70.63 to 97.82) | 78670.44(61562.36 to 101346.58) | 46.36(36.16 to 59.87) | 153865.79(127202.55 to 192553.29) | 95.09(78.62 to 119.03) | 126329.08(101584.75 to 156944.01) | 74.54(59.73 to 92.91) | 66260.46(52349.82 to 79806.43) | 40.54(31.96 to 48.84) | 59957.6(50836.37 to 73194.83) | 31.6(26.29 to 38.77) |
| Western Europe | 709036.35(616259.49 to 765568.37) | 285.35(247.62 to 308.59) | 236869.49(198209.03 to 282630.64) | 90.56(76.37 to 106.5) | 86014.44(79417.8 to 97260.67) | 34.84(32.23 to 39.5) | 34257.04(28846.74 to 40864.01) | 13.11(10.95 to 15.4) | 108319.24(91131.26 to 138048.37) | 46.64(39.27 to 59.78) | 40699.03(34639.66 to 49428.61) | 18.35(15.45 to 22.59) | 74884.46(65055.88 to 85279.66) | 24.74(21.67 to 28.1) | 118346.71(109714.2 to 128524.97) | 30.54(27.26 to 33.85) |
| Western Sub-Saharan Africa | 2996164.91(829755.56 to 4546534.07) | 802.14(235.79 to 1218.58) | 4125793.54(2522840.1 to 5783212.63) | 520.37(326.58 to 724.49) | 1228884.68(542639.68 to 1868199.55) | 317.45(141.12 to 483.04) | 1543974.2(1111721.38 to 2081571.64) | 190.43(138.72 to 255.08) | 700918.63(228476.86 to 1101348.8) | 176.35(57.61 to 279.05) | 1161421.17(731555.5 to 1590929.09) | 140.99(88.96 to 192.78) | 236620.58(67801.23 to 715139.94) | 69.52(22.74 to 196.86) | 528796.66(294901.72 to 1173109.17) | 72.09(42.7 to 150.68) |
| Countries and territories |  |  |  |  |  |  |  |  |  |  |  |  |  |  |  |  |
| Afghanistan | 576335.46(136487.74 to 945079.36) | 3002.73(705.33 to 4981.45) | 774686.35(383494.94 to 1098663.81) | 1395.89(691.74 to 1982.48) | 111139.65(34288.42 to 185513.89) | 595.66(182.68 to 998.34) | 100117.09(55929.92 to 153939.01) | 185.88(104.29 to 285.14) | 46962.68(8921.83 to 115425.35) | 236.07(44.63 to 577.4) | 73276.44(35684.86 to 129600.18) | 128.82(62.73 to 227.94) | 28577.61(4175.3 to 104031.73) | 156.03(23.56 to 554.38) | 40776.68(18529.27 to 91585.88) | 76.58(34.63 to 167.55) |
| Albania | 18968.33(14092.53 to 25799.15) | 486.05(361.66 to 660.37) | 3779.34(2425.82 to 5475.67) | 268.21(169.58 to 393.24) | 4001.28(2543.35 to 6383.9) | 102.64(65.29 to 163.46) | 607.01(403.54 to 870.62) | 42.13(27.33 to 61.39) | 1472.89(841.99 to 2298.27) | 37.78(21.6 to 58.93) | 354.54(191.43 to 648.06) | 25.52(13.5 to 47.04) | 347.12(201.54 to 738.47) | 9.04(5.29 to 19.11) | 176.77(126.3 to 268.62) | 10.03(6.79 to 15.98) |
| Algeria | 602633.66(296566.2 to 846833.52) | 1648.03(815.44 to 2313.48) | 205438.79(155409.56 to 262885.05) | 461.71(348.67 to 590.34) | 84631.99(39443.15 to 120741.85) | 232.04(108.78 to 330.45) | 25448.73(17434 to 36226.46) | 57.26(38.89 to 81.7) | 61501.8(26144.93 to 112352.33) | 167.65(71.38 to 305.94) | 31062.62(19897.72 to 44159.1) | 70.28(45.01 to 100.13) | 45142.02(17097.05 to 109845.57) | 125.11(47.69 to 302.19) | 42451.97(20319.8 to 80341.06) | 95.11(45.42 to 180.49) |
| American Samoa | 173.59(133.59 to 223.27) | 226.86(176.48 to 287.9) | 72.87(51.94 to 99.2) | 192.43(134.39 to 267.45) | 14.15(10.01 to 20.26) | 17.85(12.66 to 25.19) | 6.44(4.02 to 9.8) | 18.31(10.87 to 28.4) | 8.68(3.99 to 26.25) | 11.03(5.3 to 31.95) | 3.27(1.66 to 11.94) | 9.05(4.29 to 35.52) | 29.17(18.26 to 41.82) | 39.2(24.87 to 55.42) | 21.15(9.88 to 33.76) | 55.85(25.15 to 92.57) |
| Andorra | 64.76(48.02 to 83.42) | 205.38(146.74 to 263.34) | 23.35(15.43 to 31.73) | 47.32(33.64 to 62.27) | 7.16(4.86 to 10.1) | 22.5(14.54 to 32.68) | 2.8(2.07 to 3.65) | 5.32(3.86 to 7.09) | 10.76(6.18 to 16.8) | 38.43(21.19 to 60.56) | 2.66(1.82 to 3.68) | 7.12(4.65 to 10.24) | 5.98(4.46 to 8.07) | 13.36(9.7 to 18.78) | 12.72(8.72 to 18.45) | 16.38(11.11 to 24.2) |
| Angola | 151757.77(38521.26 to 288589.57) | 721.29(188.23 to 1355.19) | 171395.36(110471.19 to 276758.14) | 313.37(205.06 to 505.06) | 90252.6(38032.99 to 150736.26) | 412.06(172.68 to 692.92) | 92732.2(59323.95 to 134038.16) | 165.27(106.79 to 237.35) | 32162.46(6368.01 to 69239.83) | 144.6(29.38 to 309.95) | 45180.08(23089.19 to 70535.78) | 79.77(41.39 to 124) | 17228.3(3281.82 to 62291.76) | 85.09(17.25 to 301.9) | 30620.85(14529.01 to 72519.52) | 57.72(27.87 to 133.63) |
| Antigua and Barbuda | 165.05(137.47 to 197.34) | 276.37(229.57 to 330.81) | 125.41(106.4 to 143.72) | 222.28(186.1 to 257.75) | 15.68(11.91 to 19.67) | 26.21(19.8 to 32.95) | 10.11(8.33 to 12.04) | 16.98(13.68 to 20.28) | 18.03(13.79 to 28.87) | 30.52(23.31 to 48.91) | 13.88(11.32 to 20.96) | 26.16(21.29 to 39.63) | 10.03(7.15 to 14.11) | 17.17(12.35 to 23.99) | 17.51(13.55 to 21.94) | 23.71(17.86 to 30.62) |
| Argentina | 142794.28(117455.02 to 170145) | 426.82(350.99 to 508.69) | 73664.13(60625.72 to 89419.53) | 259.66(210.94 to 319.85) | 42217.99(34243.77 to 53007.74) | 126.09(102.25 to 158.41) | 14286.07(11489.58 to 17755.5) | 48.24(37.89 to 60.63) | 30119.66(23494.91 to 39679.77) | 90.23(70.35 to 118.88) | 17873.75(13279.36 to 22880.83) | 67.17(49.59 to 86.29) | 14235.52(11626.6 to 17830.17) | 42.67(34.86 to 53.44) | 13615.11(11508.32 to 16575.11) | 42.91(35.28 to 53.92) |
| Armenia | 18874.79(14591.36 to 24180.36) | 515.32(399.04 to 659.4) | 5852.16(4597.98 to 7425.12) | 311.25(243.22 to 398.16) | 7174.55(5214.22 to 9224.14) | 196.74(143.05 to 252.89) | 535.85(416.19 to 700.52) | 27.24(20.91 to 36.13) | 6940.27(4015.34 to 9616.34) | 189.89(109.83 to 263.05) | 1815.37(1281.51 to 2371.68) | 104.73(73.54 to 137.45) | 1386.63(607.16 to 2023.39) | 37.69(16.6 to 54.95) | 343.87(241.3 to 484.74) | 18.04(12.71 to 25.54) |
| Australia | 23760.08(21660.89 to 26433.59) | 177.91(162 to 197.43) | 13092.02(10749.64 to 15561.89) | 74.29(59.53 to 89.22) | 6704.95(5980.77 to 7595.3) | 51.51(45.83 to 58.31) | 2731.93(2145.04 to 3454.99) | 15.95(12.08 to 20.41) | 5936.06(5053.8 to 7491.54) | 47.23(40.19 to 59.66) | 2063.36(1575.11 to 3212.03) | 13.61(10.33 to 21.33) | 3326.99(2782.89 to 3948.28) | 22.55(18.93 to 26.57) | 5756.83(5024.85 to 6535.17) | 25.84(21.68 to 29.96) |
| Austria | 14251.78(11815.56 to 15790.97) | 290.24(239.51 to 320.65) | 4903.24(3840.14 to 6093.3) | 88.63(72.06 to 107.87) | 1278.83(1126.5 to 1466.96) | 25.95(22.76 to 30.13) | 568.5(467.26 to 705.28) | 10.32(8.46 to 12.57) | 2638.33(2253.46 to 3278.08) | 57.1(48.7 to 71.34) | 886.96(716.68 to 1131.21) | 19.16(15.21 to 25.09) | 1663.97(1452.82 to 1894.01) | 27.4(23.99 to 30.98) | 2734.57(2436.07 to 3057.37) | 36.2(31.2 to 41.13) |
| Azerbaijan | 61303.02(45469.91 to 75235.35) | 698.74(518.87 to 857.36) | 28053.63(18138.02 to 43349.74) | 405.83(259.87 to 635.46) | 10991.11(7421.22 to 14342.2) | 124.45(84.07 to 162.29) | 3486.59(2448.64 to 5195.12) | 51.48(35.44 to 77.59) | 6578.06(3524.37 to 11137.79) | 74.72(40.06 to 126.59) | 3236.88(1879.57 to 5276.9) | 48.68(27.84 to 79.96) | 1357.82(758.88 to 2921.71) | 15.61(8.8 to 33.38) | 678.31(459.01 to 1045.91) | 9.23(6.02 to 14.78) |
| Bahamas | 924.37(773.82 to 1103.53) | 348.97(291.35 to 417.23) | 498.79(385.47 to 640.85) | 213.9(159.24 to 281.02) | 84.82(64.89 to 108.59) | 31.7(24.19 to 40.62) | 43.4(33.77 to 55.48) | 17.65(13.21 to 23.59) | 104.89(76.47 to 134.53) | 39.74(28.95 to 51.01) | 61.34(43.84 to 85.56) | 29.41(20.65 to 41.45) | 57.27(45.37 to 74.37) | 22.44(17.94 to 28.98) | 93.78(73.94 to 116.96) | 28.18(22.12 to 35.89) |
| Bahrain | 3957.34(2929.95 to 5071.87) | 632.08(468.78 to 808.4) | 1477.13(1168.62 to 1830.23) | 148.94(116.92 to 186.71) | 595.58(429.9 to 783.76) | 95.69(69.3 to 125.53) | 243.64(195.91 to 302) | 23.43(18.47 to 29.33) | 400.07(215.2 to 631.66) | 63.3(34.22 to 99.61) | 172.96(107.84 to 326.6) | 18.72(11.48 to 36.05) | 185.09(129.4 to 254.44) | 30.04(21.16 to 41.19) | 240.87(184.47 to 322.42) | 22.28(16.87 to 29.51) |
| Bangladesh | 1817429.22(891746.36 to 3088202.26) | 958.36(467.08 to 1617.31) | 418333.73(207697.44 to 733858.66) | 291.23(142.49 to 516.44) | 397052.93(210732.42 to 627103.59) | 202.73(107.93 to 319.52) | 89685.72(41862.41 to 155219.3) | 64.69(29.61 to 112.88) | 212472.41(62881.14 to 483025.49) | 107.85(32.35 to 244.58) | 63706.07(27356.75 to 129004.36) | 46.65(19.85 to 94.57) | 48408.56(20538.06 to 119272.04) | 28.02(13 to 64.29) | 26182.33(14544.5 to 48676.42) | 17.84(9.81 to 33.26) |
| Barbados | 782.35(663.8 to 922.65) | 374.96(316.87 to 445.31) | 440.86(330.81 to 594.75) | 297.94(218.63 to 404.77) | 48.57(39.48 to 59.61) | 23.11(18.77 to 28.51) | 24.2(18.55 to 31.61) | 15.36(11.35 to 20.55) | 151.72(121.91 to 185.82) | 75.12(60.27 to 92.1) | 87.22(61.67 to 120.5) | 65.58(45.89 to 91.36) | 48.91(40.05 to 66.97) | 21.93(17.86 to 30.13) | 68.31(51.95 to 88.95) | 29.57(22.03 to 39.66) |
| Belarus | 43466.75(36558.49 to 51164.32) | 587.87(489.55 to 696.16) | 5915.15(4624.02 to 8597.7) | 100.89(76.55 to 159.17) | 11088.41(8951.16 to 13736.62) | 153.23(122.72 to 190.22) | 712.98(537.06 to 899.71) | 12.26(9.24 to 15.95) | 6290.56(4601.15 to 8017.9) | 89.37(65.19 to 114.02) | 1202.86(788.45 to 1755.02) | 27.37(17.19 to 40.71) | 3537.38(2287.49 to 4960.28) | 45.24(28 to 64.52) | 1804.44(1361.73 to 2359.45) | 29.54(21.85 to 39.97) |
| Belgium | 15430.2(12534.57 to 17059.29) | 234.48(188.44 to 259.25) | 6298.23(4981.1 to 7637.61) | 91.52(72.55 to 109.41) | 2420.25(2086.43 to 2840.86) | 37.11(31.79 to 43.83) | 887.37(697.61 to 1110.4) | 12.15(9.46 to 15.15) | 2431.72(2054.09 to 3131.77) | 39.05(33.02 to 50.54) | 1037.76(818.18 to 1345.17) | 16.92(13.15 to 22.48) | 1768.73(1524.7 to 2067.29) | 21.88(18.87 to 25.95) | 2992.53(2636.49 to 3369.51) | 31.23(26.36 to 36.52) |
| Belize | 1205.95(998.76 to 1471.14) | 411.67(342.46 to 499.87) | 806.74(662.59 to 989.7) | 210.31(172.14 to 259.47) | 215.51(176.1 to 274.03) | 73.04(59.93 to 92.42) | 107.46(83.44 to 137.7) | 27.76(21.38 to 35.88) | 321.18(233.17 to 403.11) | 107.29(77.97 to 134.56) | 265.68(208.8 to 340.28) | 71.21(55.69 to 91.35) | 102.97(74.98 to 132.21) | 37.98(28.77 to 47.51) | 129.71(108.16 to 155.78) | 31.91(26.49 to 38.86) |
| Benin | 88363.09(21627.02 to 142326.03) | 876.89(226.96 to 1399.67) | 120049.38(74816.62 to 177272.53) | 518.17(329.05 to 756.02) | 40105.74(16847.33 to 59856.94) | 384.68(164.03 to 577.59) | 45836.71(31297.62 to 63539.8) | 192.94(132.53 to 266.11) | 19405.01(6377.44 to 31364.79) | 182.47(60.1 to 296.68) | 32383.5(20676.89 to 47090.53) | 133.83(85.52 to 194.55) | 6976.6(1793.14 to 22772.95) | 76.02(22.15 to 233.63) | 13793.25(7232.39 to 28170.53) | 64.34(36.6 to 124.42) |
| Bermuda | 116.52(89.44 to 155.28) | 254.99(194.11 to 342.86) | 33.7(20.07 to 50.45) | 110.94(63.71 to 171.6) | 9.83(6.62 to 15.12) | 21.29(13.96 to 33.29) | 2.92(1.92 to 4.43) | 7.48(4.43 to 12.82) | 18.76(11.47 to 26.68) | 43(26.2 to 61.61) | 6.48(4.33 to 9.88) | 24.52(15.62 to 38.35) | 10.98(8.63 to 14.84) | 20.47(15.81 to 28.71) | 16.11(9.27 to 23.92) | 35.56(20.03 to 61.33) |
| Bhutan | 7558.25(3019.99 to 13413.05) | 759.27(301.03 to 1349.33) | 1961.28(994.68 to 3203.08) | 316.49(159.03 to 523.05) | 1538.02(622.86 to 2627.54) | 149.4(61.43 to 254.12) | 303.02(157.07 to 547.24) | 49.06(24.84 to 89.4) | 964.4(253.6 to 2272.96) | 93.31(24.96 to 218.97) | 320.83(135.91 to 639.01) | 53.01(22.33 to 106.13) | 190(89.18 to 423.94) | 20.96(10.55 to 43.84) | 127.34(70.01 to 229.53) | 19.98(10.79 to 36.48) |
| Bolivia (Plurinational State of) | 160717.65(66129.99 to 225250.93) | 1549.82(640.36 to 2164.73) | 75215.93(56176.41 to 98309.08) | 636.42(475.22 to 832.3) | 22501.95(11858.53 to 32616.7) | 211.99(111.48 to 306.57) | 9233.42(6382.19 to 13207.42) | 78.52(54.15 to 112.39) | 31476.99(11759.41 to 60895.91) | 295.75(110.42 to 571.01) | 19538.05(12664.22 to 27198.46) | 166.26(107.71 to 231.49) | 15008.72(4436.83 to 39537.56) | 146.34(43.95 to 382.86) | 8522.99(5296.68 to 12509.03) | 72.03(44.67 to 105.9) |
| Bosnia and Herzegovina | 8594.36(6072.01 to 11683.86) | 249.52(175.32 to 337.92) | 1843.78(1423.93 to 2398.39) | 115.73(88.91 to 153.18) | 1503.56(743.89 to 2600.72) | 44.54(21.41 to 78.34) | 274.31(193.93 to 373.51) | 15.93(10.46 to 22.45) | 1126.67(572.57 to 1800.67) | 34.05(17.13 to 54.77) | 246.98(152.23 to 483.31) | 17.15(10.33 to 34.34) | 662.82(460.02 to 1015.92) | 17.07(11.46 to 26.99) | 344.86(252.93 to 464.36) | 17.67(12.67 to 24.55) |
| Botswana | 4177.97(2969.23 to 5538.27) | 203.42(145.08 to 266.82) | 4082.16(2765.88 to 5788.68) | 172.75(116.52 to 245.5) | 1397.24(768.03 to 2238.35) | 66.86(37.15 to 105.99) | 1120.43(705.26 to 1649.5) | 47.38(29.73 to 70.02) | 826.1(397 to 1388.2) | 38.73(19 to 64.18) | 1009.58(512.3 to 1696.52) | 43.15(21.87 to 72.61) | 789.13(454.33 to 1222.41) | 39.02(23.08 to 59.47) | 1074.75(669.86 to 1641.43) | 45.67(28.35 to 69.92) |
| Brazil | 740490.74(632857.28 to 860107.57) | 471.97(403.25 to 548.98) | 456511.51(373657.67 to 547459.54) | 274.13(223.03 to 330.25) | 129867.25(109375.26 to 153072.36) | 83.46(70.26 to 98.39) | 75159.13(59028.86 to 97137.75) | 46(35.96 to 59.94) | 150310.71(124615.58 to 187808.4) | 96.56(80.08 to 120.69) | 123328.98(99220.45 to 153013.27) | 75.59(60.63 to 94.15) | 64007.2(50840.59 to 77630.69) | 40.63(32.2 to 49.33) | 57282.17(48767.12 to 70778.35) | 31.23(26.05 to 38.82) |
| Brunei Darussalam | 1211.88(955.83 to 1484.3) | 369.21(291.1 to 451.68) | 746.48(579.79 to 920.46) | 230.04(177.13 to 287.47) | 266.96(177.02 to 389) | 79.77(53.05 to 115.86) | 176.87(127.93 to 235.61) | 56.5(40.36 to 75.84) | 130.36(92.32 to 185.83) | 38.9(27.59 to 55.36) | 107.63(71.26 to 149.59) | 35.03(22.81 to 49.14) | 167.91(128.05 to 220.75) | 51.91(39.91 to 67.79) | 272.34(212.07 to 348.65) | 79.26(60.07 to 102.62) |
| Bulgaria | 32313.22(28468.59 to 36685.67) | 616.87(542.54 to 701.71) | 7258.13(6066.86 to 8635.12) | 218.72(180.14 to 262.84) | 4476.3(3704.56 to 5392.55) | 87.22(71.87 to 105.53) | 563.35(438.85 to 717.5) | 15.99(11.92 to 20.95) | 5227.78(4272.44 to 6811.99) | 104.98(85.42 to 137.26) | 965.37(742.73 to 1236.16) | 32.08(24.33 to 41.25) | 422.36(310.48 to 640.07) | 6.58(4.78 to 10.38) | 418.14(316.13 to 540.43) | 9.81(7.53 to 12.86) |
| Burkina Faso | 201125.15(39749.71 to 326194.1) | 1023.48(210.88 to 1641.48) | 296002.38(128135.74 to 455818.54) | 710.46(314.35 to 1080.86) | 75654.89(26894.8 to 121144.55) | 372.65(135.99 to 604.69) | 94688.78(55734.95 to 137791.94) | 222.06(131.78 to 322.55) | 44285.09(11878.41 to 76369.45) | 213.4(56.93 to 371.37) | 76116.84(40731.55 to 115689.37) | 174.49(94.51 to 265.06) | 15225.92(2921.31 to 49074) | 85.3(18.75 to 260.47) | 33932.3(14331.9 to 89514.76) | 88.03(39.16 to 221.24) |
| Burundi | 77999.98(21752.63 to 135048.04) | 697.15(202.57 to 1197.62) | 69466.98(41297.28 to 111444.78) | 326.75(197.69 to 523.41) | 49558.11(26832.83 to 72595.77) | 428.07(231.43 to 624.39) | 45531.81(25552.93 to 71714.74) | 208.9(117.18 to 327.64) | 18379.13(3677.76 to 38392.1) | 156.33(32.4 to 326.45) | 17231.61(8441.81 to 28579.95) | 78.58(39.37 to 129.87) | 8835.07(1758.1 to 30876.26) | 83.4(18.96 to 282.49) | 11137.84(5477.72 to 24163.31) | 54.91(28.74 to 115.86) |
| Cabo Verde | 3114.4(1250.89 to 4443.67) | 542.06(224.4 to 767.06) | 800.9(508.46 to 1175.22) | 172.97(107.95 to 257.82) | 912.08(563.25 to 1517.55) | 156.9(98.88 to 259.52) | 208.39(129.85 to 341.9) | 46.46(28.38 to 76.76) | 703.08(378.78 to 1055.99) | 118.5(64.61 to 178.01) | 186.99(68.15 to 371.48) | 44.15(15.86 to 88.24) | 196.46(101.53 to 390.9) | 37.21(21.09 to 68.85) | 128.3(64.77 to 201.66) | 27.05(13.47 to 43.17) |
| Cambodia | 293982.84(83159.13 to 436132.89) | 1560.28(461.9 to 2301.55) | 123669.63(86524.8 to 166781.26) | 713.9(499.85 to 963.1) | 45685.8(18818.81 to 70957.77) | 228.88(94.93 to 354.69) | 18355.58(10985.4 to 31512.42) | 106.89(63.87 to 183.63) | 30064.99(7723.83 to 65976.65) | 153.6(39.3 to 337.14) | 17023.57(8439.69 to 28344.93) | 99.06(49.07 to 164.96) | 6868.13(1745.45 to 22444.07) | 38.36(10.49 to 122.06) | 3150.57(1876.57 to 4916.23) | 18.05(10.69 to 28.22) |
| Cameroon | 140281.57(43825.91 to 215768.25) | 694.27(230.54 to 1059.91) | 205412.09(122328.5 to 283572.61) | 436.15(263.64 to 597.75) | 57101.61(29670.63 to 82627.23) | 272.28(144.07 to 391.33) | 77041.26(47428.62 to 105965.96) | 160.42(100 to 220.12) | 37746.23(12819.31 to 61010.98) | 175.45(60.19 to 282.93) | 66858.3(40381.77 to 98624.04) | 137.64(83.33 to 203.18) | 11022.2(4182.75 to 29537.64) | 61.1(24.62 to 151.94) | 26458.29(15697.62 to 50110.83) | 61.18(38.35 to 111.47) |
| Canada | 52703.14(43585.52 to 57255.97) | 254.08(209.36 to 277.02) | 21150.36(18169.6 to 24819.12) | 92.67(78.73 to 109.51) | 8270.61(7050.24 to 10344.87) | 40.71(34.64 to 51.44) | 3365.3(2738.83 to 4003.96) | 14.96(11.76 to 18.21) | 6812.96(5931.17 to 8549.47) | 34.34(29.84 to 43.18) | 3651.78(2809.85 to 4531.17) | 18.75(14.27 to 23.52) | 5935.44(4874.81 to 7154.85) | 24.42(20.2 to 29.41) | 10943.99(9749.79 to 12128.37) | 31.74(27.76 to 36.11) |
| Central African Republic | 42200.61(12556.99 to 80180.78) | 777.98(234.21 to 1461.55) | 45821.16(20797.54 to 78714.4) | 537.55(248.53 to 918.28) | 27737.19(14227.08 to 42727.3) | 491.75(252.12 to 757.24) | 28634.58(15009.19 to 42281.32) | 327.55(171.98 to 482.71) | 9203.63(1998.18 to 19941.94) | 162.06(36.17 to 350.96) | 9872.99(3621.5 to 17259.1) | 112.37(41.85 to 195.37) | 4147.43(922.22 to 14472.16) | 79.79(19.5 to 277.27) | 6350.68(2310.52 to 17984.01) | 76.81(28.5 to 214.5) |
| Chad | 92793.75(20446.44 to 146136.25) | 713.6(168.57 to 1125.38) | 217535.31(79064.91 to 331187.97) | 601(229.56 to 908.37) | 38059.47(15767.48 to 62953.42) | 280.39(118.08 to 466.27) | 75307.42(42692.94 to 113731.95) | 200.27(115.69 to 300.18) | 17488.62(5312.59 to 29266.56) | 124.34(37.96 to 208.94) | 44548.72(21782.11 to 69592.16) | 114.76(56.05 to 178.69) | 6025.1(1411.92 to 20268.52) | 51.93(13.9 to 160.82) | 19271.34(7261.19 to 52867.97) | 60.05(24.73 to 151.01) |
| Chile | 41581.69(36923.08 to 47104.62) | 285.5(253.92 to 323.34) | 14900.8(12726.71 to 17229.89) | 128.86(109.65 to 150.12) | 17368.06(14226.77 to 20726.66) | 118.88(97.44 to 141.88) | 4340.28(3431.98 to 5402.49) | 38.08(29.38 to 47.58) | 11886.8(10065.64 to 13857.21) | 81.16(68.75 to 94.57) | 4118.69(3356.26 to 4964.16) | 39.57(32.05 to 48.35) | 8269.45(6299.41 to 9797.76) | 57.41(43.87 to 67.86) | 4783.82(4012.8 to 5608.37) | 33.91(27.95 to 40.35) |
| China | 10962853.36(6949618.27 to 15100267.73) | 978.02(617.52 to 1348.03) | 1719717.27(1389266.32 to 2114114.28) | 241.68(191.38 to 304.84) | 1492541.85(1081129.64 to 1887283.03) | 134.59(97.47 to 170.25) | 105488.86(76232.81 to 136159.4) | 16.83(11.66 to 22.32) | 1281060.14(688871.8 to 2322525.41) | 115.78(62.16 to 210.08) | 234588.94(151523.04 to 342194.48) | 40.4(25.76 to 59.4) | 654145.15(436029.99 to 1028444.24) | 58.59(38.82 to 92.38) | 149514.6(89979.83 to 203242.76) | 23.3(13.27 to 32.03) |
| Colombia | 204331.45(171876.14 to 233302.71) | 476.8(402.24 to 543.28) | 106854.13(79605.33 to 145074.42) | 305.52(223.84 to 419.42) | 26308.86(21573.82 to 30993.9) | 61.03(50.16 to 71.78) | 6644.16(4918.78 to 9062.96) | 18(13.02 to 24.94) | 41412.31(34494.21 to 50828.98) | 94.85(79.1 to 116.41) | 25724.34(17779.21 to 36550.13) | 77(53.1 to 109.86) | 7767.71(6401.44 to 9021.61) | 18.73(15.44 to 21.74) | 7499.87(5702.06 to 9771.85) | 19.47(14.39 to 25.7) |
| Comoros | 5367.64(1783.31 to 10415.01) | 613.57(211.91 to 1183.96) | 2504.14(1504.71 to 4495.9) | 307.9(185.08 to 552.85) | 3509.93(2051.66 to 5420.69) | 389.9(229.26 to 598.78) | 1662.35(982.81 to 2488.4) | 204.06(120.58 to 305.36) | 1378.11(345.78 to 2775.89) | 152.48(39.82 to 305.18) | 785.04(401.61 to 1415.71) | 96.64(49.55 to 174.24) | 759.17(163.48 to 2819.95) | 90.26(21.73 to 332.68) | 560.58(270.35 to 1272.94) | 69.36(33.42 to 157.05) |
| Congo | 18295.53(7178.14 to 34018.34) | 452.3(182.16 to 836.93) | 13220.97(8864.57 to 20181.91) | 215.16(144.43 to 327.74) | 10874.31(5909.77 to 16525.53) | 260.66(142.31 to 396.45) | 7014.71(4169.42 to 10079.62) | 114.02(67.83 to 163.95) | 4915.69(1705.72 to 8778.89) | 116.82(41.11 to 208.13) | 4146.99(2547.84 to 7118.64) | 67.68(41.71 to 116.38) | 2402.72(869.04 to 7127.19) | 61.73(23.28 to 179.47) | 2838.06(1644.6 to 5091.48) | 46.94(27.31 to 83.59) |
| Cook Islands | 43.17(27.37 to 61.3) | 202.27(128.8 to 287.39) | 10.73(6.18 to 21.01) | 84.15(46.65 to 171.4) | 6.26(4 to 9.57) | 29.37(18.72 to 44.91) | 0.82(0.38 to 1.55) | 6.76(2.79 to 13.3) | 2.43(1.33 to 7.17) | 11.48(6.28 to 33.72) | 0.89(0.41 to 2.63) | 7.28(2.95 to 23.73) | 5.4(2.95 to 8.29) | 25.23(13.82 to 38.72) | 2.38(0.52 to 5.08) | 19.74(3.65 to 43.81) |
| Costa Rica | 15097.82(13720.73 to 16641.55) | 387.91(352.83 to 427.82) | 8204.19(6918.43 to 9637.08) | 259.58(212.35 to 310.45) | 4841.51(4002.54 to 5616.02) | 124.3(102.83 to 144.15) | 1324.12(1097.95 to 1611.16) | 43.82(35.84 to 54.15) | 3929.7(3277.32 to 4673.47) | 100.24(83.63 to 119.3) | 2053.33(1651.5 to 2555.66) | 73.4(58.3 to 91.93) | 1316.05(1089.13 to 1541.93) | 34.67(28.75 to 40.48) | 1392.05(1197.47 to 1630.05) | 37.87(31.69 to 45.47) |
| Côte d'Ivoire | 163466.52(49661.14 to 260539.1) | 690.87(222.97 to 1084.23) | 189989.16(122728.41 to 269544.8) | 447.23(294.71 to 626.29) | 74950.49(35163.37 to 108311.04) | 304.16(145.64 to 435.92) | 80351.58(57295.79 to 110809.49) | 184.23(131.79 to 254.15) | 40336.41(14090.74 to 61956.06) | 160.61(57.36 to 246.38) | 54254.54(35641.82 to 78768.95) | 122.72(80.61 to 177.66) | 12707.47(4489.09 to 33258.3) | 59.88(24.03 to 144.86) | 27883.88(15563.02 to 54945.93) | 71.11(41.81 to 132.59) |
| Croatia | 10501.37(8642.94 to 11500.35) | 354.55(288.47 to 388.79) | 2118.04(1721.79 to 2623.26) | 104.37(83.05 to 131.17) | 733.55(609.1 to 892.35) | 24.16(19.94 to 29.39) | 208.78(162.02 to 270.09) | 7.6(5.94 to 9.78) | 2043.97(1659.92 to 2446.23) | 73.92(59.82 to 88.84) | 466.71(354.21 to 599.76) | 26.02(19.41 to 33.71) | 930.31(775.42 to 1160.36) | 26.87(22.48 to 33.53) | 696.27(563.26 to 855.03) | 28.06(22.28 to 34.9) |
| Cuba | 46985.25(42043.32 to 50885.18) | 508.18(453.72 to 552.91) | 9657.16(8041.29 to 12376.43) | 148.2(121.94 to 192.23) | 2240.96(1931.61 to 2648.6) | 24.11(20.66 to 28.54) | 638.91(532.07 to 772.1) | 8.92(7.52 to 10.74) | 13396.57(9348.35 to 16256.76) | 152.93(106.71 to 185.74) | 2685.89(2215.46 to 3382.31) | 50.02(41.02 to 62.93) | 2978.61(2476.46 to 3568.75) | 29.2(24.22 to 34.84) | 2930.59(2407.29 to 3623.7) | 27.96(23.14 to 34.23) |
| Cyprus | 1903.1(1092.52 to 2604.15) | 282.07(158.94 to 387.21) | 552.62(410.88 to 714.45) | 60.57(46.16 to 77.77) | 199.67(106.19 to 322.98) | 29.4(15.36 to 48.03) | 86.45(65.49 to 113.36) | 8.84(6.63 to 11.62) | 335.85(156.01 to 559.11) | 50.47(23.32 to 84.21) | 95.31(61.76 to 157.43) | 11.74(7.37 to 20.02) | 251.13(178.82 to 362.87) | 35.31(24.61 to 51.86) | 304.06(241.77 to 376.43) | 26.66(20.78 to 34.12) |
| Czechia | 19835.3(15385.05 to 21997.74) | 295.22(226.27 to 326.51) | 3560.49(2805.95 to 4444) | 55.24(42.36 to 68.52) | 3650.29(3003.65 to 4364.3) | 55.64(45.37 to 66.8) | 530.52(401.05 to 682.5) | 7.21(5.19 to 9.42) | 4538.85(3466.2 to 5480.86) | 71.64(54.47 to 86.69) | 573.4(396.96 to 785.64) | 10.19(6.92 to 14.22) | 1552.89(1298.42 to 1826.46) | 20.5(17.16 to 24.09) | 1168.72(882.87 to 1442.03) | 15.49(11.58 to 19.59) |
| Democratic People's Republic of Korea | 165952.87(117023.02 to 225813.7) | 673.82(476.69 to 916.49) | 49664.97(33670.16 to 72912.08) | 291.98(195.53 to 438.23) | 16921.08(10535.42 to 26215.12) | 66.81(41.8 to 103.4) | 3621.59(1870.07 to 6623.78) | 24.03(12.03 to 44.75) | 23691.17(13376.36 to 37466.34) | 93.19(52.83 to 147.08) | 6271.18(3510.53 to 10172) | 42.42(23.42 to 69.4) | 6389.37(3778.07 to 9385.74) | 25.89(15.53 to 37.76) | 2545.74(1255.77 to 4088.87) | 16.15(7.57 to 26.58) |
| Democratic Republic of the Congo | 475484.76(155606.79 to 843667) | 609.38(205.86 to 1084.04) | 351219.77(214416.75 to 575120.29) | 267.42(162.87 to 439.7) | 329181.8(187603.4 to 470061.37) | 407.34(230.77 to 575.18) | 228516.97(130236.5 to 344821.2) | 170.62(98.25 to 256.57) | 117126.61(30204.79 to 217880.23) | 143.7(37.42 to 267.09) | 87899.44(48609.36 to 158893.4) | 65.22(36.51 to 117.4) | 55914.79(15591.13 to 178962.23) | 74.22(22.13 to 233.19) | 63666.83(30897.05 to 140028.94) | 50.05(24.59 to 107.46) |
| Denmark | 13636.06(11538.05 to 15155.53) | 406.43(339.89 to 453.02) | 3631.97(2892.52 to 4404.84) | 95.06(74.11 to 114.63) | 1745.67(1458.49 to 2078.06) | 52.54(43.84 to 63.19) | 625.92(515.46 to 751.27) | 16.51(13.44 to 19.81) | 2553.67(1711.75 to 3245.97) | 81.54(54.32 to 104) | 774.64(613.92 to 958.58) | 23.47(18.26 to 29.24) | 1528.92(1339.32 to 1763.93) | 36.9(32.27 to 42.99) | 1723.78(1477.57 to 2018.75) | 32.82(26.86 to 39.46) |
| Djibouti | 3101.74(1029.61 to 6271.46) | 450.73(153.79 to 903.72) | 3474.62(2024.49 to 6278.08) | 241.18(140.71 to 435.61) | 1646.42(910.68 to 2633.7) | 233.13(129.44 to 378.73) | 1585.45(895.93 to 2541.28) | 109.73(62.12 to 175.7) | 774.98(273.23 to 1488.17) | 108.94(38.83 to 209) | 977.66(499.49 to 1912.85) | 67.64(34.57 to 132.31) | 425.85(124.58 to 1430.73) | 64.06(20.01 to 213.8) | 832.71(368.96 to 1850.1) | 58.29(26 to 128.81) |
| Dominica | 245.74(192.41 to 312.14) | 280.85(220.98 to 356.04) | 152.74(102.59 to 211.24) | 436.03(288.9 to 615.06) | 28.22(19.85 to 39.3) | 32.11(22.69 to 44.56) | 19(13.17 to 27.39) | 53.53(35.41 to 79.62) | 43.36(27.37 to 64.13) | 48.75(30.9 to 71.97) | 31.35(19.45 to 45.98) | 96.56(59.11 to 143.13) | 14.45(10.51 to 18.91) | 18.46(13.42 to 23.78) | 21.18(15.53 to 27.67) | 47.69(33.64 to 64.99) |
| Dominican Republic | 54907.58(43110.44 to 76798.92) | 530.2(418.71 to 737.81) | 23305.56(13791.13 to 42841.12) | 224.69(132.1 to 414.48) | 8799.53(5998.65 to 12676.9) | 84.04(57.56 to 120.55) | 2905.94(1796.98 to 5125.2) | 28.01(17.22 to 49.7) | 18501.65(9762.72 to 28549.73) | 174.71(92.29 to 270.51) | 8965.52(4871.71 to 17062.21) | 87.22(47.33 to 166.17) | 5646.58(3268.36 to 12127.5) | 57.61(34.48 to 120.59) | 3494.49(2345.22 to 5753.07) | 32.78(21.62 to 54.62) |
| Ecuador | 62961.66(52672.98 to 71905.66) | 458.17(385.01 to 522.62) | 58407.45(45792.09 to 73926.44) | 362.47(282.28 to 462.07) | 5830.23(4125.76 to 7551.28) | 41.91(29.84 to 53.94) | 4746.68(3659.71 to 6022.35) | 29.68(22.75 to 37.9) | 11484.64(9379.07 to 14915.85) | 81.29(66.41 to 105.58) | 15237.66(11116.53 to 20334.13) | 97.2(70.77 to 129.8) | 9621.21(7215.75 to 12008.54) | 70.66(53.18 to 88.03) | 5885.16(4410.42 to 8159.48) | 36.1(26.78 to 50.63) |
| Egypt | 1467910.9(511758.98 to 2213628.85) | 1683.58(588.88 to 2533.21) | 492900.1(370101.54 to 650820.47) | 394.34(296.59 to 520.64) | 273762.86(98496.33 to 436891.72) | 317.13(115.38 to 504.88) | 56228.08(38503.3 to 78859.07) | 45.25(31.08 to 63.57) | 162895.11(41536.48 to 341074.91) | 183.97(47.09 to 384.79) | 67592.74(46626.21 to 97321.42) | 54.01(37.3 to 77.87) | 77753.91(22148.55 to 213197.19) | 91.15(26.71 to 247.24) | 36654.52(25294.78 to 49562.98) | 29.54(20.45 to 39.88) |
| El Salvador | 73362.9(40259.21 to 97158.11) | 914.68(507.33 to 1208.84) | 17711.77(11979.84 to 25368.82) | 301.36(202.6 to 433.25) | 20797.19(14206.99 to 28620.64) | 254.62(174.24 to 350.08) | 3389.01(2068.06 to 5079.43) | 58.4(35.23 to 88.2) | 5174.07(2466.77 to 8114.19) | 63.35(30.35 to 99.46) | 1561(727.07 to 3311.52) | 27.27(12.61 to 58.06) | 1570.81(845.22 to 3014.68) | 20.64(11.31 to 38.75) | 888.75(590.29 to 1188.44) | 14.43(9.45 to 19.46) |
| Equatorial Guinea | 4685.17(1681.35 to 9097.58) | 538.4(200.7 to 1035.34) | 3860.19(2312.02 to 6098.34) | 213.52(128 to 337.84) | 3056.28(1609.77 to 4631.34) | 335.33(176.61 to 500.59) | 2012.43(1068.96 to 3371.64) | 111.04(58.94 to 186.62) | 1028.62(273.8 to 2096.19) | 111.49(30.26 to 225.51) | 1453.88(788.22 to 2354.46) | 80.83(43.98 to 131.11) | 505.4(130.98 to 1659.1) | 60.81(16.9 to 196.14) | 628.91(329.82 to 1150.14) | 35.43(18.64 to 64.53) |
| Eritrea | 34605.2(9484.23 to 69840.73) | 539.96(153.15 to 1082.65) | 31218.61(17701.01 to 59962.35) | 345.11(197.52 to 662.35) | 20045.11(11137.89 to 32835.42) | 303.79(168.41 to 497.78) | 16602.97(9637.02 to 25833.58) | 181.3(105.51 to 281.42) | 7120.17(1730.93 to 14604.73) | 106.07(26.4 to 216.68) | 7324.82(3822.18 to 14544.52) | 79.49(41.99 to 157.24) | 4734.57(751.55 to 17731.5) | 77.76(13.69 to 285.6) | 5625.97(2559.27 to 12799.72) | 63.94(29.84 to 144.19) |
| Estonia | 4386.97(3894.9 to 4872.29) | 383.82(340.21 to 427.08) | 515.21(395.54 to 657.69) | 63.48(46.75 to 82.69) | 1100.28(935.8 to 1294.11) | 98.22(82.96 to 115.71) | 67(48.94 to 86.43) | 7.69(5.46 to 10.08) | 517.06(385.55 to 627.66) | 47.88(35.5 to 58.27) | 50.78(35.07 to 90.71) | 6.91(4.51 to 12.82) | 594.46(444.51 to 721.37) | 50.12(37.32 to 61.2) | 104.4(80.43 to 130.11) | 12.24(9.19 to 15.36) |
| Eswatini | 3491.81(2208.2 to 4783.65) | 247.14(157.38 to 335.33) | 2816.85(1970.62 to 3995.12) | 206.63(144.98 to 292.81) | 1066.3(645.63 to 1586.78) | 74.29(45.54 to 109.04) | 675.11(384.56 to 1097.69) | 49.48(28.31 to 79.82) | 767.03(471.82 to 1087.39) | 52(32.19 to 73.46) | 739.02(403.23 to 1258.13) | 53.7(29.37 to 91.06) | 631.2(301.88 to 1008.07) | 45.5(23.36 to 71.24) | 703.71(415.51 to 1070.39) | 52.11(30.96 to 78.88) |
| Ethiopia | 783965.32(201221.95 to 1602138.01) | 762.07(204.21 to 1553.26) | 500623.21(311491.43 to 892453.78) | 312.37(196.07 to 555.16) | 433451.59(218732.89 to 697829.2) | 406.57(206.88 to 663.4) | 256652.64(162940.3 to 392948.79) | 157.37(100.84 to 240.44) | 163023.07(35941.71 to 356912.85) | 150.87(34.35 to 329.56) | 128310.71(71134.96 to 221601.59) | 78.37(44.34 to 134.63) | 78764.77(13454.82 to 295349.12) | 81.28(16.02 to 295.35) | 70852.83(36407.34 to 157898.27) | 45.65(24.15 to 99.94) |
| Fiji | 5362.72(4177.88 to 6652.49) | 589.11(459.38 to 729.93) | 4755.73(3451.85 to 6331.82) | 529.11(382.88 to 706.36) | 313.3(202.64 to 466.7) | 34.47(22.3 to 51.4) | 307.49(199.94 to 434.26) | 34.94(22.67 to 49.47) | 233.77(134.7 to 413.51) | 25.86(14.91 to 45.61) | 296.43(157.3 to 496.46) | 33.69(17.83 to 56.55) | 439.1(301.42 to 604.71) | 49.44(34.2 to 68.17) | 801.53(527.61 to 1149.9) | 88.39(58.03 to 127.35) |
| Finland | 10335.79(9139.71 to 11540.05) | 292.47(258.33 to 325.44) | 2913.49(2313.95 to 3547.08) | 89.5(70.08 to 109.64) | 842.31(704.32 to 1003.17) | 24.26(20.03 to 29.15) | 331.25(265.73 to 411.57) | 9.56(7.64 to 11.7) | 1731.64(1357.47 to 2102.43) | 52.82(41.2 to 64.31) | 472.01(378.19 to 615.65) | 17.13(13.5 to 22.89) | 1527.69(1328.23 to 1802.71) | 35.35(30.66 to 41.88) | 2131.92(1928.95 to 2341.75) | 43.6(38.29 to 49) |
| France | 106056.13(91024.5 to 115044.81) | 262.35(221.13 to 281.94) | 36542.33(28833.07 to 45407.48) | 84.23(66.89 to 106.83) | 8928.75(7840.88 to 10325.78) | 22.13(19.35 to 25.8) | 3971.37(3187.41 to 4918.05) | 9.08(7.18 to 11.01) | 17095.74(14530.31 to 21265.95) | 44.71(37.85 to 55.74) | 6994.3(5518.66 to 8725.54) | 19.06(14.85 to 23.91) | 9518.97(8078.57 to 11143.47) | 19.5(16.7 to 22.9) | 17217.96(15053.47 to 19255.93) | 28.51(24.42 to 32.54) |
| Gabon | 5677.52(2928.04 to 10517.43) | 357.42(189.27 to 656.1) | 3889.85(2520.95 to 6460) | 187.57(121.28 to 311.44) | 3427.88(2156.42 to 5213.75) | 208.96(132.59 to 316.47) | 1958.12(1085.36 to 3209.62) | 94.33(52.32 to 154.4) | 1619.81(608.3 to 2968.28) | 97.96(37.6 to 179.22) | 1287.04(706.9 to 2124.13) | 62.15(34.14 to 102.68) | 599.38(294.24 to 1416.93) | 39.19(19.73 to 91.4) | 733.58(419.3 to 1264.71) | 35.86(20.61 to 62.17) |
| Gambia | 10152.95(3278.83 to 15118.7) | 542.47(184.91 to 800.1) | 10746.58(7360.84 to 14924.62) | 312.51(216.45 to 432.46) | 3614.2(1716.94 to 5529.31) | 185.01(91.07 to 284.25) | 3434.84(2392.36 to 5032.27) | 97.27(68.49 to 141.27) | 2163.74(960.75 to 3269.17) | 107.38(47.87 to 163.5) | 2606.32(1539 to 4476.87) | 72.61(42.9 to 124.68) | 728.96(266.61 to 2016.24) | 44.5(18.51 to 113.79) | 1759.44(953.84 to 3336.67) | 57.4(32.05 to 104.11) |
| Georgia | 11957.32(9196.37 to 14461.97) | 272.38(207.69 to 331.11) | 5646.98(4383.59 to 7031.99) | 238.31(181.92 to 299.56) | 3020.9(2137.22 to 3885.8) | 70.39(49.42 to 90.56) | 351.51(267.15 to 458.1) | 13.84(10.3 to 18.33) | 2124.25(1344.96 to 2973.42) | 49.88(31.5 to 70.09) | 1022.28(736.92 to 1369.83) | 45.43(32.34 to 60.78) | 538.15(331.72 to 791.3) | 11.99(7.17 to 17.88) | 259.88(200.06 to 354.17) | 10.48(7.96 to 14.12) |
| Germany | 130186.35(111865.01 to 149404.36) | 271.18(230.32 to 313.19) | 47363.89(38820.7 to 57422.79) | 95.94(79.59 to 113.49) | 13896.87(11605.12 to 17045.08) | 28.22(23.7 to 34.93) | 8266.32(6726.18 to 10155.97) | 16.2(13.08 to 19.83) | 17804.28(14411.23 to 25957.15) | 39.61(31.95 to 58.19) | 7663.56(6483.21 to 9317.16) | 18.03(15.11 to 22.28) | 15102.87(12708.37 to 18146.27) | 24.98(20.94 to 29.78) | 26251.94(23950.65 to 28736.71) | 34.18(30.1 to 38.44) |
| Ghana | 136941.17(47706.39 to 208931.28) | 526.95(193.54 to 795.01) | 130094.33(84754.09 to 176372.27) | 291.82(191.48 to 391.88) | 61777.29(29009.21 to 86484.53) | 228.34(109.41 to 320.66) | 51457.81(31509.82 to 74489.1) | 113.42(70.41 to 163.48) | 37048.25(14586.75 to 56501.05) | 134(53.47 to 203.52) | 41755.28(25563.95 to 71399.75) | 91.32(56 to 156.07) | 13548.97(4888.2 to 35522.62) | 58.31(22.08 to 142.42) | 23190.78(15082.9 to 36667.15) | 56.44(37.79 to 87.29) |
| Greece | 28450.05(24234.98 to 31218.06) | 500.94(422.67 to 555.44) | 6756.32(5596.02 to 8062.79) | 128.14(104.57 to 151.92) | 3970.04(3433.93 to 5083.04) | 69.8(59.8 to 91.15) | 1105.57(894.57 to 1336.63) | 20.19(15.99 to 24.4) | 2256.36(1847.23 to 3320.84) | 42.04(34.37 to 62.84) | 834.58(656.74 to 1100.63) | 18.25(14.04 to 24.81) | 2744.97(2289.94 to 3369.86) | 39.82(32.68 to 50.74) | 3199.74(2827.9 to 3672.87) | 40.09(34.52 to 47.68) |
| Greenland | 217.61(124.07 to 312.66) | 380.87(215.1 to 547.95) | 41.51(29.89 to 64.25) | 97.77(68.97 to 155.98) | 54.11(27.82 to 85.32) | 93.72(48.18 to 148.14) | 10.56(6.09 to 17.58) | 26.06(14.76 to 44.65) | 47.29(23.46 to 79.71) | 82.33(40.87 to 138.58) | 12.23(7.51 to 20.18) | 31.11(18.98 to 52.3) | 13.35(8.47 to 22.46) | 23.38(14.76 to 39.16) | 11.54(8.2 to 16.34) | 21.35(14.4 to 31.66) |
| Grenada | 514.31(425.93 to 624.48) | 454.42(378.72 to 549.41) | 243.67(200.47 to 294.04) | 337.87(274.27 to 414.9) | 47.01(34.99 to 64.74) | 41.48(31.02 to 56.88) | 21.22(16.81 to 26.51) | 29.27(22.97 to 37.14) | 43.74(29.8 to 68.61) | 37.83(25.79 to 59.51) | 20.86(15.18 to 28.29) | 30.71(22.11 to 42.18) | 27.6(20.69 to 35.54) | 27.96(21.62 to 35.39) | 22.12(18.69 to 26.66) | 23.67(19.75 to 28.99) |
| Guam | 359.56(289 to 458.38) | 211.25(170.64 to 268.45) | 230.84(177.31 to 308.55) | 174.58(133.09 to 233.89) | 43.53(31.1 to 60.66) | 24.63(17.71 to 34.17) | 40.74(27.02 to 58.44) | 31.61(20.83 to 45.54) | 19.92(8.25 to 59.82) | 11.37(4.86 to 33.39) | 13.15(6.57 to 49.02) | 10.01(4.9 to 38) | 44.13(19.82 to 68.02) | 26.59(12.08 to 41.08) | 61.17(27.13 to 96.49) | 46.44(20.32 to 73.74) |
| Guatemala | 26052.78(21842.37 to 32838.29) | 170.59(143.87 to 215.15) | 53426.88(39131.03 to 69182.34) | 362.32(264.97 to 470.86) | 17876.16(13095.33 to 23542.31) | 111.53(82.35 to 146.31) | 9468.52(6892.21 to 12719.11) | 64.21(46.4 to 86.58) | 7043.22(5333.09 to 10469.21) | 43.96(33.55 to 64.97) | 12953.8(9437.42 to 18838.11) | 89.42(65.07 to 130.14) | 4854.4(3452.35 to 6199.07) | 34.18(24.85 to 42.94) | 7826.05(5255.68 to 11195.87) | 51.64(34.3 to 74.84) |
| Guinea | 135951.19(29175.29 to 232718.34) | 1110.66(247.28 to 1896.72) | 133424.34(78156.88 to 193139.23) | 600.33(355.68 to 860.87) | 61516.06(26285.9 to 93220.35) | 484.6(205.63 to 742.59) | 51889.65(34552.63 to 72430.9) | 227.76(153.35 to 317.87) | 30194.5(7521.94 to 52215.36) | 233.21(57.84 to 410.79) | 32224.04(19639.56 to 48369.81) | 139.52(84.83 to 208.17) | 10633.12(2193.5 to 36948.32) | 93.87(21.47 to 309.11) | 15097.76(7662.44 to 33252.46) | 74.23(39.83 to 155.04) |
| Guinea-Bissau | 18482.99(3955.26 to 29908.4) | 969.89(220.28 to 1558.66) | 13452.83(8473.05 to 18562.82) | 427.01(270.75 to 591.46) | 8813.76(3239.91 to 12917.42) | 441.8(166.59 to 642.47) | 6096.68(3806 to 9093.43) | 186.13(117.29 to 276.07) | 3698.19(1125.44 to 6044.17) | 181.12(55.32 to 294.92) | 3089.53(1912.54 to 5150.9) | 92.85(57.68 to 154.65) | 1634.06(357.03 to 5487.4) | 97.5(23.03 to 303.01) | 2176.75(1017.67 to 4592.97) | 79(38.24 to 157.54) |
| Guyana | 4742.62(3914.95 to 5698.45) | 402.98(333.15 to 482.5) | 2420.3(1760.7 to 3238.88) | 329.57(239.39 to 441.46) | 859.93(659.8 to 1107.67) | 71.32(55.03 to 91.32) | 228.3(168.53 to 308.23) | 31.18(22.94 to 42.2) | 1377.44(965.19 to 1716.33) | 112.12(78.97 to 139.18) | 562.51(376.14 to 795.94) | 77.78(51.91 to 110.17) | 78.77(60.21 to 97.54) | 8.05(6.26 to 9.84) | 70.87(50.95 to 96.89) | 9.27(6.64 to 12.73) |
| Haiti | 208824.04(132573.93 to 291351.65) | 1889.82(1184.75 to 2621.42) | 174082.65(95240.94 to 304063.77) | 1093.68(595.59 to 1943.45) | 34960.5(19996.43 to 57015.84) | 312.89(178.69 to 518.86) | 24493.62(13755.34 to 44285.95) | 153.24(85.87 to 278.76) | 28274.49(9631.3 to 66205.52) | 245.76(82.63 to 575.81) | 27335.76(11820.82 to 51164.9) | 169.4(72.99 to 316.74) | 12518.78(2979.34 to 40065.92) | 126.71(33.62 to 377.05) | 14660.18(5061.71 to 33342.71) | 97.65(34.16 to 219.54) |
| Honduras | 62271.53(36815.67 to 87066.51) | 764.9(462.03 to 1064.7) | 30908.89(21723.69 to 44752.47) | 288.13(203.22 to 416.74) | 14996.86(10249.83 to 21274.68) | 179.87(123.68 to 253.15) | 5673.33(3398.15 to 8767.92) | 52.7(31.46 to 81.54) | 11325.97(6893.15 to 16833.21) | 134.37(81.92 to 199.28) | 7408.32(4540.87 to 11255.52) | 69.34(42.51 to 105.33) | 3412.76(1880.42 to 6228.32) | 44.61(25.46 to 80.35) | 2818.52(1861.26 to 4150.54) | 26.55(17.46 to 38.87) |
| Hungary | 27728.05(23239.17 to 31455.54) | 422.11(351.51 to 480.62) | 5818.92(4658.57 to 7026.01) | 108.79(84.3 to 132.51) | 4006.91(3272.89 to 4854.33) | 63.15(51.18 to 76.74) | 560.01(428.82 to 723.13) | 9.43(7.04 to 12.5) | 5768.22(4517.23 to 6707.39) | 93.81(73.19 to 109.2) | 805.45(584.21 to 1138.59) | 17.48(12.5 to 24.87) | 2625.19(2274.13 to 2993.94) | 35.97(30.66 to 41.6) | 2158.61(1685.92 to 2699.25) | 32.08(24.03 to 41.24) |
| Iceland | 532.26(446.93 to 602.33) | 231.83(194.21 to 262.6) | 210.67(166.35 to 257.59) | 80.87(62.93 to 99.3) | 35.32(29.26 to 41.89) | 15.37(12.69 to 18.22) | 20.74(16.21 to 26.51) | 7.55(6.01 to 9.61) | 94.03(67.51 to 118.56) | 41.8(29.99 to 52.75) | 36.45(28.5 to 48.91) | 15.07(11.55 to 20.36) | 70.34(61.01 to 82.04) | 29.35(25.49 to 34.3) | 126.49(106.73 to 147.02) | 41.48(33.77 to 49.16) |
| India | 7187715.83(4358211.6 to 9657873.63) | 621.08(377.27 to 832.52) | 3557478.65(2666545.8 to 4881692) | 321.95(239.51 to 447.86) | 1489482.64(1093889.94 to 1984352.55) | 126.56(93.18 to 168.38) | 580948.48(405631.75 to 860754.76) | 53.45(36.86 to 79.86) | 1039632.88(492383.15 to 1838271.08) | 88.1(41.94 to 155.4) | 649338.01(376852.44 to 1091616.22) | 60.96(35.19 to 102.9) | 284570.26(106793.23 to 909459.5) | 26.03(10.39 to 80.36) | 207053.74(128858.64 to 364853.85) | 17.78(10.74 to 32.17) |
| Indonesia | 1424623.89(785597.53 to 1920743.71) | 639.16(351.89 to 861.18) | 698665.56(501830.7 to 927272.66) | 317.12(227.69 to 423.92) | 263178.39(190153.19 to 365712.88) | 117.3(84.69 to 163.1) | 137437.38(80097.72 to 199044.51) | 64.07(37.12 to 93.25) | 177617.01(72678.42 to 316768.11) | 79.66(32.55 to 142.22) | 114836.89(64101.31 to 203677.56) | 53.49(29.7 to 95.31) | 38228.98(22332.08 to 78900.06) | 17.2(10.07 to 35.52) | 43924.91(31068.42 to 63196.69) | 19.12(13.2 to 27.7) |
| Iran (Islamic Republic of) | 1214519.61(663320.43 to 1645640.06) | 1581.07(863.23 to 2136.62) | 99049.33(75688.55 to 130022.63) | 162.84(121.28 to 218.59) | 260775.43(152258.93 to 355775.44) | 338.12(198.55 to 459.76) | 12651.85(9977.98 to 15444.54) | 19.41(14.82 to 23.9) | 132131.98(61608.95 to 226374.42) | 174.16(81.42 to 298.09) | 12114.39(7283.99 to 17440.49) | 22.07(12.76 to 32.48) | 94349.53(42308.34 to 224005.93) | 122.87(55.32 to 290.24) | 14913.73(11394.76 to 19481.33) | 23.1(17.44 to 30.87) |
| Iraq | 418850.28(228410.9 to 572855.6) | 1319.75(732.36 to 1799.55) | 176530.2(127372.96 to 245522.91) | 426.76(307.38 to 593.5) | 36123.17(23135.83 to 53306.71) | 115.59(75.68 to 169.03) | 16524.38(12015.81 to 22077.23) | 39.61(28.68 to 53.06) | 38109.79(12280.04 to 73287.21) | 117.38(38.26 to 225.2) | 20973.94(13936.18 to 33374.74) | 51.76(34.31 to 82.49) | 11933.63(6097.99 to 25107.62) | 39.21(20.72 to 79.89) | 8419.98(4919.78 to 12171.81) | 19.96(11.57 to 28.97) |
| Ireland | 7230.64(6362.32 to 7914.26) | 251.22(221.37 to 274.76) | 2643.72(2114.95 to 3313.01) | 79.56(63.75 to 98.82) | 3663.04(3252.32 to 4111.66) | 130.42(115.52 to 146.91) | 972.52(789.36 to 1204.89) | 29.99(23.89 to 37.8) | 1404.72(1162.69 to 1650.21) | 51.78(42.72 to 60.99) | 536.61(437.61 to 667.68) | 18.24(14.68 to 23.09) | 1057.41(914.41 to 1225.48) | 32.78(28.75 to 37.95) | 1619.99(1361.84 to 1873.18) | 39.14(32.16 to 46.26) |
| Israel | 15067.59(12159.35 to 17081.54) | 297.04(239.56 to 336.59) | 6901.88(5560.63 to 8376.14) | 75.95(60.96 to 92.12) | 3157.9(2534.64 to 3940.49) | 62.24(49.95 to 77.7) | 1402.58(1081.87 to 1758.28) | 15.48(11.92 to 19.41) | 2313.61(1774.06 to 3110.2) | 45.63(34.99 to 61.35) | 1244.77(984.72 to 1630.46) | 13.82(10.92 to 18.11) | 1464.61(1219.02 to 1762.19) | 29.33(24.5 to 35.25) | 2108.75(1749.72 to 2494.36) | 22.81(18.86 to 27.07) |
| Italy | 88968.75(78981.06 to 99467.4) | 287.78(253.2 to 320.44) | 28528.85(23098.63 to 34162.53) | 94.14(75.3 to 111.67) | 8245.69(7481.11 to 9394.47) | 27.73(25.34 to 31.56) | 2235.22(1773.83 to 2773.21) | 7.86(5.79 to 9.87) | 17182.44(13597.27 to 19298.17) | 60.72(47.46 to 68.07) | 3950.12(3044.41 to 4961.98) | 16.82(12.56 to 21.67) | 12977.8(11196.71 to 14849.84) | 34.67(29.04 to 39.86) | 12051.65(10860.85 to 13533.59) | 24.24(20.62 to 28.64) |
| Jamaica | 10178.87(7924.36 to 12357.59) | 369.42(288.35 to 447.51) | 4155.1(3152 to 5439.91) | 234.84(174.43 to 310.55) | 1182.72(937.77 to 1499.22) | 42.86(34.04 to 54.33) | 388.91(292.44 to 511.89) | 21.56(15.86 to 28.86) | 2742.49(2008.33 to 3590.05) | 99.15(72.63 to 129.73) | 1183.39(867.68 to 1625.98) | 72.15(52.36 to 99.28) | 624.28(473.77 to 791.09) | 23.84(18.59 to 29.8) | 543.03(411.23 to 702.94) | 23.7(17.84 to 31.41) |
| Japan | 216688.26(193886.18 to 236031.99) | 300.02(268.27 to 325.98) | 56688.49(44439.9 to 71150.37) | 85.99(65.86 to 107.35) | 8913.75(7363.62 to 10597.11) | 11.49(9.79 to 13.75) | 5882.69(4662.38 to 7460.14) | 8.61(6.83 to 10.64) | 26926.37(22427.1 to 33476.09) | 41.5(34.38 to 51.72) | 7866.99(6179.03 to 9746.98) | 15.49(11.92 to 19.73) | 18393.11(15379.8 to 21984.73) | 22.82(19.24 to 27.16) | 10464.5(8694.6 to 12448) | 14.51(11.83 to 17.51) |
| Jordan | 59733.59(41815.12 to 74909.63) | 970.05(682.52 to 1212.67) | 36248.97(28621.67 to 48111.9) | 333.62(262.11 to 445.53) | 4990.46(3545.77 to 6817.59) | 81.96(58.63 to 110.81) | 2374.4(1813.56 to 3073.1) | 21.35(16.11 to 27.95) | 8384.73(4883.58 to 12836.49) | 133.61(78.15 to 204.11) | 6899.82(4718.94 to 9875.71) | 65.46(44.51 to 93.92) | 5558.15(3467.12 to 9364.62) | 93.44(58.98 to 155.44) | 5601.13(4164.4 to 7176.52) | 50.07(37.13 to 64.87) |
| Kazakhstan | 81857.71(63510.44 to 102205.35) | 454.91(353.69 to 567.44) | 66058.95(53652.37 to 80462.33) | 336.23(273.61 to 408.94) | 14987.86(10219.68 to 20274.19) | 83.51(57 to 112.79) | 3232.73(2446.74 to 4287.15) | 16.65(12.63 to 22.03) | 15743.14(11054.24 to 19993.62) | 87.83(61.75 to 111.57) | 12037.91(8892.48 to 16671.8) | 60.78(44.99 to 84.17) | 2786.22(1798.85 to 4119.99) | 15.47(10.02 to 22.79) | 2473.64(1655.46 to 3821.76) | 12.66(8.52 to 19.39) |
| Kenya | 129920.78(57857.3 to 270677.38) | 296.17(137.39 to 612.91) | 96876.11(56673.97 to 182098.47) | 167.94(98.34 to 316.61) | 93479(62294.55 to 133312.94) | 206.79(139.27 to 294.22) | 66527.14(38753.39 to 106967.72) | 115.91(67.51 to 185.62) | 42041.2(16763.4 to 79177.75) | 92.24(37.25 to 173.11) | 34755.96(20646.88 to 66939.75) | 60.96(36.16 to 117.63) | 17320.75(6734.23 to 49570.35) | 41.07(16.97 to 114.25) | 17844(11593.03 to 32689.54) | 31.57(20.49 to 58.07) |
| Kiribati | 1095.61(303.44 to 1692.86) | 917.23(264.05 to 1401.28) | 769.72(346.59 to 1101.67) | 549.16(249.15 to 785.76) | 169.99(56.97 to 283.22) | 135.22(46.13 to 224.57) | 121.15(51.08 to 191.57) | 86.09(36.33 to 136.05) | 40.36(12.56 to 84.17) | 32.41(10.32 to 66.91) | 29.56(13.53 to 72.15) | 21.15(9.71 to 51.36) | 132.76(25.78 to 324.4) | 114.23(24.41 to 272.39) | 149.29(46.91 to 237.85) | 107.11(34.5 to 170.15) |
| Kuwait | 11749.83(9751.62 to 14317.66) | 675.24(558 to 824.02) | 6462(5362.13 to 7827.05) | 239.48(197.07 to 292.38) | 1638.38(1267.9 to 2040.71) | 93.99(72.54 to 117.4) | 561.07(456.45 to 686.54) | 18.76(15.26 to 23.21) | 1666.75(1200.07 to 2111.05) | 98.42(70.62 to 124.99) | 1463.35(1118.51 to 1932.42) | 57.87(43.65 to 76.4) | 524.28(413.56 to 662.22) | 28.95(22.74 to 36.65) | 750.22(543.99 to 951.32) | 25.79(18.54 to 33.12) |
| Kyrgyzstan | 25154.15(20651.22 to 29223.61) | 408.37(337.13 to 473.24) | 26912.75(21983.07 to 32269.8) | 355.11(289.76 to 425.62) | 6943.34(5046.19 to 8904.06) | 110.63(80.5 to 141.43) | 2501.36(1655.12 to 3703.75) | 33.36(22.09 to 49.3) | 7702.88(4897.26 to 9954.46) | 122.06(77.84 to 157.55) | 6889.83(5574.41 to 8500.36) | 91.18(73.79 to 112.59) | 1531.5(962.1 to 2001.46) | 24.89(15.79 to 32.38) | 551.41(433.82 to 762.43) | 7.29(5.74 to 10.06) |
| Lao People's Democratic Republic | 134198.24(31371.45 to 205246.7) | 1786.88(439 to 2702.01) | 70563.66(44478.25 to 101158.68) | 845.73(535.03 to 1211.07) | 21833.47(6992.81 to 37309.28) | 272.23(87.51 to 466.93) | 10644.55(6079.1 to 16275.22) | 126.79(72.48 to 193.79) | 14322.8(2207.34 to 36103.67) | 183.86(28.34 to 466.65) | 9675.68(4262.77 to 18225.75) | 115.51(51.07 to 217.51) | 2909.66(571.25 to 10749.81) | 40.7(8.54 to 145) | 1819.6(1064.27 to 2798.28) | 21.93(12.89 to 33.6) |
| Latvia | 10534.95(9346.22 to 12279.39) | 552.09(488.09 to 646.54) | 971.75(810.66 to 1227.48) | 89(73.02 to 115.54) | 1837.27(1547.81 to 2275.06) | 97.36(81.75 to 121.18) | 109.6(84.42 to 140.44) | 9.59(7.23 to 12.27) | 997.56(740.69 to 1291.69) | 54.66(40.56 to 70.95) | 87.59(57.55 to 152.84) | 9.17(5.72 to 16.46) | 2015.48(1673.5 to 2390.29) | 102.33(84.36 to 122.16) | 290.25(239.3 to 349.29) | 24.95(20.19 to 30.5) |
| Lebanon | 20850.56(9454.26 to 30749.64) | 523.58(237.89 to 770.21) | 6328.36(4548.31 to 8768.55) | 152.48(108.4 to 216.17) | 2408.87(1276.25 to 3691.1) | 61.45(33.47 to 93.38) | 784.69(577.43 to 1040.21) | 17.84(12.84 to 23.97) | 4708.84(1659.1 to 9582.99) | 116.13(41.19 to 236.06) | 1500.16(980.27 to 2269.16) | 38.08(24.58 to 58.05) | 1125.7(516.71 to 2380.76) | 29.2(13.72 to 60.82) | 719.51(523.7 to 975.77) | 16.4(11.63 to 22.74) |
| Lesotho | 6522.41(3723.01 to 9923.6) | 268.62(157.09 to 405.4) | 5335.74(3256.68 to 8045.46) | 263.94(161.02 to 398.09) | 2160.26(1288.65 to 3426.69) | 87.7(52.78 to 138.4) | 1545.1(949.15 to 2489.16) | 76.39(46.89 to 123.22) | 1212.72(698.78 to 1845.67) | 48.76(28.34 to 73.83) | 1156(612.79 to 1818.69) | 57.68(30.59 to 90.83) | 1125.01(465.89 to 1847.84) | 47.17(20.76 to 76.24) | 1197.03(653.92 to 1847.96) | 60(33.07 to 92.86) |
| Liberia | 60490.48(11166.52 to 104887.97) | 1252.66(244.26 to 2157.19) | 33251.61(20164.39 to 48984.51) | 442.46(273.75 to 647.58) | 24832.52(8254.63 to 39051.76) | 499.62(168.77 to 789.22) | 11975.63(7950.74 to 16995.57) | 157.22(104.64 to 223.51) | 15547.05(2969.74 to 29247.51) | 308.3(59.34 to 580.52) | 8920.61(5407.4 to 13572.86) | 116.44(70.89 to 176.91) | 5091.26(883.36 to 18102.26) | 112.41(22.77 to 379.58) | 4910.74(2625.52 to 10583.94) | 70.82(38.18 to 146.53) |
| Libya | 70788.26(44948.1 to 95244.99) | 1139.96(728.93 to 1530.83) | 26378.55(17969.93 to 36272.43) | 615.72(413.41 to 863.68) | 13312.74(8857.77 to 18836.52) | 215.66(144.36 to 303.46) | 4664.99(2943.45 to 6801.78) | 104.24(64.62 to 154.35) | 8913.71(4679.01 to 14793.18) | 142.73(75.27 to 236.53) | 4089.38(2142.96 to 6986.88) | 101.49(52.53 to 173.93) | 4956.9(2870.35 to 9351.37) | 81.44(48.37 to 151.58) | 5488.68(2420.66 to 13279.28) | 117.32(50.07 to 292.48) |
| Lithuania | 13303.44(12025.16 to 15351.24) | 465.17(419.73 to 537.84) | 1749.3(1430.93 to 2140.44) | 118.32(92.82 to 148) | 2778.4(2347.32 to 3267.24) | 98.26(83.11 to 116.17) | 212.22(165.28 to 272.14) | 12.4(9.32 to 15.84) | 2358.24(1913.35 to 2846.79) | 85.41(69.2 to 103.21) | 256.16(188.22 to 359.06) | 19.95(14.34 to 28.44) | 1306.15(1076.79 to 1528.71) | 44.3(36.38 to 52.07) | 240.73(197.26 to 293.47) | 14.44(11.37 to 18.21) |
| Luxembourg | 451.89(383.59 to 509.64) | 170.57(144.8 to 192.35) | 220.88(161.92 to 292.5) | 52.26(39.96 to 68.98) | 89.05(76.7 to 103.54) | 34.32(29.36 to 40.19) | 37.88(30.42 to 46.55) | 9.05(7.13 to 11.16) | 53.84(44.58 to 75.24) | 21.43(17.74 to 30.18) | 27.71(21.07 to 47.58) | 7.22(5.4 to 13.07) | 35.01(28.06 to 43.1) | 10.53(8.44 to 13) | 61.85(50.63 to 74.26) | 10.99(8.96 to 13.38) |
| Madagascar | 125324.36(37120.26 to 253522) | 550.2(166.6 to 1106.98) | 122355.83(69999.19 to 214214.73) | 307.24(179.46 to 537.86) | 80001.05(45015.95 to 125718.11) | 342.69(192.57 to 538.99) | 83725.62(47618.79 to 126475.75) | 208.27(118.51 to 314.11) | 31909.88(8549.42 to 65719.18) | 134.92(36.83 to 278.78) | 34360.84(16580.62 to 58354.55) | 85.43(41.74 to 144.97) | 16454.25(3593.76 to 59350.8) | 75.26(18.05 to 266.88) | 20143.45(8487.36 to 45648.52) | 51.92(22.47 to 116.58) |
| Malawi | 172969.05(40910.72 to 335747.72) | 814.86(199.16 to 1567.38) | 83288.93(51240.06 to 137690.45) | 310.36(191.21 to 513.09) | 120608.77(62640.73 to 183991.88) | 547.87(281.94 to 838.45) | 56830.94(31990.15 to 98340.67) | 209.62(118.09 to 362.78) | 42928.01(8872.55 to 89799.47) | 193.17(40.63 to 409.61) | 23785.46(14319.84 to 38977.03) | 87.85(53.26 to 143.86) | 21871.4(3783.22 to 82666.79) | 107.37(20.59 to 400.87) | 14945.58(7656.25 to 30301.57) | 57.21(29.99 to 114.67) |
| Malaysia | 71784.54(45583.68 to 93022.2) | 309.9(198.41 to 399.85) | 39576.11(31840.35 to 48840.98) | 153.03(121.21 to 192.36) | 17407.63(11818.97 to 25186.53) | 73.65(50.22 to 106.11) | 7352.91(5211.88 to 10251.64) | 30.27(20.96 to 42.9) | 6204.39(3468.7 to 11701.53) | 26.31(14.84 to 49.4) | 4051.36(2263.12 to 8446.22) | 16.71(9.07 to 35.72) | 3013.04(2132.1 to 4222.02) | 13.41(9.6 to 18.68) | 2632.75(2042.14 to 3252.09) | 9.37(7.13 to 11.93) |
| Maldives | 2979.48(930.96 to 4440.59) | 743.3(236.74 to 1098.57) | 736.62(558.53 to 994.42) | 228.45(170.49 to 313.23) | 488.04(210.12 to 805.32) | 116.59(50.78 to 190.86) | 113.92(73.62 to 170.8) | 36.91(22.99 to 56.74) | 493.93(135.18 to 1104.3) | 118.74(32.92 to 264.18) | 157.61(97.2 to 284.66) | 52.28(31.64 to 95.55) | 145.01(48.06 to 415.16) | 37.93(13.22 to 106.18) | 124.18(90.67 to 169.55) | 35.66(25.65 to 49.55) |
| Mali | 260617.85(132451.66 to 435151.7) | 1470.37(765.66 to 2434.33) | 310706.5(177559.56 to 459658.98) | 689.03(387.47 to 1028.02) | 90560.47(45832.05 to 156316.24) | 485.21(240.55 to 855.32) | 109256.36(65724.41 to 164153.98) | 231.7(139.38 to 351.22) | 53414.62(21981.16 to 93230.23) | 278.22(110.71 to 495.91) | 77394.58(42675.04 to 115220.8) | 159.79(86.98 to 240.27) | 18511.32(5896.89 to 59317.56) | 114.69(41.93 to 336.82) | 34590.29(17209.75 to 66848.87) | 85.99(45.17 to 151.37) |
| Malta | 874.34(687.92 to 986.25) | 306.65(239.33 to 347.43) | 334.3(271.18 to 408.95) | 136.66(110.81 to 167.83) | 253.01(219.02 to 295.37) | 90.29(77.61 to 105.79) | 84.38(66.88 to 105.04) | 34.56(27.16 to 43.25) | 201.38(158.61 to 249.13) | 73.5(57.71 to 91.15) | 82.62(64.3 to 104.94) | 37.05(28.52 to 47.23) | 90.38(77.07 to 105.9) | 27.34(23.33 to 31.8) | 142.87(119.02 to 169.84) | 43.72(35.59 to 53.17) |
| Marshall Islands | 296.1(155.9 to 398.33) | 436.07(232.47 to 582.21) | 194.55(136.97 to 276.98) | 339.83(238.42 to 484.85) | 53.55(29.8 to 83.63) | 75.31(42.32 to 117.15) | 30.9(17.45 to 50.94) | 55.57(31.28 to 92) | 11.7(5.28 to 29.88) | 16.74(7.75 to 41.91) | 8.29(3.88 to 23.57) | 14.84(6.9 to 42.55) | 29.27(14.35 to 45.51) | 44.33(22.89 to 67.97) | 35.49(17.59 to 56.37) | 62.19(30.64 to 99.18) |
| Mauritania | 18459.27(5332.91 to 27106.17) | 498.52(151.08 to 723.98) | 15504.63(10854.42 to 21310.59) | 248.52(175.25 to 341.75) | 7180.22(2834.51 to 11491.12) | 187.02(75.29 to 297.92) | 5175.61(3140.15 to 7880.53) | 82.13(51 to 124.79) | 4690.87(1748.74 to 7523.13) | 118.95(45.04 to 192.27) | 4564.9(2376.97 to 7801.38) | 71.32(37.16 to 122.35) | 2059.95(528.87 to 5907.5) | 62.66(17.12 to 166.75) | 3197.18(1743.86 to 5582.68) | 55.75(31.2 to 94.4) |
| Mauritius | 4505.07(3898.82 to 5612.5) | 401.48(347.05 to 501.43) | 2358.87(1970.94 to 3033.43) | 328.05(269.85 to 427.63) | 650.04(497.98 to 860.31) | 58.34(44.66 to 77.21) | 186.16(140.95 to 257.82) | 27.11(20.27 to 38.48) | 638.62(495.89 to 813.57) | 57.81(44.91 to 73.71) | 393.58(317.97 to 474.41) | 60.67(48.63 to 73.73) | 87.87(67.15 to 121.13) | 7.73(5.87 to 10.79) | 74.56(59.28 to 96.02) | 9.05(7.13 to 11.92) |
| Mexico | 551727.44(483506.5 to 652778.04) | 469.26(412.73 to 552.48) | 436767.9(344284.24 to 546054.86) | 447.62(348.34 to 565.01) | 220658.53(169575.72 to 253461.14) | 183.9(141.55 to 211.12) | 37014.1(29639.05 to 46611.33) | 37.43(29.24 to 47.39) | 128267.78(110200.22 to 160559.09) | 106.69(91.68 to 133.36) | 86948.14(64401.49 to 112662.92) | 92.91(68.36 to 120.71) | 48934.68(39168.48 to 56662.53) | 42.43(34.48 to 48.96) | 42789.48(33979.29 to 54104.63) | 39.65(31.16 to 51.06) |
| Micronesia (Federated States of) | 885.58(397.83 to 1247.54) | 603.56(276.87 to 849.09) | 273.88(210.5 to 365.29) | 280.48(212.92 to 376.18) | 139.85(67.46 to 215.14) | 92.92(45.14 to 142.69) | 39.53(22.71 to 65.01) | 42.92(24.36 to 71.2) | 35.72(14.66 to 75.69) | 24.04(9.95 to 50.24) | 12.18(6.25 to 36.04) | 13.12(6.6 to 39.57) | 120.46(39.5 to 221.58) | 83.49(29 to 149.39) | 54.11(31.23 to 82.61) | 55.9(31.75 to 85.92) |
| Monaco | 35.49(26.51 to 46.15) | 223.54(164.06 to 293.99) | 25.61(19.28 to 32.46) | 123.83(93.13 to 162.24) | 3.66(2.05 to 5.7) | 23.51(12.42 to 38.1) | 2.3(1.64 to 3.06) | 10.75(7.41 to 14.69) | 5.76(3.44 to 9.2) | 42.59(24.41 to 69.37) | 3.49(1.95 to 5.62) | 19.55(10.16 to 33.11) | 4.05(2.84 to 6.13) | 17.69(12.04 to 27.2) | 9.31(6.33 to 12.98) | 30.16(21.63 to 41.97) |
| Mongolia | 24384.86(11241.75 to 35297.39) | 728.87(342.28 to 1049.84) | 10528.34(7566.69 to 13621.37) | 286.17(206.28 to 369.31) | 3202.97(1706.38 to 5172.38) | 94.79(51.13 to 152.52) | 457.47(301.44 to 659.68) | 12.62(8.36 to 18.14) | 7606.16(3682.14 to 14302.54) | 222.3(107.76 to 417.55) | 3215.91(2040.94 to 4504.81) | 87.03(55.31 to 121.91) | 3216.62(1551.86 to 6844.87) | 96.34(47.58 to 202.68) | 1353.23(787.42 to 1991.33) | 36.51(21.36 to 53.68) |
| Montenegro | 1270.4(926.82 to 1693.92) | 253.21(183.69 to 338.15) | 226.49(160.78 to 334.77) | 57.05(39.82 to 85.68) | 45.37(29.45 to 68.09) | 8.72(5.64 to 13.36) | 14.6(10.68 to 19.74) | 3.03(2.2 to 4.14) | 231.86(138.29 to 379.7) | 47.45(28.12 to 77.69) | 33.94(17.66 to 65.94) | 9.26(4.67 to 18.38) | 223.64(160.86 to 304.48) | 42.37(30.26 to 58.36) | 88.42(62.31 to 125.12) | 20.18(13.56 to 29.81) |
| Morocco | 253331.58(177472.55 to 337377.66) | 690.3(484.81 to 916.52) | 57923.16(35166.2 to 118141.89) | 181.56(109.6 to 369.08) | 41610.7(26018.12 to 63222.14) | 114.15(71.82 to 171.96) | 7187.56(4346.78 to 15151.05) | 22.2(13.37 to 46.33) | 24387.58(9533.15 to 46911.27) | 65.75(25.83 to 126.31) | 7517.58(4293.77 to 15247.32) | 23.99(13.63 to 48.73) | 15776.12(7371.64 to 40578.7) | 43.7(20.69 to 110.8) | 7426.49(4487.37 to 12533.7) | 22.76(13.56 to 38.64) |
| Mozambique | 247584.19(60689.23 to 476375.97) | 949.94(243.35 to 1808.38) | 211956.22(120750.58 to 375746.93) | 418.4(243.42 to 741.79) | 140279.58(65568.61 to 237835.36) | 524.44(243.31 to 888.15) | 119082.84(72157.95 to 184422.59) | 230.57(139.73 to 356.23) | 54930.98(10985.9 to 121358.55) | 203.02(41.39 to 450.9) | 56751.93(27444.9 to 100180.01) | 109.37(53.8 to 192.14) | 31400.29(5013.55 to 130012.55) | 124.19(22.31 to 506.71) | 31286.86(15311.86 to 71700.9) | 63.9(32.79 to 143.04) |
| Myanmar | 889331.56(274311.46 to 1412312.82) | 1724.36(533.54 to 2740.69) | 459686.97(286194.95 to 638790.88) | 880.79(545.06 to 1226.29) | 129893.1(55870.24 to 211830.17) | 246.31(105.52 to 404.56) | 67560.16(40634.99 to 101175.36) | 130.69(78.44 to 196.01) | 91378.61(23018.8 to 208239.52) | 175.93(43.85 to 401.85) | 65125.7(33226.37 to 105612.95) | 126.11(64.19 to 204.68) | 24137.13(5673.56 to 80771.07) | 47.53(11.29 to 158.47) | 21217.15(11686.71 to 41262.95) | 40.16(21.94 to 78.4) |
| Namibia | 4972.51(3222.96 to 6835.09) | 220.77(145.74 to 299.67) | 4465.56(3000.31 to 6502.61) | 163.67(110.03 to 237.38) | 1472.1(883.59 to 2289.81) | 64.14(39.12 to 98.53) | 1030.64(624.12 to 1542.49) | 37.72(22.84 to 56.32) | 1013.26(552.44 to 1624.17) | 43.25(23.81 to 68.74) | 1071.36(485.77 to 1895.8) | 39.1(17.77 to 69.02) | 960.94(429.72 to 1424.89) | 43.56(20.36 to 63.92) | 1163.75(652.59 to 1779.87) | 42.95(24.08 to 65.39) |
| Nauru | 84.85(44.04 to 118.4) | 546.38(286.4 to 759.58) | 60.84(38.98 to 87.33) | 446.99(286.87 to 641.66) | 11.16(6.31 to 17.21) | 67.59(38.58 to 103.61) | 8.02(4.61 to 11.88) | 57.8(33.33 to 85.61) | 3.45(1.65 to 8.33) | 21.13(10.29 to 49.81) | 2.67(1.24 to 6.77) | 19.41(9.08 to 48.84) | 12.96(4.87 to 22.92) | 84.27(32.56 to 147.15) | 11.91(6.22 to 19.42) | 88.16(46.23 to 143.38) |
| Nepal | 170338.65(119041.1 to 228959.28) | 494.23(349.58 to 658.16) | 52944.37(30019.77 to 111668.68) | 169.69(96 to 358.14) | 37663.01(23128.79 to 56989.05) | 105.82(66.44 to 158.2) | 9009.32(5056.48 to 19071.36) | 28.88(16.12 to 61.39) | 21075.63(7278.64 to 40657.71) | 58.28(20.52 to 111.77) | 8832.71(4335.46 to 20623.66) | 28.59(14 to 66.82) | 6220.72(2201.15 to 18069.15) | 19.47(7.5 to 54.51) | 2417.46(1204.44 to 6859.38) | 7.78(3.81 to 22.19) |
| Netherlands | 26475.74(22473.51 to 29181.07) | 254.36(212.21 to 279.98) | 8180.92(6676.78 to 10095.75) | 75.75(62.83 to 93.41) | 6655.23(5865.16 to 7618.8) | 65.26(57.42 to 75.12) | 1970.91(1644.81 to 2346.01) | 18.34(15.35 to 21.73) | 4833.02(4107.47 to 5975.73) | 49.45(41.85 to 61.35) | 1860.68(1575.04 to 2233.71) | 19.76(16.63 to 23.83) | 4575.07(4130.8 to 5090.44) | 36.92(33 to 41.42) | 7653.12(6923.46 to 8351.64) | 48.63(42.26 to 54.4) |
| New Zealand | 5859.51(5313.64 to 6450.91) | 194.78(175.85 to 214.96) | 2922.86(2451.68 to 3645.77) | 80(65.27 to 100.42) | 1660.85(1429.24 to 1865.52) | 55.83(47.72 to 62.95) | 568.24(476.1 to 677.49) | 16.45(13.56 to 19.75) | 1358.29(1066.66 to 1566.04) | 46.61(36.55 to 53.72) | 542.04(431.8 to 724.73) | 17.31(13.73 to 23.1) | 725.07(620.33 to 833.27) | 22.74(19.43 to 26.12) | 1478.7(1266.71 to 1676.79) | 32.82(27.07 to 37.81) |
| Nicaragua | 50431.37(26345.24 to 72702.94) | 763.28(404.38 to 1095.81) | 16460.59(11488.67 to 22994.88) | 260.11(180.69 to 364.38) | 10943.76(7506.89 to 15924.56) | 163.95(112.92 to 237.98) | 2950.4(2048.84 to 4180.77) | 46.88(32.33 to 66.82) | 15131.67(7905.55 to 25034.52) | 224.8(117.66 to 372.38) | 6690.43(4201.63 to 10103.89) | 107.64(67.58 to 162.74) | 1852.06(1054.03 to 3658.3) | 29.37(17.21 to 56.19) | 1075.51(673.91 to 1489.43) | 16.49(10.17 to 22.94) |
| Niger | 176389.57(28642.17 to 300628.21) | 984.72(168.49 to 1676.96) | 243756.28(107974.5 to 372528.9) | 482.46(221.6 to 731.27) | 44530.83(10362.47 to 87606.05) | 240.43(56.6 to 467.73) | 57672.9(28334.36 to 95609.56) | 111.27(56.05 to 181.09) | 33630.25(7474.59 to 61960.04) | 175.64(38.01 to 334.77) | 47024.92(25405.19 to 71286.12) | 87.51(47.29 to 133.32) | 12430.37(1668.24 to 45584.76) | 76.26(11.94 to 264.63) | 22215.15(10085.43 to 49846.2) | 49.43(23.2 to 103.14) |
| Nigeria | 1229497.27(298401.29 to 1875860.59) | 726.28(183.83 to 1112.35) | 1996675.17(1069619.73 to 3002588.54) | 538.04(298.04 to 805.65) | 533507.62(207786.61 to 843576.37) | 305.05(119.82 to 485.06) | 798459.56(467807.31 to 1168733.07) | 211.33(125.36 to 307.16) | 301888.31(87398.64 to 485882.52) | 167.4(47.96 to 273.04) | 617961.84(329526.94 to 898089.82) | 161.37(86.85 to 234.57) | 97773.66(26443.91 to 299023.74) | 63.02(19.11 to 183.49) | 268795.7(135861.6 to 652061.05) | 77(41.84 to 177.92) |
| Niue | 9.97(6.9 to 13.31) | 422.79(293.39 to 565.34) | 12.25(9.75 to 15.16) | 1026.71(811.13 to 1278.38) | 1.36(0.81 to 2.12) | 59.9(35.13 to 93.47) | 1.18(0.83 to 1.61) | 104.39(72.7 to 142.32) | 0.46(0.23 to 1.2) | 20.32(10.04 to 52.76) | 0.62(0.33 to 1.75) | 54.86(28.38 to 156.24) | 1.47(0.82 to 2.31) | 61.68(35.22 to 96.37) | 2.91(1.73 to 4.43) | 244.11(145.21 to 373.63) |
| North Macedonia | 12153.03(8772.29 to 15298.12) | 730.86(527.57 to 920.38) | 1167.95(898.48 to 1559.8) | 106.33(80.96 to 144.41) | 2459.69(1506.87 to 3889.16) | 148.97(90.7 to 236.07) | 163.74(121.09 to 221.7) | 14.66(10.26 to 20.49) | 2270.54(986.27 to 4152.94) | 138.14(59.84 to 253) | 252.94(154.9 to 388.66) | 26.15(15.69 to 40.55) | 504.04(261.91 to 1248.52) | 29.03(14.47 to 74.15) | 196.06(149.39 to 257.48) | 14.49(10.55 to 19.79) |
| Northern Mariana Islands | 90.49(66.46 to 122.35) | 167.74(124.22 to 227.12) | 43.63(33.24 to 56.2) | 121.22(91.64 to 159.01) | 22.09(13.92 to 34.09) | 38.18(24.2 to 58.43) | 14.87(10.75 to 20.02) | 49.09(35.05 to 66.73) | 6.31(3.11 to 17.48) | 11.17(5.64 to 30.57) | 1.27(0.69 to 2.84) | 3.27(1.68 to 8.53) | 12.52(5.78 to 19.6) | 24.19(11.13 to 37.97) | 9.11(3.83 to 14.29) | 24.96(9.8 to 39.59) |
| Norway | 8122.27(7472.1 to 8886.26) | 261.39(241 to 287.16) | 2492.61(1895.72 to 3507.37) | 68.34(52.27 to 100.36) | 507.71(448.84 to 583.44) | 16.53(14.69 to 18.95) | 338.08(268.92 to 423.29) | 9.32(7.29 to 11.49) | 1546.88(783.78 to 1838.21) | 53.27(26.63 to 63.44) | 382.54(252.3 to 515.11) | 13.04(8.31 to 17.9) | 1009.1(892.61 to 1138.53) | 26.12(23.08 to 29.61) | 1443.6(1305.58 to 1586.98) | 25.76(22.54 to 28.88) |
| Oman | 22725.8(13303.58 to 32370.58) | 681.42(400.44 to 967.01) | 7957.66(6212.66 to 10435.5) | 198.07(154.77 to 260.75) | 15257.51(9747.2 to 21926.24) | 462.84(296.77 to 662.05) | 4908.36(3439.66 to 6631.41) | 122.18(85.33 to 166.95) | 1569.4(722.3 to 2931.08) | 46.33(21.61 to 86.03) | 906.72(500.37 to 2228.11) | 23.02(12.51 to 57.1) | 472.22(270.43 to 853.82) | 15.2(9.05 to 26.68) | 482.16(374.31 to 617.7) | 11.5(8.86 to 14.89) |
| Pakistan | 1071238.72(736016.01 to 1433538.51) | 562.67(388.15 to 752.08) | 1071387.73(657933.45 to 1571086.18) | 364.84(225.32 to 534.24) | 180954.2(113686.6 to 291360.38) | 92.96(58.91 to 148.22) | 156483.11(73481.37 to 291075.39) | 53.11(25.13 to 98.53) | 159322.64(61475.81 to 309773.38) | 81.16(31.75 to 156.9) | 177544.87(86911.4 to 297764.65) | 60.06(29.59 to 100.7) | 50492.09(21235.27 to 149903.57) | 28.33(13.07 to 80.23) | 65689.16(36666.76 to 129246.61) | 23.34(13.16 to 44.91) |
| Palau | 73.73(40.9 to 106.75) | 483.23(266.07 to 702.22) | 30.65(22.46 to 39.85) | 289.49(207.54 to 380.82) | 11.68(4.54 to 19.06) | 77.6(29.97 to 126.91) | 4.06(2.05 to 6.17) | 44.43(21.9 to 68.48) | 4.29(1.96 to 9.26) | 28.54(12.84 to 61.79) | 1.69(0.98 to 4.02) | 17.32(9.67 to 44.28) | 12.3(5.33 to 19.64) | 81.77(35.56 to 131.23) | 8.21(4.68 to 11.63) | 79.15(42.62 to 115.18) |
| Palestine | 40097.12(25531.88 to 53942.21) | 1002.3(643 to 1343.76) | 16880.18(12787.92 to 23049.1) | 285.05(215.04 to 389.41) | 2245.65(1475.77 to 3055.25) | 58.03(39.51 to 78.67) | 907.64(672.83 to 1193.3) | 15.39(11.41 to 20.28) | 3532.13(1797.46 to 5875.76) | 85.82(43.91 to 142.57) | 1907.18(1169.42 to 3341.6) | 32.64(20.01 to 57.21) | 3162.15(1636.31 to 6313.17) | 82.14(44.03 to 160.42) | 2095.66(1388.94 to 2961.98) | 35.31(23.42 to 50.23) |
| Panama | 18213.6(15425.87 to 21718.91) | 643.96(545.93 to 767.21) | 17118.1(13401.05 to 21597.83) | 480.15(373.69 to 607.28) | 2223.76(1788.79 to 2778.13) | 78.5(63.21 to 98.06) | 1130.16(880.08 to 1446.86) | 31.66(24.5 to 40.69) | 3618.84(2921 to 4423.49) | 127.68(103.08 to 156.02) | 3627.85(2683.37 to 4699.26) | 104.63(77.18 to 135.78) | 816.54(679.43 to 1003.88) | 29.16(24.26 to 35.75) | 1142.72(859.84 to 1463.29) | 30.66(22.83 to 39.75) |
| Papua New Guinea | 70313.1(20924.1 to 108525.3) | 1057.28(329.39 to 1622.19) | 135212.4(54032.44 to 206019.23) | 868.19(362.8 to 1316.05) | 11391.13(5053.64 to 20051.68) | 165.35(73.69 to 290.69) | 24448.73(12093.99 to 40110.83) | 152.02(76.07 to 248.91) | 2230.01(701.21 to 5436.87) | 32.7(10.61 to 79.36) | 4728.52(1763.61 to 11293.43) | 29.7(11.42 to 70.17) | 7062.64(1416.27 to 18769.25) | 109.8(23.62 to 284.78) | 20401.87(5852.04 to 38910.62) | 133.57(40.49 to 249.07) |
| Paraguay | 26345.71(19713.94 to 37403.7) | 434.75(326.37 to 614.79) | 19713.89(12995.72 to 28611.41) | 307.39(201.83 to 448.43) | 4805.29(3426.93 to 6759.32) | 78.4(56.17 to 109.99) | 3511.31(2083.87 to 5515.58) | 55.65(32.85 to 87.9) | 3555.08(2173.94 to 5707.47) | 57.97(35.56 to 92.72) | 3000.1(1589.36 to 5343.67) | 47.47(24.99 to 84.92) | 2253.26(1409.3 to 3947.96) | 38.86(24.91 to 66.56) | 2675.43(1665.4 to 3923.56) | 40.87(25 to 60.53) |
| Peru | 247323.44(132294.2 to 330169.91) | 828.77(444.76 to 1103.61) | 86380.74(57398.48 to 120262.92) | 260.36(171.67 to 363.51) | 42129.43(26113.18 to 59528.14) | 139.23(86.74 to 196.24) | 12484.33(8449.84 to 18007.9) | 37.79(25.47 to 54.87) | 53970.78(26450.42 to 89890.5) | 177.64(87.35 to 295.61) | 20716.41(12660.59 to 30963.7) | 63.34(38.57 to 94.76) | 21303.86(10243.59 to 42610.79) | 71.82(34.75 to 142.82) | 9086.43(5506.86 to 13296.95) | 27.29(16.39 to 40.1) |
| Philippines | 657448.51(400893.81 to 909939.68) | 712.34(437.53 to 978.32) | 421054.98(340454.18 to 537930.56) | 374.05(301.48 to 480.51) | 78335.05(56980.22 to 115674.49) | 82.65(60.28 to 121.61) | 62812.39(49822.42 to 80686.82) | 56.89(45.07 to 73.21) | 89756(52362.32 to 150315.37) | 94.74(55.2 to 158.72) | 68757.07(50437.32 to 96587.64) | 62.31(45.68 to 87.51) | 23834.36(14581.04 to 46419.22) | 26.61(16.62 to 50.95) | 25588.94(19168.81 to 31080.9) | 22.44(16.79 to 27.31) |
| Poland | 156707.56(131646.17 to 178940.1) | 563.78(472.05 to 646.22) | 29981.77(24363.88 to 35380.25) | 153.3(121.68 to 183.46) | 27906.18(24450.18 to 33747.94) | 102.34(89.77 to 123.72) | 2404.21(1901.6 to 2948.62) | 11.23(8.53 to 14.12) | 23193.09(18151.56 to 28616.16) | 86.52(67.57 to 106.85) | 4019.11(2918.63 to 5315.12) | 23.11(16.58 to 30.99) | 1377.69(934.38 to 1946.86) | 4.04(2.74 to 5.76) | 2461.83(1833.4 to 2910.41) | 8.73(6.68 to 10.44) |
| Portugal | 25507.48(21744.45 to 29049.3) | 413.96(349.52 to 474.08) | 4841.98(4026.46 to 5752.12) | 88.34(70.77 to 104.88) | 3754.01(3288.63 to 4389.88) | 61.35(53.61 to 71.99) | 612.85(483.86 to 763.47) | 10.04(7.69 to 12.38) | 4166.45(3455.15 to 5326.88) | 72.49(59.85 to 92.91) | 760.93(575.97 to 996.82) | 16.49(11.95 to 21.88) | 1953.89(1666.18 to 2272.27) | 25.2(21.42 to 29.48) | 2031.8(1775.97 to 2331.74) | 23.49(19.77 to 27.87) |
| Puerto Rico | 9481.37(8228.2 to 10594.22) | 291.86(253.1 to 326.81) | 2243.64(1841 to 2666.66) | 176.54(140.61 to 212.35) | 2016.14(1742.32 to 2361.64) | 62.33(53.67 to 73.1) | 423.14(342.56 to 513.86) | 30.14(22.73 to 37.79) | 1890.65(1537.02 to 2282.86) | 59.4(48.27 to 71.9) | 446.59(326.88 to 580.49) | 43.68(30.77 to 57.26) | 822.17(727.14 to 947.93) | 24.03(21.27 to 27.63) | 680.47(566.39 to 813.08) | 28.32(23.4 to 34.59) |
| Qatar | 2560.22(1620.46 to 3488.86) | 484.38(305.5 to 655.79) | 2317.39(1718.96 to 3038.93) | 114.64(84.9 to 151.22) | 559.75(374.83 to 785.1) | 106.86(72.27 to 149.32) | 358.73(275.66 to 467.12) | 16.35(12.31 to 21.4) | 170.87(89 to 351.94) | 31.79(16.82 to 65.01) | 214.25(122.64 to 526.6) | 10.75(5.97 to 27.21) | 187.22(124.71 to 275.16) | 36.09(24.3 to 52.61) | 454.17(334.71 to 615.18) | 20.95(15.22 to 28.64) |
| Republic of Korea | 146386.71(95008.08 to 186651.67) | 421.4(269.95 to 540.79) | 17319.21(13310.33 to 23676.78) | 76.09(59.63 to 106.49) | 5317.35(3912.8 to 7147.78) | 14.68(10.56 to 19.98) | 2206.77(1668.97 to 2916.2) | 7.63(5.87 to 9.79) | 14935.04(8983.65 to 22344.42) | 44.63(26.76 to 67.01) | 2294.77(1606.67 to 3787.24) | 12.85(8.29 to 23.43) | 7902.94(5838.95 to 10743.6) | 20.13(14.36 to 27.62) | 4088.4(3221.64 to 5128.57) | 15.07(11.52 to 19.46) |
| Republic of Moldova | 24998.14(18268.76 to 32570.89) | 631.48(460.99 to 824.41) | 4077.81(3181.81 to 5232.83) | 258.95(195.62 to 338.84) | 5339.19(4089.39 to 7162.16) | 135.16(102.74 to 181.36) | 445(341.05 to 575.41) | 26.71(20.04 to 36.24) | 7334.44(4596.9 to 10683.1) | 189.38(118.58 to 276.09) | 1208.77(888.58 to 1668.69) | 85.01(61.68 to 118.47) | 1047.2(763.08 to 1395) | 25.73(18.71 to 34.38) | 206.2(162.77 to 265.74) | 11.19(8.53 to 14.71) |
| Romania | 106537.17(89632.15 to 125008.8) | 664.21(558.93 to 782.31) | 18358.36(15513.19 to 21282.8) | 188.87(158.38 to 219.85) | 20593.1(15904.95 to 26512.69) | 130.92(100.37 to 168.52) | 1844.72(1495.77 to 2223.21) | 16.99(13.53 to 20.26) | 26809.69(16641.23 to 37368.23) | 175.46(108.7 to 244.49) | 4619.65(3634.61 to 5558.07) | 51.42(40.32 to 62.39) | 2718.53(1908.89 to 3553.27) | 15.48(10.52 to 20.5) | 746.21(582.77 to 937.07) | 6.24(4.86 to 7.97) |
| Russian Federation | 435476.37(388079.33 to 536715.88) | 414.43(368.41 to 512.85) | 111831.11(93952.79 to 135165.09) | 133.98(108.56 to 165.61) | 49824.44(45009.96 to 59612.34) | 48.49(43.96 to 58.11) | 9648.19(7709.96 to 12230.36) | 10.14(8.05 to 12.47) | 113300.84(91654.21 to 130926.37) | 114.86(92.78 to 133.22) | 22483.54(16294.46 to 29784.08) | 31.44(22.44 to 42.13) | 42133.29(37209.45 to 50482.68) | 37.82(33.57 to 45.52) | 11117.68(9332.63 to 13307.38) | 12.19(10.05 to 14.51) |
| Rwanda | 95498.6(26468.43 to 179828.95) | 696.06(200.61 to 1303.3) | 51270.37(32306.88 to 83675.67) | 295.97(187.5 to 483.43) | 60731.88(28221.31 to 92870.5) | 428.16(199.43 to 652.36) | 29080.59(17270.52 to 47833.86) | 166.21(98.8 to 272.92) | 24159.86(4431.15 to 50120.43) | 167.89(31.91 to 348.57) | 14211.63(7397.81 to 24158.18) | 81.07(42.49 to 137.47) | 15003(2541.54 to 54759.21) | 115.07(21.6 to 410.03) | 10609.02(4896.09 to 22367) | 62.65(29.74 to 131.09) |
| Saint Kitts and Nevis | 194.25(167.62 to 223.81) | 432.19(373.65 to 497.73) | 86.33(67.92 to 109.55) | 269.61(209.12 to 346.83) | 30.46(24.59 to 37.24) | 67.6(54.5 to 82.64) | 10.78(8.43 to 13.94) | 33.48(25.51 to 44.24) | 31.64(23.47 to 39.68) | 70.41(52.22 to 88.33) | 15.77(12.04 to 20.83) | 53.69(40.62 to 71.41) | 10.88(8.56 to 14.16) | 26.24(20.54 to 33.92) | 11.71(9.09 to 15.34) | 25.03(18.99 to 33.81) |
| Saint Lucia | 614.52(499.23 to 736.95) | 363.27(296.66 to 435.15) | 221.49(166.48 to 292.43) | 234.78(173.21 to 316.35) | 104.33(76.54 to 131.85) | 61.42(45.23 to 77.5) | 35.6(26.72 to 46.92) | 38.16(27.97 to 51.78) | 108.67(75.8 to 156.7) | 63.4(44.25 to 91.42) | 40.79(28.92 to 61.84) | 47.76(33.24 to 73.07) | 34.38(26.71 to 42.1) | 23.06(18.07 to 27.71) | 40.44(31.67 to 51.15) | 28.57(21.74 to 37.05) |
| Saint Vincent and the Grenadines | 593.36(483.62 to 723.76) | 476.39(388.82 to 581.25) | 170.59(134.58 to 216.23) | 231.22(179.76 to 295.78) | 111.14(86.21 to 148.04) | 88.64(68.75 to 117.86) | 31.63(24.66 to 41.15) | 43.18(33 to 57.08) | 129.55(91.76 to 166.5) | 104.35(73.87 to 134.11) | 40.78(30.59 to 54.98) | 61.71(45.64 to 83.77) | 34.03(27.22 to 42.92) | 29.27(23.49 to 36.76) | 32.28(24.83 to 39.33) | 33.37(26.28 to 41.13) |
| Samoa | 1089.3(654.37 to 1514.67) | 437.04(270.06 to 603.51) | 663.35(476.76 to 909.31) | 238.51(172.29 to 325.19) | 162.45(96.28 to 248.19) | 63.49(37.85 to 96.66) | 97.56(52.99 to 156.92) | 33.54(18.54 to 53.62) | 49.17(23.52 to 117.22) | 19.45(9.51 to 45.61) | 34.54(16.58 to 110.47) | 12.11(6.02 to 37.49) | 120.36(59.69 to 195.53) | 49.22(25.63 to 78.38) | 140.65(68.68 to 234.08) | 50.93(24.64 to 83.89) |
| San Marino | 15.45(11.28 to 20.52) | 110.05(79.12 to 150.31) | 6.98(4.38 to 9.93) | 34.86(23.01 to 49.72) | 5.12(3.98 to 6.78) | 38.01(28.81 to 51.96) | 1.79(1.26 to 2.43) | 9.87(6.27 to 14.45) | 4.85(3.19 to 7.15) | 38.97(24.93 to 58.09) | 1.45(0.88 to 2.21) | 9.69(5.39 to 15.65) | 2.18(1.65 to 2.8) | 11.32(8.56 to 14.5) | 2.91(2.13 to 3.9) | 11.53(8.39 to 16.12) |
| Sao Tome and Principe | 1393.77(449.91 to 2106.65) | 676.35(232.41 to 1013.36) | 486.34(302.88 to 778.75) | 200.71(125.38 to 321.24) | 489.58(264.4 to 735.24) | 234.15(127.2 to 352.71) | 149.66(88.07 to 280.24) | 62.11(36.5 to 116.47) | 328.2(135.27 to 496.48) | 154.25(63.95 to 233.85) | 126.32(48.72 to 243.13) | 53.23(20.5 to 102.59) | 122.98(44.3 to 334.85) | 63.87(25.06 to 166.33) | 78.97(45.13 to 133.02) | 35.1(20.08 to 57.98) |
| Saudi Arabia | 237608.69(127041.5 to 340762.6) | 995.64(536.5 to 1423.99) | 35465.87(24891.26 to 51617.47) | 122.35(81.93 to 186.27) | 18601.74(12557.11 to 27056.08) | 79.43(54.28 to 114.11) | 3675.64(2817.82 to 4861.36) | 11.62(8.47 to 15.79) | 19569.6(9626.99 to 32475.28) | 80.86(40.08 to 134.02) | 3699.82(1881.6 to 8511.96) | 14.62(6.83 to 35.47) | 25941.99(13658.3 to 51704.78) | 112.15(59.84 to 218.37) | 11481.28(8270.3 to 16148.44) | 34.29(23.54 to 49.47) |
| Senegal | 108177.99(29260 to 168522.69) | 735.68(208.85 to 1139.18) | 77975.72(56596.1 to 105820.68) | 353.7(258.14 to 476.96) | 41486.21(17129.2 to 65400.72) | 272.87(113.43 to 431.16) | 28704.51(18706.24 to 40100.69) | 127.91(83.21 to 178.15) | 23084.27(8028.2 to 35598.71) | 147.87(51.89 to 230.08) | 19080.07(10124.06 to 34277.06) | 84.19(44.59 to 151.1) | 10189.06(2474.47 to 31179.78) | 75.9(20.94 to 217.24) | 13943.92(8023.54 to 24311.29) | 68.45(40.03 to 114.7) |
| Serbia | 50721.83(32793.66 to 65256.13) | 742.26(476.93 to 956.1) | 5024.43(3926.89 to 6451.95) | 123.29(94.8 to 163.9) | 3698.12(2125.25 to 5791.14) | 54.28(30.71 to 85.63) | 359.19(275.18 to 467.68) | 7.7(5.74 to 10.38) | 6245.85(3243.01 to 10041.57) | 93.3(48.07 to 150.52) | 760.24(470.82 to 1449.88) | 21.2(12.51 to 41.7) | 3333.01(2305.63 to 5126.63) | 45.69(30.62 to 71.94) | 935.72(726.09 to 1193.31) | 17.59(12.44 to 23.09) |
| Seychelles | 342.71(283.2 to 421.47) | 429.95(355.87 to 528.56) | 287.62(219.82 to 366.77) | 357.11(270.64 to 458.72) | 55.32(39.33 to 75.33) | 69.76(49.58 to 95.03) | 43.28(26.62 to 64.2) | 55.86(34 to 83.13) | 49.4(33.77 to 67.82) | 62.32(42.59 to 85.47) | 59.71(38.04 to 87.04) | 77.08(48.81 to 112.8) | 9.58(5.48 to 13.53) | 12.02(6.83 to 17.04) | 17.82(12.06 to 24.44) | 21.08(14.02 to 29.43) |
| Sierra Leone | 106033.69(19940.68 to 178494) | 1256.13(247.45 to 2102.5) | 89486.69(48036.87 to 136014.6) | 660.92(361.91 to 1003.56) | 43950.97(14116.99 to 69542.58) | 504.32(163.12 to 807.43) | 30696.16(20955.63 to 42720.89) | 222.86(153.2 to 309.11) | 25305.56(5738.94 to 47374.2) | 283.93(63.9 to 531.57) | 22253.56(13189.69 to 32179.71) | 159.47(94.62 to 229.98) | 7693.74(1646.73 to 25756.49) | 97.79(23.7 to 312.76) | 10204.25(4872.87 to 24461.42) | 80.76(40.57 to 183.95) |
| Singapore | 10712.41(9212.61 to 11728.11) | 435.74(372.94 to 479.04) | 2209.31(1643.65 to 2816.55) | 63.94(47.09 to 83.08) | 1394.33(1187.23 to 1713.94) | 56.21(47.89 to 69.69) | 606.03(461.83 to 774.04) | 16.78(12.4 to 21.23) | 1288(1018.84 to 1627.26) | 53.6(42.42 to 67.57) | 332.94(255.75 to 460.25) | 10.81(8.11 to 15.57) | 624.58(506.65 to 777.51) | 23.18(19.09 to 29.16) | 342.98(254.24 to 465.48) | 8.52(6.38 to 11.48) |
| Slovakia | 14926.34(12397.03 to 17187.29) | 368.77(305.93 to 427.19) | 4311.53(3503.67 to 5451.78) | 141.83(114.72 to 180.41) | 1462.46(1169.48 to 1787.66) | 36.27(28.93 to 44.7) | 358.84(283.08 to 457.28) | 10.64(8.34 to 13.54) | 2195.34(1619.67 to 3030.96) | 56.3(41.37 to 78) | 761.46(497.11 to 1200.65) | 27.05(17.55 to 43.09) | 522.6(421.83 to 647.88) | 11.75(9.46 to 14.49) | 621.15(481.91 to 797.67) | 17.64(13.56 to 23.05) |
| Slovenia | 3952.1(3079.01 to 4440.09) | 325.16(251.43 to 365.05) | 712.93(549.34 to 863.17) | 63.02(47.92 to 76.5) | 453.94(381.76 to 531.55) | 37.31(31.31 to 44.21) | 94.34(68.98 to 127.31) | 6.33(4.81 to 8.37) | 596.84(482.45 to 707.7) | 53.3(42.8 to 63.16) | 93.46(69.62 to 140.6) | 9.25(6.69 to 14.39) | 380.71(327.94 to 439.85) | 26.5(22.69 to 30.69) | 282.63(221.39 to 342.56) | 20.63(15.75 to 25.42) |
| Solomon Islands | 3745.66(1908.57 to 5326.23) | 626.26(331.16 to 877.79) | 3579.12(2605.96 to 4794.82) | 389.37(284.77 to 511.76) | 676.48(361.06 to 1116.46) | 106.41(57.16 to 175.2) | 627.82(293.03 to 1071.67) | 65.23(30.59 to 110.64) | 131.17(54.8 to 313.52) | 21.03(9.07 to 48.89) | 148.62(68.15 to 456.45) | 15.73(7.35 to 47.15) | 367.86(132.69 to 746.91) | 63.83(25.22 to 124.16) | 590.2(305.84 to 909.88) | 64.89(34.23 to 98.4) |
| Somalia | 104009.98(24946.28 to 223798.03) | 628.71(158 to 1346.46) | 172341.05(66782.17 to 361408.12) | 407.24(166.24 to 846.27) | 60110.62(32705.64 to 103502.24) | 352.06(193.88 to 607.29) | 99985.24(52517.01 to 167252.33) | 229.67(121.38 to 384.01) | 20144.6(4523.41 to 45445.56) | 116.25(27.05 to 261.26) | 35223.56(11985.03 to 69628.17) | 79.64(28.06 to 156.72) | 13405.04(1776.28 to 50498.2) | 85.61(13.17 to 320.61) | 20015.65(5246.58 to 61639.93) | 50.47(15.25 to 150.36) |
| South Africa | 121443.61(99910.23 to 157620.25) | 252.49(208.09 to 326.28) | 88994.09(61493.12 to 124818.51) | 179.51(123.37 to 254.56) | 34838.25(27201.61 to 44688.11) | 71.69(56.07 to 91.62) | 20286.22(15307.09 to 27686.58) | 40.95(30.65 to 56.46) | 32321.25(23087.1 to 41983.51) | 65.89(47.19 to 85.64) | 22516.3(13927.46 to 32872.65) | 46.66(28.68 to 68.44) | 22138.09(17365.91 to 31271.54) | 46.24(36.46 to 65.15) | 22535.86(14299.89 to 30578.17) | 45.56(28.58 to 62.28) |
| South Sudan | 82031.73(19562.24 to 167380.78) | 740.29(183.45 to 1499.5) | 111890.88(42471.45 to 199116.05) | 665.83(257.83 to 1185.27) | 45455.77(23247.33 to 77198.77) | 399.84(202.08 to 685.99) | 60631.99(33396.58 to 100145.12) | 351.62(194.14 to 579.97) | 18044.51(3394.11 to 40902.53) | 157.82(30.36 to 359.45) | 27640.89(7463.82 to 54706.79) | 159.2(43.69 to 316.63) | 9291.32(1445.09 to 36653.67) | 87.26(14.69 to 340.7) | 16625.25(4019.81 to 55038.5) | 102.34(25.95 to 333.45) |
| Spain | 75455.6(65974.41 to 83416.51) | 337.02(293.03 to 375.96) | 21389.26(17422.63 to 26215.28) | 90.44(72.67 to 107.96) | 8981.78(7985.84 to 10397.18) | 40.6(35.86 to 47.87) | 2293.71(1809.25 to 2894.49) | 9.04(6.86 to 11.46) | 8815.83(7592.01 to 11686.68) | 43.2(37.21 to 57.65) | 2820.87(2093.88 to 3684.81) | 14.72(10.55 to 19.25) | 8731.83(7459.33 to 10157.7) | 30.35(26.19 to 35.16) | 9947.09(8585.66 to 11935.65) | 26.33(21.26 to 33.63) |
| Sri Lanka | 84686.91(55316.34 to 114288.63) | 470.54(307.83 to 639.85) | 39006(28644.02 to 53180.1) | 235.52(172.66 to 324.25) | 5907.31(3983.38 to 8514.56) | 33.5(22.47 to 48.48) | 2262.34(1687.32 to 3029.25) | 13.56(10 to 18.45) | 8978.1(5444.42 to 14419.47) | 51.33(31.08 to 82.8) | 5136.2(3078.06 to 8220.86) | 33.64(19.79 to 54.63) | 2257.1(1656.87 to 3260.63) | 12.29(9 to 17.6) | 2071.37(1462.51 to 2754.7) | 11.63(8.02 to 15.69) |
| Sudan | 1037173.26(302390.21 to 1723574.19) | 2712.55(791.15 to 4535.25) | 517688.73(342470.32 to 717065.09) | 936.59(620.61 to 1297.33) | 260823.08(100534.66 to 481895.11) | 693.61(271.21 to 1271.81) | 83297.1(55102.69 to 122566.32) | 150.92(99.84 to 222.41) | 96380.22(15333.13 to 293397.74) | 243.79(38.93 to 747.1) | 63614.37(37441.68 to 100991.17) | 115.2(67.87 to 182.74) | 43809(11136.35 to 143628.01) | 118.15(31.31 to 382.64) | 35968.97(21675.98 to 58428.07) | 65.43(39.54 to 106.02) |
| Suriname | 2214.95(1623.41 to 2819.25) | 506.4(372.03 to 643.99) | 1412.64(952.73 to 1952.97) | 314.94(210.73 to 439.51) | 677.91(489.47 to 933.24) | 154.36(111.43 to 212.43) | 361.09(245.59 to 512.7) | 80.78(54.3 to 115.58) | 942.08(508.48 to 1333.45) | 214.84(116.04 to 304.09) | 660.85(424.91 to 973.24) | 152.6(97.79 to 225.02) | 108.03(64.57 to 189.19) | 25.93(15.87 to 44.8) | 151.7(103.01 to 241.03) | 29.43(19.19 to 49.22) |
| Sweden | 15659.84(13410.49 to 17161.24) | 249.99(214.17 to 274.65) | 4423.74(3553.84 to 5692.7) | 63.12(50.46 to 83.72) | 1208.64(1031.51 to 1488.43) | 18.91(16.12 to 23.46) | 892.56(725.05 to 1090.13) | 12.47(9.85 to 15.15) | 2765.34(1884.57 to 3208.62) | 45.98(31.2 to 53.48) | 737.44(576.19 to 1004.53) | 12.06(9.12 to 16.85) | 1718.85(1490.44 to 1988.58) | 23.07(19.89 to 26.78) | 2825.75(2433.04 to 3182.82) | 29.39(25.24 to 33.56) |
| Switzerland | 16999.62(14098.32 to 19060.77) | 388.75(320.31 to 435.97) | 5220.86(4309.74 to 6329.52) | 99.88(83.63 to 119.45) | 1811.96(1557.66 to 2161.71) | 41.67(35.58 to 49.66) | 867.64(708.03 to 1047.24) | 16.71(13.7 to 20.16) | 2788.01(1797.27 to 3697.8) | 67.29(42.97 to 89.58) | 989.19(798.89 to 1212.29) | 21.41(17.26 to 26.23) | 2453.26(2089.37 to 2968.61) | 47.6(40.02 to 58.38) | 3460.34(3097.66 to 3874.67) | 50.2(43.35 to 58.15) |
| Syrian Arab Republic | 273294.92(155881.67 to 363632.51) | 1270.21(726.63 to 1689.98) | 34762.69(26001.51 to 46351.49) | 328.55(244.22 to 438.61) | 24752.68(14247.64 to 36329.72) | 116.83(68.61 to 169.82) | 4040.94(2422.6 to 6117.88) | 36.56(21.53 to 57.25) | 28016.03(10920.85 to 56683.5) | 128.67(50.5 to 259.68) | 4286.3(2622.46 to 6982.61) | 43.3(25.94 to 71.58) | 16536.45(8123.07 to 34990.2) | 79(39.5 to 164.24) | 6144.43(3812.7 to 8967.46) | 55.16(33.65 to 80.89) |
| Taiwan (Province of China) | 51792.95(48231.31 to 54951.68) | 303.83(283.74 to 322.68) | 13321.22(11364.72 to 15238.33) | 122.21(102.26 to 139.32) | 1381.63(1203.33 to 1593.59) | 7.89(6.9 to 9.02) | 385.2(292.95 to 497.38) | 2.67(2.11 to 3.3) | 6445.19(5190.93 to 7317.94) | 39.69(31.86 to 45.09) | 1715.12(1381.83 to 2142.18) | 18.29(14.69 to 23.36) | 547.15(388.33 to 754.94) | 2.72(1.97 to 3.71) | 1566.02(1070.32 to 1979.93) | 14.93(9.51 to 19.26) |
| Tajikistan | 28835.58(22226.52 to 41471.8) | 308.48(238.39 to 438.32) | 35988.58(21788.79 to 73038.72) | 270.09(164.37 to 544.42) | 5935.96(4381.03 to 7638.09) | 61.69(45.89 to 79) | 7095.06(3784.82 to 12085.79) | 53.18(28.87 to 90.04) | 5022.58(2804.36 to 7930.91) | 51.4(28.86 to 81.09) | 6760.32(3454.34 to 12152.23) | 50.04(25.66 to 89.65) | 5765.9(2950.72 to 13117.72) | 60.73(31.58 to 137.57) | 6516.76(3697.99 to 12492.67) | 48.68(27.74 to 92.94) |
| Thailand | 241545.11(172118.73 to 338059.08) | 466.52(330.54 to 659.07) | 63263.08(48178.02 to 77577.41) | 197.21(144.15 to 245.88) | 32075.43(22395.19 to 47109.39) | 61.93(42.65 to 92.05) | 5599.57(4233.25 to 7178.07) | 15.46(11.1 to 20.29) | 22080.96(12113.59 to 36303.56) | 43.42(23.67 to 71.41) | 6277.94(3911.57 to 10773.96) | 21.55(12.83 to 38.5) | 5244.45(3502.6 to 9389.32) | 9.5(6.22 to 17.22) | 4361.62(3243.25 to 5438.71) | 11.6(8.31 to 14.6) |
| Timor-Leste | 19014.89(5348.37 to 30141.92) | 1258.57(367.11 to 1976.91) | 11699.08(8342.6 to 16366.46) | 617.7(446.77 to 859.1) | 2797.51(1286.35 to 4788.02) | 175.38(81.08 to 299.66) | 1706.4(1055.63 to 2598.14) | 88.28(54.94 to 133.95) | 1896.7(488.73 to 4643.44) | 121.03(30.67 to 295.79) | 1451.19(880.48 to 2269.62) | 75.46(45.87 to 117.84) | 472.29(115.47 to 1672.56) | 32.95(8.58 to 113.46) | 404.31(255.52 to 645.87) | 21.93(14.13 to 34.48) |
| Togo | 44333.35(12767.84 to 66043.76) | 645.48(199.41 to 951.12) | 40395.16(27042.59 to 56642.78) | 360.21(242.45 to 503.68) | 19800.11(10236.41 to 28021.01) | 277.42(145.27 to 392.28) | 15552.69(10550.52 to 22750.44) | 136.36(92.38 to 198.25) | 9937.25(4210.95 to 14478.35) | 136.34(58.31 to 199.34) | 10056.76(6426.48 to 16377.04) | 87.67(55.81 to 142.68) | 4041.56(1214.07 to 11879.25) | 66.21(21.82 to 181.72) | 7162.2(3775.19 to 14882.34) | 69.57(37.43 to 139.36) |
| Tokelau | 8.14(4.95 to 10.72) | 445.19(274.29 to 583.45) | 11.6(8.37 to 16.48) | 1181.48(842.16 to 1714.99) | 1.15(0.64 to 1.77) | 63.89(35.16 to 98.19) | 1.05(0.54 to 1.72) | 116(58.76 to 193.65) | 0.33(0.16 to 1.02) | 18.47(9.09 to 57.25) | 0.5(0.18 to 1.89) | 57.14(19.78 to 216.01) | 1.55(0.52 to 2.69) | 83.63(29.61 to 142.59) | 3.23(1.69 to 4.87) | 329.15(171.44 to 500.56) |
| Tonga | 385.3(257.9 to 529.13) | 258.78(178.18 to 352.92) | 219.54(156.19 to 328.69) | 161.93(115.71 to 239.7) | 67.69(41.7 to 104.6) | 44.18(27.47 to 67.84) | 34.55(19.58 to 55.32) | 24.71(14.12 to 39.3) | 20.61(10.88 to 61.67) | 13.68(7.45 to 39.94) | 14.07(6.49 to 49.85) | 10.25(4.88 to 35.16) | 55.58(26.37 to 101.58) | 37.48(18.65 to 67.19) | 46.76(26.29 to 79.12) | 33.68(19.4 to 56.56) |
| Trinidad and Tobago | 6100.85(5225.52 to 7151.28) | 506.94(434.12 to 595.74) | 3333.77(2537.76 to 4373.32) | 410.77(309.69 to 541.3) | 639.09(521.55 to 777.14) | 53.64(43.62 to 65.54) | 215.19(162.99 to 282.59) | 25.91(19.13 to 34.45) | 1254.54(966.67 to 1556.51) | 106.98(82.17 to 132.84) | 632.62(460.61 to 849.77) | 84.75(61.09 to 114.5) | 501.3(425.35 to 592.8) | 42.1(35.78 to 49.75) | 720.45(511.48 to 965.9) | 63.95(45.85 to 85.57) |
| Tunisia | 147676.58(70033.81 to 206548.95) | 1407.37(668.81 to 1966.63) | 25680.39(19231.24 to 34210.76) | 296.3(222.3 to 393.36) | 15686.19(6828.15 to 25548.8) | 149.6(65.58 to 243.19) | 2426.2(1701.18 to 3445.98) | 27.1(18.47 to 38.81) | 16044.96(6269.88 to 30491.23) | 152.84(59.79 to 290.38) | 3789.88(2510.8 to 5623.21) | 45.6(29.95 to 67.81) | 7474.11(3539.93 to 15824.89) | 71.6(33.97 to 151.1) | 3782.54(2463.93 to 5509.82) | 41.81(26.89 to 62.39) |
| Turkey | 1356705.56(610151.86 to 2080646.48) | 1935.1(871.82 to 2966.89) | 176352.65(135605.57 to 220180.19) | 330.78(250.79 to 420.93) | 208014.07(82095.86 to 336679.58) | 296.29(117.39 to 479.85) | 26628.75(20939.58 to 33869.63) | 48.52(37.06 to 62.49) | 215682.8(61843.98 to 415654.41) | 306.79(88.05 to 591.42) | 38875.23(27927.07 to 53281.8) | 76.43(54.43 to 105.8) | 87803.19(36143.57 to 192401.06) | 126.13(52.08 to 275.94) | 26500.8(20992.56 to 33974.04) | 47.12(36.55 to 61.55) |
| Turkmenistan | 25162.49(19519.83 to 31783.32) | 436.74(339.51 to 547.65) | 30408.65(22052.35 to 39442.06) | 570.58(413.9 to 740.07) | 3877.39(2908.8 to 5148.81) | 66.06(49.69 to 87.61) | 2699.4(1806.53 to 4097.02) | 50.83(34.02 to 77.15) | 5802.64(3876.04 to 7827.37) | 97.84(65.62 to 131.93) | 7325.16(4362.22 to 10737.65) | 137.87(82.18 to 202.11) | 1327.58(561.2 to 2132.31) | 23.21(10.17 to 36.81) | 1272.6(780.29 to 1874.75) | 23.83(14.63 to 35.08) |
| Tuvalu | 150.07(44.64 to 232.71) | 978.61(309.5 to 1502.66) | 40.67(29.07 to 57.04) | 316.37(226.1 to 444.3) | 24.09(9.9 to 38.91) | 149.8(62.24 to 240.68) | 6.27(3.83 to 9.51) | 48.93(29.91 to 74.18) | 5.36(1.74 to 11.46) | 33.43(11.24 to 70.72) | 2(0.97 to 5.63) | 15.6(7.61 to 43.91) | 18.09(3.68 to 45.91) | 121.44(26.61 to 300.5) | 6.98(3.78 to 10.76) | 54.56(29.5 to 84.02) |
| Uganda | 210211.61(59975.59 to 407523.65) | 531.53(157.09 to 1017.94) | 232399.99(146590.39 to 382890.28) | 321.75(207.61 to 531.15) | 119339.06(69220.49 to 188350.74) | 291.93(170.69 to 464.74) | 138101.74(88441.7 to 206858.34) | 187.45(120.31 to 281.2) | 50068.23(10321.43 to 104090.44) | 120.96(25.88 to 252.61) | 72308.47(34607.03 to 114331.19) | 97.4(47.61 to 153.65) | 19417.16(4867.53 to 66930.35) | 51.41(14.64 to 173.32) | 32333.64(19066.44 to 61285.19) | 46.25(27.95 to 85.61) |
| Ukraine | 197601.06(172738.9 to 229670.81) | 562.81(488.01 to 657.72) | 45606.37(38177.53 to 54418.86) | 239.71(197.69 to 289.25) | 47594.97(39547.26 to 56885.52) | 138.75(114.76 to 166.79) | 7467.57(5974.5 to 9476.99) | 38.81(30.12 to 51.56) | 32463.67(24457.36 to 40874.48) | 97.89(73.47 to 123.83) | 5056.65(3616.65 to 7194.65) | 33.56(23.74 to 47.8) | 5344.42(4283.35 to 7690.26) | 13.91(11.17 to 20.37) | 3056.81(2288.11 to 4591.22) | 13.73(10.2 to 21.22) |
| United Arab Emirates | 15929.46(10607.84 to 21596.46) | 703.08(467.75 to 950.25) | 7320.18(4690.92 to 9682.47) | 158.17(98.65 to 212.53) | 2364.97(1358.33 to 3439.32) | 104.9(60.8 to 152.33) | 1060.66(661.26 to 1503.73) | 21.58(12.35 to 31.7) | 1999.07(1091.01 to 3482.58) | 86.7(47.55 to 150.78) | 986.86(521.16 to 1661.71) | 24.07(12.23 to 41.18) | 656.81(421.75 to 924.12) | 29.72(19.16 to 41.32) | 2104.76(1124.66 to 3806.16) | 41.52(22.11 to 75.6) |
| United Kingdom | 106742.45(100032.71 to 113743.14) | 253.35(238.13 to 270.58) | 42253.23(36526.7 to 50212.56) | 98.42(84.8 to 117.09) | 14282.07(13330.44 to 15932.77) | 34.14(31.87 to 38.18) | 6648.15(5866.96 to 7468.24) | 15.99(13.92 to 17.95) | 15195.7(13332.19 to 21243.3) | 38.36(33.64 to 53.76) | 8466.55(7203.99 to 10411.21) | 22.81(19.26 to 28.59) | 4565.9(3395.42 to 6106.99) | 8.91(6.56 to 11.93) | 18187.78(14494.98 to 20129.25) | 27.98(21.49 to 31.18) |
| United Republic of Tanzania | 391459.81(104051.43 to 742263.38) | 753.28(210.93 to 1425.83) | 337861.78(199132.46 to 588423.02) | 385.94(232.16 to 670.41) | 228265.61(132376.52 to 350725.99) | 425.43(246.81 to 649.14) | 208554.85(132595.7 to 310296.68) | 234.83(149.61 to 349.67) | 107183.76(23613.63 to 227367.89) | 197.94(44.36 to 418.81) | 113518.55(56035.22 to 175943.88) | 127.27(63.28 to 196.97) | 45673.84(10553.71 to 160769.28) | 90.73(23.35 to 312) | 60414.97(31548.51 to 123969.01) | 70.22(37.07 to 141.97) |
| United States of America | 507423.49(447904.58 to 548719.76) | 242.12(212.6 to 262.18) | 220211.58(192475.49 to 258675.57) | 101.57(88.58 to 120.58) | 74785.47(69116 to 86523.57) | 36.56(33.78 to 42.39) | 45065.22(38220.8 to 51409.36) | 21.51(17.83 to 24.73) | 64248.74(58641.99 to 80751.12) | 31.54(28.78 to 39.69) | 42453.37(34070.84 to 49300.62) | 21.98(17.42 to 25.82) | 32600.75(28292.03 to 38422.86) | 14.3(12.46 to 16.82) | 68748.86(60362.85 to 75996.08) | 21.13(18.84 to 23.27) |
| United States Virgin Islands | 410.62(321.62 to 499.22) | 369.25(289.62 to 448.73) | 61.85(42.78 to 93.42) | 134.87(89.42 to 214.81) | 70.25(49.72 to 93.8) | 63.16(44.71 to 84.34) | 6.69(4.48 to 9.85) | 14.36(9.03 to 22.55) | 56.36(33.31 to 92.42) | 50.57(29.91 to 82.92) | 7.6(4.03 to 16.57) | 19(9.34 to 43.69) | 26.43(20.52 to 34.07) | 24.02(18.63 to 30.95) | 14.63(10.34 to 20.18) | 23.29(15.59 to 33.91) |
| Uruguay | 11660.94(9625.53 to 13684) | 427.03(352 to 501.64) | 4652.79(3759.83 to 5628.53) | 237.18(188.06 to 292.25) | 2081.06(1766 to 2523.2) | 75.97(64.31 to 92.29) | 627.47(502.38 to 789.12) | 29.14(22.94 to 37.93) | 2643.33(2025.5 to 3264.29) | 97.8(74.84 to 120.8) | 992.17(765.11 to 1271.5) | 55.2(42.04 to 71.41) | 2129.12(1781.89 to 2572.63) | 76.72(64.12 to 93.08) | 1396.93(1204.58 to 1694.03) | 58.79(48.28 to 74.18) |
| Uzbekistan | 107724.43(89648.97 to 124665.1) | 329.28(274.94 to 380) | 208874.5(152088.5 to 268732.52) | 548.53(400.19 to 704.41) | 35380.5(26781.46 to 45573.35) | 105.81(80.48 to 135.64) | 8485.36(5507.57 to 16477.41) | 22.62(14.82 to 43.44) | 10499.25(7356.23 to 18792.71) | 31.39(22.13 to 56.09) | 14825.28(9440.28 to 23983.63) | 38.76(24.77 to 62.53) | 3271.85(1936.66 to 4651.02) | 10.22(6.27 to 14.32) | 2058.75(1356.15 to 4114.62) | 5.52(3.66 to 10.88) |
| Vanuatu | 1269.16(564.08 to 1778.61) | 478(224.89 to 659.2) | 1309.21(868.64 to 1740.64) | 326.09(220.85 to 429.12) | 212.79(116.03 to 353.4) | 75.02(41.57 to 123.24) | 214.37(119.13 to 366.52) | 51.57(28.82 to 88.04) | 49.17(20.3 to 124.43) | 17.66(7.61 to 43.42) | 57.47(26.73 to 155.95) | 14.04(6.64 to 37.37) | 117.74(45.28 to 222.58) | 45.95(19.26 to 83.19) | 172.52(86.26 to 278.63) | 43.34(21.84 to 68.72) |
| Venezuela (Bolivarian Republic of) | 98614.96(89108.98 to 108111.11) | 387.24(350.6 to 423.74) | 92061.92(65478.7 to 122173.93) | 411.18(291.16 to 547.32) | 20344.8(15533.9 to 24809.47) | 78.64(60.27 to 95.87) | 9948.28(6989 to 13991.37) | 44.59(31.03 to 62.83) | 24542.6(20118.71 to 29473.29) | 93.99(77.18 to 112.77) | 31909.62(22637.86 to 43210.37) | 145.4(103.07 to 197.15) | 9037.63(7658.19 to 10419.91) | 36.82(31.41 to 42.27) | 14057.54(9383.09 to 19222.55) | 60.46(40.39 to 82.93) |
| Viet Nam | 302593.87(134718.01 to 412532.76) | 330.82(149.73 to 449.94) | 120456.65(83323.87 to 170783.6) | 151.31(104.44 to 215.92) | 47232.63(24943.53 to 71652.15) | 51.46(27.47 to 77.66) | 14833.34(9121.02 to 24996.44) | 19.14(11.46 to 32.76) | 34840.05(14794.68 to 54532.38) | 37.73(16.14 to 58.92) | 17874.2(10582.05 to 28401.34) | 23.15(13.53 to 36.94) | 5821.13(3108.7 to 8770.22) | 6.52(3.55 to 9.77) | 4865.59(2565.94 to 8077.01) | 5.84(2.96 to 9.98) |
| Yemen | 721303.69(209823.58 to 1163465.43) | 2443.08(724.69 to 3949.24) | 470727.42(309137.49 to 639256.07) | 1017(671.07 to 1380.82) | 145686.01(60036.61 to 266712.13) | 498.2(209.66 to 908.79) | 76305.95(45279.34 to 129816.05) | 165.46(98.46 to 281.66) | 58693.8(12823.66 to 154097.03) | 194.74(42.72 to 515.11) | 49467.66(26497.11 to 83049.29) | 106.54(57.16 to 178.66) | 33209.87(6628.51 to 108465.07) | 116.16(24.58 to 371.29) | 38197.66(17387.33 to 82082.44) | 83.42(38.45 to 177.86) |
| Zambia | 113187.15(32597.64 to 216579.08) | 693.66(208 to 1318.45) | 89642.63(55170.89 to 155429.98) | 315.86(195.37 to 546.54) | 62535.67(31444.29 to 100237.61) | 375.06(187.9 to 602.95) | 53056.79(28667.22 to 89540.28) | 185.05(100.39 to 312.06) | 31505.94(6952.24 to 65937.04) | 185.15(41.95 to 389.82) | 30463.64(18146.14 to 51253.07) | 105.88(63.08 to 177.66) | 14002.03(3090.42 to 51357.94) | 89.7(21.96 to 323.03) | 17217.35(9869.39 to 29523.54) | 62.12(35.81 to 105.68) |
| Zimbabwe | 35732.92(26169.96 to 46456.98) | 206.67(150.91 to 264.85) | 49616.81(33692.16 to 68988.75) | 233.33(158.89 to 323.1) | 6362.33(3953.23 to 9119.84) | 36.69(22.91 to 52.61) | 9661.08(6112.8 to 13874.73) | 45.3(28.76 to 65.26) | 7641.5(3679.3 to 13140.63) | 42.62(20.87 to 72.38) | 10894.43(4641.34 to 19810.72) | 49.89(21.39 to 90.2) | 6119.52(4008.14 to 8708.29) | 36.12(24.05 to 50.41) | 11471.44(6722.14 to 17193.88) | 54.63(32.15 to 80.29) |

## Table S4 Age-standardized DALYs rates of CHD, NTDs, DCAs, and DS with slope index of inequality analysis, categorized by global, SDI, and GBD regions

| **Region** | **Cause** | **Year** | **lm** | **lm_lower** | **lm_upper** | **ncvTest_p** | **rlm** | **rlm_lower** | **rlm_upper** |
| --- | --- | --- | --- | --- | --- | --- | --- | --- | --- |
| All included | Digestive congenital anomalies | 1990 | -110.1789177 | -132.5745348 | -87.78330057 | 0.000154221 | -110.4827779 | -130.2491965 | -90.71635932 |
| All included | Neural tube defects | 1990 | -327.4421441 | -365.034325 | -289.8499632 | 1.89193E-21 | -302.2813695 | -333.6730231 | -270.8897158 |
| All included | Congenital heart anomalies | 1990 | -703.82441 | -862.0277028 | -545.6211173 | 8.84315E-17 | -524.6138348 | -614.1427574 | -435.0849122 |
| All included | Down syndrome | 1990 | -54.36435513 | -65.79558806 | -42.9331222 | 0.000526054 | -54.14809618 | -64.13509852 | -44.16109385 |
| All included | Digestive congenital anomalies | 2021 | -80.19531545 | -93.04811646 | -67.34251444 | 8.8177E-08 | -80.36586088 | -91.11916095 | -69.61256081 |
| All included | Neural tube defects | 2021 | -156.3572578 | -174.772173 | -137.9423426 | 1.1744E-19 | -139.1932972 | -155.5094873 | -122.877107 |
| All included | Congenital heart anomalies | 2021 | -384.0587733 | -459.0761373 | -309.0414094 | 1.22324E-07 | -330.5563396 | -377.4824904 | -283.6301889 |
| All included | Down syndrome | 2021 | -33.29451476 | -46.84288271 | -19.74614681 | 0.173810013 | -37.88752996 | -45.72492957 | -30.05013035 |
| Low SDI | Digestive congenital anomalies | 1990 | -62.25426943 | -141.3780808 | 16.86954197 | 0.451227539 | -57.56447657 | -134.7085474 | 19.57959427 |
| Low SDI | Neural tube defects | 1990 | -124.8527484 | -279.9050266 | 30.19952986 | 0.793731202 | -123.4610678 | -287.8470139 | 40.92487822 |
| Low SDI | Congenital heart anomalies | 1990 | -152.7251766 | -837.5396436 | 532.0892904 | 0.557664109 | -124.079898 | -513.4859708 | 265.3261749 |
| Low SDI | Down syndrome | 1990 | -4.629582491 | -40.37080417 | 31.11163919 | 0.871271048 | -2.431744189 | -38.77184749 | 33.90835911 |
| Low SDI | Digestive congenital anomalies | 2021 | -45.9242782 | -88.22283949 | -3.625716901 | 0.231729076 | -49.45346613 | -95.08513679 | -3.821795475 |
| Low SDI | Neural tube defects | 2021 | -112.8937587 | -176.2805834 | -49.50693406 | 0.716840035 | -98.67307337 | -151.0787597 | -46.26738709 |
| Low SDI | Congenital heart anomalies | 2021 | -64.65238285 | -369.3791562 | 240.0743905 | 0.214040911 | -167.4207238 | -368.0770417 | 33.23559403 |
| Low SDI | Down syndrome | 2021 | -8.477011665 | -34.31654905 | 17.36252572 | 0.043792051 | -5.96987684 | -25.96043976 | 14.02068608 |
| Low-middle SDI | Neural tube defects | 1990 | -176.439697 | -257.7655945 | -95.11379947 | 0.002345314 | -152.7578916 | -228.7467566 | -76.76902673 |
| Low-middle SDI | Congenital heart anomalies | 1990 | -479.9902986 | -851.5850418 | -108.3955553 | 0.015615918 | -324.6069407 | -551.5692344 | -97.64464709 |
| Low-middle SDI | Down syndrome | 1990 | 4.033034499 | -19.31776193 | 27.38383093 | 0.552189848 | 0.984101612 | -19.83100529 | 21.79920851 |
| Low-middle SDI | Digestive congenital anomalies | 1990 | -63.46890921 | -112.0851125 | -14.85270591 | 0.07492878 | -77.81802959 | -118.3955135 | -37.24054567 |
| Low-middle SDI | Neural tube defects | 2021 | -68.07528287 | -105.6976037 | -30.45296205 | 0.017154938 | -60.01521065 | -96.31005691 | -23.7203644 |
| Low-middle SDI | Congenital heart anomalies | 2021 | -92.04519318 | -249.0525426 | 64.96215627 | 0.158660559 | -44.26451375 | -138.0149272 | 49.48589967 |
| Low-middle SDI | Down syndrome | 2021 | -1.049158173 | -18.94158026 | 16.84326391 | 0.449079977 | -0.057923476 | -17.60534589 | 17.48949894 |
| Low-middle SDI | Digestive congenital anomalies | 2021 | -10.95431772 | -45.01617771 | 23.10754228 | 0.352484523 | -17.61377893 | -51.09238057 | 15.8648227 |
| Middle SDI | Neural tube defects | 1990 | -60.01350068 | -120.7007111 | 0.673709692 | 0.002331822 | -33.00231064 | -81.45500479 | 15.45038351 |
| Middle SDI | Congenital heart anomalies | 1990 | -294.5707785 | -590.1839245 | 1.042367577 | 0.001522657 | -108.5572868 | -297.0640339 | 79.94946035 |
| Middle SDI | Down syndrome | 1990 | -16.23190418 | -41.22318222 | 8.759373868 | 0.273704611 | -14.61654105 | -38.94014864 | 9.707066531 |
| Middle SDI | Digestive congenital anomalies | 1990 | -14.08889954 | -59.2814198 | 31.10362072 | 0.565980457 | -18.81643205 | -69.21214942 | 31.57928532 |
| Middle SDI | Neural tube defects | 2021 | -8.976150049 | -29.24588371 | 11.29358361 | 0.785706416 | -10.1645239 | -25.89441032 | 5.565362531 |
| Middle SDI | Congenital heart anomalies | 2021 | 117.5669991 | -27.62602692 | 262.7600251 | 0.000813105 | 66.37678075 | -34.87424171 | 167.6278032 |
| Middle SDI | Down syndrome | 2021 | 9.61657903 | -33.06591293 | 52.29907099 | 0.000128245 | -10.4605794 | -22.8247786 | 1.903619811 |
| Middle SDI | Digestive congenital anomalies | 2021 | 3.922575453 | -22.19805145 | 30.04320235 | 0.793146902 | 5.067594775 | -18.94116412 | 29.07635367 |
| High-middle SDI | Down syndrome | 1990 | -11.07623239 | -54.97151468 | 32.81904991 | 0.043613668 | -3.202653779 | -36.63660708 | 30.23129952 |
| High-middle SDI | Digestive congenital anomalies | 1990 | -59.56979658 | -155.4059941 | 36.26640091 | 0.040009357 | -53.54599075 | -132.5713851 | 25.47940363 |
| High-middle SDI | Neural tube defects | 1990 | -345.0067205 | -442.3692446 | -247.6441965 | 8.54158E-05 | -242.9591839 | -325.32073 | -160.5976378 |
| High-middle SDI | Congenital heart anomalies | 1990 | -707.5327866 | -1207.278756 | -207.7868172 | 0.004256185 | -572.6683904 | -897.2663751 | -248.0704057 |
| High-middle SDI | Down syndrome | 2021 | -7.805473398 | -58.95620951 | 43.34526272 | 0.915965085 | -11.75045914 | -36.00115783 | 12.50023955 |
| High-middle SDI | Digestive congenital anomalies | 2021 | -43.19424672 | -75.02485171 | -11.36364172 | 0.767265804 | -44.03701067 | -73.86486498 | -14.20915635 |
| High-middle SDI | Neural tube defects | 2021 | -10.38431701 | -44.98433809 | 24.21570408 | 0.369176267 | -15.20198855 | -36.82236823 | 6.418391143 |
| High-middle SDI | Congenital heart anomalies | 2021 | -177.7620369 | -391.6046245 | 36.08055059 | 0.930524563 | -179.5697964 | -301.4180668 | -57.72152591 |
| High SDI | Neural tube defects | 1990 | -47.59886817 | -75.4914111 | -19.70632523 | 0.056259796 | -47.47749882 | -75.48483326 | -19.47016437 |
| High SDI | Congenital heart anomalies | 1990 | -237.95102 | -382.0059651 | -93.89607495 | 0.010181741 | -179.8427199 | -304.2235754 | -55.46186431 |
| High SDI | Down syndrome | 1990 | -11.22711054 | -31.2475013 | 8.793280212 | 0.001919574 | -2.966281032 | -14.89843323 | 8.965871168 |
| High SDI | Digestive congenital anomalies | 1990 | -16.96636516 | -33.52977382 | -0.402956495 | 0.50924185 | -14.78859844 | -30.14145205 | 0.564255175 |
| High SDI | Neural tube defects | 2021 | -1.485290543 | -7.109507846 | 4.13892676 | 0.783973036 | -0.941658734 | -6.060480114 | 4.177162646 |
| High SDI | Congenital heart anomalies | 2021 | -33.06288476 | -65.80029911 | -0.325470407 | 0.05035062 | -22.77805579 | -52.06859761 | 6.512486041 |
| High SDI | Down syndrome | 2021 | 5.687184301 | -3.907286013 | 15.28165462 | 0.168462712 | 4.930360787 | -5.653351076 | 15.51407265 |
| High SDI | Digestive congenital anomalies | 2021 | -5.097130647 | -13.47964302 | 3.285381726 | 0.007307792 | -1.93514018 | -7.741185616 | 3.870905255 |
| Eastern Sub-Saharan Africa | Digestive congenital anomalies | 1990 | -16.98191818 | -86.92556667 | 52.96173031 | 0.978786455 | -16.98191818 | -86.92556667 | 52.96173031 |
| Eastern Sub-Saharan Africa | Neural tube defects | 1990 | -144.0613042 | -313.1372118 | 25.01460342 | 0.60858261 | -142.9722363 | -322.7986704 | 36.85419784 |
| Eastern Sub-Saharan Africa | Congenital heart anomalies | 1990 | -296.9608736 | -568.8569625 | -25.0647847 | 0.708977064 | -283.8330474 | -592.2243199 | 24.55822514 |
| Eastern Sub-Saharan Africa | Down syndrome | 1990 | -22.27061055 | -63.77034096 | 19.22911986 | 0.96604375 | -17.22165686 | -60.17705837 | 25.73374465 |
| Eastern Sub-Saharan Africa | Digestive congenital anomalies | 2021 | -21.00737897 | -62.05473608 | 20.03997814 | 0.217926583 | -12.0897433 | -47.78914923 | 23.60966263 |
| Eastern Sub-Saharan Africa | Neural tube defects | 2021 | -111.245347 | -187.161495 | -35.32919894 | 0.301854531 | -91.30169303 | -166.2057444 | -16.39764171 |
| Eastern Sub-Saharan Africa | Congenital heart anomalies | 2021 | -224.0016721 | -364.0121778 | -83.99116642 | 0.030006124 | -158.3022864 | -261.4483786 | -55.15619421 |
| Eastern Sub-Saharan Africa | Down syndrome | 2021 | -12.57980488 | -38.38854024 | 13.22893049 | 0.197856318 | 2.081137258 | -15.45743369 | 19.61970821 |
| Western Sub-Saharan Africa | Neural tube defects | 1990 | -186.8018689 | -351.3387878 | -22.26494989 | 0.12680512 | -188.262184 | -396.2450172 | 19.72064911 |
| Western Sub-Saharan Africa | Congenital heart anomalies | 1990 | -568.6431904 | -941.3088124 | -195.9775685 | 0.148584928 | -506.513414 | -945.2007301 | -67.82609789 |
| Western Sub-Saharan Africa | Down syndrome | 1990 | -29.45981209 | -62.15707231 | 3.237448135 | 0.147198346 | -29.17278842 | -63.89720243 | 5.551625577 |
| Western Sub-Saharan Africa | Digestive congenital anomalies | 1990 | -80.87812156 | -169.669374 | 7.913130896 | 0.132445438 | -68.72165216 | -157.9506468 | 20.50734252 |
| Western Sub-Saharan Africa | Neural tube defects | 2021 | -119.6139682 | -197.0993092 | -42.12862725 | 0.978766303 | -126.2859599 | -208.2547586 | -44.31716109 |
| Western Sub-Saharan Africa | Congenital heart anomalies | 2021 | -384.3676108 | -574.1007281 | -194.6344935 | 0.854101075 | -392.6331161 | -597.646346 | -187.6198862 |
| Western Sub-Saharan Africa | Down syndrome | 2021 | -32.92649525 | -53.70446039 | -12.14853012 | 0.82465351 | -35.86047918 | -56.87667933 | -14.84427903 |
| Western Sub-Saharan Africa | Digestive congenital anomalies | 2021 | -60.94649525 | -116.9461153 | -4.946875194 | 0.809791921 | -63.33576859 | -120.6742722 | -5.99726499 |
| Central Sub-Saharan Africa | Digestive congenital anomalies | 1990 | -32.75619337 | -75.44919929 | 9.936812543 | 0.373367405 | -32.75619337 | -75.44919929 | 9.936812543 |
| Central Sub-Saharan Africa | Neural tube defects | 1990 | -187.0622298 | -326.7081059 | -47.41635356 | 0.508754161 | -187.0622298 | -326.7081059 | -47.41635356 |
| Central Sub-Saharan Africa | Congenital heart anomalies | 1990 | -291.1974926 | -493.8405204 | -88.55446474 | 0.306763916 | -317.9598575 | -519.122898 | -116.796817 |
| Central Sub-Saharan Africa | Down syndrome | 1990 | -25.10857666 | -51.99346294 | 1.776309613 | 0.967794272 | -25.10857666 | -51.99346294 | 1.776309613 |
| Central Sub-Saharan Africa | Digestive congenital anomalies | 2021 | -30.8601225 | -64.02032081 | 2.300075815 | 0.256011465 | -33.49918024 | -69.66616594 | 2.667805452 |
| Central Sub-Saharan Africa | Neural tube defects | 2021 | -197.4454376 | -274.8532163 | -120.0376589 | 0.155359588 | -213.4533196 | -270.7460699 | -156.1605693 |
| Central Sub-Saharan Africa | Congenital heart anomalies | 2021 | -279.9868976 | -434.1568838 | -125.8169114 | 0.172000006 | -318.3907836 | -453.5104115 | -183.2711558 |
| Central Sub-Saharan Africa | Down syndrome | 2021 | -32.28583361 | -52.47329241 | -12.0983748 | 0.861032673 | -32.71271061 | -66.88255829 | 1.457137079 |
| North Africa and Middle East | Digestive congenital anomalies | 1990 | -63.59813678 | -144.4695582 | 17.27328465 | 0.735381275 | -75.79966402 | -149.7650867 | -1.834241316 |
| North Africa and Middle East | Neural tube defects | 1990 | -319.7660659 | -500.3296698 | -139.202462 | 0.00140196 | -374.6808598 | -535.1753259 | -214.1863937 |
| North Africa and Middle East | Congenital heart anomalies | 1990 | -1244.29688 | -1929.539529 | -559.0542311 | 0.018980331 | -1313.033429 | -2117.005397 | -509.0614605 |
| North Africa and Middle East | Down syndrome | 1990 | -41.80487756 | -89.4280713 | 5.818316182 | 0.696503847 | -42.2320677 | -91.74719026 | 7.28305485 |
| North Africa and Middle East | Digestive congenital anomalies | 2021 | -59.64843803 | -99.45431854 | -19.84255752 | 0.494166653 | -66.69659566 | -104.6653375 | -28.72785379 |
| North Africa and Middle East | Neural tube defects | 2021 | -94.95212186 | -156.6850368 | -33.21920694 | 0.151360808 | -101.6438221 | -166.8408301 | -36.44681417 |
| North Africa and Middle East | Congenital heart anomalies | 2021 | -732.438239 | -1053.693952 | -411.1825257 | 0.002918459 | -672.1423368 | -959.2871406 | -384.997533 |
| North Africa and Middle East | Down syndrome | 2021 | -30.25045403 | -66.78582259 | 6.284914532 | 0.59067261 | -37.04468734 | -65.43284815 | -8.656526534 |
| Oceania | Down syndrome | 1990 | -43.29662768 | -102.7396894 | 16.14643403 | 0.582175256 | -43.02928616 | -105.0778999 | 19.01932759 |
| Oceania | Digestive congenital anomalies | 1990 | -11.24732975 | -25.67512595 | 3.180466448 | 0.683653172 | -11.24732975 | -25.67512595 | 3.180466448 |
| Oceania | Neural tube defects | 1990 | -110.5649301 | -178.5199904 | -42.60986984 | 0.077395182 | -108.4517158 | -181.9976322 | -34.90579936 |
| Oceania | Congenital heart anomalies | 1990 | -590.8932498 | -1042.747057 | -139.0394421 | 0.108206366 | -576.7809546 | -1071.800545 | -81.76136456 |
| Oceania | Down syndrome | 2021 | -13.06424994 | -294.8735252 | 268.7450253 | 0.123400051 | -112.949045 | -221.5787132 | -4.319376833 |
| Oceania | Digestive congenital anomalies | 2021 | -6.586293227 | -60.18862158 | 47.01603513 | 0.109648005 | -26.55993527 | -50.73438556 | -2.385484986 |
| Oceania | Neural tube defects | 2021 | -165.5320372 | -268.4515752 | -62.61249925 | 0.160747421 | -193.055135 | -267.4107736 | -118.6994965 |
| Oceania | Congenital heart anomalies | 2021 | -653.558239 | -1719.792454 | 412.6759758 | 0.115522901 | -1037.646392 | -1573.511329 | -501.7814536 |
| South Asia | Neural tube defects | 1990 | -31.8785982 | -223.2181528 | 159.4609564 | 0.527308549 | -31.8785982 | -223.2181528 | 159.4609564 |
| South Asia | Congenital heart anomalies | 1990 | -143.250026 | -955.7820318 | 669.2819798 | 0.410775296 | -107.1927479 | -1060.339803 | 845.9543077 |
| South Asia | Down syndrome | 1990 | 6.557214568 | -10.33623752 | 23.45066666 | 0.549813892 | 6.557214568 | -10.33623752 | 23.45066666 |
| South Asia | Digestive congenital anomalies | 1990 | 8.178612919 | -73.27399705 | 89.63122288 | 0.330865077 | 0.667774247 | -98.20446028 | 99.54000877 |
| South Asia | Neural tube defects | 2021 | 13.48137096 | -42.42900873 | 69.39175065 | 0.346851844 | 6.894641301 | -22.01792872 | 35.80721132 |
| South Asia | Congenital heart anomalies | 2021 | 113.0126311 | -187.733873 | 413.7591352 | 0.468297039 | 55.3905715 | -224.8169536 | 335.5980966 |
| South Asia | Down syndrome | 2021 | 4.933013424 | -20.18803887 | 30.05406571 | 0.466275958 | -0.089909233 | -21.72833423 | 21.54851576 |
| South Asia | Digestive congenital anomalies | 2021 | 31.67643361 | -14.68801154 | 78.04087877 | 0.430757928 | 31.67643361 | -14.68801154 | 78.04087877 |
| Caribbean | Digestive congenital anomalies | 1990 | -122.0310297 | -199.8711738 | -44.1908856 | 0.00662062 | -114.7274588 | -190.708223 | -38.74669455 |
| Caribbean | Neural tube defects | 1990 | -128.0620619 | -214.1112347 | -42.01288911 | 4.67354E-05 | -66.1612361 | -108.1108645 | -24.21160769 |
| Caribbean | Congenital heart anomalies | 1990 | -619.0738807 | -1092.052295 | -146.0954667 | 3.13486E-06 | -229.2694049 | -369.4868916 | -89.05191831 |
| Caribbean | Down syndrome | 1990 | -42.90286801 | -77.10314657 | -8.702589445 | 5.36531E-05 | -16.20818072 | -27.2519187 | -5.164442744 |
| Caribbean | Digestive congenital anomalies | 2021 | -112.9308206 | -170.7947223 | -55.06691904 | 0.188412726 | -110.0179903 | -171.6573263 | -48.37865422 |
| Caribbean | Neural tube defects | 2021 | -86.99872113 | -136.7377632 | -37.25967902 | 1.39589E-05 | -41.29293234 | -77.25790263 | -5.327962049 |
| Caribbean | Congenital heart anomalies | 2021 | -462.6926335 | -811.7764215 | -113.6088454 | 5.0735E-06 | -136.0942022 | -330.3461708 | 58.15776637 |
| Caribbean | Down syndrome | 2021 | -31.50570723 | -65.22534501 | 2.213930559 | 0.003104002 | -11.16901666 | -20.3227164 | -2.015316919 |
| Southeast Asia | Neural tube defects | 1990 | -147.9396243 | -218.2477248 | -77.63152389 | 0.056125224 | -155.6408517 | -234.7656972 | -76.51600615 |
| Southeast Asia | Congenital heart anomalies | 1990 | -976.3478978 | -1466.135846 | -486.5599496 | 0.053445274 | -1052.478339 | -1606.48236 | -498.4743171 |
| Southeast Asia | Down syndrome | 1990 | -25.16513085 | -38.58779718 | -11.74246453 | 0.27116339 | -27.51649335 | -39.31522156 | -15.71776514 |
| Southeast Asia | Digestive congenital anomalies | 1990 | -94.37541115 | -142.6075634 | -46.1432589 | 0.19316645 | -100.7213794 | -141.7453394 | -59.69741944 |
| Southeast Asia | Neural tube defects | 2021 | -72.02376904 | -113.8695502 | -30.17798793 | 0.180630906 | -76.81268469 | -120.3380383 | -33.28733103 |
| Southeast Asia | Congenital heart anomalies | 2021 | -428.8800576 | -701.0570901 | -156.7030251 | 0.108327708 | -456.9341217 | -740.0560668 | -173.8121765 |
| Southeast Asia | Down syndrome | 2021 | -12.67096579 | -25.12846411 | -0.213467469 | 0.134793712 | -11.31012143 | -20.80808107 | -1.812161789 |
| Southeast Asia | Digestive congenital anomalies | 2021 | -52.08080852 | -92.16842011 | -11.99319694 | 0.545226002 | -54.1181781 | -94.76052424 | -13.47583197 |
| Southern Sub-Saharan Africa | Digestive congenital anomalies | 1990 | 31.93127643 | 0.219020341 | 63.64353252 | 0.798898406 | 31.93127643 | 0.219020341 | 63.64353252 |
| Southern Sub-Saharan Africa | Neural tube defects | 1990 | 2.15677442 | -75.35222379 | 79.66577263 | 0.162640238 | -3.479283384 | -63.8641878 | 56.90562104 |
| Southern Sub-Saharan Africa | Congenital heart anomalies | 1990 | 7.991767846 | -114.1894436 | 130.1729793 | 0.405099019 | 7.991767846 | -114.1894436 | 130.1729793 |
| Southern Sub-Saharan Africa | Down syndrome | 1990 | 4.837524262 | -14.84776949 | 24.52281801 | 0.259705021 | 4.837524262 | -14.84776949 | 24.52281801 |
| Southern Sub-Saharan Africa | Digestive congenital anomalies | 2021 | -8.852709949 | -43.36962979 | 25.6642099 | 0.655128292 | -8.852709949 | -43.36962979 | 25.6642099 |
| Southern Sub-Saharan Africa | Neural tube defects | 2021 | -22.38188332 | -91.03435993 | 46.2705933 | 0.432718611 | -12.78031482 | -51.62742008 | 26.06679044 |
| Southern Sub-Saharan Africa | Congenital heart anomalies | 2021 | -98.78137473 | -278.1929761 | 80.63022667 | 0.655846221 | -93.64690774 | -302.6603776 | 115.3665621 |
| Southern Sub-Saharan Africa | Down syndrome | 2021 | -17.45476342 | -46.92565952 | 12.01613269 | 0.652915789 | -16.57158458 | -57.90644733 | 24.76327816 |
| Central Latin America | Neural tube defects | 1990 | -68.09588377 | -167.4084196 | 31.21665206 | 0.325302406 | -63.467132 | -189.1200896 | 62.18582563 |
| Central Latin America | Congenital heart anomalies | 1990 | -157.8006669 | -554.8946737 | 239.2933399 | 0.146646718 | -237.3551039 | -594.0141201 | 119.3039123 |
| Central Latin America | Down syndrome | 1990 | 3.790477775 | -11.48669625 | 19.0676518 | 0.304501527 | 4.477116585 | -14.25725984 | 23.21149301 |
| Central Latin America | Digestive congenital anomalies | 1990 | -5.078610877 | -95.68959526 | 85.53237351 | 0.071613252 | 13.35604236 | -68.21860517 | 94.93068988 |
| Central Latin America | Neural tube defects | 2021 | -18.23474618 | -40.92830129 | 4.458808941 | 0.770936598 | -18.23130568 | -37.13925777 | 0.676646416 |
| Central Latin America | Congenital heart anomalies | 2021 | 87.93831847 | -53.54825881 | 229.4248958 | 0.08981706 | 111.0889528 | -29.34418598 | 251.5220916 |
| Central Latin America | Down syndrome | 2021 | 4.926977284 | -24.76951949 | 34.62347406 | 0.238141008 | 5.336131475 | -27.81953854 | 38.49180149 |
| Central Latin America | Digestive congenital anomalies | 2021 | 6.386126098 | -54.31352399 | 67.08577619 | 0.300182846 | 4.978600111 | -49.02852552 | 58.98572574 |
| Central Asia | Neural tube defects | 1990 | 10.05869693 | -85.64576496 | 105.7631588 | 0.83223875 | 9.440191807 | -66.27831956 | 85.15870318 |
| Central Asia | Congenital heart anomalies | 1990 | -7.096540007 | -381.2106225 | 367.0175424 | 0.867274016 | -7.096540007 | -381.2106225 | 367.0175424 |
| Central Asia | Down syndrome | 1990 | -63.70454426 | -109.6631197 | -17.7459688 | 0.06347902 | -55.45868207 | -81.71530696 | -29.20205717 |
| Central Asia | Digestive congenital anomalies | 1990 | -56.51093754 | -200.3925653 | 87.3706902 | 0.086232717 | -37.88823033 | -238.1134359 | 162.3369752 |
| Central Asia | Neural tube defects | 2021 | -14.63436456 | -49.95858964 | 20.68986053 | 0.745612724 | -15.65302812 | -62.49788889 | 31.19183265 |
| Central Asia | Congenital heart anomalies | 2021 | -9.160357553 | -271.4582747 | 253.1375596 | 0.973353664 | -9.027389826 | -302.1230585 | 284.0682788 |
| Central Asia | Down syndrome | 2021 | -23.27901546 | -50.96310403 | 4.405073121 | 0.046858907 | -26.85505502 | -62.3534399 | 8.643329846 |
| Central Asia | Digestive congenital anomalies | 2021 | -5.350709672 | -79.18074697 | 68.47932763 | 0.755560319 | -7.324766332 | -80.42725138 | 65.77771871 |
| East Asia | Neural tube defects | 1990 | -197.2773655 | -386.0042068 | -8.550524297 | 0.38829262 | -197.2773655 | -386.0042068 | -8.550524297 |
| East Asia | Congenital heart anomalies | 1990 | -996.1474217 | -2195.141883 | 202.8470394 | 0.38829262 | -996.1474217 | -2195.141883 | 202.8470394 |
| East Asia | Down syndrome | 1990 | -89.66525595 | -163.2036966 | -16.12681531 | 0.38829262 | -89.66525595 | -163.2036966 | -16.12681531 |
| East Asia | Digestive congenital anomalies | 1990 | -101.2850488 | -277.119397 | 74.54929932 | 0.38829262 | -101.2850488 | -277.119397 | 74.54929932 |
| East Asia | Neural tube defects | 2021 | -21.73106755 | -29.75291857 | -13.70921652 | 0.998861019 | -21.73112765 | -39.29020662 | -4.172048684 |
| East Asia | Congenital heart anomalies | 2021 | -172.685053 | -252.4990253 | -92.87108077 | 0.998861019 | -172.685651 | -347.3909451 | 2.019642975 |
| East Asia | Down syndrome | 2021 | -1.232745723 | -19.09686888 | 16.63137743 | 0.998861019 | -1.232879574 | -40.33576845 | 37.8700093 |
| East Asia | Digestive congenital anomalies | 2021 | -24.5360994 | -47.69008955 | -1.382109241 | 0.998861019 | -24.53627288 | -75.21818413 | 26.14563836 |
| Andean Latin America | Down syndrome | 1990 | -94.44570837 | -206.6786479 | 17.7872312 | 0.928057696 | -94.44570837 | -206.6786479 | 17.7872312 |
| Andean Latin America | Digestive congenital anomalies | 1990 | -272.4015384 | -322.3691899 | -222.433887 | 0.928057696 | -272.4015384 | -322.3691899 | -222.433887 |
| Andean Latin America | Neural tube defects | 1990 | -217.1091384 | -237.7260846 | -196.4921922 | 0.928057696 | -217.1091384 | -237.7260846 | -196.4921922 |
| Andean Latin America | Congenital heart anomalies | 1990 | -1380.503073 | -1980.126692 | -780.8794532 | 0.928057696 | -1380.503073 | -1980.126692 | -780.8794532 |
| Andean Latin America | Down syndrome | 2021 | -64.33418471 | -133.7112756 | 5.042906218 | 0.678667513 | -64.33418471 | -133.7112756 | 5.042906218 |
| Andean Latin America | Digestive congenital anomalies | 2021 | -152.0438038 | -264.620315 | -39.4672927 | 0.678667513 | -152.0438038 | -264.620315 | -39.4672927 |
| Andean Latin America | Neural tube defects | 2021 | -53.8011009 | -172.7103597 | 65.10815791 | 0.678667513 | -53.8011009 | -172.7103597 | 65.10815791 |
| Andean Latin America | Congenital heart anomalies | 2021 | -549.1381383 | -1035.230739 | -63.04553798 | 0.678667513 | -549.1381383 | -1035.230739 | -63.04553798 |
| Central Europe | Digestive congenital anomalies | 1990 | -28.52813794 | -91.12935157 | 34.07307569 | 0.006895725 | -42.24054565 | -94.9717539 | 10.49066259 |
| Central Europe | Neural tube defects | 1990 | -81.00244056 | -128.7720286 | -33.23285255 | 0.040399195 | -95.16114421 | -135.1401205 | -55.18216796 |
| Central Europe | Congenital heart anomalies | 1990 | -209.5314348 | -466.4969787 | 47.43410901 | 0.150645504 | -292.1953187 | -521.4081105 | -62.98252688 |
| Central Europe | Down syndrome | 1990 | 12.16460139 | -8.029694733 | 32.35889751 | 0.72194526 | 12.14950744 | -9.908117813 | 34.20713269 |
| Central Europe | Digestive congenital anomalies | 2021 | -17.57341523 | -35.18510831 | 0.038277859 | 0.264315497 | -15.19240159 | -30.24599943 | -0.138803745 |
| Central Europe | Neural tube defects | 2021 | -18.22440325 | -32.13327853 | -4.315527977 | 0.041407957 | -12.06566054 | -19.41565638 | -4.715664692 |
| Central Europe | Congenital heart anomalies | 2021 | -119.8784138 | -209.9572567 | -29.79957083 | 0.130721543 | -113.9338612 | -207.5166926 | -20.3510297 |
| Central Europe | Down syndrome | 2021 | 5.051899164 | -8.059892962 | 18.16369129 | 0.917008251 | 5.443432083 | -8.8771374 | 19.76400157 |
| Eastern Europe | Neural tube defects | 1990 | -55.95689843 | -110.7121616 | -1.201635247 | 0.519588246 | -49.21521081 | -100.7610288 | 2.330607202 |
| Eastern Europe | Congenital heart anomalies | 1990 | -145.8047588 | -291.1758349 | -0.4336826 | 0.20593619 | -159.4719549 | -319.5538184 | 0.609908599 |
| Eastern Europe | Down syndrome | 1990 | 45.95021063 | 4.208093953 | 87.69232731 | 0.262397735 | 45.95021063 | 4.208093953 | 87.69232731 |
| Eastern Europe | Digestive congenital anomalies | 1990 | -76.31097582 | -147.1048032 | -5.517148411 | 0.157222208 | -54.8076135 | -101.6834056 | -7.931821438 |
| Eastern Europe | Neural tube defects | 2021 | -20.11093179 | -35.31201446 | -4.909849113 | 0.15615998 | -17.80613066 | -34.85407642 | -0.758184907 |
| Eastern Europe | Congenital heart anomalies | 2021 | -141.2266745 | -230.574111 | -51.87923801 | 0.247102825 | -169.548589 | -225.3404451 | -113.7567329 |
| Eastern Europe | Down syndrome | 2021 | 0.54410898 | -14.08387231 | 15.17209027 | 0.635191616 | 2.176528664 | -14.14747634 | 18.50053367 |
| Eastern Europe | Digestive congenital anomalies | 2021 | -47.30762488 | -79.86898269 | -14.74626706 | 0.058665452 | -37.81341021 | -64.47694838 | -11.14987205 |
| Southern Latin America | Digestive congenital anomalies | 1990 | -5.111305889 | -53.72881435 | 43.50620257 | 0.531926786 | -5.111305889 | -53.72881435 | 43.50620257 |
| Southern Latin America | Neural tube defects | 1990 | 65.14864443 | -33.70910885 | 164.0063977 | 0.531926786 | 65.14864443 | -33.70910885 | 164.0063977 |
| Southern Latin America | Congenital heart anomalies | 1990 | 65.63197684 | -403.7169523 | 534.980906 | 0.531926786 | 65.63197684 | -403.7169523 | 534.980906 |
| Southern Latin America | Down syndrome | 1990 | -48.85283035 | -83.3930883 | -14.31257239 | 0.531926786 | -48.85283035 | -83.3930883 | -14.31257239 |
| Southern Latin America | Digestive congenital anomalies | 2021 | -20.70877486 | -71.07545378 | 29.65790405 | 0.840141185 | -20.70877486 | -71.07545378 | 29.65790405 |
| Southern Latin America | Neural tube defects | 2021 | 9.05228064 | -31.93854228 | 50.04310356 | 0.840141185 | 9.05228064 | -31.93854228 | 50.04310356 |
| Southern Latin America | Congenital heart anomalies | 2021 | -137.0366707 | -323.4282558 | 49.35491441 | 0.840141185 | -137.0366707 | -323.4282558 | 49.35491441 |
| Southern Latin America | Down syndrome | 2021 | -29.20052192 | -43.01522019 | -15.38582365 | 0.840141185 | -29.20052192 | -43.01522019 | -15.38582365 |
| Western Europe | Neural tube defects | 1990 | -39.67104253 | -73.6993533 | -5.642731757 | 0.077995029 | -32.90313002 | -61.36473553 | -4.441524516 |
| Western Europe | Congenital heart anomalies | 1990 | -111.8587054 | -218.1927989 | -5.52461195 | 0.504038925 | -106.0305577 | -184.6735633 | -27.38755221 |
| Western Europe | Down syndrome | 1990 | -5.69163397 | -19.43542904 | 8.052161102 | 0.048435342 | -7.981359566 | -23.4609508 | 7.498231665 |
| Western Europe | Digestive congenital anomalies | 1990 | -8.674385708 | -27.48898286 | 10.14021144 | 0.565099628 | -10.17920845 | -27.8830143 | 7.524597407 |
| Western Europe | Neural tube defects | 2021 | -2.170615656 | -12.04965318 | 7.708421864 | 0.101283386 | 0.524932592 | -6.904088407 | 7.953953591 |
| Western Europe | Congenital heart anomalies | 2021 | -20.39081937 | -53.77268314 | 12.9910444 | 0.656732214 | -15.93025184 | -51.9221729 | 20.06166922 |
| Western Europe | Down syndrome | 2021 | 4.658539232 | -10.21938808 | 19.53646654 | 0.288710328 | 5.401988623 | -10.67145234 | 21.47542958 |
| Western Europe | Digestive congenital anomalies | 2021 | -3.273058876 | -11.87244625 | 5.326328493 | 0.184251278 | 0.327130048 | -6.874418061 | 7.528678157 |
| High-income North America | Neural tube defects | 1990 | -54.57438727 | -130.6382937 | 21.48951913 | 0.939368683 | -54.57438727 | -130.6382937 | 21.48951913 |
| High-income North America | Congenital heart anomalies | 1990 | -130.4595221 | -317.1972493 | 56.27820514 | 0.939368683 | -130.4595221 | -317.1972493 | 56.27820514 |
| High-income North America | Down syndrome | 1990 | 1.43536355 | -21.33064447 | 24.20137157 | 0.939368683 | 1.43536355 | -21.33064447 | 24.20137157 |
| High-income North America | Digestive congenital anomalies | 1990 | -49.44876549 | -116.035691 | 17.13816005 | 0.939368683 | -49.44876549 | -116.035691 | 17.13816005 |
| High-income North America | Neural tube defects | 2021 | -11.71263686 | -13.38765514 | -10.03761857 | 0.936466285 | -11.71263686 | -13.38765514 | -10.03761857 |
| High-income North America | Congenital heart anomalies | 2021 | -5.600509116 | -20.41472863 | 9.213710396 | 0.936466285 | -5.600509116 | -20.41472863 | 9.213710396 |
| High-income North America | Down syndrome | 2021 | 11.13715726 | -1.111977844 | 23.38629236 | 0.936466285 | 11.13715726 | -1.111977844 | 23.38629236 |
| High-income North America | Digestive congenital anomalies | 2021 | -12.89351066 | -20.69688197 | -5.090139353 | 0.936466285 | -12.89351066 | -20.69688197 | -5.090139353 |
| High-income Asia Pacific | Neural tube defects | 1990 | -82.00313785 | -184.8326413 | 20.82636556 | 0.630502834 | -82.20625562 | -246.203306 | 81.79079475 |
| High-income Asia Pacific | Congenital heart anomalies | 1990 | -169.0743052 | -329.5795889 | -8.569021627 | 0.419842267 | -169.0743052 | -329.5795889 | -8.569021627 |
| High-income Asia Pacific | Down syndrome | 1990 | -21.09053832 | -83.96780922 | 41.78673258 | 0.384198172 | -21.09053832 | -83.96780922 | 41.78673258 |
| High-income Asia Pacific | Digestive congenital anomalies | 1990 | -7.148854107 | -35.05228804 | 20.75457983 | 0.293830361 | -6.180846838 | -47.286351 | 34.92465732 |
| High-income Asia Pacific | Neural tube defects | 2021 | -36.74506311 | -97.62346254 | 24.13333632 | 0.351808448 | -36.74506311 | -97.62346254 | 24.13333632 |
| High-income Asia Pacific | Congenital heart anomalies | 2021 | -90.0094741 | -326.5205814 | 146.5016332 | 0.248145437 | -90.0094741 | -326.5205814 | 146.5016332 |
| High-income Asia Pacific | Down syndrome | 2021 | -37.22837848 | -139.6857892 | 65.22903222 | 0.269306359 | -37.22837848 | -139.6857892 | 65.22903222 |
| High-income Asia Pacific | Digestive congenital anomalies | 2021 | -12.63943986 | -46.69693538 | 21.41805566 | 0.232638686 | -12.63943986 | -46.69693538 | 21.41805566 |
| Sub-Saharan Africa | Digestive congenital anomalies | 1990 | -126.598635 | -174.3859185 | -78.81135146 | 0.073697227 | -123.1538681 | -168.4028081 | -77.9049281 |
| Sub-Saharan Africa | Neural tube defects | 1990 | -328.6340576 | -427.2402297 | -230.0278854 | 0.112379336 | -340.3792727 | -446.0099394 | -234.7486061 |
| Sub-Saharan Africa | Congenital heart anomalies | 1990 | -721.0560368 | -913.1288871 | -528.9831866 | 0.006944888 | -681.0898378 | -859.4190703 | -502.7606052 |
| Sub-Saharan Africa | Down syndrome | 1990 | -49.43930953 | -67.63929986 | -31.23931919 | 0.018142323 | -51.54015881 | -68.46375057 | -34.61656706 |
| Sub-Saharan Africa | Digestive congenital anomalies | 2021 | -63.64197429 | -90.23518552 | -37.04876306 | 0.327495343 | -63.55995769 | -90.98170917 | -36.13820622 |
| Sub-Saharan Africa | Neural tube defects | 2021 | -164.0659877 | -210.4415612 | -117.6904143 | 0.234426754 | -163.6614193 | -206.3471963 | -120.9756422 |
| Sub-Saharan Africa | Congenital heart anomalies | 2021 | -365.2877314 | -454.6942968 | -275.8811661 | 0.019032766 | -353.6587959 | -440.5409735 | -266.7766183 |
| Sub-Saharan Africa | Down syndrome | 2021 | -28.74772441 | -40.25125242 | -17.24419639 | 0.084882858 | -28.49805089 | -40.54387859 | -16.45222319 |
| North Africa and Middle East | Digestive congenital anomalies | 1990 | -63.59813678 | -144.4695582 | 17.27328465 | 0.735381275 | -75.79966402 | -149.7650867 | -1.834241316 |
| North Africa and Middle East | Neural tube defects | 1990 | -319.7660659 | -500.3296698 | -139.202462 | 0.00140196 | -374.6808598 | -535.1753259 | -214.1863937 |
| North Africa and Middle East | Congenital heart anomalies | 1990 | -1244.29688 | -1929.539529 | -559.0542311 | 0.018980331 | -1313.033429 | -2117.005397 | -509.0614605 |
| North Africa and Middle East | Down syndrome | 1990 | -41.80487756 | -89.4280713 | 5.818316182 | 0.696503847 | -42.2320677 | -91.74719026 | 7.28305485 |
| North Africa and Middle East | Digestive congenital anomalies | 2021 | -59.64843803 | -99.45431854 | -19.84255752 | 0.494166653 | -66.69659566 | -104.6653375 | -28.72785379 |
| North Africa and Middle East | Neural tube defects | 2021 | -94.95212186 | -156.6850368 | -33.21920694 | 0.151360808 | -101.6438221 | -166.8408301 | -36.44681417 |
| North Africa and Middle East | Congenital heart anomalies | 2021 | -732.438239 | -1053.693952 | -411.1825257 | 0.002918459 | -672.1423368 | -959.2871406 | -384.997533 |
| North Africa and Middle East | Down syndrome | 2021 | -30.25045403 | -66.78582259 | 6.284914532 | 0.59067261 | -37.04468734 | -65.43284815 | -8.656526534 |
| Southeast Asia, east Asia, and Oceania | Neural tube defects | 1990 | -105.1970378 | -138.8434437 | -71.55063193 | 8.68003E-05 | -96.94386648 | -123.3144986 | -70.57323437 |
| Southeast Asia, east Asia, and Oceania | Congenital heart anomalies | 1990 | -618.4654193 | -859.7214218 | -377.2094167 | 6.87414E-05 | -553.1232485 | -762.9937127 | -343.2527842 |
| Southeast Asia, east Asia, and Oceania | Down syndrome | 1990 | -19.97918135 | -43.46347887 | 3.505116171 | 0.29369054 | -16.01267874 | -39.20458465 | 7.17922718 |
| Southeast Asia, east Asia, and Oceania | Digestive congenital anomalies | 1990 | -50.85700367 | -84.39786575 | -17.3161416 | 0.00072057 | -38.3180747 | -76.55030642 | -0.085842974 |
| Southeast Asia, east Asia, and Oceania | Neural tube defects | 2021 | -34.3852484 | -65.06189776 | -3.708599035 | 0.374559348 | -32.15889825 | -61.66371759 | -2.654078922 |
| Southeast Asia, east Asia, and Oceania | Congenital heart anomalies | 2021 | -199.8558723 | -428.2029596 | 28.49121498 | 0.701911285 | -221.7517661 | -396.7999048 | -46.70362749 |
| Southeast Asia, east Asia, and Oceania | Down syndrome | 2021 | 4.922587874 | -52.08925831 | 61.93443406 | 0.225540805 | -7.592493243 | -36.25561459 | 21.0706281 |
| Southeast Asia, east Asia, and Oceania | Digestive congenital anomalies | 2021 | -20.38696717 | -46.43952513 | 5.665590786 | 0.105722916 | -15.59458458 | -38.48603879 | 7.296869622 |
| South Asia | Neural tube defects | 1990 | -31.8785982 | -223.2181528 | 159.4609564 | 0.527308549 | -31.8785982 | -223.2181528 | 159.4609564 |
| South Asia | Congenital heart anomalies | 1990 | -143.250026 | -955.7820318 | 669.2819798 | 0.410775296 | -107.1927479 | -1060.339803 | 845.9543077 |
| South Asia | Down syndrome | 1990 | 6.557214568 | -10.33623752 | 23.45066666 | 0.549813892 | 6.557214568 | -10.33623752 | 23.45066666 |
| South Asia | Digestive congenital anomalies | 1990 | 8.178612919 | -73.27399705 | 89.63122288 | 0.330865077 | 0.667774247 | -98.20446028 | 99.54000877 |
| South Asia | Neural tube defects | 2021 | 13.48137096 | -42.42900873 | 69.39175065 | 0.346851844 | 6.894641301 | -22.01792872 | 35.80721132 |
| South Asia | Congenital heart anomalies | 2021 | 113.0126311 | -187.733873 | 413.7591352 | 0.468297039 | 55.3905715 | -224.8169536 | 335.5980966 |
| South Asia | Down syndrome | 2021 | 4.933013424 | -20.18803887 | 30.05406571 | 0.466275958 | -0.089909233 | -21.72833423 | 21.54851576 |
| South Asia | Digestive congenital anomalies | 2021 | 31.67643361 | -14.68801154 | 78.04087877 | 0.430757928 | 31.67643361 | -14.68801154 | 78.04087877 |
| Latin America and Caribbean | Digestive congenital anomalies | 1990 | -57.37289078 | -105.8266053 | -8.919176217 | 0.002306812 | -40.52898429 | -84.80490182 | 3.746933249 |
| Latin America and Caribbean | Neural tube defects | 1990 | -83.6457653 | -132.8489445 | -34.44258609 | 0.002826561 | -68.2747785 | -114.5264118 | -22.02314516 |
| Latin America and Caribbean | Congenital heart anomalies | 1990 | -363.3328712 | -617.0752851 | -109.5904572 | 3.11952E-06 | -206.8583758 | -333.924561 | -79.7921907 |
| Latin America and Caribbean | Down syndrome | 1990 | -15.58400083 | -38.97895323 | 7.81095157 | 0.001148132 | -5.067680343 | -17.92012084 | 7.78476015 |
| Latin America and Caribbean | Digestive congenital anomalies | 2021 | -52.45525178 | -80.36400218 | -24.54650138 | 0.009793589 | -49.61892297 | -78.04988189 | -21.18796405 |
| Latin America and Caribbean | Neural tube defects | 2021 | -38.61409015 | -56.88566539 | -20.34251491 | 0.000129061 | -29.67178393 | -43.84485588 | -15.49871197 |
| Latin America and Caribbean | Congenital heart anomalies | 2021 | -160.7258768 | -297.3230365 | -24.12871707 | 0.000267082 | -90.8657012 | -173.3305147 | -8.400887725 |
| Latin America and Caribbean | Down syndrome | 2021 | -7.153750141 | -21.87478358 | 7.567283294 | 0.00136662 | -2.034439927 | -13.29922296 | 9.230343107 |
| Central Europe, eastern Europe, and central Asia | Neural tube defects | 1990 | -61.2342159 | -104.3470825 | -18.12134929 | 0.501088003 | -58.55803686 | -101.0077802 | -16.10829351 |
| Central Europe, eastern Europe, and central Asia | Congenital heart anomalies | 1990 | -167.8464993 | -328.886419 | -6.806579733 | 0.127206892 | -184.8030027 | -361.9491186 | -7.656886732 |
| Central Europe, eastern Europe, and central Asia | Down syndrome | 1990 | 13.50796119 | -12.86829847 | 39.88422084 | 0.97850878 | 13.46355663 | -6.024813595 | 32.95192686 |
| Central Europe, eastern Europe, and central Asia | Digestive congenital anomalies | 1990 | -55.87674345 | -107.913818 | -3.839668917 | 0.002151762 | -48.80474544 | -89.8950805 | -7.714410372 |
| Central Europe, eastern Europe, and central Asia | Neural tube defects | 2021 | -27.59918954 | -40.46102812 | -14.73735096 | 0.015894899 | -22.99772062 | -35.55587983 | -10.43956142 |
| Central Europe, eastern Europe, and central Asia | Congenital heart anomalies | 2021 | -285.9040761 | -398.3980483 | -173.4101039 | 0.027818251 | -250.070578 | -343.6293435 | -156.5118125 |
| Central Europe, eastern Europe, and central Asia | Down syndrome | 2021 | -2.828919667 | -13.96118066 | 8.303341321 | 0.00978894 | 1.537725851 | -7.843972017 | 10.91942372 |
| Central Europe, eastern Europe, and central Asia | Digestive congenital anomalies | 2021 | -61.23164964 | -89.52035869 | -32.9429406 | 0.008212896 | -49.27839122 | -73.12597917 | -25.43080327 |
| High income | Neural tube defects | 1990 | -30.13526885 | -55.99753872 | -4.272998975 | 0.103385927 | -26.16889985 | -49.77392482 | -2.563874871 |
| High income | Congenital heart anomalies | 1990 | -96.76042692 | -180.0638882 | -13.45696562 | 0.767520224 | -99.98615899 | -194.9481782 | -5.024139759 |
| High income | Down syndrome | 1990 | -3.803863741 | -14.13544056 | 6.527713081 | 0.443977283 | -4.722061243 | -15.99780772 | 6.553685233 |
| High income | Digestive congenital anomalies | 1990 | -5.276701349 | -19.63085012 | 9.077447424 | 0.428774496 | -5.69030554 | -16.81060891 | 5.429997831 |
| High income | Neural tube defects | 2021 | -6.126072927 | -15.54143579 | 3.28928993 | 0.008579025 | -2.752263531 | -8.765936354 | 3.261409291 |
| High income | Congenital heart anomalies | 2021 | -27.19650201 | -57.97221104 | 3.579207015 | 0.060193551 | -13.83278347 | -34.45214592 | 6.786578991 |
| High income | Down syndrome | 2021 | -2.435026153 | -15.86407157 | 10.99401927 | 0.515896816 | -0.450622205 | -13.41404085 | 12.51279644 |
| High income | Digestive congenital anomalies | 2021 | -5.145314393 | -11.47341248 | 1.182783688 | 0.075657331 | -2.427988838 | -7.942615874 | 3.086638198 |

## Table S5 Age-standardized DALYs rates of CHD, NTDs, DCAs, and DS with concentration index analysis, categorized by global, SDI, and GBD regions

| **Region** | **Cause** | **Year** | **Concentration_index** | **UCI** | **LCI** | **SE** | **P** |
| --- | --- | --- | --- | --- | --- | --- | --- |
| All included | Congenital heart anomalies | 1990 | -0.13805713 | -0.098031147 | -0.178083113 | 0.02042142 | 0 |
| All included | Congenital heart anomalies | 2021 | -0.20983911 | -0.17545334 | -0.24422488 | 0.01754376 | 0 |
| All included | Digestive congenital anomalies | 1990 | -0.12444251 | -0.090759028 | -0.158125992 | 0.01718545 | 0 |
| All included | Digestive congenital anomalies | 2021 | -0.23958092 | -0.206271053 | -0.272890787 | 0.01699483 | 0 |
| All included | Down syndrome | 1990 | -0.10946469 | -0.061163842 | -0.157765538 | 0.02464329 | 0 |
| All included | Down syndrome | 2021 | -0.15430011 | -0.108537971 | -0.200062249 | 0.02334803 | 0 |
| All included | Neural tube defects | 1990 | -0.2867794 | -0.241358948 | -0.332199852 | 0.0231737 | 0 |
| All included | Neural tube defects | 2021 | -0.44384908 | -0.390498468 | -0.497199692 | 0.0272197 | 0 |
| Low SDI | Congenital heart anomalies | 1990 | -0.03510007 | 0.087136878 | -0.157337018 | 0.06236579 | 0.5776 |
| Low SDI | Congenital heart anomalies | 2021 | -0.03358685 | 0.086338455 | -0.153512155 | 0.06118638 | 0.587 |
| Low SDI | Digestive congenital anomalies | 1990 | -0.01916433 | 0.043577994 | -0.081906654 | 0.03201139 | 0.5537 |
| Low SDI | Digestive congenital anomalies | 2021 | -0.03498227 | 0.034168118 | -0.104132658 | 0.03528081 | 0.3291 |
| Low SDI | Down syndrome | 1990 | -0.00596595 | 0.060714975 | -0.072646875 | 0.03402088 | 0.8619 |
| Low SDI | Down syndrome | 2021 | -0.00479384 | 0.062390903 | -0.071978583 | 0.03427793 | 0.8897 |
| Low SDI | Neural tube defects | 1990 | -0.0111025 | 0.047233646 | -0.069438646 | 0.02976334 | 0.7117 |
| Low SDI | Neural tube defects | 2021 | -0.0431358 | 0.011061022 | -0.097332622 | 0.02765144 | 0.1289 |
| Low-middle SDI | Congenital heart anomalies | 1990 | -0.05504248 | 0.024924266 | -0.135009226 | 0.04079936 | 0.1847 |
| Low-middle SDI | Congenital heart anomalies | 2021 | -0.05069937 | 0.007448636 | -0.108847376 | 0.02966735 | 0.095 |
| Low-middle SDI | Digestive congenital anomalies | 1990 | -0.04455485 | 0.012711548 | -0.101821248 | 0.02921755 | 0.135 |
| Low-middle SDI | Digestive congenital anomalies | 2021 | -0.09026337 | -0.020990835 | -0.159535905 | 0.03534313 | 0.0145 |
| Low-middle SDI | Down syndrome | 1990 | -0.00614781 | 0.085053499 | -0.097349119 | 0.04653128 | 0.8955 |
| Low-middle SDI | Down syndrome | 2021 | -0.17129127 | -0.074684556 | -0.267897984 | 0.04928914 | 0.0012 |
| Low-middle SDI | Neural tube defects | 1990 | -0.10304523 | -0.01847074 | -0.18761972 | 0.04315025 | 0.0216 |
| Low-middle SDI | Neural tube defects | 2021 | -0.20542202 | -0.117988968 | -0.292855072 | 0.0446087 | 0 |
| Middle SDI | Congenital heart anomalies | 1990 | -0.11463947 | -0.020378268 | -0.208900672 | 0.04809245 | 0.0221 |
| Middle SDI | Congenital heart anomalies | 2021 | 0.01373345 | 0.073327916 | -0.045861016 | 0.03040534 | 0.654 |
| Middle SDI | Digestive congenital anomalies | 1990 | -0.01171686 | 0.063523953 | -0.086957673 | 0.03838817 | 0.7618 |
| Middle SDI | Digestive congenital anomalies | 2021 | -0.02638845 | 0.044252145 | -0.097029045 | 0.03604112 | 0.4684 |
| Middle SDI | Down syndrome | 1990 | -0.05298304 | 0.097577065 | -0.203543145 | 0.07681638 | 0.4944 |
| Middle SDI | Down syndrome | 2021 | 0.04686113 | 0.157581275 | -0.063859015 | 0.05648987 | 0.4118 |
| Middle SDI | Neural tube defects | 1990 | -0.09610872 | 0.010848323 | -0.203065763 | 0.05456992 | 0.086 |
| Middle SDI | Neural tube defects | 2021 | -0.06785259 | 0.004524781 | -0.140229961 | 0.03692723 | 0.0738 |
| High-middle SDI | Congenital heart anomalies | 1990 | -0.11964181 | -0.07917367 | -0.16010995 | 0.02064701 | 0 |
| High-middle SDI | Congenital heart anomalies | 2021 | -0.09094218 | -0.065463964 | -0.116420396 | 0.01299909 | 0 |
| High-middle SDI | Digestive congenital anomalies | 1990 | -0.0225368 | 0.027878928 | -0.072952528 | 0.02572231 | 0.3856 |
| High-middle SDI | Digestive congenital anomalies | 2021 | -0.07943459 | -0.04272426 | -0.11614492 | 0.01872976 | 0.0001 |
| High-middle SDI | Down syndrome | 1990 | -0.08610218 | -0.040507602 | -0.131696758 | 0.02326254 | 0.0006 |
| High-middle SDI | Down syndrome | 2021 | -0.06053452 | -0.008338818 | -0.112730222 | 0.02663046 | 0.0278 |
| High-middle SDI | Neural tube defects | 1990 | -0.09874497 | -0.050305354 | -0.147184586 | 0.02471409 | 0.0002 |
| High-middle SDI | Neural tube defects | 2021 | -0.04530205 | 0.036816893 | -0.127420993 | 0.04189742 | 0.2853 |
| High SDI | Congenital heart anomalies | 1990 | -0.10051914 | -0.032055223 | -0.168983057 | 0.03493057 | 0.0065 |
| High SDI | Congenital heart anomalies | 2021 | -0.04189481 | -0.005913326 | -0.077876294 | 0.0183579 | 0.0282 |
| High SDI | Digestive congenital anomalies | 1990 | -0.08082516 | -0.025946414 | -0.135703906 | 0.02799936 | 0.0064 |
| High SDI | Digestive congenital anomalies | 2021 | -0.0328824 | 0.009298584 | -0.075063384 | 0.02152091 | 0.1348 |
| High SDI | Down syndrome | 1990 | 0.01238975 | 0.148904534 | -0.124125034 | 0.0696504 | 0.8598 |
| High SDI | Down syndrome | 2021 | 0.01884902 | 0.083295839 | -0.045597799 | 0.03288103 | 0.5699 |
| High SDI | Neural tube defects | 1990 | -0.15773434 | -0.051801201 | -0.263667479 | 0.05404752 | 0.0059 |
| High SDI | Neural tube defects | 2021 | -0.01226378 | 0.055363217 | -0.079890777 | 0.03450357 | 0.7242 |
| Eastern Sub-Saharan Africa | Congenital heart anomalies | 1990 | -0.08767163 | -0.022805665 | -0.152537595 | 0.03309488 | 0.02 |
| Eastern Sub-Saharan Africa | Congenital heart anomalies | 2021 | -0.08300996 | -0.016638108 | -0.149381812 | 0.03386319 | 0.0291 |
| Eastern Sub-Saharan Africa | Digestive congenital anomalies | 1990 | -0.01943819 | 0.051606263 | -0.090482643 | 0.03624717 | 0.6008 |
| Eastern Sub-Saharan Africa | Digestive congenital anomalies | 2021 | -0.00244346 | 0.072862954 | -0.077749874 | 0.03842164 | 0.9503 |
| Eastern Sub-Saharan Africa | Down syndrome | 1990 | -0.0483203 | 0.03361311 | -0.13025371 | 0.04180276 | 0.2685 |
| Eastern Sub-Saharan Africa | Down syndrome | 2021 | -0.02716119 | 0.051350785 | -0.105673165 | 0.04005713 | 0.5096 |
| Eastern Sub-Saharan Africa | Neural tube defects | 1990 | -0.06009414 | 0.005639595 | -0.125827875 | 0.03353762 | 0.0965 |
| Eastern Sub-Saharan Africa | Neural tube defects | 2021 | -0.05096807 | 0.018570221 | -0.120506361 | 0.03547872 | 0.1745 |
| Western Sub-Saharan Africa | Congenital heart anomalies | 1990 | -0.10317704 | -0.052725327 | -0.153628753 | 0.02574067 | 0.0009 |
| Western Sub-Saharan Africa | Congenital heart anomalies | 2021 | -0.04122734 | 0.007915191 | -0.090369871 | 0.02507272 | 0.1185 |
| Western Sub-Saharan Africa | Digestive congenital anomalies | 1990 | -0.06098571 | -0.011303983 | -0.110667437 | 0.02534782 | 0.0278 |
| Western Sub-Saharan Africa | Digestive congenital anomalies | 2021 | 0.02729475 | 0.080742696 | -0.026153196 | 0.02726936 | 0.3309 |
| Western Sub-Saharan Africa | Down syndrome | 1990 | -0.07166925 | -0.02666322 | -0.11667528 | 0.02296226 | 0.0062 |
| Western Sub-Saharan Africa | Down syndrome | 2021 | 0.00659848 | 0.042399389 | -0.029202429 | 0.01826577 | 0.7224 |
| Western Sub-Saharan Africa | Neural tube defects | 1990 | -0.06175095 | -0.010426253 | -0.113075647 | 0.02618607 | 0.0306 |
| Western Sub-Saharan Africa | Neural tube defects | 2021 | 0.00372689 | 0.057861384 | -0.050407604 | 0.02761964 | 0.8942 |
| Central Sub-Saharan Africa | Congenital heart anomalies | 1990 | -0.05238007 | -0.03168688 | -0.07307326 | 0.01055775 | 0.0077 |
| Central Sub-Saharan Africa | Congenital heart anomalies | 2021 | -0.02422624 | 0.06852733 | -0.11697981 | 0.04732325 | 0.6357 |
| Central Sub-Saharan Africa | Digestive congenital anomalies | 1990 | -0.01858441 | 0.00649769 | -0.04366651 | 0.01279699 | 0.2201 |
| Central Sub-Saharan Africa | Digestive congenital anomalies | 2021 | 0.00860077 | 0.078753678 | -0.061552138 | 0.0357923 | 0.8219 |
| Central Sub-Saharan Africa | Down syndrome | 1990 | -0.03599345 | -0.00915515 | -0.06283175 | 0.01369301 | 0.0583 |
| Central Sub-Saharan Africa | Down syndrome | 2021 | -0.0061228 | 0.052261856 | -0.064507456 | 0.02978809 | 0.8472 |
| Central Sub-Saharan Africa | Neural tube defects | 1990 | -0.03357319 | 0.005747938 | -0.072894318 | 0.0200618 | 0.1695 |
| Central Sub-Saharan Africa | Neural tube defects | 2021 | -0.06115779 | 0.015109612 | -0.137425192 | 0.03891194 | 0.1911 |
| North Africa and Middle East | Congenital heart anomalies | 1990 | -0.0587067 | 0.023612869 | -0.141026269 | 0.04199978 | 0.1783 |
| North Africa and Middle East | Congenital heart anomalies | 2021 | -0.30468087 | -0.168478647 | -0.440883093 | 0.06949093 | 0.0003 |
| North Africa and Middle East | Digestive congenital anomalies | 1990 | 0.04269828 | 0.142541111 | -0.057144551 | 0.05094022 | 0.4123 |
| North Africa and Middle East | Digestive congenital anomalies | 2021 | -0.17137686 | -0.049049262 | -0.293704458 | 0.06241204 | 0.0128 |
| North Africa and Middle East | Down syndrome | 1990 | 0.04088145 | 0.120253924 | -0.038491024 | 0.04049616 | 0.3254 |
| North Africa and Middle East | Down syndrome | 2021 | -0.09721764 | 0.037824282 | -0.232259562 | 0.06889894 | 0.1744 |
| North Africa and Middle East | Neural tube defects | 1990 | -0.13966571 | -0.015504394 | -0.263827026 | 0.06334761 | 0.04 |
| North Africa and Middle East | Neural tube defects | 2021 | -0.33380376 | -0.163585247 | -0.504022273 | 0.08684618 | 0.0011 |
| Oceania | Congenital heart anomalies | 1990 | -0.10419817 | -0.052814653 | -0.155581687 | 0.02621608 | 0.0011 |
| Oceania | Congenital heart anomalies | 2021 | -0.10517708 | -0.087229713 | -0.123124447 | 0.00915682 | 0 |
| Oceania | Digestive congenital anomalies | 1990 | -0.05738903 | -0.015704573 | -0.099073487 | 0.02126758 | 0.0158 |
| Oceania | Digestive congenital anomalies | 2021 | -0.05285097 | -0.018388133 | -0.087313807 | 0.01758308 | 0.0084 |
| Oceania | Down syndrome | 1990 | -0.10693179 | -0.054129135 | -0.159734445 | 0.02694013 | 0.0011 |
| Oceania | Down syndrome | 2021 | -0.09185452 | -0.07269505 | -0.11101399 | 0.00977524 | 0 |
| Oceania | Neural tube defects | 1990 | -0.15873546 | -0.102323896 | -0.215147024 | 0.02878141 | 0 |
| Oceania | Neural tube defects | 2021 | -0.13967151 | -0.132244345 | -0.147098675 | 0.00378937 | 0 |
| South Asia | Congenital heart anomalies | 1990 | -0.03555746 | 0.03106735 | -0.10218227 | 0.03399225 | 0.3724 |
| South Asia | Congenital heart anomalies | 2021 | 0.00409144 | 0.043808782 | -0.035625902 | 0.02026395 | 0.8529 |
| South Asia | Digestive congenital anomalies | 1990 | -0.00805963 | 0.031803732 | -0.047922992 | 0.02033845 | 0.7184 |
| South Asia | Digestive congenital anomalies | 2021 | 0.02935576 | 0.061959713 | -0.003248193 | 0.01663467 | 0.1758 |
| South Asia | Down syndrome | 1990 | -0.00791973 | 0.013047135 | -0.028886595 | 0.01069738 | 0.5128 |
| South Asia | Down syndrome | 2021 | -0.01674047 | 0.041171434 | -0.074652374 | 0.02954689 | 0.6106 |
| South Asia | Neural tube defects | 1990 | -0.03031481 | 0.05826298 | -0.1188926 | 0.04519275 | 0.5504 |
| South Asia | Neural tube defects | 2021 | -0.00813586 | 0.03201668 | -0.0482884 | 0.02048599 | 0.7178 |
| Caribbean | Congenital heart anomalies | 1990 | -0.32041432 | -0.182487238 | -0.458341402 | 0.07037096 | 0.0003 |
| Caribbean | Congenital heart anomalies | 2021 | -0.39430593 | -0.251633022 | -0.536978838 | 0.0727923 | 0 |
| Caribbean | Digestive congenital anomalies | 1990 | -0.17478151 | -0.12094374 | -0.22861928 | 0.02746825 | 0 |
| Caribbean | Digestive congenital anomalies | 2021 | -0.25418059 | -0.189553647 | -0.318807533 | 0.03297293 | 0 |
| Caribbean | Down syndrome | 1990 | -0.33385868 | -0.221765418 | -0.445951942 | 0.05719044 | 0 |
| Caribbean | Down syndrome | 2021 | -0.27538927 | -0.170217787 | -0.380560753 | 0.05365892 | 0.0001 |
| Caribbean | Neural tube defects | 1990 | -0.44865872 | -0.268652242 | -0.628665198 | 0.09184004 | 0.0001 |
| Caribbean | Neural tube defects | 2021 | -0.46426541 | -0.304269395 | -0.624261425 | 0.08163062 | 0 |
| Southeast Asia | Congenital heart anomalies | 1990 | -0.15016877 | 0.01509302 | -0.31543056 | 0.08431724 | 0.1025 |
| Southeast Asia | Congenital heart anomalies | 2021 | -0.17467085 | -0.014758272 | -0.334583428 | 0.08158805 | 0.0555 |
| Southeast Asia | Digestive congenital anomalies | 1990 | -0.11851799 | 0.033350121 | -0.270386101 | 0.07748373 | 0.1544 |
| Southeast Asia | Digestive congenital anomalies | 2021 | -0.1675406 | -0.0177633 | -0.3173179 | 0.07641699 | 0.0508 |
| Southeast Asia | Down syndrome | 1990 | -0.09512541 | 0.094282326 | -0.284533146 | 0.0966366 | 0.3461 |
| Southeast Asia | Down syndrome | 2021 | -0.08986435 | 0.057280768 | -0.237009468 | 0.07507404 | 0.2565 |
| Southeast Asia | Neural tube defects | 1990 | -0.17154807 | -0.024650029 | -0.318446111 | 0.07494798 | 0.0429 |
| Southeast Asia | Neural tube defects | 2021 | -0.14839099 | 0.020190276 | -0.316972256 | 0.08601085 | 0.1124 |
| Southern Sub-Saharan Africa | Congenital heart anomalies | 1990 | 0.03017054 | 0.052745212 | 0.007595868 | 0.01151769 | 0.0588 |
| Southern Sub-Saharan Africa | Congenital heart anomalies | 2021 | -0.04937989 | -0.017753761 | -0.081006019 | 0.01613578 | 0.0376 |
| Southern Sub-Saharan Africa | Digestive congenital anomalies | 1990 | 0.07788121 | 0.098392159 | 0.057370261 | 0.01046477 | 0.0017 |
| Southern Sub-Saharan Africa | Digestive congenital anomalies | 2021 | -0.01162015 | 0.011423315 | -0.034663615 | 0.01175687 | 0.3789 |
| Southern Sub-Saharan Africa | Down syndrome | 1990 | 0.0360185 | 0.058927156 | 0.013109844 | 0.01168809 | 0.0369 |
| Southern Sub-Saharan Africa | Down syndrome | 2021 | -0.03423057 | -0.013745963 | -0.054715177 | 0.01045133 | 0.0306 |
| Southern Sub-Saharan Africa | Neural tube defects | 1990 | 0.07406707 | 0.142947879 | 0.005186261 | 0.03514327 | 0.1028 |
| Southern Sub-Saharan Africa | Neural tube defects | 2021 | -0.02968856 | 0.022441364 | -0.081818484 | 0.0265969 | 0.3268 |
| Central Latin America | Congenital heart anomalies | 1990 | -0.03552229 | 0.068341481 | -0.139386061 | 0.05299172 | 0.5241 |
| Central Latin America | Congenital heart anomalies | 2021 | 0.06326235 | 0.113907006 | 0.012617694 | 0.02583911 | 0.0442 |
| Central Latin America | Digestive congenital anomalies | 1990 | 0.01340556 | 0.111051349 | -0.084240229 | 0.04981928 | 0.7956 |
| Central Latin America | Digestive congenital anomalies | 2021 | -0.00617056 | 0.087988644 | -0.100329764 | 0.04804041 | 0.9014 |
| Central Latin America | Down syndrome | 1990 | 0.07819958 | 0.167876695 | -0.011477535 | 0.04575363 | 0.1312 |
| Central Latin America | Down syndrome | 2021 | 0.0154321 | 0.15294372 | -0.12207952 | 0.07015899 | 0.8322 |
| Central Latin America | Neural tube defects | 1990 | 0.01904597 | 0.175454793 | -0.137362853 | 0.07980042 | 0.8182 |
| Central Latin America | Neural tube defects | 2021 | -0.05004974 | 0.072759685 | -0.172859165 | 0.06265787 | 0.4507 |
| Central Asia | Congenital heart anomalies | 1990 | 0.06496833 | 0.184592128 | -0.054655468 | 0.06103255 | 0.3224 |
| Central Asia | Congenital heart anomalies | 2021 | -0.02122265 | 0.092303686 | -0.134748986 | 0.0579216 | 0.7249 |
| Central Asia | Digestive congenital anomalies | 1990 | 0.04501913 | 0.303011519 | -0.212973259 | 0.13162877 | 0.7424 |
| Central Asia | Digestive congenital anomalies | 2021 | 0.01830909 | 0.202444348 | -0.165826168 | 0.09394656 | 0.851 |
| Central Asia | Down syndrome | 1990 | -0.22021134 | 0.108998806 | -0.549421486 | 0.16796436 | 0.2312 |
| Central Asia | Down syndrome | 2021 | -0.21126265 | 0.137561157 | -0.560086457 | 0.17797133 | 0.2739 |
| Central Asia | Neural tube defects | 1990 | -0.00042428 | 0.123314244 | -0.124162804 | 0.0631319 | 0.9948 |
| Central Asia | Neural tube defects | 2021 | -0.09862295 | 0.089581758 | -0.286827658 | 0.09602281 | 0.3386 |
| East Asia | Congenital heart anomalies | 1990 | -0.01662749 | -0.004704202 | -0.028550778 | 0.00608331 | 0.2233 |
| East Asia | Congenital heart anomalies | 2021 | -0.01151761 | -0.00226545 | -0.02076977 | 0.00472049 | 0.2476 |
| East Asia | Digestive congenital anomalies | 1990 | -0.01415903 | 0.000574153 | -0.028892213 | 0.00751693 | 0.3107 |
| East Asia | Digestive congenital anomalies | 2021 | -0.00959672 | 0.006520399 | -0.025713839 | 0.00822302 | 0.451 |
| East Asia | Down syndrome | 1990 | -0.02531273 | -0.012998109 | -0.037627351 | 0.00628297 | 0.1549 |
| East Asia | Down syndrome | 2021 | -0.00027302 | 0.021359304 | -0.021905344 | 0.0110369 | 0.9843 |
| East Asia | Neural tube defects | 1990 | -0.0241796 | -0.01043706 | -0.03792214 | 0.0070115 | 0.1797 |
| East Asia | Neural tube defects | 2021 | -0.02094702 | -0.007568374 | -0.034325666 | 0.00682584 | 0.2005 |
| Andean Latin America | Congenital heart anomalies | 1990 | -0.20237363 | -0.083343418 | -0.321403842 | 0.0607297 | 0.1856 |
| Andean Latin America | Congenital heart anomalies | 2021 | -0.18416352 | -0.022771808 | -0.345555232 | 0.08234271 | 0.2677 |
| Andean Latin America | Digestive congenital anomalies | 1990 | -0.20422776 | -0.155112826 | -0.253342694 | 0.02505864 | 0.0777 |
| Andean Latin America | Digestive congenital anomalies | 2021 | -0.20350742 | -0.057469819 | -0.349545021 | 0.07450898 | 0.2234 |
| Andean Latin America | Down syndrome | 1990 | -0.12659834 | 0.099439797 | -0.352636477 | 0.11532558 | 0.4704 |
| Andean Latin America | Down syndrome | 2021 | -0.19765161 | 0.019594829 | -0.414898049 | 0.11084002 | 0.3254 |
| Andean Latin America | Neural tube defects | 1990 | -0.23047221 | -0.202757026 | -0.258187394 | 0.0141404 | 0.039 |
| Andean Latin America | Neural tube defects | 2021 | -0.12022595 | 0.207252908 | -0.447704808 | 0.16708105 | 0.6029 |
| Central Europe | Congenital heart anomalies | 1990 | -0.07536886 | 0.002340691 | -0.153078411 | 0.03964773 | 0.0838 |
| Central Europe | Congenital heart anomalies | 2021 | -0.10629024 | -0.017530288 | -0.195050192 | 0.04528569 | 0.0387 |
| Central Europe | Digestive congenital anomalies | 1990 | -0.1078014 | 0.013036246 | -0.228639046 | 0.06165186 | 0.1082 |
| Central Europe | Digestive congenital anomalies | 2021 | -0.15798095 | -0.033112251 | -0.282849649 | 0.06370852 | 0.0306 |
| Central Europe | Down syndrome | 1990 | 0.1565568 | 0.40337811 | -0.09026451 | 0.12592924 | 0.2396 |
| Central Europe | Down syndrome | 2021 | -0.01175087 | 0.177387268 | -0.200889008 | 0.09649905 | 0.9053 |
| Central Europe | Neural tube defects | 1990 | -0.16473817 | -0.08001323 | -0.24946311 | 0.04322701 | 0.0029 |
| Central Europe | Neural tube defects | 2021 | -0.16126065 | -0.037844585 | -0.284676715 | 0.06296738 | 0.0265 |
| Eastern Europe | Congenital heart anomalies | 1990 | -0.06986014 | -0.044943013 | -0.094777267 | 0.01271282 | 0.0027 |
| Eastern Europe | Congenital heart anomalies | 2021 | -0.12260632 | -0.061466648 | -0.183745992 | 0.03119371 | 0.0111 |
| Eastern Europe | Digestive congenital anomalies | 1990 | 0.01084522 | 0.07268957 | -0.05099913 | 0.03155324 | 0.745 |
| Eastern Europe | Digestive congenital anomalies | 2021 | -0.05132614 | 0.036959473 | -0.139611753 | 0.04504368 | 0.3061 |
| Eastern Europe | Down syndrome | 1990 | 0.12752513 | 0.247575444 | 0.007474816 | 0.06125016 | 0.0918 |
| Eastern Europe | Down syndrome | 2021 | -0.0358554 | 0.073661246 | -0.145372046 | 0.05587584 | 0.5493 |
| Eastern Europe | Neural tube defects | 1990 | -0.24249216 | -0.15947144 | -0.32551288 | 0.04235751 | 0.0023 |
| Eastern Europe | Neural tube defects | 2021 | -0.29340152 | -0.165091159 | -0.421711881 | 0.06546447 | 0.0065 |
| Southern Latin America | Congenital heart anomalies | 1990 | 0.05893316 | 0.155312397 | -0.037446077 | 0.04917308 | 0.4427 |
| Southern Latin America | Congenital heart anomalies | 2021 | -0.11324009 | -0.014243116 | -0.212237064 | 0.05050866 | 0.2671 |
| Southern Latin America | Digestive congenital anomalies | 1990 | 0.01156522 | 0.055551697 | -0.032421257 | 0.02244208 | 0.6971 |
| Southern Latin America | Digestive congenital anomalies | 2021 | -0.08425971 | 0.016653614 | -0.185173034 | 0.05148639 | 0.3492 |
| Southern Latin America | Down syndrome | 1990 | -0.09047511 | -0.03392374 | -0.14702648 | 0.02885274 | 0.1965 |
| Southern Latin America | Down syndrome | 2021 | -0.06218914 | -0.022626383 | -0.101751897 | 0.02018508 | 0.1998 |
| Southern Latin America | Neural tube defects | 1990 | 0.0342717 | 0.099533604 | -0.030990204 | 0.03329689 | 0.4908 |
| Southern Latin America | Neural tube defects | 2021 | -0.02530055 | 0.083513378 | -0.134114478 | 0.05551731 | 0.7278 |
| Western Europe | Congenital heart anomalies | 1990 | -0.0437453 | -0.00409989 | -0.08339071 | 0.02022725 | 0.0417 |
| Western Europe | Congenital heart anomalies | 2021 | -0.00187545 | 0.024835763 | -0.028586663 | 0.01362817 | 0.8918 |
| Western Europe | Digestive congenital anomalies | 1990 | -0.04030348 | 0.007599665 | -0.088206625 | 0.02444038 | 0.1133 |
| Western Europe | Digestive congenital anomalies | 2021 | 0.03068701 | 0.065684672 | -0.004310652 | 0.01785595 | 0.0997 |
| Western Europe | Down syndrome | 1990 | -0.04158199 | 0.043602021 | -0.126766001 | 0.04346123 | 0.3491 |
| Western Europe | Down syndrome | 2021 | 0.07230001 | 0.11294896 | 0.03165106 | 0.02073926 | 0.0021 |
| Western Europe | Neural tube defects | 1990 | -0.05840573 | 0.047481506 | -0.164292966 | 0.0540241 | 0.2914 |
| Western Europe | Neural tube defects | 2021 | 0.11968829 | 0.181759608 | 0.057616972 | 0.03166904 | 0.001 |
| High-income North America | Congenital heart anomalies | 1990 | 0.00418774 | 0.009192855 | -0.000817375 | 0.00255363 | 0.3486 |
| High-income North America | Congenital heart anomalies | 2021 | -0.00804034 | -0.007186682 | -0.008893998 | 0.00043554 | 0.0345 |
| High-income North America | Digestive congenital anomalies | 1990 | 0.00738481 | 0.021032564 | -0.006262944 | 0.00696314 | 0.4813 |
| High-income North America | Digestive congenital anomalies | 2021 | -0.01363698 | -0.011546444 | -0.015727516 | 0.0010666 | 0.0497 |
| High-income North America | Down syndrome | 1990 | 0.05785845 | 0.067575758 | 0.048141142 | 0.00495781 | 0.0544 |
| High-income North America | Down syndrome | 2021 | 0.04349663 | 0.046697428 | 0.040295832 | 0.00163306 | 0.0239 |
| High-income North America | Neural tube defects | 1990 | 0.00952608 | 0.022943044 | -0.003890884 | 0.00684539 | 0.3967 |
| High-income North America | Neural tube defects | 2021 | -0.0285977 | -0.028131592 | -0.029063808 | 0.00023781 | 0.0053 |
| High-income Asia Pacific | Congenital heart anomalies | 1990 | -0.07295259 | -0.065287285 | -0.080617895 | 0.00391087 | 0.0029 |
| High-income Asia Pacific | Congenital heart anomalies | 2021 | -0.02028016 | 0.045035194 | -0.085595514 | 0.03332416 | 0.6047 |
| High-income Asia Pacific | Digestive congenital anomalies | 1990 | -0.01809477 | -0.002670668 | -0.033518872 | 0.00786944 | 0.1482 |
| High-income Asia Pacific | Digestive congenital anomalies | 2021 | -0.02995454 | 0.031896416 | -0.091805496 | 0.03155661 | 0.4427 |
| High-income Asia Pacific | Down syndrome | 1990 | 0.01967052 | 0.056813108 | -0.017472068 | 0.0189503 | 0.4083 |
| High-income Asia Pacific | Down syndrome | 2021 | 0.00916488 | 0.157484038 | -0.139154278 | 0.07567304 | 0.9147 |
| High-income Asia Pacific | Neural tube defects | 1990 | -0.10969053 | 0.153674709 | -0.373055769 | 0.13437002 | 0.5001 |
| High-income Asia Pacific | Neural tube defects | 2021 | -0.0638482 | 0.125060422 | -0.252756822 | 0.09638195 | 0.5758 |
| Sub-Saharan Africa | Congenital heart anomalies | 1990 | -0.14266312 | -0.099302765 | -0.186023475 | 0.02212263 | 0 |
| Sub-Saharan Africa | Congenital heart anomalies | 2021 | -0.05945812 | -0.000999101 | -0.117917139 | 0.02982603 | 0.0524 |
| Sub-Saharan Africa | Digestive congenital anomalies | 1990 | -0.09871923 | -0.05391998 | -0.14351848 | 0.02285676 | 0.0001 |
| Sub-Saharan Africa | Digestive congenital anomalies | 2021 | 0.00630216 | 0.069079137 | -0.056474817 | 0.03202907 | 0.8449 |
| Sub-Saharan Africa | Down syndrome | 1990 | -0.11079023 | -0.075693314 | -0.145887146 | 0.01790659 | 0 |
| Sub-Saharan Africa | Down syndrome | 2021 | -0.0060331 | 0.03492598 | -0.04699218 | 0.02089749 | 0.7742 |
| Sub-Saharan Africa | Neural tube defects | 1990 | -0.15354111 | -0.109453968 | -0.197628252 | 0.02249344 | 0 |
| Sub-Saharan Africa | Neural tube defects | 2021 | -0.0729795 | -0.024935039 | -0.121023961 | 0.02451248 | 0.0047 |
| North Africa and Middle East | Congenital heart anomalies | 1990 | -0.0587067 | 0.023612869 | -0.141026269 | 0.04199978 | 0.1783 |
| North Africa and Middle East | Congenital heart anomalies | 2021 | -0.30468087 | -0.168478647 | -0.440883093 | 0.06949093 | 0.0003 |
| North Africa and Middle East | Digestive congenital anomalies | 1990 | 0.04269828 | 0.142541111 | -0.057144551 | 0.05094022 | 0.4123 |
| North Africa and Middle East | Digestive congenital anomalies | 2021 | -0.17137686 | -0.049049262 | -0.293704458 | 0.06241204 | 0.0128 |
| North Africa and Middle East | Down syndrome | 1990 | 0.04088145 | 0.120253924 | -0.038491024 | 0.04049616 | 0.3254 |
| North Africa and Middle East | Down syndrome | 2021 | -0.09721764 | 0.037824282 | -0.232259562 | 0.06889894 | 0.1744 |
| North Africa and Middle East | Neural tube defects | 1990 | -0.13966571 | -0.015504394 | -0.263827026 | 0.06334761 | 0.04 |
| North Africa and Middle East | Neural tube defects | 2021 | -0.33380376 | -0.163585247 | -0.504022273 | 0.08684618 | 0.0011 |
| Southeast Asia, east Asia, and Oceania | Congenital heart anomalies | 1990 | -0.02074919 | 0.025093211 | -0.066591591 | 0.02338898 | 0.3816 |
| Southeast Asia, east Asia, and Oceania | Congenital heart anomalies | 2021 | -0.12321358 | -0.056172015 | -0.190255145 | 0.03420488 | 0.0011 |
| Southeast Asia, east Asia, and Oceania | Digestive congenital anomalies | 1990 | -0.01226273 | 0.031257796 | -0.055783256 | 0.02220435 | 0.5846 |
| Southeast Asia, east Asia, and Oceania | Digestive congenital anomalies | 2021 | -0.09511661 | -0.035887468 | -0.154345752 | 0.03021895 | 0.0036 |
| Southeast Asia, east Asia, and Oceania | Down syndrome | 1990 | 0.02519905 | 0.092864777 | -0.042466677 | 0.03452333 | 0.4708 |
| Southeast Asia, east Asia, and Oceania | Down syndrome | 2021 | 0.00315009 | 0.077718878 | -0.071418698 | 0.0380453 | 0.9345 |
| Southeast Asia, east Asia, and Oceania | Neural tube defects | 1990 | -0.05428196 | -0.011216017 | -0.097347903 | 0.02197242 | 0.019 |
| Southeast Asia, east Asia, and Oceania | Neural tube defects | 2021 | -0.3215618 | -0.214786586 | -0.428337014 | 0.05447715 | 0 |
| South Asia | Congenital heart anomalies | 1990 | -0.03555746 | 0.03106735 | -0.10218227 | 0.03399225 | 0.3724 |
| South Asia | Congenital heart anomalies | 2021 | 0.00409144 | 0.043808782 | -0.035625902 | 0.02026395 | 0.8529 |
| South Asia | Digestive congenital anomalies | 1990 | -0.00805963 | 0.031803732 | -0.047922992 | 0.02033845 | 0.7184 |
| South Asia | Digestive congenital anomalies | 2021 | 0.02935576 | 0.061959713 | -0.003248193 | 0.01663467 | 0.1758 |
| South Asia | Down syndrome | 1990 | -0.00791973 | 0.013047135 | -0.028886595 | 0.01069738 | 0.5128 |
| South Asia | Down syndrome | 2021 | -0.01674047 | 0.041171434 | -0.074652374 | 0.02954689 | 0.6106 |
| South Asia | Neural tube defects | 1990 | -0.03031481 | 0.05826298 | -0.1188926 | 0.04519275 | 0.5504 |
| South Asia | Neural tube defects | 2021 | -0.00813586 | 0.03201668 | -0.0482884 | 0.02048599 | 0.7178 |
| Latin America and Caribbean | Congenital heart anomalies | 1990 | -0.08041226 | 0.003851648 | -0.164676168 | 0.04299179 | 0.0701 |
| Latin America and Caribbean | Congenital heart anomalies | 2021 | -0.04463979 | 0.03418163 | -0.12346121 | 0.04021501 | 0.2748 |
| Latin America and Caribbean | Digestive congenital anomalies | 1990 | -0.03010247 | 0.038405939 | -0.098610879 | 0.03495327 | 0.3951 |
| Latin America and Caribbean | Digestive congenital anomalies | 2021 | -0.06758492 | -0.013494565 | -0.121675275 | 0.02759712 | 0.0196 |
| Latin America and Caribbean | Down syndrome | 1990 | 0.00516445 | 0.091146436 | -0.080817536 | 0.04386836 | 0.907 |
| Latin America and Caribbean | Down syndrome | 2021 | -0.03533612 | 0.03419196 | -0.1048642 | 0.03547351 | 0.3262 |
| Latin America and Caribbean | Neural tube defects | 1990 | 0.00010402 | 0.090897786 | -0.090689746 | 0.04632335 | 0.9982 |
| Latin America and Caribbean | Neural tube defects | 2021 | -0.11640721 | -0.041530878 | -0.191283542 | 0.03820221 | 0.0044 |
| Central Europe, eastern Europe, and central Asia | Congenital heart anomalies | 1990 | -0.05353988 | -0.006676143 | -0.100403617 | 0.02391007 | 0.0336 |
| Central Europe, eastern Europe, and central Asia | Congenital heart anomalies | 2021 | -0.27154564 | -0.187507328 | -0.355583952 | 0.04287669 | 0 |
| Central Europe, eastern Europe, and central Asia | Digestive congenital anomalies | 1990 | 0.02773541 | 0.098001547 | -0.042530727 | 0.03585007 | 0.4459 |
| Central Europe, eastern Europe, and central Asia | Digestive congenital anomalies | 2021 | -0.17935094 | -0.089291645 | -0.269410235 | 0.04594862 | 0.0006 |
| Central Europe, eastern Europe, and central Asia | Down syndrome | 1990 | 0.15948734 | 0.273822882 | 0.045151798 | 0.05833446 | 0.0109 |
| Central Europe, eastern Europe, and central Asia | Down syndrome | 2021 | -0.05380155 | 0.069844129 | -0.177447229 | 0.06308453 | 0.4012 |
| Central Europe, eastern Europe, and central Asia | Neural tube defects | 1990 | -0.16636455 | -0.091453193 | -0.241275907 | 0.03822008 | 0.0002 |
| Central Europe, eastern Europe, and central Asia | Neural tube defects | 2021 | -0.27495402 | -0.168288566 | -0.381619474 | 0.05442115 | 0 |
| High income | Congenital heart anomalies | 1990 | -0.03407198 | 0.004051725 | -0.072195685 | 0.01945087 | 0.0897 |
| High income | Congenital heart anomalies | 2021 | -0.00917936 | 0.013425575 | -0.031784295 | 0.01153313 | 0.4321 |
| High income | Digestive congenital anomalies | 1990 | -0.03796688 | 0.006561654 | -0.082495414 | 0.02271864 | 0.1048 |
| High income | Digestive congenital anomalies | 2021 | -0.0098415 | 0.025624818 | -0.045307818 | 0.01809506 | 0.5904 |
| High income | Down syndrome | 1990 | -0.00597754 | 0.067247766 | -0.079202846 | 0.03735985 | 0.8739 |
| High income | Down syndrome | 2021 | 0.00380158 | 0.066313781 | -0.058710621 | 0.03189398 | 0.9059 |
| High income | Neural tube defects | 1990 | -0.06446542 | 0.025256422 | -0.154187262 | 0.04577645 | 0.169 |
| High income | Neural tube defects | 2021 | 0.02192221 | 0.096472045 | -0.052627625 | 0.03803563 | 0.5685 |

## Table S6 Age-standardized DALYs rates of CHD, NTDs, DCAs, and DS with frontier analysis across all countries and territories

| **Location** | **SDI** | **Congenital heart anomalies** | | | | **Neural tube defects** | | | | **Digestive congenital anomalies** | | | | **Down syndrome** | | | |
| --- | --- | --- | --- | --- | --- | --- | --- | --- | --- | --- | --- | --- | --- | --- | --- | --- | --- |
|  |  | **Rate of DALYs (95% UI)** | **Frontier DALYs** | **Effective difference** | **Effective difference rank (Age-standardized DALYs rank)** | **Rate of DALYs (95% UI)** | **Frontier DALYs** | **Effective difference** | **Effective difference rank (Age-standardized DALYs rank)** | **Rate of DALYs (95% UI)** | **Frontier DALYs** | **Effective difference** | **Effective difference rank (Age-standardized DALYs rank)** | **Rate of DALYs (95% UI)** | **Frontier DALYs** | **Effective difference** | **Effective difference rank (Age-standardized DALYs rank)** |
| Afghanistan | 0.337199998 | 1395.89 (691.74 to 1982.48) | 170.87 | 1225.01 | 204 (204) | 185.88 (104.29 to 285.14) | 76.48 | 109.39 | 176 (186) | 128.82 (62.73 to 227.94) | 20.62 | 108.2 | 189 (191) | 76.58 (34.63 to 167.55) | 10.89 | 65.69 | 184 (185) |
| Albania | 0.706849791 | 268.21 (169.58 to 393.24) | 112.24 | 155.98 | 118 (109) | 42.13 (27.33 to 61.39) | 7.85 | 34.28 | 128 (111) | 25.52 (13.5 to 47.04) | 8.92 | 16.6 | 63 (68) | 10.03 (6.79 to 15.98) | 2.75 | 7.28 | 15 (13) |
| Algeria | 0.659500924 | 461.71 (348.67 to 590.34) | 142.58 | 319.13 | 179 (176) | 57.26 (38.89 to 81.7) | 9.24 | 48.02 | 147 (142) | 70.28 (45.01 to 100.13) | 9.65 | 60.63 | 146 (138) | 95.11 (45.42 to 180.49) | 4.09 | 91.02 | 198 (197) |
| American Samoa | 0.723727533 | 192.43 (134.39 to 267.45) | 98.73 | 93.7 | 85 (75) | 18.31 (10.87 to 28.4) | 6.24 | 12.07 | 57 (63) | 9.05 (4.29 to 35.52) | 7.95 | 1.1 | 6 (6) | 55.85 (25.15 to 92.57) | 2.74 | 53.11 | 163 (156) |
| Andorra | 0.869444113 | 47.32 (33.64 to 62.27) | 47.32 | 0 | 2.5 (2) | 5.32 (3.86 to 7.09) | 2.76 | 2.56 | 8 (3) | 7.12 (4.65 to 10.24) | 3.43 | 3.69 | 12 (3) | 16.38 (11.11 to 24.2) | 2.75 | 13.62 | 40 (33) |
| Angola | 0.453721949 | 313.37 (205.06 to 505.06) | 148.35 | 165.01 | 129 (133) | 165.27 (106.79 to 237.35) | 29.61 | 135.66 | 186 (179) | 79.77 (41.39 to 124) | 16.11 | 63.67 | 150 (154) | 57.72 (27.87 to 133.63) | 6.55 | 51.16 | 161 (161) |
| Antigua and Barbuda | 0.749886887 | 222.28 (186.1 to 257.75) | 66.28 | 156.01 | 119 (85) | 16.98 (13.68 to 20.28) | 5.32 | 11.66 | 56 (58) | 26.16 (21.29 to 39.63) | 3.6 | 22.56 | 77 (71) | 23.71 (17.86 to 30.62) | 2.76 | 20.95 | 72 (67) |
| Argentina | 0.723122973 | 259.66 (210.94 to 319.85) | 96.37 | 163.28 | 126 (104) | 48.24 (37.89 to 60.63) | 5.45 | 42.79 | 139 (123) | 67.17 (49.59 to 86.29) | 8.49 | 58.68 | 143 (134) | 42.91 (35.28 to 53.92) | 2.72 | 40.18 | 134 (127) |
| Armenia | 0.701833194 | 311.25 (243.22 to 398.16) | 110.17 | 201.07 | 148 (130) | 27.24 (20.91 to 36.13) | 7.9 | 19.33 | 88 (83) | 104.73 (73.54 to 137.45) | 9.11 | 95.61 | 183 (178) | 18.04 (12.71 to 25.54) | 2.74 | 15.3 | 46 (41) |
| Australia | 0.844252814 | 74.29 (59.53 to 89.22) | 54.34 | 19.95 | 22 (12) | 15.95 (12.08 to 20.41) | 3.28 | 12.67 | 60 (47) | 13.61 (10.33 to 21.33) | 3.23 | 10.37 | 32 (22) | 25.84 (21.68 to 29.96) | 2.76 | 23.08 | 79 (75) |
| Austria | 0.853837004 | 88.63 (72.06 to 107.87) | 54.91 | 33.72 | 35 (23) | 10.32 (8.46 to 12.57) | 2.8 | 7.53 | 36 (27) | 19.16 (15.21 to 25.09) | 3.31 | 15.85 | 60 (49) | 36.2 (31.2 to 41.13) | 2.74 | 33.46 | 119 (116) |
| Azerbaijan | 0.694851274 | 405.83 (259.87 to 635.46) | 116.91 | 288.92 | 174 (163) | 51.48 (35.44 to 77.59) | 7.8 | 43.68 | 142 (130) | 48.68 (27.84 to 79.96) | 9.01 | 39.67 | 110 (104) | 9.23 (6.02 to 14.78) | 2.74 | 6.49 | 12 (9) |
| Bahamas | 0.805020668 | 213.9 (159.24 to 281.02) | 54.83 | 159.07 | 121 (82) | 17.65 (13.21 to 23.59) | 3.06 | 14.59 | 72 (60) | 29.41 (20.65 to 41.45) | 3.3 | 26.12 | 83 (76) | 28.18 (22.12 to 35.89) | 2.72 | 25.46 | 90 (84) |
| Bahrain | 0.753043204 | 148.94 (116.92 to 186.71) | 66.09 | 82.86 | 78 (56) | 23.43 (18.47 to 29.33) | 4.96 | 18.47 | 84 (75) | 18.72 (11.48 to 36.05) | 4.62 | 14.1 | 51 (45) | 22.28 (16.87 to 29.51) | 2.73 | 19.55 | 61 (56) |
| Bangladesh | 0.492420885 | 291.23 (142.49 to 516.44) | 152.52 | 138.7 | 106 (118) | 64.69 (29.61 to 112.88) | 28.96 | 35.72 | 130 (148) | 46.65 (19.85 to 94.57) | 14.49 | 32.16 | 94 (100) | 17.84 (9.81 to 33.26) | 6.51 | 11.33 | 30 (40) |
| Barbados | 0.746748764 | 297.94 (218.63 to 404.77) | 62.33 | 235.61 | 161 (123) | 15.36 (11.35 to 20.55) | 5.43 | 9.93 | 50 (42) | 65.58 (45.89 to 91.36) | 4.15 | 61.43 | 147 (133) | 29.57 (22.03 to 39.66) | 2.74 | 26.84 | 97 (92) |
| Belarus | 0.784484711 | 100.89 (76.55 to 159.17) | 57 | 43.89 | 46 (35) | 12.26 (9.24 to 15.95) | 3.41 | 8.86 | 43 (33) | 27.37 (17.19 to 40.71) | 3.17 | 24.19 | 80 (74) | 29.54 (21.85 to 39.97) | 2.74 | 26.8 | 96 (90) |
| Belgium | 0.853654016 | 91.52 (72.55 to 109.41) | 54.44 | 37.08 | 38 (27) | 12.15 (9.46 to 15.15) | 2.79 | 9.37 | 45 (32) | 16.92 (13.15 to 22.48) | 3.16 | 13.75 | 48 (35) | 31.23 (26.36 to 36.52) | 2.76 | 28.47 | 102 (96) |
| Belize | 0.610229002 | 210.31 (172.14 to 259.47) | 151.87 | 58.43 | 58 (80) | 27.76 (21.38 to 35.88) | 10.08 | 17.68 | 80 (84) | 71.21 (55.69 to 91.35) | 10.65 | 60.56 | 145 (139) | 31.91 (26.49 to 38.86) | 5.97 | 25.94 | 93 (99) |
| Benin | 0.373486574 | 518.17 (329.05 to 756.02) | 146.81 | 371.36 | 184 (179) | 192.94 (132.53 to 266.11) | 41.93 | 151 | 192 (189) | 133.83 (85.52 to 194.55) | 17.54 | 116.29 | 194 (192) | 64.34 (36.6 to 124.42) | 9.15 | 55.19 | 169 (174) |
| Bermuda | 0.821365422 | 110.94 (63.71 to 171.6) | 55.3 | 55.64 | 56 (40) | 7.48 (4.43 to 12.82) | 3.11 | 4.37 | 12 (7) | 24.52 (15.62 to 38.35) | 3.2 | 21.33 | 74 (67) | 35.56 (20.03 to 61.33) | 2.73 | 32.83 | 118 (112) |
| Bhutan | 0.473062378 | 316.49 (159.03 to 523.05) | 154.66 | 161.83 | 125 (137) | 49.06 (24.84 to 89.4) | 28.97 | 20.1 | 90 (126) | 53.01 (22.33 to 106.13) | 14.92 | 38.09 | 107 (111) | 19.98 (10.79 to 36.48) | 6.55 | 13.43 | 39 (47) |
| Bolivia (Plurinational State of) | 0.599010799 | 636.42 (475.22 to 832.3) | 151.18 | 485.25 | 192 (190) | 78.52 (54.15 to 112.39) | 16.16 | 62.36 | 157 (151) | 166.26 (107.71 to 231.49) | 10.88 | 155.38 | 204 (202) | 72.03 (44.67 to 105.9) | 5.96 | 66.06 | 186 (183) |
| Bosnia and Herzegovina | 0.723077893 | 115.73 (88.91 to 153.18) | 97.37 | 18.36 | 20 (42) | 15.93 (10.46 to 22.45) | 5.59 | 10.34 | 51 (46) | 17.15 (10.33 to 34.34) | 7.93 | 9.22 | 29 (37) | 17.67 (12.67 to 24.55) | 2.74 | 14.92 | 44 (38) |
| Botswana | 0.642721629 | 172.75 (116.52 to 245.5) | 150.17 | 22.58 | 23 (67) | 47.38 (29.73 to 70.02) | 9.24 | 38.13 | 136 (122) | 43.15 (21.87 to 72.61) | 10.27 | 32.87 | 96 (94) | 45.67 (28.35 to 69.92) | 4.08 | 41.6 | 139 (134) |
| Brazil | 0.653043887 | 274.13 (223.03 to 330.25) | 140.49 | 133.64 | 101 (112) | 46 (35.96 to 59.94) | 9.24 | 36.77 | 133 (119) | 75.59 (60.63 to 94.15) | 9.92 | 65.67 | 153 (145) | 31.23 (26.05 to 38.82) | 4.08 | 27.14 | 98 (95) |
| Brunei Darussalam | 0.810234367 | 230.04 (177.13 to 287.47) | 54.39 | 175.65 | 138 (88) | 56.5 (40.36 to 75.84) | 3.14 | 53.36 | 152 (140) | 35.03 (22.81 to 49.14) | 3.28 | 31.75 | 93 (86) | 79.26 (60.07 to 102.62) | 2.73 | 76.53 | 193 (190) |
| Bulgaria | 0.768150939 | 218.72 (180.14 to 262.84) | 63.63 | 155.09 | 117 (84) | 15.99 (11.92 to 20.95) | 4.34 | 11.65 | 55 (48) | 32.08 (24.33 to 41.25) | 3.44 | 28.64 | 87 (81) | 9.81 (7.53 to 12.86) | 2.74 | 7.07 | 14 (12) |
| Burkina Faso | 0.285118402 | 710.46 (314.35 to 1080.86) | 378.59 | 331.87 | 180 (194) | 222.06 (131.78 to 322.55) | 84.71 | 137.35 | 189 (196) | 174.49 (94.51 to 265.06) | 49.29 | 125.21 | 197 (204) | 88.03 (39.16 to 221.24) | 14.96 | 73.07 | 191 (194) |
| Burundi | 0.289374365 | 326.75 (197.69 to 523.41) | 326.75 | 0 | 2.5 (142) | 208.9 (117.18 to 327.64) | 83.1 | 125.8 | 183 (193) | 78.58 (39.37 to 129.87) | 48.19 | 30.38 | 91 (151) | 54.91 (28.74 to 115.86) | 16.65 | 38.26 | 129 (153) |
| Cabo Verde | 0.533534539 | 172.97 (107.95 to 257.82) | 146.64 | 26.33 | 27 (68) | 46.46 (28.38 to 76.76) | 29.06 | 17.4 | 79 (120) | 44.15 (15.86 to 88.24) | 13.65 | 30.5 | 92 (97) | 27.05 (13.47 to 43.17) | 6.45 | 20.6 | 69 (79) |
| Cambodia | 0.473621491 | 713.9 (499.85 to 963.1) | 153.49 | 560.41 | 195 (195) | 106.89 (63.87 to 183.63) | 28.97 | 77.92 | 159 (160) | 99.06 (49.07 to 164.96) | 14.18 | 84.88 | 174 (175) | 18.05 (10.69 to 28.22) | 6.55 | 11.5 | 32 (42) |
| Cameroon | 0.479691223 | 436.15 (263.64 to 597.75) | 153.55 | 282.6 | 171 (171) | 160.42 (100 to 220.12) | 29.57 | 130.84 | 184 (178) | 137.64 (83.33 to 203.18) | 14.38 | 123.26 | 196 (193) | 61.18 (38.35 to 111.47) | 6.55 | 54.63 | 167 (167) |
| Canada | 0.87317068 | 92.67 (78.73 to 109.51) | 47.71 | 44.96 | 47 (28) | 14.96 (11.76 to 18.21) | 2.71 | 12.25 | 58 (41) | 18.75 (14.27 to 23.52) | 3.3 | 15.45 | 58 (46) | 31.74 (27.76 to 36.11) | 2.73 | 29.01 | 104 (98) |
| Central African Republic | 0.30916769 | 537.55 (248.53 to 918.28) | 323.91 | 213.64 | 153 (181) | 327.55 (171.98 to 482.71) | 60.44 | 267.11 | 204 (203) | 112.37 (41.85 to 195.37) | 26.74 | 85.63 | 175 (183) | 76.81 (28.5 to 214.5) | 10.94 | 65.86 | 185 (186) |
| Chad | 0.240436019 | 601 (229.56 to 908.37) | 414.54 | 186.46 | 143 (187) | 200.27 (115.69 to 300.18) | 99.19 | 101.08 | 170 (190) | 114.76 (56.05 to 178.69) | 58.3 | 56.46 | 139 (184) | 60.05 (24.73 to 151.01) | 18.18 | 41.87 | 140 (165) |
| Chile | 0.771514716 | 128.86 (109.65 to 150.12) | 68.42 | 60.44 | 62 (50) | 38.08 (29.38 to 47.58) | 4.03 | 34.05 | 126 (106) | 39.57 (32.05 to 48.35) | 3.37 | 36.21 | 101 (91) | 33.91 (27.95 to 40.35) | 2.76 | 31.15 | 111 (106) |
| China | 0.72162976 | 241.68 (191.38 to 304.84) | 106.12 | 135.56 | 103 (100) | 16.83 (11.66 to 22.32) | 7.57 | 9.25 | 44 (57) | 40.4 (25.76 to 59.4) | 6.85 | 33.55 | 97 (92) | 23.3 (13.27 to 32.03) | 2.73 | 20.57 | 68 (62) |
| Colombia | 0.655442913 | 305.52 (223.84 to 419.42) | 139.72 | 165.79 | 130 (125) | 18 (13.02 to 24.94) | 9.24 | 8.77 | 42 (62) | 77 (53.1 to 109.86) | 9.7 | 67.3 | 155 (147) | 19.47 (14.39 to 25.7) | 4.07 | 15.4 | 48 (44) |
| Comoros | 0.475978688 | 307.9 (185.08 to 552.85) | 152.93 | 154.97 | 116 (128) | 204.06 (120.58 to 305.36) | 29.35 | 174.71 | 200 (191) | 96.64 (49.55 to 174.24) | 14.47 | 82.17 | 172 (172) | 69.36 (33.42 to 157.05) | 6.54 | 62.82 | 180 (178) |
| Congo | 0.583075236 | 215.16 (144.43 to 327.74) | 151.37 | 63.79 | 65 (83) | 114.02 (67.83 to 163.95) | 16.85 | 97.17 | 165 (165) | 67.68 (41.71 to 116.38) | 12.67 | 55.01 | 136 (136) | 46.94 (27.31 to 83.59) | 6.44 | 40.5 | 135 (137) |
| Cook Islands | 0.779109955 | 84.15 (46.65 to 171.4) | 67.14 | 17.01 | 18 (19) | 6.76 (2.79 to 13.3) | 4.21 | 2.55 | 7 (5) | 7.28 (2.95 to 23.73) | 3.29 | 4 | 15 (5) | 19.74 (3.65 to 43.81) | 2.72 | 17.02 | 53 (45) |
| Costa Rica | 0.700340477 | 259.58 (212.35 to 310.45) | 108.54 | 151.04 | 115 (103) | 43.82 (35.84 to 54.15) | 7.81 | 36.01 | 131 (114) | 73.4 (58.3 to 91.93) | 9.29 | 64.12 | 151 (143) | 37.87 (31.69 to 45.47) | 3.23 | 34.64 | 121 (118) |
| Coted'Ivoire | 0.425941883 | 447.23 (294.71 to 626.29) | 152.47 | 294.76 | 175 (174) | 184.23 (131.79 to 254.15) | 31.16 | 153.07 | 193 (184) | 122.72 (80.61 to 177.66) | 16.54 | 106.18 | 188 (188) | 71.11 (41.81 to 132.59) | 6.56 | 64.56 | 183 (182) |
| Croatia | 0.798341027 | 104.37 (83.05 to 131.17) | 54.96 | 49.41 | 51 (37) | 7.6 (5.94 to 9.78) | 3.06 | 4.53 | 14 (9) | 26.02 (19.41 to 33.71) | 3.35 | 22.66 | 78 (69) | 28.06 (22.28 to 34.9) | 2.72 | 25.34 | 89 (83) |
| Cuba | 0.668729864 | 148.2 (121.94 to 192.23) | 141.87 | 6.33 | 10 (55) | 8.92 (7.52 to 10.74) | 8.37 | 0.54 | 6 (16) | 50.02 (41.02 to 62.93) | 9.59 | 40.44 | 111 (106) | 27.96 (23.14 to 34.23) | 4.05 | 23.91 | 84 (81) |
| Cyprus | 0.835630545 | 60.57 (46.16 to 77.77) | 56.37 | 4.19 | 8 (6) | 8.84 (6.63 to 11.62) | 3.14 | 5.7 | 21 (15) | 11.74 (7.37 to 20.02) | 3.55 | 8.2 | 25 (16) | 26.66 (20.78 to 34.12) | 2.76 | 23.9 | 83 (78) |
| Czechia | 0.828450433 | 55.24 (42.36 to 68.52) | 54.46 | 0.78 | 5 (4) | 7.21 (5.19 to 9.42) | 3.15 | 4.06 | 11 (6) | 10.19 (6.92 to 14.22) | 3.67 | 6.53 | 19 (12) | 15.49 (11.58 to 19.59) | 2.74 | 12.75 | 38 (31) |
| Democratic People's Republic of Korea | 0.569854634 | 291.98 (195.53 to 438.23) | 154.28 | 137.7 | 105 (120) | 24.03 (12.03 to 44.75) | 15.64 | 8.39 | 40 (76) | 42.42 (23.42 to 69.4) | 13.49 | 28.92 | 88 (93) | 16.15 (7.57 to 26.58) | 6.45 | 9.7 | 26 (32) |
| Democratic Republic of the Congo | 0.383179849 | 267.42 (162.87 to 439.7) | 157.67 | 109.75 | 91 (108) | 170.62 (98.25 to 256.57) | 45.06 | 125.57 | 182 (182) | 65.22 (36.51 to 117.4) | 17.55 | 47.67 | 125 (131) | 50.05 (24.59 to 107.46) | 9.09 | 40.96 | 137 (143) |
| Denmark | 0.896424204 | 95.06 (74.11 to 114.63) | 35.78 | 59.28 | 61 (30) | 16.51 (13.44 to 19.81) | 2.66 | 13.85 | 69 (53) | 23.47 (18.26 to 29.24) | 3.24 | 20.23 | 71 (64) | 32.82 (26.86 to 39.46) | 2.74 | 30.08 | 107 (102) |
| Djibouti | 0.487958371 | 241.18 (140.71 to 435.61) | 152.52 | 88.66 | 84 (99) | 109.73 (62.12 to 175.7) | 29.64 | 80.09 | 160 (161) | 67.64 (34.57 to 132.31) | 14.32 | 53.32 | 132 (135) | 58.29 (26 to 128.81) | 6.53 | 51.76 | 162 (162) |
| Dominica | 0.746967185 | 436.03 (288.9 to 615.06) | 79.07 | 356.95 | 182 (170) | 53.53 (35.41 to 79.62) | 5.16 | 48.37 | 148 (136) | 96.56 (59.11 to 143.13) | 5.23 | 91.33 | 179 (171) | 47.69 (33.64 to 64.99) | 2.76 | 44.93 | 147 (139) |
| Dominican Republic | 0.619388201 | 224.69 (132.1 to 414.48) | 151.83 | 72.86 | 70 (86) | 28.01 (17.22 to 49.7) | 9.31 | 18.7 | 85 (85) | 87.22 (47.33 to 166.17) | 10.52 | 76.7 | 167 (162) | 32.78 (21.62 to 54.62) | 6.01 | 26.78 | 95 (101) |
| Ecuador | 0.661017053 | 362.47 (282.28 to 462.07) | 140.53 | 221.93 | 155 (157) | 29.68 (22.75 to 37.9) | 9.24 | 20.44 | 91 (89) | 97.2 (70.77 to 129.8) | 9.75 | 87.45 | 176 (173) | 36.1 (26.78 to 50.63) | 4.12 | 31.98 | 117 (115) |
| Egypt | 0.606787094 | 394.34 (296.59 to 520.64) | 152.37 | 241.97 | 165 (162) | 45.25 (31.08 to 63.57) | 9.64 | 35.62 | 129 (117) | 54.01 (37.3 to 77.87) | 10.85 | 43.15 | 116 (116) | 29.54 (20.45 to 39.88) | 6.01 | 23.53 | 81 (91) |
| El Salvador | 0.563775188 | 301.36 (202.6 to 433.25) | 151.57 | 149.79 | 114 (124) | 58.4 (35.23 to 88.2) | 15.37 | 43.03 | 141 (144) | 27.27 (12.61 to 58.06) | 13.67 | 13.6 | 47 (73) | 14.43 (9.45 to 19.46) | 6.54 | 7.89 | 17 (25) |
| Equatorial Guinea | 0.657857456 | 213.52 (128 to 337.84) | 144.22 | 69.3 | 68 (81) | 111.04 (58.94 to 186.62) | 9.26 | 101.78 | 172 (162) | 80.83 (43.98 to 131.11) | 10.11 | 70.73 | 162 (155) | 35.43 (18.64 to 64.53) | 4.1 | 31.32 | 112 (111) |
| Eritrea | 0.403863943 | 345.11 (197.52 to 662.35) | 154.41 | 190.7 | 144 (151) | 181.3 (105.51 to 281.42) | 39.12 | 142.18 | 190 (183) | 79.49 (41.99 to 157.24) | 17.54 | 61.95 | 148 (152) | 63.94 (29.84 to 144.19) | 9.01 | 54.93 | 168 (172) |
| Estonia | 0.844917787 | 63.48 (46.75 to 82.69) | 55.14 | 8.34 | 12 (9) | 7.69 (5.46 to 10.08) | 3.15 | 4.54 | 15 (11) | 6.91 (4.51 to 12.82) | 3.25 | 3.66 | 11 (2) | 12.24 (9.19 to 15.36) | 2.73 | 9.52 | 25 (22) |
| Eswatini | 0.585459713 | 206.63 (144.98 to 292.81) | 151.18 | 55.45 | 55 (79) | 49.48 (28.31 to 79.82) | 17.62 | 31.87 | 121 (128) | 53.7 (29.37 to 91.06) | 11.65 | 42.05 | 113 (115) | 52.11 (30.96 to 78.88) | 6.27 | 45.84 | 149 (150) |
| Ethiopia | 0.358823295 | 312.37 (196.07 to 555.16) | 152.73 | 159.63 | 122 (131) | 157.37 (100.84 to 240.44) | 52.88 | 104.48 | 173 (177) | 78.37 (44.34 to 134.63) | 19.84 | 58.53 | 142 (150) | 45.65 (24.15 to 99.94) | 10.03 | 35.63 | 123 (133) |
| Fiji | 0.675051631 | 529.11 (382.88 to 706.36) | 125.41 | 403.69 | 187 (180) | 34.94 (22.67 to 49.47) | 8.05 | 26.89 | 106 (100) | 33.69 (17.83 to 56.55) | 9.44 | 24.25 | 81 (85) | 88.39 (58.03 to 127.35) | 3.9 | 84.49 | 196 (196) |
| Finland | 0.859831368 | 89.5 (70.08 to 109.64) | 54.67 | 34.83 | 36 (25) | 9.56 (7.64 to 11.7) | 2.77 | 6.79 | 31 (22) | 17.13 (13.5 to 22.89) | 3.13 | 14 | 50 (36) | 43.6 (38.29 to 49) | 2.73 | 40.87 | 136 (130) |
| France | 0.838364875 | 84.23 (66.89 to 106.83) | 54.89 | 29.34 | 30 (20) | 9.08 (7.18 to 11.01) | 3.15 | 5.93 | 24 (19) | 19.06 (14.85 to 23.91) | 3.32 | 15.73 | 59 (48) | 28.51 (24.42 to 32.54) | 2.73 | 25.78 | 92 (86) |
| Gabon | 0.634691393 | 187.57 (121.28 to 311.44) | 147.63 | 39.94 | 42 (73) | 94.33 (52.32 to 154.4) | 9.26 | 85.07 | 161 (156) | 62.15 (34.14 to 102.68) | 10.55 | 51.6 | 128 (128) | 35.86 (20.61 to 62.17) | 4.08 | 31.78 | 116 (114) |
| Gambia | 0.40971416 | 312.51 (216.45 to 432.46) | 151.78 | 160.73 | 123 (132) | 97.27 (68.49 to 141.27) | 38.81 | 58.46 | 155 (157) | 72.61 (42.9 to 124.68) | 17.54 | 55.08 | 137 (142) | 57.4 (32.05 to 104.11) | 8.56 | 48.84 | 156 (160) |
| Georgia | 0.732473604 | 238.31 (181.92 to 299.56) | 98.64 | 139.68 | 108 (95) | 13.84 (10.3 to 18.33) | 5.7 | 8.14 | 38 (38) | 45.43 (32.34 to 60.78) | 8.3 | 37.13 | 105 (98) | 10.48 (7.96 to 14.12) | 2.72 | 7.76 | 16 (14) |
| Germany | 0.902957091 | 95.94 (79.59 to 113.49) | 43.59 | 52.35 | 53 (31) | 16.2 (13.08 to 19.83) | 2.67 | 13.54 | 67 (50) | 18.03 (15.11 to 22.28) | 3.15 | 14.88 | 53 (41) | 34.18 (30.1 to 38.44) | 2.74 | 31.45 | 113 (107) |
| Ghana | 0.56493039 | 291.82 (191.48 to 391.88) | 151.06 | 140.76 | 110 (119) | 113.42 (70.41 to 163.48) | 17.16 | 96.26 | 164 (164) | 91.32 (56 to 156.07) | 13.49 | 77.84 | 168 (168) | 56.44 (37.79 to 87.29) | 6.53 | 49.91 | 160 (158) |
| Greece | 0.791854408 | 128.14 (104.57 to 151.92) | 56 | 72.14 | 69 (49) | 20.19 (15.99 to 24.4) | 3.24 | 16.95 | 78 (68) | 18.25 (14.04 to 24.81) | 3.26 | 14.99 | 55 (43) | 40.09 (34.52 to 47.68) | 2.73 | 37.36 | 128 (121) |
| Greenland | 0.826210336 | 97.77 (68.97 to 155.98) | 56.81 | 40.96 | 44 (32) | 26.06 (14.76 to 44.65) | 3.24 | 22.82 | 99 (79) | 31.11 (18.98 to 52.3) | 3.29 | 27.82 | 84 (79) | 21.35 (14.4 to 31.66) | 2.74 | 18.61 | 60 (53) |
| Grenada | 0.668993028 | 337.87 (274.27 to 414.9) | 128.92 | 208.95 | 151 (149) | 29.27 (22.97 to 37.14) | 8.45 | 20.82 | 92 (88) | 30.71 (22.11 to 42.18) | 9.55 | 21.17 | 73 (78) | 23.67 (19.75 to 28.99) | 4.01 | 19.66 | 62 (65) |
| Guam | 0.803982203 | 174.58 (133.09 to 233.89) | 54.71 | 119.87 | 96 (69) | 31.61 (20.83 to 45.54) | 3.06 | 28.55 | 114 (94) | 10.01 (4.9 to 38) | 3.45 | 6.56 | 21 (11) | 46.44 (20.32 to 73.74) | 2.73 | 43.71 | 144 (136) |
| Guatemala | 0.539972424 | 362.32 (264.97 to 470.86) | 152.65 | 209.67 | 152 (156) | 64.21 (46.4 to 86.58) | 26.81 | 37.4 | 134 (147) | 89.42 (65.07 to 130.14) | 13.69 | 75.73 | 164 (166) | 51.64 (34.3 to 74.84) | 6.51 | 45.13 | 148 (148) |
| Guinea | 0.336401293 | 600.33 (355.68 to 860.87) | 170.77 | 429.56 | 189 (186) | 227.76 (153.35 to 317.87) | 76.92 | 150.84 | 191 (198) | 139.52 (84.83 to 208.17) | 20.44 | 119.08 | 195 (195) | 74.23 (39.83 to 155.04) | 10.47 | 63.76 | 182 (184) |
| Guinea-Bissau | 0.353109621 | 427.01 (270.75 to 591.46) | 160.03 | 266.98 | 168 (169) | 186.13 (117.29 to 276.07) | 71.55 | 114.58 | 177 (187) | 92.85 (57.68 to 154.65) | 20.18 | 72.67 | 163 (169) | 79 (38.24 to 157.54) | 10.29 | 68.71 | 188 (188) |
| Guyana | 0.650812335 | 329.57 (239.39 to 441.46) | 149.98 | 179.59 | 141 (145) | 31.18 (22.94 to 42.2) | 9.27 | 21.91 | 97 (93) | 77.78 (51.91 to 110.17) | 9.85 | 67.93 | 158 (149) | 9.27 (6.64 to 12.73) | 4.22 | 5.05 | 8 (10) |
| Haiti | 0.448278285 | 1093.68 (595.59 to 1943.45) | 152.56 | 941.12 | 202 (202) | 153.24 (85.87 to 278.76) | 28.97 | 124.26 | 181 (175) | 169.4 (72.99 to 316.74) | 15.92 | 153.49 | 203 (203) | 97.65 (34.16 to 219.54) | 6.59 | 91.06 | 199 (198) |
| Honduras | 0.513037248 | 288.13 (203.22 to 416.74) | 146.33 | 141.8 | 111 (116) | 52.7 (31.46 to 81.54) | 29.18 | 23.53 | 100 (132) | 69.34 (42.51 to 105.33) | 13.68 | 55.66 | 138 (137) | 26.55 (17.46 to 38.87) | 6.5 | 20.05 | 63 (77) |
| Hungary | 0.790754768 | 108.79 (84.3 to 132.51) | 60.89 | 47.9 | 49 (39) | 9.43 (7.04 to 12.5) | 3.24 | 6.19 | 26 (21) | 17.48 (12.5 to 24.87) | 3.25 | 14.23 | 52 (40) | 32.08 (24.03 to 41.24) | 2.72 | 29.36 | 106 (100) |
| Iceland | 0.87636168 | 80.87 (62.93 to 99.3) | 48.37 | 32.5 | 33 (18) | 7.55 (6.01 to 9.61) | 2.67 | 4.88 | 18 (8) | 15.07 (11.55 to 20.36) | 3.15 | 11.92 | 37 (28) | 41.48 (33.77 to 49.16) | 2.74 | 38.74 | 131 (124) |
| India | 0.575401649 | 321.95 (239.51 to 447.86) | 154.12 | 167.83 | 131 (140) | 53.45 (36.86 to 79.86) | 16.95 | 36.51 | 132 (135) | 60.96 (35.19 to 102.9) | 13.38 | 47.58 | 124 (125) | 17.78 (10.74 to 32.17) | 6.34 | 11.45 | 31 (39) |
| Indonesia | 0.656868336 | 317.12 (227.69 to 423.92) | 139.21 | 177.91 | 139 (138) | 64.07 (37.12 to 93.25) | 9.25 | 54.82 | 153 (146) | 53.49 (29.7 to 95.31) | 9.74 | 43.75 | 118 (113) | 19.12 (13.2 to 27.7) | 4.11 | 15 | 45 (43) |
| Iran (Islamic Republic of) | 0.697207398 | 162.84 (121.28 to 218.59) | 113.9 | 48.94 | 50 (63) | 19.41 (14.82 to 23.9) | 7.8 | 11.61 | 54 (67) | 22.07 (12.76 to 32.48) | 9.11 | 12.96 | 44 (59) | 23.1 (17.44 to 30.87) | 2.74 | 20.36 | 65 (60) |
| Iraq | 0.662626231 | 426.76 (307.38 to 593.5) | 142.7 | 284.06 | 172 (168) | 39.61 (28.68 to 53.06) | 9.24 | 30.36 | 118 (109) | 51.76 (34.31 to 82.49) | 9.57 | 42.18 | 114 (109) | 19.96 (11.57 to 28.97) | 4.19 | 15.78 | 50 (46) |
| Ireland | 0.87375385 | 79.56 (63.75 to 98.82) | 48.4 | 31.16 | 31 (16) | 29.99 (23.89 to 37.8) | 2.71 | 27.28 | 108 (90) | 18.24 (14.68 to 23.09) | 3.2 | 15.03 | 56 (42) | 39.14 (32.16 to 46.26) | 2.73 | 36.41 | 125 (119) |
| Israel | 0.809011652 | 75.95 (60.96 to 92.12) | 56.88 | 19.08 | 21 (14) | 15.48 (11.92 to 19.41) | 3.09 | 12.39 | 59 (45) | 13.82 (10.92 to 18.11) | 3.35 | 10.47 | 33 (23) | 22.81 (18.86 to 27.07) | 2.75 | 20.05 | 64 (59) |
| Italy | 0.805773534 | 94.14 (75.3 to 111.67) | 54.76 | 39.37 | 41 (29) | 7.86 (5.79 to 9.87) | 3.12 | 4.74 | 16 (13) | 16.82 (12.56 to 21.67) | 3.24 | 13.58 | 46 (34) | 24.24 (20.62 to 28.64) | 2.73 | 21.51 | 73 (69) |
| Jamaica | 0.683263064 | 234.84 (174.43 to 310.55) | 124.96 | 109.87 | 92 (92) | 21.56 (15.86 to 28.86) | 7.97 | 13.59 | 68 (71) | 72.15 (52.36 to 99.28) | 9.14 | 63.01 | 149 (141) | 23.7 (17.84 to 31.41) | 3.04 | 20.66 | 70 (66) |
| Japan | 0.871241813 | 85.99 (65.86 to 107.35) | 48.4 | 37.59 | 40 (21) | 8.61 (6.83 to 10.64) | 2.87 | 5.74 | 22 (14) | 15.49 (11.92 to 19.73) | 3.39 | 12.1 | 38 (29) | 14.51 (11.83 to 17.51) | 2.72 | 11.79 | 35 (28) |
| Jordan | 0.725307227 | 333.62 (262.11 to 445.53) | 96.89 | 236.72 | 162 (147) | 21.35 (16.11 to 27.95) | 5.24 | 16.11 | 77 (69) | 65.46 (44.51 to 93.92) | 7.11 | 58.34 | 141 (132) | 50.07 (37.13 to 64.87) | 2.73 | 47.34 | 151 (144) |
| Kazakhstan | 0.725144495 | 336.23 (273.61 to 408.94) | 99.05 | 237.18 | 163 (148) | 16.65 (12.63 to 22.03) | 5.23 | 11.42 | 53 (54) | 60.78 (44.99 to 84.17) | 6.9 | 53.88 | 134 (124) | 12.66 (8.52 to 19.39) | 2.73 | 9.93 | 27 (23) |
| Kenya | 0.523768077 | 167.94 (98.34 to 316.61) | 152.7 | 15.24 | 16 (65) | 115.91 (67.51 to 185.62) | 29.68 | 86.23 | 162 (166) | 60.96 (36.16 to 117.63) | 13.63 | 47.33 | 122 (126) | 31.57 (20.49 to 58.07) | 6.44 | 25.14 | 87 (97) |
| Kiribati | 0.527186583 | 549.16 (249.15 to 785.76) | 152.95 | 396.2 | 186 (184) | 86.09 (36.33 to 136.05) | 28.94 | 57.15 | 154 (154) | 21.15 (9.71 to 51.36) | 13.69 | 7.46 | 23 (54) | 107.11 (34.5 to 170.15) | 6.54 | 100.56 | 200 (200) |
| Kuwait | 0.846651055 | 239.48 (197.07 to 292.38) | 54.64 | 184.84 | 142 (97) | 18.76 (15.26 to 23.21) | 3.07 | 15.69 | 75 (65) | 57.87 (43.65 to 76.4) | 3.37 | 54.49 | 135 (121) | 25.79 (18.54 to 33.12) | 2.73 | 23.07 | 78 (74) |
| Kyrgyzstan | 0.603979328 | 355.11 (289.76 to 425.62) | 146.76 | 208.36 | 150 (153) | 33.36 (22.09 to 49.3) | 11.63 | 21.73 | 95 (96) | 91.18 (73.79 to 112.59) | 10.72 | 80.46 | 169 (167) | 7.29 (5.74 to 10.06) | 5.99 | 1.3 | 5 (4) |
| Lao People's Democratic Republic | 0.489136091 | 845.73 (535.03 to 1211.07) | 150.73 | 695 | 196 (196) | 126.79 (72.48 to 193.79) | 29.55 | 97.24 | 166 (169) | 115.51 (51.07 to 217.51) | 14.3 | 101.22 | 186 (186) | 21.93 (12.89 to 33.6) | 6.53 | 15.41 | 49 (55) |
| Latvia | 0.830663516 | 89 (73.02 to 115.54) | 56.3 | 32.7 | 34 (24) | 9.59 (7.23 to 12.27) | 3.31 | 6.28 | 27 (23) | 9.17 (5.72 to 16.46) | 3.2 | 5.96 | 16 (7) | 24.95 (20.19 to 30.5) | 2.73 | 22.22 | 74 (70) |
| Lebanon | 0.744746351 | 152.48 (108.4 to 216.17) | 65.06 | 87.42 | 83 (58) | 17.84 (12.84 to 23.97) | 5.03 | 12.81 | 61 (61) | 38.08 (24.58 to 58.05) | 4.27 | 33.81 | 98 (88) | 16.4 (11.63 to 22.74) | 2.76 | 13.64 | 41 (34) |
| Lesotho | 0.510393066 | 263.94 (161.02 to 398.09) | 152.59 | 111.35 | 93 (107) | 76.39 (46.89 to 123.22) | 29.51 | 46.88 | 145 (150) | 57.68 (30.59 to 90.83) | 14.03 | 43.65 | 117 (120) | 60 (33.07 to 92.86) | 6.43 | 53.57 | 165 (164) |
| Liberia | 0.352442452 | 442.46 (273.75 to 647.58) | 158.15 | 284.31 | 173 (172) | 157.22 (104.64 to 223.51) | 52.73 | 104.49 | 174 (176) | 116.44 (70.89 to 176.91) | 20.17 | 96.27 | 185 (187) | 70.82 (38.18 to 146.53) | 10.2 | 60.63 | 177 (181) |
| Libya | 0.725771399 | 615.72 (413.41 to 863.68) | 96.09 | 519.63 | 194 (188) | 104.24 (64.62 to 154.35) | 5.48 | 98.77 | 168 (158) | 101.49 (52.53 to 173.93) | 7.96 | 93.53 | 181 (176) | 117.32 (50.07 to 292.48) | 2.72 | 114.6 | 201 (201) |
| Lithuania | 0.856484049 | 118.32 (92.82 to 148) | 54.71 | 63.61 | 64 (43) | 12.4 (9.32 to 15.84) | 2.79 | 9.61 | 46 (34) | 19.95 (14.34 to 28.44) | 3.26 | 16.69 | 64 (53) | 14.44 (11.37 to 18.21) | 2.76 | 11.68 | 33 (26) |
| Luxembourg | 0.884428955 | 52.26 (39.96 to 68.98) | 47.39 | 4.86 | 9 (3) | 9.05 (7.13 to 11.16) | 2.67 | 6.39 | 28 (18) | 7.22 (5.4 to 13.07) | 3.25 | 3.97 | 13 (4) | 10.99 (8.96 to 13.38) | 2.73 | 8.26 | 18 (15) |
| Madagascar | 0.400246943 | 307.24 (179.46 to 537.86) | 157.64 | 149.6 | 113 (126) | 208.27 (118.51 to 314.11) | 38.42 | 169.85 | 198 (192) | 85.43 (41.74 to 144.97) | 17.53 | 67.9 | 157 (160) | 51.92 (22.47 to 116.58) | 8.89 | 43.03 | 143 (149) |
| Malawi | 0.384553634 | 310.36 (191.21 to 513.09) | 152.67 | 157.69 | 120 (129) | 209.62 (118.09 to 362.78) | 43.37 | 166.25 | 197 (194) | 87.85 (53.26 to 143.86) | 17.54 | 70.31 | 161 (165) | 57.21 (29.99 to 114.67) | 9.05 | 48.16 | 155 (159) |
| Malaysia | 0.742523828 | 153.03 (121.21 to 192.36) | 68.47 | 84.56 | 81 (59) | 30.27 (20.96 to 42.9) | 5.11 | 25.16 | 103 (92) | 16.71 (9.07 to 35.72) | 5.29 | 11.42 | 35 (33) | 9.37 (7.13 to 11.93) | 2.73 | 6.64 | 13 (11) |
| Maldives | 0.650886627 | 228.45 (170.49 to 313.23) | 150.26 | 78.19 | 73 (87) | 36.91 (22.99 to 56.74) | 9.24 | 27.67 | 110 (102) | 52.28 (31.64 to 95.55) | 9.87 | 42.41 | 115 (110) | 35.66 (25.65 to 49.55) | 4.07 | 31.6 | 115 (113) |
| Mali | 0.268579941 | 689.03 (387.47 to 1028.02) | 386.59 | 302.44 | 177 (193) | 231.7 (139.38 to 351.22) | 99.06 | 132.63 | 185 (201) | 159.79 (86.98 to 240.27) | 51.39 | 108.4 | 190 (200) | 85.99 (45.17 to 151.37) | 18.28 | 67.71 | 187 (193) |
| Malta | 0.801585034 | 136.66 (110.81 to 167.83) | 55.27 | 81.39 | 76 (53) | 34.56 (27.16 to 43.25) | 3.14 | 31.42 | 119 (99) | 37.05 (28.52 to 47.23) | 3.11 | 33.95 | 99 (87) | 43.72 (35.59 to 53.17) | 2.72 | 41 | 138 (131) |
| Marshall Islands | 0.574091128 | 339.83 (238.42 to 484.85) | 147.31 | 192.52 | 145 (150) | 55.57 (31.28 to 92) | 17.7 | 37.88 | 135 (137) | 14.84 (6.9 to 42.55) | 13.48 | 1.35 | 9 (27) | 62.19 (30.64 to 99.18) | 6.44 | 55.75 | 171 (169) |
| Mauritania | 0.4989451 | 248.52 (175.25 to 341.75) | 151.19 | 97.33 | 87 (101) | 82.13 (51 to 124.79) | 29.14 | 53 | 151 (153) | 71.32 (37.16 to 122.35) | 14.41 | 56.91 | 140 (140) | 55.75 (31.2 to 94.4) | 6.54 | 49.2 | 157 (155) |
| Mauritius | 0.718260446 | 328.05 (269.85 to 427.63) | 86.42 | 241.63 | 164 (143) | 27.11 (20.27 to 38.48) | 7.21 | 19.9 | 89 (82) | 60.67 (48.63 to 73.73) | 8.69 | 51.98 | 130 (123) | 9.05 (7.13 to 11.92) | 2.74 | 6.31 | 11 (8) |
| Mexico | 0.664575304 | 447.62 (348.34 to 565.01) | 141.66 | 305.96 | 178 (175) | 37.43 (29.24 to 47.39) | 8.77 | 28.67 | 115 (103) | 92.91 (68.36 to 120.71) | 9.58 | 83.33 | 173 (170) | 39.65 (31.16 to 51.06) | 4.09 | 35.56 | 122 (120) |
| Micronesia (Federated States of) | 0.587534967 | 280.48 (212.92 to 376.18) | 154.14 | 126.35 | 99 (113) | 42.92 (24.36 to 71.2) | 16.67 | 26.26 | 104 (112) | 13.12 (6.6 to 39.57) | 10.98 | 2.14 | 10 (21) | 55.9 (31.75 to 85.92) | 6.11 | 49.79 | 159 (157) |
| Monaco | 0.908262831 | 123.83 (93.13 to 162.24) | 40.35 | 83.49 | 79 (48) | 10.75 (7.41 to 14.69) | 2.65 | 8.1 | 37 (29) | 19.55 (10.16 to 33.11) | 3.27 | 16.28 | 61 (51) | 30.16 (21.63 to 41.97) | 2.73 | 27.43 | 99 (93) |
| Mongolia | 0.617621565 | 286.17 (206.28 to 369.31) | 151.41 | 134.75 | 102 (115) | 12.62 (8.36 to 18.14) | 9.3 | 3.33 | 10 (36) | 87.03 (55.31 to 121.91) | 10.56 | 76.47 | 165 (161) | 36.51 (21.36 to 53.68) | 5.97 | 30.54 | 110 (117) |
| Montenegro | 0.795800584 | 57.05 (39.82 to 85.68) | 55.16 | 1.89 | 7 (5) | 3.03 (2.2 to 4.14) | 3.03 | 0 | 2.5 (2) | 9.26 (4.67 to 18.38) | 3.17 | 6.09 | 18 (9) | 20.18 (13.56 to 29.81) | 2.74 | 17.44 | 54 (48) |
| Morocco | 0.562698301 | 181.56 (109.6 to 369.08) | 154.53 | 27.04 | 29 (72) | 22.2 (13.37 to 46.33) | 15.43 | 6.77 | 30 (73) | 23.99 (13.63 to 48.73) | 13.66 | 10.33 | 31 (65) | 22.76 (13.56 to 38.64) | 6.52 | 16.24 | 51 (58) |
| Mozambique | 0.326462614 | 418.4 (243.42 to 741.79) | 171.79 | 246.61 | 166 (167) | 230.57 (139.73 to 356.23) | 75.05 | 155.53 | 194 (200) | 109.37 (53.8 to 192.14) | 20.74 | 88.63 | 177 (182) | 63.9 (32.79 to 143.04) | 10.73 | 53.18 | 164 (171) |
| Myanmar | 0.53390084 | 880.79 (545.06 to 1226.29) | 153.55 | 727.24 | 198 (198) | 130.69 (78.44 to 196.01) | 29.11 | 101.58 | 171 (171) | 126.11 (64.19 to 204.68) | 13.63 | 112.48 | 193 (189) | 40.16 (21.94 to 78.4) | 6.44 | 33.71 | 120 (122) |
| Namibia | 0.617564872 | 163.67 (110.03 to 237.38) | 151.82 | 11.85 | 15 (64) | 37.72 (22.84 to 56.32) | 9.28 | 28.44 | 111 (104) | 39.1 (17.77 to 69.02) | 10.58 | 28.52 | 86 (90) | 42.95 (24.08 to 65.39) | 6.01 | 36.94 | 127 (128) |
| Nauru | 0.625177834 | 446.99 (286.87 to 641.66) | 148.06 | 298.94 | 176 (173) | 57.8 (33.33 to 85.61) | 9.39 | 48.41 | 149 (143) | 19.41 (9.08 to 48.84) | 10.57 | 8.83 | 28 (50) | 88.16 (46.23 to 143.38) | 5.91 | 82.24 | 195 (195) |
| Nepal | 0.433174635 | 169.69 (96 to 358.14) | 146.32 | 23.37 | 25 (66) | 28.88 (16.12 to 61.39) | 28.88 | 0 | 2.5 (86) | 28.59 (14 to 66.82) | 16.13 | 12.46 | 42 (75) | 7.78 (3.81 to 22.19) | 6.55 | 1.23 | 3 (5) |
| Netherlands | 0.888464256 | 75.75 (62.83 to 93.41) | 39.21 | 36.54 | 37 (13) | 18.34 (15.35 to 21.73) | 2.66 | 15.68 | 74 (64) | 19.76 (16.63 to 23.83) | 3.43 | 16.32 | 62 (52) | 48.63 (42.26 to 54.4) | 2.73 | 45.9 | 150 (140) |
| New Zealand | 0.849442499 | 80 (65.27 to 100.42) | 55.02 | 24.98 | 26 (17) | 16.45 (13.56 to 19.75) | 3.02 | 13.43 | 66 (52) | 17.31 (13.73 to 23.1) | 3.33 | 13.99 | 49 (38) | 32.82 (27.07 to 37.81) | 2.72 | 30.1 | 108 (103) |
| Nicaragua | 0.523958472 | 260.11 (180.69 to 364.38) | 153.61 | 106.5 | 89 (105) | 46.88 (32.33 to 66.82) | 29.14 | 17.74 | 81 (121) | 107.64 (67.58 to 162.74) | 13.67 | 93.97 | 182 (181) | 16.49 (10.17 to 22.94) | 6.46 | 10.03 | 28 (35) |
| Niger | 0.168072774 | 482.46 (221.6 to 731.27) | 424.28 | 58.18 | 57 (178) | 111.27 (56.05 to 181.09) | 111.27 | 0 | 2.5 (163) | 87.51 (47.29 to 133.32) | 86.32 | 1.19 | 8 (163) | 49.43 (23.2 to 103.14) | 49.43 | 0.01 | 1 (142) |
| Nigeria | 0.503390833 | 538.04 (298.04 to 805.65) | 154.9 | 383.14 | 185 (182) | 211.33 (125.36 to 307.16) | 29.78 | 181.55 | 201 (195) | 161.37 (86.85 to 234.57) | 14.14 | 147.24 | 202 (201) | 77 (41.84 to 177.92) | 6.48 | 70.51 | 190 (187) |
| Niue | 0.72622205 | 1026.71 (811.13 to 1278.38) | 86.24 | 940.47 | 201 (201) | 104.39 (72.7 to 142.32) | 5.39 | 99 | 169 (159) | 54.86 (28.38 to 156.24) | 8.01 | 46.85 | 121 (117) | 244.11 (145.21 to 373.63) | 2.73 | 241.38 | 203 (203) |
| North Macedonia | 0.750629703 | 106.33 (80.96 to 144.41) | 65.93 | 40.39 | 43 (38) | 14.66 (10.26 to 20.49) | 5.01 | 9.65 | 47 (40) | 26.15 (15.69 to 40.55) | 4.14 | 22.01 | 75 (70) | 14.49 (10.55 to 19.79) | 2.74 | 11.74 | 34 (27) |
| Northern Mariana Islands | 0.771535213 | 121.22 (91.64 to 159.01) | 71.14 | 50.08 | 52 (44) | 49.09 (35.05 to 66.73) | 4.41 | 44.68 | 143 (127) | 3.27 (1.68 to 8.53) | 3.09 | 0.17 | 5 (1) | 24.96 (9.8 to 39.59) | 2.72 | 22.24 | 75 (71) |
| Norway | 0.91613281 | 68.34 (52.27 to 100.36) | 41.98 | 26.37 | 28 (11) | 9.32 (7.29 to 11.49) | 2.66 | 6.66 | 29 (20) | 13.04 (8.31 to 17.9) | 3.3 | 9.73 | 30 (20) | 25.76 (22.54 to 28.88) | 2.74 | 23.02 | 77 (73) |
| Oman | 0.773391602 | 198.07 (154.77 to 260.75) | 57.73 | 140.35 | 109 (77) | 122.18 (85.33 to 166.95) | 4.35 | 117.83 | 178 (168) | 23.02 (12.51 to 57.1) | 3.45 | 19.58 | 69 (61) | 11.5 (8.86 to 14.89) | 2.72 | 8.78 | 21 (17) |
| Pakistan | 0.504028689 | 364.84 (225.32 to 534.24) | 151.12 | 213.72 | 154 (158) | 53.11 (25.13 to 98.53) | 28.93 | 24.18 | 102 (133) | 60.06 (29.59 to 100.7) | 14.33 | 45.74 | 119 (122) | 23.34 (13.16 to 44.91) | 6.48 | 16.86 | 52 (63) |
| Palau | 0.754046931 | 289.49 (207.54 to 380.82) | 67.38 | 222.1 | 156 (117) | 44.43 (21.9 to 68.48) | 5.04 | 39.4 | 137 (115) | 17.32 (9.67 to 44.28) | 4.15 | 13.17 | 45 (39) | 79.15 (42.62 to 115.18) | 2.74 | 76.42 | 192 (189) |
| Palestine | 0.631011665 | 285.05 (215.04 to 389.41) | 149.45 | 135.6 | 104 (114) | 15.39 (11.41 to 20.28) | 9.36 | 6.03 | 25 (43) | 32.64 (20.01 to 57.21) | 10.51 | 22.13 | 76 (82) | 35.31 (23.42 to 50.23) | 4.94 | 30.38 | 109 (110) |
| Panama | 0.708864828 | 480.15 (373.69 to 607.28) | 114.59 | 365.56 | 183 (177) | 31.66 (24.5 to 40.69) | 7.8 | 23.86 | 101 (95) | 104.63 (77.18 to 135.78) | 8.98 | 95.65 | 184 (177) | 30.66 (22.83 to 39.75) | 2.76 | 27.91 | 101 (94) |
| Papua New Guinea | 0.417797443 | 868.19 (362.8 to 1316.05) | 146.7 | 721.49 | 197 (197) | 152.02 (76.07 to 248.91) | 33.43 | 118.59 | 179 (174) | 29.7 (11.42 to 70.17) | 17.54 | 12.16 | 40 (77) | 133.57 (40.49 to 249.07) | 7.77 | 125.8 | 202 (202) |
| Paraguay | 0.635718099 | 307.39 (201.83 to 448.43) | 146.19 | 161.2 | 124 (127) | 55.65 (32.85 to 87.9) | 9.25 | 46.39 | 144 (138) | 47.47 (24.99 to 84.92) | 10.43 | 37.03 | 104 (102) | 40.87 (25 to 60.53) | 4.47 | 36.4 | 124 (123) |
| Peru | 0.662054037 | 260.36 (171.67 to 363.51) | 141.25 | 119.11 | 95 (106) | 37.79 (25.47 to 54.87) | 9.24 | 28.55 | 113 (105) | 63.34 (38.57 to 94.76) | 9.59 | 53.75 | 133 (130) | 27.29 (16.39 to 40.1) | 4.15 | 23.14 | 80 (80) |
| Philippines | 0.651219329 | 374.05 (301.48 to 480.51) | 147.96 | 226.09 | 158 (159) | 56.89 (45.07 to 73.21) | 9.24 | 47.65 | 146 (141) | 62.31 (45.68 to 87.51) | 10.19 | 52.12 | 131 (129) | 22.44 (16.79 to 27.31) | 4.09 | 18.36 | 58 (57) |
| Poland | 0.812042809 | 153.3 (121.68 to 183.46) | 56.51 | 96.79 | 86 (60) | 11.23 (8.53 to 14.12) | 3.08 | 8.16 | 39 (30) | 23.11 (16.58 to 30.99) | 3.26 | 19.85 | 70 (62) | 8.73 (6.68 to 10.44) | 2.73 | 6 | 10 (7) |
| Portugal | 0.744151851 | 88.34 (70.77 to 104.88) | 73.01 | 15.33 | 17 (22) | 10.04 (7.69 to 12.38) | 5.16 | 4.88 | 17 (25) | 16.49 (11.95 to 21.88) | 4.36 | 12.13 | 39 (32) | 23.49 (19.77 to 27.87) | 2.74 | 20.75 | 71 (64) |
| Puerto Rico | 0.825525847 | 176.54 (140.61 to 212.35) | 56.59 | 119.96 | 97 (70) | 30.14 (22.73 to 37.79) | 3.29 | 26.85 | 105 (91) | 43.68 (30.77 to 57.26) | 3.11 | 40.57 | 112 (96) | 28.32 (23.4 to 34.59) | 2.74 | 25.59 | 91 (85) |
| Qatar | 0.846860584 | 114.64 (84.9 to 151.22) | 55.62 | 59.02 | 60 (41) | 16.35 (12.31 to 21.4) | 3 | 13.34 | 64 (51) | 10.75 (5.97 to 27.21) | 3.3 | 7.45 | 22 (14) | 20.95 (15.22 to 28.64) | 2.74 | 18.22 | 56 (50) |
| Republic of Korea | 0.886675267 | 76.09 (59.63 to 106.49) | 44.59 | 31.49 | 32 (15) | 7.63 (5.87 to 9.79) | 2.67 | 4.96 | 19 (10) | 12.85 (8.29 to 23.43) | 4.06 | 8.79 | 27 (19) | 15.07 (11.52 to 19.46) | 2.73 | 12.35 | 37 (30) |
| Republic of Moldova | 0.732214875 | 258.95 (195.62 to 338.84) | 95.28 | 163.66 | 127 (102) | 26.71 (20.04 to 36.24) | 5.18 | 21.53 | 94 (80) | 85.01 (61.68 to 118.47) | 8.33 | 76.68 | 166 (159) | 11.19 (8.53 to 14.71) | 2.74 | 8.45 | 19 (16) |
| Romania | 0.768453864 | 188.87 (158.38 to 219.85) | 57.36 | 131.52 | 100 (74) | 16.99 (13.53 to 20.26) | 4.12 | 12.87 | 62 (59) | 51.42 (40.32 to 62.39) | 3.37 | 48.05 | 126 (108) | 6.24 (4.86 to 7.97) | 2.73 | 3.52 | 7 (3) |
| Russian Federation | 0.808536005 | 133.98 (108.56 to 165.61) | 54.68 | 79.29 | 74 (51) | 10.14 (8.05 to 12.47) | 3.11 | 7.03 | 32 (26) | 31.44 (22.44 to 42.13) | 3.21 | 28.23 | 85 (80) | 12.19 (10.05 to 14.51) | 2.73 | 9.46 | 24 (21) |
| Rwanda | 0.435588706 | 295.97 (187.5 to 483.43) | 151 | 144.97 | 112 (121) | 166.21 (98.8 to 272.92) | 29.13 | 137.07 | 188 (181) | 81.07 (42.49 to 137.47) | 15.9 | 65.17 | 152 (156) | 62.65 (29.74 to 131.09) | 6.6 | 56.05 | 173 (170) |
| Saint Kitts and Nevis | 0.754987055 | 269.61 (209.12 to 346.83) | 73.63 | 195.98 | 146 (110) | 33.48 (25.51 to 44.24) | 5.01 | 28.47 | 112 (97) | 53.69 (40.62 to 71.41) | 4.45 | 49.25 | 127 (114) | 25.03 (18.99 to 33.81) | 2.75 | 22.27 | 76 (72) |
| Saint Lucia | 0.672509735 | 234.78 (173.21 to 316.35) | 127.53 | 107.26 | 90 (91) | 38.16 (27.97 to 51.78) | 8.03 | 30.13 | 117 (107) | 47.76 (33.24 to 73.07) | 9.54 | 38.22 | 108 (103) | 28.57 (21.74 to 37.05) | 4.05 | 24.52 | 85 (87) |
| Saint Vincent and the Grenadines | 0.637195963 | 231.22 (179.76 to 295.78) | 149.34 | 81.88 | 77 (89) | 43.18 (33 to 57.08) | 9.25 | 33.93 | 125 (113) | 61.71 (45.64 to 83.77) | 9.87 | 51.84 | 129 (127) | 33.37 (26.28 to 41.13) | 4.06 | 29.31 | 105 (104) |
| Samoa | 0.593392769 | 238.51 (172.29 to 325.19) | 154.29 | 84.23 | 80 (96) | 33.54 (18.54 to 53.62) | 15.38 | 18.16 | 82 (98) | 12.11 (6.02 to 37.49) | 11.01 | 1.1 | 7 (18) | 50.93 (24.64 to 83.89) | 6.07 | 44.86 | 146 (147) |
| San Marino | 0.888005474 | 34.86 (23.01 to 49.72) | 34.86 | 0 | 2.5 (1) | 9.87 (6.27 to 14.45) | 2.67 | 7.2 | 34 (24) | 9.69 (5.39 to 15.65) | 3.14 | 6.55 | 20 (10) | 11.53 (8.39 to 16.12) | 2.74 | 8.79 | 22 (18) |
| Sao Tome and Principe | 0.505413747 | 200.71 (125.38 to 321.24) | 147.19 | 53.51 | 54 (78) | 62.11 (36.5 to 116.47) | 29.19 | 32.92 | 122 (145) | 53.23 (20.5 to 102.59) | 14.18 | 39.05 | 109 (112) | 35.1 (20.08 to 57.98) | 6.44 | 28.66 | 103 (109) |
| Saudi Arabia | 0.815143493 | 122.35 (81.93 to 186.27) | 54.99 | 67.35 | 67 (46) | 11.62 (8.47 to 15.79) | 3.05 | 8.57 | 41 (31) | 14.62 (6.83 to 35.47) | 3.23 | 11.39 | 34 (25) | 34.29 (23.54 to 49.47) | 2.75 | 31.54 | 114 (108) |
| Senegal | 0.408054193 | 353.7 (258.14 to 476.96) | 155.55 | 198.15 | 147 (152) | 127.91 (83.21 to 178.15) | 38.75 | 89.16 | 163 (170) | 84.19 (44.59 to 151.1) | 17.55 | 66.64 | 154 (157) | 68.45 (40.03 to 114.7) | 8.5 | 59.94 | 176 (177) |
| Serbia | 0.792416294 | 123.29 (94.8 to 163.9) | 58.28 | 65.01 | 66 (47) | 7.7 (5.74 to 10.38) | 3.25 | 4.45 | 13 (12) | 21.2 (12.51 to 41.7) | 3.13 | 18.07 | 66 (55) | 17.59 (12.44 to 23.09) | 2.75 | 14.83 | 42 (36) |
| Seychelles | 0.730150775 | 357.11 (270.64 to 458.72) | 87.81 | 269.3 | 169 (154) | 55.86 (34 to 83.13) | 5.41 | 50.45 | 150 (139) | 77.08 (48.81 to 112.8) | 8.29 | 68.79 | 159 (148) | 21.08 (14.02 to 29.43) | 2.74 | 18.34 | 57 (51) |
| Sierra Leone | 0.358665881 | 660.92 (361.91 to 1003.56) | 159.23 | 501.69 | 193 (191) | 222.86 (153.2 to 309.11) | 52.65 | 170.21 | 199 (197) | 159.47 (94.62 to 229.98) | 20.17 | 139.31 | 200 (199) | 80.76 (40.57 to 183.95) | 10.29 | 70.46 | 189 (191) |
| Singapore | 0.856097766 | 63.94 (47.09 to 83.08) | 55.05 | 8.9 | 13 (10) | 16.78 (12.4 to 21.23) | 2.79 | 13.99 | 70 (56) | 10.81 (8.11 to 15.57) | 3.23 | 7.58 | 24 (15) | 8.52 (6.38 to 11.48) | 2.73 | 5.79 | 9 (6) |
| Slovakia | 0.81061053 | 141.83 (114.72 to 180.41) | 55.19 | 86.64 | 82 (54) | 10.64 (8.34 to 13.54) | 3.45 | 7.2 | 33 (28) | 27.05 (17.55 to 43.09) | 3.27 | 23.78 | 79 (72) | 17.64 (13.56 to 23.05) | 2.73 | 14.91 | 43 (37) |
| Slovenia | 0.842430731 | 63.02 (47.92 to 76.5) | 55.32 | 7.7 | 11 (7) | 6.33 (4.81 to 8.37) | 3.08 | 3.25 | 9 (4) | 9.25 (6.69 to 14.39) | 3.23 | 6.01 | 17 (8) | 20.63 (15.75 to 25.42) | 2.73 | 17.89 | 55 (49) |
| Solomon Islands | 0.429360316 | 389.37 (284.77 to 511.76) | 154.16 | 235.2 | 160 (161) | 65.23 (30.59 to 110.64) | 31.68 | 33.55 | 124 (149) | 15.73 (7.35 to 47.15) | 15.73 | 0 | 2.5 (31) | 64.89 (34.23 to 98.4) | 6.54 | 58.35 | 174 (175) |
| Somalia | 0.077688109 | 407.24 (166.24 to 846.27) | 407.24 | 0 | 2.5 (164) | 229.67 (121.38 to 384.01) | 229.67 | 0 | 2.5 (199) | 79.64 (28.06 to 156.72) | 79.64 | 0 | 2.5 (153) | 50.47 (15.25 to 150.36) | 49.97 | 0.5 | 2 (146) |
| South Africa | 0.679626598 | 179.51 (123.37 to 254.56) | 142.21 | 37.31 | 39 (71) | 40.95 (30.65 to 56.46) | 7.95 | 33 | 123 (110) | 46.66 (28.68 to 68.44) | 9.48 | 37.18 | 106 (101) | 45.56 (28.58 to 62.28) | 3.1 | 42.46 | 142 (132) |
| South Sudan | 0.278371125 | 665.83 (257.83 to 1185.27) | 394.99 | 270.83 | 170 (192) | 351.62 (194.14 to 579.97) | 85.4 | 266.23 | 203 (204) | 159.2 (43.69 to 316.63) | 48.03 | 111.17 | 191 (198) | 102.34 (25.95 to 333.45) | 14.46 | 87.88 | 197 (199) |
| Spain | 0.769283698 | 90.44 (72.67 to 107.96) | 67.29 | 23.15 | 24 (26) | 9.04 (6.86 to 11.46) | 4.05 | 4.99 | 20 (17) | 14.72 (10.55 to 19.25) | 3.28 | 11.44 | 36 (26) | 26.33 (21.26 to 33.63) | 2.72 | 23.61 | 82 (76) |
| Sri Lanka | 0.701534935 | 235.52 (172.66 to 324.25) | 124.13 | 111.39 | 94 (93) | 13.56 (10 to 18.45) | 7.73 | 5.82 | 23 (37) | 33.64 (19.79 to 54.63) | 8.96 | 24.68 | 82 (84) | 11.63 (8.02 to 15.69) | 2.74 | 8.9 | 23 (20) |
| Sudan | 0.541949735 | 936.59 (620.61 to 1297.33) | 153.44 | 783.14 | 199 (199) | 150.92 (99.84 to 222.41) | 29.09 | 121.82 | 180 (173) | 115.2 (67.87 to 182.74) | 13.62 | 101.58 | 187 (185) | 65.43 (39.54 to 106.02) | 6.54 | 58.89 | 175 (176) |
| Suriname | 0.633665739 | 314.94 (210.73 to 439.51) | 146.91 | 168.02 | 132 (134) | 80.78 (54.3 to 115.58) | 9.26 | 71.51 | 158 (152) | 152.6 (97.79 to 225.02) | 10.3 | 142.3 | 201 (197) | 29.43 (19.19 to 49.22) | 4.5 | 24.93 | 86 (89) |
| Sweden | 0.886880299 | 63.12 (50.46 to 83.72) | 44.9 | 18.22 | 19 (8) | 12.47 (9.85 to 15.15) | 2.66 | 9.81 | 48 (35) | 12.06 (9.12 to 16.85) | 3.27 | 8.78 | 26 (17) | 29.39 (25.24 to 33.56) | 2.73 | 26.66 | 94 (88) |
| Switzerland | 0.933059111 | 99.88 (83.63 to 119.45) | 41.07 | 58.81 | 59 (34) | 16.71 (13.7 to 20.16) | 2.7 | 14.01 | 71 (55) | 21.41 (17.26 to 26.23) | 3.4 | 18.01 | 65 (56) | 50.2 (43.35 to 58.15) | 2.72 | 47.48 | 152 (145) |
| Syrian Arab Republic | 0.623004075 | 328.55 (244.22 to 438.61) | 153.4 | 175.15 | 137 (144) | 36.56 (21.53 to 57.25) | 9.38 | 27.18 | 107 (101) | 43.3 (25.94 to 71.58) | 10.53 | 32.77 | 95 (95) | 55.16 (33.65 to 80.89) | 5.94 | 49.23 | 158 (154) |
| Taiwan (Province of China) | 0.874747053 | 122.21 (102.26 to 139.32) | 48.48 | 73.73 | 71 (45) | 2.67 (2.11 to 3.3) | 2.67 | 0 | 5 (1) | 18.29 (14.69 to 23.36) | 3.32 | 14.97 | 54 (44) | 14.93 (9.51 to 19.26) | 2.72 | 12.21 | 36 (29) |
| Tajikistan | 0.541511187 | 270.09 (164.37 to 544.42) | 147.28 | 122.81 | 98 (111) | 53.18 (28.87 to 90.04) | 25.62 | 27.57 | 109 (134) | 50.04 (25.66 to 89.65) | 13.68 | 36.36 | 103 (107) | 48.68 (27.74 to 92.94) | 6.51 | 42.17 | 141 (141) |
| Thailand | 0.682547933 | 197.21 (144.15 to 245.88) | 135.04 | 62.16 | 63 (76) | 15.46 (11.1 to 20.29) | 7.98 | 7.48 | 35 (44) | 21.55 (12.83 to 38.5) | 9.09 | 12.45 | 41 (57) | 11.6 (8.31 to 14.6) | 2.9 | 8.7 | 20 (19) |
| Timor-Leste | 0.444667619 | 617.7 (446.77 to 859.1) | 154.11 | 463.59 | 191 (189) | 88.28 (54.94 to 133.95) | 28.97 | 59.31 | 156 (155) | 75.46 (45.87 to 117.84) | 16 | 59.46 | 144 (144) | 21.93 (14.13 to 34.48) | 6.58 | 15.35 | 47 (54) |
| Togo | 0.408533695 | 360.21 (242.45 to 503.68) | 154.25 | 205.96 | 149 (155) | 136.36 (92.38 to 198.25) | 39.05 | 97.31 | 167 (172) | 87.67 (55.81 to 142.68) | 17.55 | 70.13 | 160 (164) | 69.57 (37.43 to 139.36) | 8.13 | 61.44 | 179 (179) |
| Tokelau | 0.686425621 | 1181.48 (842.16 to 1714.99) | 125.42 | 1056.06 | 203 (203) | 116 (58.76 to 193.65) | 7.97 | 108.03 | 175 (167) | 57.14 (19.78 to 216.01) | 9.56 | 47.58 | 123 (119) | 329.15 (171.44 to 500.56) | 3.73 | 325.42 | 204 (204) |
| Tonga | 0.626349936 | 161.93 (115.71 to 239.7) | 152.39 | 9.53 | 14 (62) | 24.71 (14.12 to 39.3) | 9.37 | 15.34 | 73 (77) | 10.25 (4.88 to 35.16) | 10.25 | 0 | 2.5 (13) | 33.68 (19.4 to 56.56) | 6.03 | 27.65 | 100 (105) |
| Trinidad and Tobago | 0.768763254 | 410.77 (309.69 to 541.3) | 57.54 | 353.23 | 181 (165) | 25.91 (19.13 to 34.45) | 4.4 | 21.51 | 93 (78) | 84.75 (61.09 to 114.5) | 3.32 | 81.43 | 171 (158) | 63.95 (45.85 to 85.57) | 2.72 | 61.23 | 178 (173) |
| Tunisia | 0.682432216 | 296.3 (222.3 to 393.36) | 118.25 | 178.05 | 140 (122) | 27.1 (18.47 to 38.81) | 8.01 | 19.09 | 87 (81) | 45.6 (29.95 to 67.81) | 9.28 | 36.32 | 102 (99) | 41.81 (26.89 to 62.39) | 3.45 | 38.35 | 130 (126) |
| Turkey | 0.712692673 | 330.78 (250.79 to 420.93) | 106.83 | 223.96 | 157 (146) | 48.52 (37.06 to 62.49) | 6.54 | 41.99 | 138 (124) | 76.43 (54.43 to 105.8) | 8.71 | 67.72 | 156 (146) | 47.12 (36.55 to 61.55) | 2.75 | 44.38 | 145 (138) |
| Turkmenistan | 0.682160776 | 570.58 (413.9 to 740.07) | 120.6 | 449.98 | 190 (185) | 50.83 (34.02 to 77.15) | 8 | 42.83 | 140 (129) | 137.87 (82.18 to 202.11) | 9.15 | 128.73 | 198 (194) | 23.83 (14.63 to 35.08) | 3.3 | 20.53 | 66 (68) |
| Tuvalu | 0.576620529 | 316.37 (226.1 to 444.3) | 146.71 | 169.67 | 135 (136) | 48.93 (29.91 to 74.18) | 17.1 | 31.83 | 120 (125) | 15.6 (7.61 to 43.91) | 11.61 | 3.99 | 14 (30) | 54.56 (29.5 to 84.02) | 6.43 | 48.13 | 154 (151) |
| Uganda | 0.423261181 | 321.75 (207.61 to 531.15) | 153.59 | 168.16 | 133 (139) | 187.45 (120.31 to 281.2) | 31.43 | 156.02 | 195 (188) | 97.4 (47.61 to 153.65) | 16.56 | 80.83 | 170 (174) | 46.25 (27.95 to 85.61) | 6.59 | 39.66 | 133 (135) |
| Ukraine | 0.760773913 | 239.71 (197.69 to 289.25) | 70.74 | 168.97 | 134 (98) | 38.81 (30.12 to 51.56) | 4.75 | 34.07 | 127 (108) | 33.56 (23.74 to 47.8) | 3.34 | 30.22 | 90 (83) | 13.73 (10.2 to 21.22) | 2.8 | 10.94 | 29 (24) |
| United Arab Emirates | 0.849317734 | 158.17 (98.65 to 212.53) | 54.76 | 103.41 | 88 (61) | 21.58 (12.35 to 31.7) | 3.13 | 18.45 | 83 (72) | 24.07 (12.23 to 41.18) | 3.75 | 20.33 | 72 (66) | 41.52 (22.11 to 75.6) | 2.72 | 38.8 | 132 (125) |
| United Kingdom | 0.859000182 | 98.42 (84.8 to 117.09) | 54.64 | 43.78 | 45 (33) | 15.99 (13.92 to 17.95) | 2.82 | 13.18 | 63 (49) | 22.81 (19.26 to 28.59) | 3.26 | 19.55 | 68 (60) | 27.98 (21.49 to 31.18) | 2.74 | 25.24 | 88 (82) |
| United Republic of Tanzania | 0.446568273 | 385.94 (232.16 to 670.41) | 151.47 | 234.47 | 159 (160) | 234.83 (149.61 to 349.67) | 30.69 | 204.14 | 202 (202) | 127.27 (63.28 to 196.97) | 15.72 | 111.55 | 192 (190) | 70.22 (37.07 to 141.97) | 6.62 | 63.61 | 181 (180) |
| United States Virgin Islands | 0.821830853 | 134.87 (89.42 to 214.81) | 56.83 | 78.04 | 72 (52) | 14.36 (9.03 to 22.55) | 3.15 | 11.21 | 52 (39) | 19 (9.34 to 43.69) | 3.58 | 15.42 | 57 (47) | 23.29 (15.59 to 33.91) | 2.74 | 20.55 | 67 (61) |
| United States of America | 0.862448354 | 101.57 (88.58 to 120.58) | 54.6 | 46.97 | 48 (36) | 21.51 (17.83 to 24.73) | 2.76 | 18.75 | 86 (70) | 21.98 (17.42 to 25.82) | 3.27 | 18.71 | 67 (58) | 21.13 (18.84 to 23.27) | 2.73 | 18.4 | 59 (52) |
| Uruguay | 0.719283445 | 237.18 (188.06 to 292.25) | 97.65 | 139.53 | 107 (94) | 29.14 (22.94 to 37.93) | 6.88 | 22.27 | 98 (87) | 55.2 (42.04 to 71.41) | 8.67 | 46.53 | 120 (118) | 58.79 (48.28 to 74.18) | 2.75 | 56.04 | 172 (163) |
| Uzbekistan | 0.662621694 | 548.53 (400.19 to 704.41) | 139.36 | 409.17 | 188 (183) | 22.62 (14.82 to 43.44) | 9.26 | 13.37 | 65 (74) | 38.76 (24.77 to 62.53) | 9.58 | 29.18 | 89 (89) | 5.52 (3.66 to 10.88) | 4.07 | 1.45 | 6 (1) |
| Vanuatu | 0.473100706 | 326.09 (220.85 to 429.12) | 151.21 | 174.88 | 136 (141) | 51.57 (28.82 to 88.04) | 29.73 | 21.85 | 96 (131) | 14.04 (6.64 to 37.37) | 14.04 | 0 | 2.5 (24) | 43.34 (21.84 to 68.72) | 6.53 | 36.81 | 126 (129) |
| Venezuela (Bolivarian Republic of) | 0.596513059 | 411.18 (291.16 to 547.32) | 152.52 | 258.66 | 167 (166) | 44.59 (31.03 to 62.83) | 15.34 | 29.25 | 116 (116) | 145.4 (103.07 to 197.15) | 10.95 | 134.45 | 199 (196) | 60.46 (40.39 to 82.93) | 6.26 | 54.2 | 166 (166) |
| Viet Nam | 0.627933721 | 151.31 (104.44 to 215.92) | 150.25 | 1.06 | 6 (57) | 19.14 (11.46 to 32.76) | 9.25 | 9.88 | 49 (66) | 23.15 (13.53 to 36.94) | 10.51 | 12.64 | 43 (63) | 5.84 (2.96 to 9.98) | 4.6 | 1.23 | 4 (2) |
| Yemen | 0.450376375 | 1017 (671.07 to 1380.82) | 148.46 | 868.54 | 200 (200) | 165.46 (98.46 to 281.66) | 29.66 | 135.8 | 187 (180) | 106.54 (57.16 to 178.66) | 16.17 | 90.37 | 178 (180) | 83.42 (38.45 to 177.86) | 6.55 | 76.87 | 194 (192) |
| Zambia | 0.505948954 | 315.86 (195.37 to 546.54) | 151.35 | 164.51 | 128 (135) | 185.05 (100.39 to 312.06) | 28.9 | 156.15 | 196 (185) | 105.88 (63.08 to 177.66) | 14.3 | 91.58 | 180 (179) | 62.12 (35.81 to 105.68) | 6.44 | 55.68 | 170 (168) |
| Zimbabwe | 0.473819486 | 233.33 (158.89 to 323.1) | 153.63 | 79.7 | 75 (90) | 45.3 (28.76 to 65.26) | 29.33 | 15.97 | 76 (118) | 49.89 (21.39 to 90.2) | 14.35 | 35.55 | 100 (105) | 54.63 (32.15 to 80.29) | 6.56 | 48.07 | 153 (152) |

## Table S7 Age-standardized DALYs rates of CHD, NTDs, DCAs, and DS with decomposition analysis, categorized by global and SDI regions

| **Location** | **Cause** | **Overll difference** | **Aging** | **Population** | **Epidemiological change** | **Percent change of aging** | **Percent change of population** | **Percent change of epidemiological change** |
| --- | --- | --- | --- | --- | --- | --- | --- | --- |
| Global | Congenital heart anomalies | -24515386.09 | -10666542.59 | 14267206.4 | -28116049.9 | 43.50958435 | -58.19694761 | 114.6873633 |
| Middle SDI | Congenital heart anomalies | -10414019.63 | -4268342.405 | 3682118.02 | -9827795.246 | 40.98650239 | -35.35731783 | 94.37081544 |
| High SDI | Congenital heart anomalies | -1449394.722 | -375001.4445 | 305288.9743 | -1379682.252 | 25.87296882 | -21.0632045 | 95.19023568 |
| High-middle SDI | Congenital heart anomalies | -5707813.961 | -1713076.828 | 862432.0093 | -4857169.142 | 30.01283574 | -15.10967273 | 85.09683699 |
| Low-middle SDI | Congenital heart anomalies | -6792731.607 | -4080351.242 | 5748017.694 | -8460398.059 | 60.06937235 | -84.62012084 | 124.5507485 |
| Low SDI | Congenital heart anomalies | -136558.3499 | -1647806.544 | 7144357.322 | -5633109.128 | 1206.668464 | -5231.724993 | 4125.056528 |
| Global | Neural tube defects | -4910707.632 | -2487445.776 | 3186491.772 | -5609753.628 | 50.65350989 | -64.88864764 | 114.2351378 |
| Middle SDI | Neural tube defects | -1904476.647 | -680782.6179 | 572323.9644 | -1796017.994 | 35.74644083 | -30.05150865 | 94.30506782 |
| High SDI | Neural tube defects | -140952.0585 | -47781.96794 | 37303.16341 | -130473.2539 | 33.89944671 | -26.4651427 | 92.56569599 |
| High-middle SDI | Neural tube defects | -912741.5095 | -247001.5474 | 120869.2902 | -786609.2523 | 27.06150042 | -13.24244476 | 86.18094434 |
| Low-middle SDI | Neural tube defects | -1630031 | -905213.0967 | 1249513.364 | -1974331.267 | 55.53348964 | -76.65580368 | 121.122314 |
| Low SDI | Neural tube defects | -319959.6841 | -650432.6637 | 2755286.426 | -2424813.446 | 203.2858188 | -861.1355003 | 757.8496814 |
| Global | Digestive congenital anomalies | 31358.39881 | -22508.34902 | 77514.56437 | -23647.81654 | -71.777737 | 247.1891657 | -75.41142866 |
| High SDI | Digestive congenital anomalies | -2558.0106 | -2567.873379 | 5467.911598 | -5458.04882 | 100.3855644 | -213.7564089 | 213.3708445 |
| Low SDI | Digestive congenital anomalies | 19719.8574 | -1697.175033 | 26414.62811 | -4997.595677 | -8.6064265 | 133.949387 | -25.34296052 |
| Middle SDI | Digestive congenital anomalies | 5123.885936 | -10686.00384 | 21998.40224 | -6188.512456 | -208.5527269 | 429.330444 | -120.777717 |
| High-middle SDI | Digestive congenital anomalies | -5301.796288 | -4535.987678 | 6042.815976 | -6808.624586 | 85.55567645 | -113.9767665 | 128.4210901 |
| Low-middle SDI | Digestive congenital anomalies | 14389.64928 | -6366.31628 | 25616.22735 | -4860.261788 | -44.2423311 | 178.0184273 | -33.77609623 |
| Global | Down syndrome | -450910.7922 | -635062.3409 | 962223.1234 | -778071.5747 | 140.8399071 | -213.3954521 | 172.555545 |
| Middle SDI | Down syndrome | -415641.293 | -243050.9533 | 213671.2044 | -386261.5442 | 58.4761325 | -51.40759785 | 92.93146535 |
| High SDI | Down syndrome | 57114.21329 | -11356.08377 | 29523.34642 | 38946.95064 | -19.88311336 | 51.69176763 | 68.19134573 |
| High-middle SDI | Down syndrome | -296295.5898 | -111188.4389 | 51657.93981 | -236765.0908 | 37.5261876 | -17.43459625 | 79.90840866 |
| Low-middle SDI | Down syndrome | -68206.09668 | -202448.2647 | 313729.6484 | -179487.4804 | 296.8184291 | -459.9730283 | 263.1545992 |
| Low SDI | Down syndrome | 271642.3569 | -144314.7435 | 642346.1788 | -226389.0784 | -53.12674545 | 236.4676062 | -83.34086076 |

## Table S8 Joinpoint regression analysis results for CHD, NTDs, DCAs, and DS, categorized by global and SDI regions

| **Location** | **Cause** | **Joinpoint** | **Segment** | **Segment**  **Start** | **Segment**  **End** | **APC** | **APC_LCI** | **APC_UCI** | **Significant**  **indicator** | **Test.Statistic** | **P.Value** | **APC_95CI** |
| --- | --- | --- | --- | --- | --- | --- | --- | --- | --- | --- | --- | --- |
| Global | Congenital heart anomalies | 4 | 0 | 1990 | 2000 | -3.728190662 | -3.802320993 | -3.654003206 | Yes | -103.6258959 | 0 | -3.73 (-3.8 to -3.65) |
| Global | Congenital heart anomalies | 4 | 1 | 2000 | 2009 | -2.652919202 | -2.733706089 | -2.572065214 | Yes | -68.03951326 | 0 | -2.65 (-2.73 to -2.57) |
| Global | Congenital heart anomalies | 4 | 2 | 2009 | 2016 | -2.961908369 | -3.064071196 | -2.859637871 | Yes | -59.9673291 | 0 | -2.96 (-3.06 to -2.86) |
| Global | Congenital heart anomalies | 4 | 3 | 2016 | 2019 | -5.226133282 | -5.78008234 | -4.66892738 | Yes | -19.23713697 | 0 | -5.23 (-5.78 to -4.67) |
| Global | Congenital heart anomalies | 4 | 4 | 2019 | 2021 | -7.12347395 | -7.676822084 | -6.566809269 | Yes | -25.98127916 | 0 | -7.12 (-7.68 to -6.57) |
| Global | Digestive congenital anomalies | 3 | 0 | 1990 | 2000 | -2.956930035 | -3.037403263 | -2.87639002 | Yes | -75.24162353 | 0 | -2.96 (-3.04 to -2.88) |
| Global | Digestive congenital anomalies | 3 | 1 | 2000 | 2016 | -1.637227121 | -1.668198209 | -1.606246279 | Yes | -109.0127364 | 0 | -1.64 (-1.67 to -1.61) |
| Global | Digestive congenital anomalies | 3 | 2 | 2016 | 2019 | -4.594032747 | -5.14099704 | -4.043914615 | Yes | -17.01052294 | 0 | -4.59 (-5.14 to -4.04) |
| Global | Digestive congenital anomalies | 3 | 3 | 2019 | 2021 | -6.066788962 | -6.613842617 | -5.516530681 | Yes | -22.28346154 | 0 | -6.07 (-6.61 to -5.52) |
| Global | Down syndrome | 5 | 0 | 1990 | 1996 | -2.693371722 | -2.878725727 | -2.507663974 | Yes | -30.52201087 | 0 | -2.69 (-2.88 to -2.51) |
| Global | Down syndrome | 5 | 1 | 1996 | 2005 | -1.923324424 | -2.02647134 | -1.820068914 | Yes | -39.33860218 | 0 | -1.92 (-2.03 to -1.82) |
| Global | Down syndrome | 5 | 2 | 2005 | 2012 | -0.877494753 | -1.023741218 | -0.731032196 | Yes | -12.72325875 | 0 | -0.88 (-1.02 to -0.73) |
| Global | Down syndrome | 5 | 3 | 2012 | 2016 | 0.264735325 | -0.127121522 | 0.658129644 | No | 1.43907506 | 0.171 | 0.26 (-0.13 to 0.66) |
| Global | Down syndrome | 5 | 4 | 2016 | 2019 | -2.510965333 | -3.159525415 | -1.858061715 | Yes | -8.120506486 | 0 | -2.51 (-3.16 to -1.86) |
| Global | Down syndrome | 5 | 5 | 2019 | 2021 | -4.97988993 | -5.546514911 | -4.409865776 | Yes | -18.20377705 | 0 | -4.98 (-5.55 to -4.41) |
| Global | Neural tube defects | 4 | 0 | 1990 | 1995 | -2.893716083 | -3.038634279 | -2.748581292 | Yes | -41.30729295 | 0 | -2.89 (-3.04 to -2.75) |
| Global | Neural tube defects | 4 | 1 | 1995 | 2004 | -3.361536557 | -3.424023305 | -3.299009379 | Yes | -111.0639873 | 0 | -3.36 (-3.42 to -3.3) |
| Global | Neural tube defects | 4 | 2 | 2004 | 2016 | -2.699053537 | -2.735355474 | -2.662738052 | Yes | -154.0478372 | 0 | -2.7 (-2.74 to -2.66) |
| Global | Neural tube defects | 4 | 3 | 2016 | 2019 | -4.868184168 | -5.317762317 | -4.416471295 | Yes | -22.13407287 | 0 | -4.87 (-5.32 to -4.42) |
| Global | Neural tube defects | 4 | 4 | 2019 | 2021 | -5.652225563 | -6.087778623 | -5.214652463 | Yes | -26.4173069 | 0 | -5.65 (-6.09 to -5.21) |
| High SDI | Congenital heart anomalies | 5 | 0 | 1990 | 1992 | -4.652105267 | -6.033197815 | -3.250713883 | Yes | -6.959079839 | 0 | -4.65 (-6.03 to -3.25) |
| High SDI | Congenital heart anomalies | 5 | 1 | 1992 | 1998 | -6.244086937 | -6.471099447 | -6.016523425 | Yes | -56.68811378 | 0 | -6.24 (-6.47 to -6.02) |
| High SDI | Congenital heart anomalies | 5 | 2 | 1998 | 2004 | -4.551394251 | -4.765710321 | -4.336595883 | Yes | -44.16947908 | 0 | -4.55 (-4.77 to -4.34) |
| High SDI | Congenital heart anomalies | 5 | 3 | 2004 | 2013 | -3.444328917 | -3.590806604 | -3.297628681 | Yes | -49.20907903 | 0 | -3.44 (-3.59 to -3.3) |
| High SDI | Congenital heart anomalies | 5 | 4 | 2013 | 2017 | -2.668488769 | -3.488029294 | -1.841989037 | Yes | -6.81786896 | 0 | -2.67 (-3.49 to -1.84) |
| High SDI | Congenital heart anomalies | 5 | 5 | 2017 | 2021 | -4.705065626 | -5.273827455 | -4.132888796 | Yes | -17.1594718 | 0 | -4.71 (-5.27 to -4.13) |
| High SDI | Digestive congenital anomalies | 5 | 0 | 1990 | 1997 | -5.711728225 | -5.873056787 | -5.550123154 | Yes | -73.20247544 | 0 | -5.71 (-5.87 to -5.55) |
| High SDI | Digestive congenital anomalies | 5 | 1 | 1997 | 2002 | -2.882067746 | -3.242978087 | -2.519811184 | Yes | -16.74194603 | 0 | -2.88 (-3.24 to -2.52) |
| High SDI | Digestive congenital anomalies | 5 | 2 | 2002 | 2007 | -1.157482877 | -1.563146506 | -0.750147487 | Yes | -6.033930112 | 0 | -1.16 (-1.56 to -0.75) |
| High SDI | Digestive congenital anomalies | 5 | 3 | 2007 | 2011 | -3.690673129 | -4.368869015 | -3.00766762 | Yes | -11.34228221 | 0 | -3.69 (-4.37 to -3.01) |
| High SDI | Digestive congenital anomalies | 5 | 4 | 2011 | 2018 | -2.677848387 | -2.92113299 | -2.4339541 | Yes | -23.11508784 | 0 | -2.68 (-2.92 to -2.43) |
| High SDI | Digestive congenital anomalies | 5 | 5 | 2018 | 2021 | -5.635711862 | -6.383507072 | -4.881943372 | Yes | -15.54023567 | 0 | -5.64 (-6.38 to -4.88) |
| High SDI | Down syndrome | 5 | 0 | 1990 | 1997 | -1.545541713 | -1.743464997 | -1.347219743 | Yes | -16.49815219 | 0 | -1.55 (-1.74 to -1.35) |
| High SDI | Down syndrome | 5 | 1 | 1997 | 2002 | 2.258842105 | 1.902933618 | 2.615993646 | Yes | 13.65548476 | 0 | 2.26 (1.9 to 2.62) |
| High SDI | Down syndrome | 5 | 2 | 2002 | 2012 | -0.203130982 | -0.292909602 | -0.113271523 | Yes | -4.815498598 | 0 | -0.2 (-0.29 to -0.11) |
| High SDI | Down syndrome | 5 | 3 | 2012 | 2016 | 3.694542408 | 3.236080554 | 4.155040249 | Yes | 17.45118002 | 0 | 3.69 (3.24 to 4.16) |
| High SDI | Down syndrome | 5 | 4 | 2016 | 2019 | 0.62810042 | -0.166715268 | 1.429243978 | No | 1.682967846 | 0.113 | 0.63 (-0.17 to 1.43) |
| High SDI | Down syndrome | 5 | 5 | 2019 | 2021 | -3.010504186 | -3.888880425 | -2.124100314 | Yes | -7.161518549 | 0 | -3.01 (-3.89 to -2.12) |
| High SDI | Neural tube defects | 5 | 0 | 1990 | 1997 | -6.764215625 | -6.923620586 | -6.604537663 | Yes | -87.2412136 | 0 | -6.76 (-6.92 to -6.6) |
| High SDI | Neural tube defects | 5 | 1 | 1997 | 2001 | -4.176195683 | -4.761631931 | -3.587160722 | Yes | -14.83713371 | 0 | -4.18 (-4.76 to -3.59) |
| High SDI | Neural tube defects | 5 | 2 | 2001 | 2004 | -2.464539925 | -3.74130374 | -1.170841268 | Yes | -4.036567417 | 0.001 | -2.46 (-3.74 to -1.17) |
| High SDI | Neural tube defects | 5 | 3 | 2004 | 2007 | -0.944120273 | -2.389283824 | 0.522439471 | No | -1.375740145 | 0.189 | -0.94 (-2.39 to 0.52) |
| High SDI | Neural tube defects | 5 | 4 | 2007 | 2019 | -1.948109125 | -2.063404846 | -1.832677673 | Yes | -35.64024421 | 0 | -1.95 (-2.06 to -1.83) |
| High SDI | Neural tube defects | 5 | 5 | 2019 | 2021 | -4.99232361 | -7.105300713 | -2.831284861 | Yes | -4.853333742 | 0 | -4.99 (-7.11 to -2.83) |
| High-middle SDI | Congenital heart anomalies | 3 | 0 | 1990 | 2009 | -5.506169056 | -5.564032725 | -5.448269931 | Yes | -192.2812559 | 0 | -5.51 (-5.56 to -5.45) |
| High-middle SDI | Congenital heart anomalies | 3 | 1 | 2009 | 2012 | -6.546946503 | -7.788986167 | -5.28817714 | Yes | -10.52440867 | 0 | -6.55 (-7.79 to -5.29) |
| High-middle SDI | Congenital heart anomalies | 3 | 2 | 2012 | 2017 | -4.322060414 | -4.740575064 | -3.901707053 | Yes | -20.95954592 | 0 | -4.32 (-4.74 to -3.9) |
| High-middle SDI | Congenital heart anomalies | 3 | 3 | 2017 | 2021 | -7.68233284 | -8.205616372 | -7.156066276 | Yes | -29.24366333 | 0 | -7.68 (-8.21 to -7.16) |
| High-middle SDI | Digestive congenital anomalies | 4 | 0 | 1990 | 2002 | -5.13617312 | -5.212409404 | -5.059875519 | Yes | -137.7886083 | 0 | -5.14 (-5.21 to -5.06) |
| High-middle SDI | Digestive congenital anomalies | 4 | 1 | 2002 | 2008 | -4.261265725 | -4.425571045 | -4.096677941 | Yes | -53.2639685 | 0 | -4.26 (-4.43 to -4.1) |
| High-middle SDI | Digestive congenital anomalies | 4 | 2 | 2008 | 2011 | -5.364077061 | -5.953441796 | -4.771018935 | Yes | -18.54120503 | 0 | -5.36 (-5.95 to -4.77) |
| High-middle SDI | Digestive congenital anomalies | 4 | 3 | 2011 | 2016 | -3.251141302 | -3.448291632 | -3.053588408 | Yes | -34.0414902 | 0 | -3.25 (-3.45 to -3.05) |
| High-middle SDI | Digestive congenital anomalies | 4 | 4 | 2016 | 2021 | -8.435726804 | -8.632408868 | -8.238621353 | Yes | -86.1039992 | 0 | -8.44 (-8.63 to -8.24) |
| High-middle SDI | Down syndrome | 4 | 0 | 1990 | 2001 | -3.413156794 | -3.755436164 | -3.069660158 | Yes | -20.5518992 | 0 | -3.41 (-3.76 to -3.07) |
| High-middle SDI | Down syndrome | 4 | 1 | 2001 | 2005 | -5.692102062 | -7.091363889 | -4.271766482 | Yes | -8.236706034 | 0 | -5.69 (-7.09 to -4.27) |
| High-middle SDI | Down syndrome | 4 | 2 | 2005 | 2012 | -3.53843175 | -3.962540648 | -3.112449953 | Yes | -17.17673334 | 0 | -3.54 (-3.96 to -3.11) |
| High-middle SDI | Down syndrome | 4 | 3 | 2012 | 2017 | -1.402158806 | -2.214885333 | -0.582677423 | Yes | -3.584236947 | 0.002 | -1.4 (-2.21 to -0.58) |
| High-middle SDI | Down syndrome | 4 | 4 | 2017 | 2021 | -5.007353369 | -5.965833337 | -4.039103719 | Yes | -10.64221641 | 0 | -5.01 (-5.97 to -4.04) |
| High-middle SDI | Neural tube defects | 5 | 0 | 1990 | 1997 | -5.609363714 | -5.898289673 | -5.319550649 | Yes | -40.13661288 | 0 | -5.61 (-5.9 to -5.32) |
| High-middle SDI | Neural tube defects | 5 | 1 | 1997 | 2001 | -7.26648988 | -8.0612949 | -6.464813814 | Yes | -18.68042182 | 0 | -7.27 (-8.06 to -6.46) |
| High-middle SDI | Neural tube defects | 5 | 2 | 2001 | 2006 | -8.582784711 | -9.043778526 | -8.119454439 | Yes | -37.83372801 | 0 | -8.58 (-9.04 to -8.12) |
| High-middle SDI | Neural tube defects | 5 | 3 | 2006 | 2011 | -6.915949215 | -7.351571141 | -6.478279047 | Yes | -32.56443974 | 0 | -6.92 (-7.35 to -6.48) |
| High-middle SDI | Neural tube defects | 5 | 4 | 2011 | 2017 | -4.667774209 | -4.986661714 | -4.347816442 | Yes | -30.4087479 | 0 | -4.67 (-4.99 to -4.35) |
| High-middle SDI | Neural tube defects | 5 | 5 | 2017 | 2021 | -9.022777059 | -9.574534959 | -8.467652444 | Yes | -33.13225335 | 0 | -9.02 (-9.57 to -8.47) |
| Middle SDI | Congenital heart anomalies | 5 | 0 | 1990 | 1999 | -5.325068212 | -5.58831246 | -5.061089971 | Yes | -41.88901793 | 0 | -5.33 (-5.59 to -5.06) |
| Middle SDI | Congenital heart anomalies | 5 | 1 | 1999 | 2005 | -3.971561113 | -4.402813751 | -3.538363033 | Yes | -19.19100617 | 0 | -3.97 (-4.4 to -3.54) |
| Middle SDI | Congenital heart anomalies | 5 | 2 | 2005 | 2008 | -2.863914352 | -4.476397373 | -1.224211865 | Yes | -3.699858127 | 0.002 | -2.86 (-4.48 to -1.22) |
| Middle SDI | Congenital heart anomalies | 5 | 3 | 2008 | 2015 | -3.625613189 | -3.865141808 | -3.385487762 | Yes | -31.63115069 | 0 | -3.63 (-3.87 to -3.39) |
| Middle SDI | Congenital heart anomalies | 5 | 4 | 2015 | 2018 | -5.425398189 | -6.85114488 | -3.977828862 | Yes | -7.827100943 | 0 | -5.43 (-6.85 to -3.98) |
| Middle SDI | Congenital heart anomalies | 5 | 5 | 2018 | 2021 | -8.266634704 | -9.070657227 | -7.455502791 | Yes | -20.89076315 | 0 | -8.27 (-9.07 to -7.46) |
| Middle SDI | Digestive congenital anomalies | 4 | 0 | 1990 | 1998 | -4.256331785 | -4.586757608 | -3.924761663 | Yes | -26.432713 | 0 | -4.26 (-4.59 to -3.92) |
| Middle SDI | Digestive congenital anomalies | 4 | 1 | 1998 | 2003 | -3.257305899 | -3.952683321 | -2.556893981 | Yes | -9.644320021 | 0 | -3.26 (-3.95 to -2.56) |
| Middle SDI | Digestive congenital anomalies | 4 | 2 | 2003 | 2015 | -2.163125699 | -2.275509837 | -2.050612319 | Yes | -39.97419744 | 0 | -2.16 (-2.28 to -2.05) |
| Middle SDI | Digestive congenital anomalies | 4 | 3 | 2015 | 2018 | -5.041641445 | -6.554580763 | -3.5042067 | Yes | -6.766991547 | 0 | -5.04 (-6.55 to -3.5) |
| Middle SDI | Digestive congenital anomalies | 4 | 4 | 2018 | 2021 | -8.29068524 | -9.13545843 | -7.438058142 | Yes | -19.64823208 | 0 | -8.29 (-9.14 to -7.44) |
| Middle SDI | Down syndrome | 5 | 0 | 1990 | 1997 | -4.112572411 | -4.688282817 | -3.533384547 | Yes | -14.86368268 | 0 | -4.11 (-4.69 to -3.53) |
| Middle SDI | Down syndrome | 5 | 1 | 1997 | 2000 | -2.170768017 | -5.157075403 | 0.909568859 | No | -1.508916149 | 0.152 | -2.17 (-5.16 to 0.91) |
| Middle SDI | Down syndrome | 5 | 2 | 2000 | 2005 | -4.376852629 | -5.16318204 | -3.584003451 | Yes | -11.55276491 | 0 | -4.38 (-5.16 to -3.58) |
| Middle SDI | Down syndrome | 5 | 3 | 2005 | 2011 | -2.006648139 | -2.535356244 | -1.475071997 | Yes | -7.986324628 | 0 | -2.01 (-2.54 to -1.48) |
| Middle SDI | Down syndrome | 5 | 4 | 2011 | 2018 | -1.400438127 | -1.720387679 | -1.079446978 | Yes | -9.248808451 | 0 | -1.4 (-1.72 to -1.08) |
| Middle SDI | Down syndrome | 5 | 5 | 2018 | 2021 | -6.673607061 | -7.495060216 | -5.844859319 | Yes | -16.65138819 | 0 | -6.67 (-7.5 to -5.84) |
| Middle SDI | Neural tube defects | 4 | 0 | 1990 | 1995 | -4.796511913 | -5.203874138 | -4.387399153 | Yes | -24.08278001 | 0 | -4.8 (-5.2 to -4.39) |
| Middle SDI | Neural tube defects | 4 | 1 | 1995 | 2004 | -6.134940183 | -6.284902201 | -5.984738198 | Yes | -83.19003182 | 0 | -6.13 (-6.28 to -5.98) |
| Middle SDI | Neural tube defects | 4 | 2 | 2004 | 2015 | -4.506222484 | -4.619897898 | -4.39241159 | Yes | -81.32919423 | 0 | -4.51 (-4.62 to -4.39) |
| Middle SDI | Neural tube defects | 4 | 3 | 2015 | 2018 | -6.107850617 | -7.550450554 | -4.642740082 | Yes | -8.551401554 | 0 | -6.11 (-7.55 to -4.64) |
| Middle SDI | Neural tube defects | 4 | 4 | 2018 | 2021 | -8.410313355 | -9.156628064 | -7.657867371 | Yes | -22.55840017 | 0 | -8.41 (-9.16 to -7.66) |
| Low-middle SDI | Congenital heart anomalies | 4 | 0 | 1990 | 2001 | -3.283091827 | -3.355337916 | -3.21079173 | Yes | -93.85283711 | 0 | -3.28 (-3.36 to -3.21) |
| Low-middle SDI | Congenital heart anomalies | 4 | 1 | 2001 | 2010 | -2.480808427 | -2.565826745 | -2.395715925 | Yes | -60.51108002 | 0 | -2.48 (-2.57 to -2.4) |
| Low-middle SDI | Congenital heart anomalies | 4 | 2 | 2010 | 2016 | -3.714792447 | -3.852884083 | -3.576502475 | Yes | -55.41398086 | 0 | -3.71 (-3.85 to -3.58) |
| Low-middle SDI | Congenital heart anomalies | 4 | 3 | 2016 | 2019 | -5.652671974 | -6.239950639 | -5.061714811 | Yes | -19.57797869 | 0 | -5.65 (-6.24 to -5.06) |
| Low-middle SDI | Congenital heart anomalies | 4 | 4 | 2019 | 2021 | -8.803589257 | -9.426792933 | -8.176097526 | Yes | -28.23493294 | 0 | -8.8 (-9.43 to -8.18) |
| Low-middle SDI | Digestive congenital anomalies | 4 | 0 | 1990 | 2001 | -2.49732294 | -2.558477194 | -2.436130305 | Yes | -84.68732887 | 0 | -2.5 (-2.56 to -2.44) |
| Low-middle SDI | Digestive congenital anomalies | 4 | 1 | 2001 | 2011 | -1.456334914 | -1.518903868 | -1.393726208 | Yes | -48.52715809 | 0 | -1.46 (-1.52 to -1.39) |
| Low-middle SDI | Digestive congenital anomalies | 4 | 2 | 2011 | 2016 | -2.500396891 | -2.675975304 | -2.324501724 | Yes | -29.51523568 | 0 | -2.5 (-2.68 to -2.32) |
| Low-middle SDI | Digestive congenital anomalies | 4 | 3 | 2016 | 2019 | -4.504389191 | -4.971020957 | -4.035466068 | Yes | -19.76794712 | 0 | -4.5 (-4.97 to -4.04) |
| Low-middle SDI | Digestive congenital anomalies | 4 | 4 | 2019 | 2021 | -7.084404964 | -7.550029251 | -6.616435561 | Yes | -30.72796795 | 0 | -7.08 (-7.55 to -6.62) |
| Low-middle SDI | Down syndrome | 2 | 0 | 1990 | 2002 | -2.080064458 | -2.250704533 | -1.909126497 | Yes | -24.87327577 | 0 | -2.08 (-2.25 to -1.91) |
| Low-middle SDI | Down syndrome | 2 | 1 | 2002 | 2017 | -0.960699625 | -1.052853353 | -0.86846007 | Yes | -21.40242718 | 0 | -0.96 (-1.05 to -0.87) |
| Low-middle SDI | Down syndrome | 2 | 2 | 2017 | 2021 | -4.987538903 | -5.365739975 | -4.607826369 | Yes | -26.4745756 | 0 | -4.99 (-5.37 to -4.61) |
| Low-middle SDI | Neural tube defects | 5 | 0 | 1990 | 1995 | -3.047520557 | -3.182516861 | -2.912336023 | Yes | -47.34339733 | 0 | -3.05 (-3.18 to -2.91) |
| Low-middle SDI | Neural tube defects | 5 | 1 | 1995 | 2002 | -3.557551316 | -3.651126189 | -3.463885564 | Yes | -79.53651941 | 0 | -3.56 (-3.65 to -3.46) |
| Low-middle SDI | Neural tube defects | 5 | 2 | 2002 | 2010 | -3.165088316 | -3.230900199 | -3.099231674 | Yes | -100.8338175 | 0 | -3.17 (-3.23 to -3.1) |
| Low-middle SDI | Neural tube defects | 5 | 3 | 2010 | 2016 | -4.111797931 | -4.220949357 | -4.002522114 | Yes | -78.5743492 | 0 | -4.11 (-4.22 to -4) |
| Low-middle SDI | Neural tube defects | 5 | 4 | 2016 | 2019 | -5.778653941 | -6.277644652 | -5.277006535 | Yes | -23.89281271 | 0 | -5.78 (-6.28 to -5.28) |
| Low-middle SDI | Neural tube defects | 5 | 5 | 2019 | 2021 | -7.181625702 | -7.705090825 | -6.655191664 | Yes | -28.08659067 | 0 | -7.18 (-7.71 to -6.66) |
| Low SDI | Congenital heart anomalies | 5 | 0 | 1990 | 1995 | -1.368009417 | -1.615761867 | -1.119633074 | Yes | -11.67362212 | 0 | -1.37 (-1.62 to -1.12) |
| Low SDI | Congenital heart anomalies | 5 | 1 | 1995 | 2001 | -2.244882658 | -2.477934048 | -2.011274338 | Yes | -20.27492354 | 0 | -2.24 (-2.48 to -2.01) |
| Low SDI | Congenital heart anomalies | 5 | 2 | 2001 | 2007 | -1.82038855 | -2.034288123 | -1.606021947 | Yes | -17.95396041 | 0 | -1.82 (-2.03 to -1.61) |
| Low SDI | Congenital heart anomalies | 5 | 3 | 2007 | 2010 | -2.913790208 | -3.77634653 | -2.043501864 | Yes | -7.062726887 | 0 | -2.91 (-3.78 to -2.04) |
| Low SDI | Congenital heart anomalies | 5 | 4 | 2010 | 2016 | -2.246117703 | -2.424948254 | -2.066959401 | Yes | -26.44392832 | 0 | -2.25 (-2.42 to -2.07) |
| Low SDI | Congenital heart anomalies | 5 | 5 | 2016 | 2021 | -5.238476646 | -5.387418706 | -5.089300117 | Yes | -72.90961647 | 0 | -5.24 (-5.39 to -5.09) |
| Low SDI | Digestive congenital anomalies | 4 | 0 | 1990 | 1995 | -1.554016934 | -1.722310001 | -1.385435678 | Yes | -19.23191556 | 0 | -1.55 (-1.72 to -1.39) |
| Low SDI | Digestive congenital anomalies | 4 | 1 | 1995 | 2001 | -2.072957735 | -2.23097001 | -1.914690083 | Yes | -27.25224706 | 0 | -2.07 (-2.23 to -1.91) |
| Low SDI | Digestive congenital anomalies | 4 | 2 | 2001 | 2011 | -1.589833476 | -1.644312694 | -1.535324082 | Yes | -60.80311139 | 0 | -1.59 (-1.64 to -1.54) |
| Low SDI | Digestive congenital anomalies | 4 | 3 | 2011 | 2016 | -1.131016477 | -1.28736539 | -0.974419925 | Yes | -15.09969158 | 0 | -1.13 (-1.29 to -0.97) |
| Low SDI | Digestive congenital anomalies | 4 | 4 | 2016 | 2021 | -4.269243857 | -4.359611059 | -4.178791271 | Yes | -97.05910958 | 0 | -4.27 (-4.36 to -4.18) |
| Low SDI | Down syndrome | 5 | 0 | 1990 | 2002 | -1.968741568 | -2.039199708 | -1.89823275 | Yes | -58.94562917 | 0 | -1.97 (-2.04 to -1.9) |
| Low SDI | Down syndrome | 5 | 1 | 2002 | 2007 | -0.201921801 | -0.536130962 | 0.133410338 | No | -1.284318451 | 0.219 | -0.2 (-0.54 to 0.13) |
| Low SDI | Down syndrome | 5 | 2 | 2007 | 2011 | -0.775855006 | -1.259417478 | -0.289924383 | Yes | -3.398209981 | 0.004 | -0.78 (-1.26 to -0.29) |
| Low SDI | Down syndrome | 5 | 3 | 2011 | 2016 | 0.088021531 | -0.190344515 | 0.367163932 | No | 0.673339543 | 0.511 | 0.09 (-0.19 to 0.37) |
| Low SDI | Down syndrome | 5 | 4 | 2016 | 2019 | -3.007039062 | -3.743651987 | -2.26478912 | Yes | -8.536378531 | 0 | -3.01 (-3.74 to -2.26) |
| Low SDI | Down syndrome | 5 | 5 | 2019 | 2021 | -4.341212005 | -4.983877112 | -3.694200074 | Yes | -14.03347295 | 0 | -4.34 (-4.98 to -3.69) |
| Low SDI | Neural tube defects | 4 | 0 | 1990 | 1995 | -1.74708329 | -1.902473218 | -1.591447219 | Yes | -23.39505845 | 0 | -1.75 (-1.9 to -1.59) |
| Low SDI | Neural tube defects | 4 | 1 | 1995 | 1999 | -2.339720638 | -2.666300906 | -2.012044606 | Yes | -14.84928955 | 0 | -2.34 (-2.67 to -2.01) |
| Low SDI | Neural tube defects | 4 | 2 | 1999 | 2011 | -2.759547155 | -2.798120098 | -2.720958905 | Yes | -148.1795069 | 0 | -2.76 (-2.8 to -2.72) |
| Low SDI | Neural tube defects | 4 | 3 | 2011 | 2016 | -2.369666059 | -2.530872152 | -2.208193343 | Yes | -30.48869343 | 0 | -2.37 (-2.53 to -2.21) |
| Low SDI | Neural tube defects | 4 | 4 | 2016 | 2021 | -5.138304403 | -5.233410142 | -5.043103219 | Yes | -110.4842413 | 0 | -5.14 (-5.23 to -5.04) |

## Table S9 Forecast of DALYs all-age cases and ASDR for CHD, NTDs, DCAs, and DS based on disease burden from 1990 to 2021, grouped by sex, projected to 2040

| **Cause** | **Sex** | **Year** | **Pred all ages number (95% CI)** | **Pred ASDR (95% CI)** |
| --- | --- | --- | --- | --- |
| Congenital heart anomalies | Both | 1990 | 46825527.27(46773654.52 to 46877400.01) | 763.42(762.51 to 764.32) |
| Congenital heart anomalies | Both | 1991 | 46000711.08(45949034.85 to 46052387.32) | 747.35(746.46 to 748.25) |
| Congenital heart anomalies | Both | 1992 | 45043281.83(44991793.31 to 45094770.36) | 730.86(729.98 to 731.74) |
| Congenital heart anomalies | Both | 1993 | 43999058.72(43947826.66 to 44050290.78) | 714.33(713.46 to 715.2) |
| Congenital heart anomalies | Both | 1994 | 42893962.46(42843094.24 to 42944830.67) | 698.02(697.15 to 698.88) |
| Congenital heart anomalies | Both | 1995 | 41846593.03(41796038.12 to 41897147.93) | 683.16(682.3 to 684.01) |
| Congenital heart anomalies | Both | 1996 | 40654255.06(40604143.4 to 40704366.72) | 665.19(664.36 to 666.03) |
| Congenital heart anomalies | Both | 1997 | 39624811.25(39575081.94 to 39674540.56) | 649.16(648.33 to 649.99) |
| Congenital heart anomalies | Both | 1998 | 38570688.26(38521309.88 to 38620066.64) | 632.27(631.45 to 633.09) |
| Congenital heart anomalies | Both | 1999 | 37703388.59(37654226.52 to 37752550.66) | 618.03(617.22 to 618.84) |
| Congenital heart anomalies | Both | 2000 | 37022408.27(36973429.6 to 37071386.94) | 606.3(605.5 to 607.1) |
| Congenital heart anomalies | Both | 2001 | 36350688.18(36301944.52 to 36399431.84) | 594.14(593.35 to 594.93) |
| Congenital heart anomalies | Both | 2002 | 35789433.26(35740937.78 to 35837928.74) | 583.07(582.29 to 583.85) |
| Congenital heart anomalies | Both | 2003 | 35131091.49(35082836.76 to 35179346.21) | 569.54(568.77 to 570.31) |
| Congenital heart anomalies | Both | 2004 | 34653576.34(34605450.36 to 34701702.32) | 558.32(557.56 to 559.09) |
| Congenital heart anomalies | Both | 2005 | 34171623.3(34123666.99 to 34219579.62) | 546.6(545.85 to 547.35) |
| Congenital heart anomalies | Both | 2006 | 33778819.91(33731118.08 to 33826521.74) | 535.72(534.98 to 536.46) |
| Congenital heart anomalies | Both | 2007 | 33399130.55(33351621.32 to 33446639.79) | 524.43(523.7 to 525.16) |
| Congenital heart anomalies | Both | 2008 | 33055006.84(33007591.12 to 33102422.56) | 513.32(512.6 to 514.04) |
| Congenital heart anomalies | Both | 2009 | 32572125.98(32524839.4 to 32619412.57) | 500.14(499.43 to 500.85) |
| Congenital heart anomalies | Both | 2010 | 31884948.61(31837950.45 to 31931946.77) | 484.97(484.28 to 485.67) |
| Congenital heart anomalies | Both | 2011 | 31234348.4(31187691.29 to 31281005.51) | 471.84(471.15 to 472.52) |
| Congenital heart anomalies | Both | 2012 | 30803060.24(30756642.05 to 30849478.43) | 462.5(461.83 to 463.18) |
| Congenital heart anomalies | Both | 2013 | 30410357.43(30364112.8 to 30456602.06) | 453.85(453.18 to 454.51) |
| Congenital heart anomalies | Both | 2014 | 29857883.55(29811875.64 to 29903891.46) | 443.05(442.4 to 443.71) |
| Congenital heart anomalies | Both | 2015 | 29312795.08(29267019.22 to 29358570.93) | 432.03(431.39 to 432.68) |
| Congenital heart anomalies | Both | 2016 | 28567139.15(28521622.13 to 28612656.17) | 417.76(417.12 to 418.4) |
| Congenital heart anomalies | Both | 2017 | 27593751.51(27548629.48 to 27638873.54) | 401.38(400.75 to 402) |
| Congenital heart anomalies | Both | 2018 | 26437802.14(26393233.93 to 26482370.35) | 384.25(383.64 to 384.87) |
| Congenital heart anomalies | Both | 2019 | 25343259.93(25299181.5 to 25387338.36) | 369.69(369.09 to 370.3) |
| Congenital heart anomalies | Both | 2020 | 23572290.31(23529106.14 to 23615474.49) | 346.73(346.14 to 347.32) |
| Congenital heart anomalies | Both | 2021 | 22310165.28(22267644.29 to 22352686.28) | 332.38(331.8 to 332.96) |
| Congenital heart anomalies | Both | 2022 | 22133948.82(20659207.86 to 23608689.78) | 324.4(302.71 to 346.08) |
| Congenital heart anomalies | Both | 2023 | 21265399.15(19635489.05 to 22895309.25) | 311.69(287.69 to 335.69) |
| Congenital heart anomalies | Both | 2024 | 20418741.95(18577558.46 to 22259925.43) | 299.49(272.31 to 326.66) |
| Congenital heart anomalies | Both | 2025 | 19593043.11(17500291.03 to 21685795.2) | 287.78(256.81 to 318.74) |
| Congenital heart anomalies | Both | 2026 | 18792518.49(16421897.84 to 21163139.14) | 276.55(241.37 to 311.73) |
| Congenital heart anomalies | Both | 2027 | 18019505.15(15355775.03 to 20683235.27) | 265.8(226.15 to 305.45) |
| Congenital heart anomalies | Both | 2028 | 17276139.64(14312371.05 to 20239908.24) | 255.51(211.24 to 299.77) |
| Congenital heart anomalies | Both | 2029 | 16563984.73(13298859.38 to 19829110.08) | 245.66(196.73 to 294.59) |
| Congenital heart anomalies | Both | 2030 | 15884429.32(12320178.51 to 19448680.13) | 236.24(182.64 to 289.83) |
| Congenital heart anomalies | Both | 2031 | 15237759.65(11378874.9 to 19096644.39) | 227.23(169.02 to 285.44) |
| Congenital heart anomalies | Both | 2032 | 14622522.89(10475208.62 to 18769837.16) | 218.63(155.87 to 281.39) |
| Congenital heart anomalies | Both | 2033 | 14036988.63(9608829.63 to 18465147.63) | 210.42(143.21 to 277.64) |
| Congenital heart anomalies | Both | 2034 | 13479903.91(8779116.24 to 18180691.58) | 202.59(131.03 to 274.16) |
| Congenital heart anomalies | Both | 2035 | 12950884.83(7985614.17 to 17916155.49) | 195.13(119.32 to 270.94) |
| Congenital heart anomalies | Both | 2036 | 12449014.32(7227172.47 to 17670856.17) | 188.01(108.06 to 267.96) |
| Congenital heart anomalies | Both | 2037 | 11973322.1(6502384.13 to 17444260.06) | 181.23(97.25 to 265.22) |
| Congenital heart anomalies | Both | 2038 | 11521687.19(5809146.34 to 17234228.04) | 174.78(86.86 to 262.7) |
| Congenital heart anomalies | Both | 2039 | 11091932.72(5145310.92 to 17038554.51) | 168.64(76.87 to 260.41) |
| Congenital heart anomalies | Both | 2040 | 10682983.45(4509261.76 to 16856705.13) | 162.8(67.27 to 258.34) |
| Congenital heart anomalies | Female | 1990 | 19707927.44(19683273.25 to 19732581.64) | 662.8(662.51 to 663.09) |
| Congenital heart anomalies | Female | 1991 | 19385153.77(19360583.43 to 19409724.12) | 650.07(649.78 to 650.35) |
| Congenital heart anomalies | Female | 1992 | 19017355.44(18992845.58 to 19041865.31) | 637.22(636.93 to 637.51) |
| Congenital heart anomalies | Female | 1993 | 18606726.58(18582313.3 to 18631139.85) | 624.04(623.76 to 624.33) |
| Congenital heart anomalies | Female | 1994 | 18149105.29(18124856.81 to 18173353.76) | 610.26(609.98 to 610.54) |
| Congenital heart anomalies | Female | 1995 | 17721322.06(17697214.87 to 17745429.25) | 597.86(597.58 to 598.14) |
| Congenital heart anomalies | Female | 1996 | 17239512.54(17215609.2 to 17263415.88) | 582.98(582.71 to 583.26) |
| Congenital heart anomalies | Female | 1997 | 16819839.96(16796111.59 to 16843568.34) | 569.53(569.26 to 569.8) |
| Congenital heart anomalies | Female | 1998 | 16380526.26(16356971.11 to 16404081.41) | 554.98(554.71 to 555.25) |
| Congenital heart anomalies | Female | 1999 | 16062855.59(16039360.17 to 16086351.01) | 544.14(543.87 to 544.41) |
| Congenital heart anomalies | Female | 2000 | 15811403.81(15787984.22 to 15834823.4) | 535.09(534.82 to 535.35) |
| Congenital heart anomalies | Female | 2001 | 15540196.83(15516896.62 to 15563497.05) | 524.87(524.61 to 525.13) |
| Congenital heart anomalies | Female | 2002 | 15329599.96(15306386.79 to 15352813.14) | 516.02(515.76 to 516.27) |
| Congenital heart anomalies | Female | 2003 | 15067678.01(15044568.98 to 15090787.04) | 504.67(504.41 to 504.92) |
| Congenital heart anomalies | Female | 2004 | 14861505.07(14838482.31 to 14884527.83) | 494.67(494.42 to 494.92) |
| Congenital heart anomalies | Female | 2005 | 14663377.54(14640445.35 to 14686309.73) | 484.53(484.28 to 484.78) |
| Congenital heart anomalies | Female | 2006 | 14535699.36(14512880.95 to 14558517.76) | 476.2(475.96 to 476.45) |
| Congenital heart anomalies | Female | 2007 | 14398689.65(14375956.85 to 14421422.44) | 466.97(466.73 to 467.22) |
| Congenital heart anomalies | Female | 2008 | 14291516.26(14268806.89 to 14314225.63) | 458.34(458.11 to 458.58) |
| Congenital heart anomalies | Female | 2009 | 14124448.39(14101784.46 to 14147112.31) | 447.82(447.59 to 448.06) |
| Congenital heart anomalies | Female | 2010 | 13867961.77(13845428.41 to 13890495.12) | 435.44(435.22 to 435.67) |
| Congenital heart anomalies | Female | 2011 | 13628128(13605740.76 to 13650515.25) | 424.85(424.63 to 425.08) |
| Congenital heart anomalies | Female | 2012 | 13455422.24(13433164.06 to 13477680.42) | 416.82(416.6 to 417.04) |
| Congenital heart anomalies | Female | 2013 | 13325401.87(13303192.17 to 13347611.58) | 410.24(410.02 to 410.46) |
| Congenital heart anomalies | Female | 2014 | 13133929.52(13111807.05 to 13156051.99) | 402.01(401.79 to 402.23) |
| Congenital heart anomalies | Female | 2015 | 12921600.56(12899576.48 to 12943624.65) | 392.87(392.66 to 393.09) |
| Congenital heart anomalies | Female | 2016 | 12635715.71(12613776.59 to 12657654.82) | 381.24(381.03 to 381.45) |
| Congenital heart anomalies | Female | 2017 | 12264308.11(12242529.78 to 12286086.44) | 368.11(367.9 to 368.31) |
| Congenital heart anomalies | Female | 2018 | 11784913.41(11763390.51 to 11806436.32) | 353.42(353.22 to 353.63) |
| Congenital heart anomalies | Female | 2019 | 11344123.71(11322823.76 to 11365423.65) | 341.4(341.2 to 341.6) |
| Congenital heart anomalies | Female | 2020 | 10411706.46(10390889.23 to 10432523.69) | 315.68(315.49 to 315.87) |
| Congenital heart anomalies | Female | 2021 | 9885596.39(9865049.15 to 9906143.63) | 303.35(303.16 to 303.54) |
| Congenital heart anomalies | Female | 2022 | 9868787.39(9248460.12 to 10489114.66) | 297.88(281.4 to 314.37) |
| Congenital heart anomalies | Female | 2023 | 9489713.96(8799670 to 10179757.92) | 286.3(267.54 to 305.06) |
| Congenital heart anomalies | Female | 2024 | 9119169.61(8334189.62 to 9904149.61) | 275.13(253.31 to 296.94) |
| Congenital heart anomalies | Female | 2025 | 8757053.82(7859029.38 to 9655078.26) | 264.36(238.96 to 289.76) |
| Congenital heart anomalies | Female | 2026 | 8405598.33(7382708.32 to 9428488.34) | 253.99(224.66 to 283.32) |
| Congenital heart anomalies | Female | 2027 | 8065973.31(6911336.35 to 9220610.28) | 244.02(210.58 to 277.45) |
| Congenital heart anomalies | Female | 2028 | 7739037.82(6449484.91 to 9028590.73) | 234.41(196.78 to 272.04) |
| Congenital heart anomalies | Female | 2029 | 7425440.88(6000302.89 to 8850578.87) | 225.16(183.35 to 266.98) |
| Congenital heart anomalies | Female | 2030 | 7125813.89(5566003.92 to 8685623.86) | 216.26(170.31 to 262.22) |
| Congenital heart anomalies | Female | 2031 | 6840408.11(5147831.95 to 8532984.28) | 207.7(157.7 to 257.7) |
| Congenital heart anomalies | Female | 2032 | 6568548.86(4745886.97 to 8391210.75) | 199.47(145.55 to 253.39) |
| Congenital heart anomalies | Female | 2033 | 6309440.01(4360012.75 to 8258867.28) | 191.55(133.85 to 249.25) |
| Congenital heart anomalies | Female | 2034 | 6062512.34(3989954.17 to 8135070.51) | 183.94(122.63 to 245.25) |
| Congenital heart anomalies | Female | 2035 | 5827650.49(3635586.43 to 8019714.56) | 176.62(111.86 to 241.37) |
| Congenital heart anomalies | Female | 2036 | 5604542.43(3296500 to 7912584.85) | 169.58(101.57 to 237.59) |
| Congenital heart anomalies | Female | 2037 | 5392783.98(2972112.44 to 7813455.51) | 162.82(91.74 to 233.89) |
| Congenital heart anomalies | Female | 2038 | 5191418.27(2661503.11 to 7721333.44) | 156.32(82.36 to 230.27) |
| Congenital heart anomalies | Female | 2039 | 4999537.08(2363778.44 to 7635295.72) | 150.07(73.43 to 226.71) |
| Congenital heart anomalies | Female | 2040 | 4816745.08(2078292.12 to 7555198.05) | 144.07(64.94 to 223.2) |
| Congenital heart anomalies | Male | 1990 | 27117599.83(27090381.27 to 27144818.38) | 858.04(857.72 to 858.37) |
| Congenital heart anomalies | Male | 1991 | 26615557.31(26588451.43 to 26642663.19) | 838.71(838.39 to 839.03) |
| Congenital heart anomalies | Male | 1992 | 26025926.39(25998947.73 to 26052905.05) | 818.66(818.35 to 818.98) |
| Congenital heart anomalies | Male | 1993 | 25392332.14(25365513.36 to 25419150.92) | 798.89(798.58 to 799.2) |
| Congenital heart anomalies | Male | 1994 | 24744857.17(24718237.43 to 24771476.91) | 780.12(779.81 to 780.43) |
| Congenital heart anomalies | Male | 1995 | 24125270.96(24098823.25 to 24151718.68) | 762.9(762.59 to 763.2) |
| Congenital heart anomalies | Male | 1996 | 23414742.52(23388534.2 to 23440950.84) | 742.01(741.71 to 742.31) |
| Congenital heart anomalies | Male | 1997 | 22804971.29(22778970.35 to 22830972.22) | 723.52(723.23 to 723.82) |
| Congenital heart anomalies | Male | 1998 | 22190162(22164338.77 to 22215985.22) | 704.44(704.14 to 704.73) |
| Congenital heart anomalies | Male | 1999 | 21640533(21614866.36 to 21666199.65) | 687.01(686.72 to 687.3) |
| Congenital heart anomalies | Male | 2000 | 21211004.46(21185445.38 to 21236563.54) | 672.76(672.47 to 673.05) |
| Congenital heart anomalies | Male | 2001 | 20810491.34(20785047.9 to 20835934.79) | 658.8(658.51 to 659.08) |
| Congenital heart anomalies | Male | 2002 | 20459833.3(20434550.99 to 20485115.6) | 645.64(645.36 to 645.92) |
| Congenital heart anomalies | Male | 2003 | 20063413.48(20038267.78 to 20088559.17) | 630.09(629.81 to 630.36) |
| Congenital heart anomalies | Male | 2004 | 19792071.27(19766968.05 to 19817174.49) | 617.75(617.48 to 618.02) |
| Congenital heart anomalies | Male | 2005 | 19508245.77(19483221.65 to 19533269.89) | 604.56(604.29 to 604.83) |
| Congenital heart anomalies | Male | 2006 | 19243120.56(19218237.13 to 19268003.98) | 591.33(591.07 to 591.6) |
| Congenital heart anomalies | Male | 2007 | 19000440.9(18975664.46 to 19025217.34) | 578.13(577.87 to 578.39) |
| Congenital heart anomalies | Male | 2008 | 18763490.59(18738784.23 to 18788196.94) | 564.71(564.46 to 564.97) |
| Congenital heart anomalies | Male | 2009 | 18447677.6(18423054.93 to 18472300.26) | 549.07(548.82 to 549.32) |
| Congenital heart anomalies | Male | 2010 | 18016986.84(17992522.03 to 18041451.64) | 531.32(531.07 to 531.56) |
| Congenital heart anomalies | Male | 2011 | 17606220.4(17581950.53 to 17630490.26) | 515.83(515.59 to 516.07) |
| Congenital heart anomalies | Male | 2012 | 17347638(17323477.99 to 17371798.01) | 505.29(505.06 to 505.53) |
| Congenital heart anomalies | Male | 2013 | 17084955.56(17060920.63 to 17108990.48) | 494.71(494.47 to 494.94) |
| Congenital heart anomalies | Male | 2014 | 16723954.03(16700068.59 to 16747839.47) | 481.52(481.29 to 481.75) |
| Congenital heart anomalies | Male | 2015 | 16391194.51(16367442.75 to 16414946.28) | 468.74(468.51 to 468.97) |
| Congenital heart anomalies | Male | 2016 | 15931423.44(15907845.54 to 15955001.35) | 451.98(451.76 to 452.2) |
| Congenital heart anomalies | Male | 2017 | 15329443.4(15306099.71 to 15352787.1) | 432.54(432.32 to 432.75) |
| Congenital heart anomalies | Male | 2018 | 14652888.73(14629843.42 to 14675934.03) | 413.13(412.91 to 413.34) |
| Congenital heart anomalies | Male | 2019 | 13999136.22(13976357.74 to 14021914.71) | 396.2(395.99 to 396.41) |
| Congenital heart anomalies | Male | 2020 | 13160583.85(13138216.91 to 13182950.8) | 375.81(375.61 to 376.01) |
| Congenital heart anomalies | Male | 2021 | 12424568.89(12402595.14 to 12446542.65) | 359.58(359.38 to 359.78) |
| Congenital heart anomalies | Male | 2022 | 12265161.43(11410747.74 to 13119575.11) | 348.89(327.19 to 370.58) |
| Congenital heart anomalies | Male | 2023 | 11775685.19(10835819.05 to 12715551.33) | 335.04(310.71 to 359.36) |
| Congenital heart anomalies | Male | 2024 | 11299572.33(10243368.84 to 12355775.83) | 321.72(293.87 to 349.57) |
| Congenital heart anomalies | Male | 2025 | 10835989.3(9641261.65 to 12030716.95) | 308.91(276.9 to 340.93) |
| Congenital heart anomalies | Male | 2026 | 10386920.16(9039189.52 to 11734650.8) | 296.6(260.01 to 333.18) |
| Congenital heart anomalies | Male | 2027 | 9953531.84(8444438.68 to 11462625) | 284.76(243.38 to 326.14) |
| Congenital heart anomalies | Male | 2028 | 9537101.82(7862886.13 to 11211317.51) | 273.38(227.11 to 319.66) |
| Congenital heart anomalies | Male | 2029 | 9138543.85(7298556.49 to 10978531.21) | 262.45(211.28 to 313.62) |
| Congenital heart anomalies | Male | 2030 | 8758615.43(6754174.6 to 10763056.27) | 251.93(195.93 to 307.94) |
| Congenital heart anomalies | Male | 2031 | 8397351.53(6231042.95 to 10563660.11) | 241.83(181.1 to 302.56) |
| Congenital heart anomalies | Male | 2032 | 8053974.03(5729321.65 to 10378626.4) | 232.12(166.81 to 297.43) |
| Congenital heart anomalies | Male | 2033 | 7727548.62(5248816.89 to 10206280.36) | 222.8(153.08 to 292.51) |
| Congenital heart anomalies | Male | 2034 | 7417391.57(4789162.07 to 10045621.08) | 213.84(139.91 to 287.77) |
| Congenital heart anomalies | Male | 2035 | 7123234.33(4350027.74 to 9896440.93) | 205.24(127.3 to 283.17) |
| Congenital heart anomalies | Male | 2036 | 6844471.89(3930672.47 to 9758271.31) | 196.98(115.25 to 278.7) |
| Congenital heart anomalies | Male | 2037 | 6580538.12(3530271.69 to 9630804.55) | 189.04(103.75 to 274.33) |
| Congenital heart anomalies | Male | 2038 | 6330268.91(3147643.23 to 9512894.6) | 181.42(92.8 to 270.05) |
| Congenital heart anomalies | Male | 2039 | 6092395.64(2781532.48 to 9403258.8) | 174.11(82.37 to 265.85) |
| Congenital heart anomalies | Male | 2040 | 5866238.36(2430969.64 to 9301507.09) | 167.09(72.47 to 261.71) |
| Neural tube defects | Both | 1990 | 10205223.01(10184440.18 to 10226006.54) | 165.83(165.47 to 166.18) |
| Neural tube defects | Both | 1991 | 10043510.69(10022825 to 10064197.03) | 162.72(162.36 to 163.07) |
| Neural tube defects | Both | 1992 | 9897822.45(9877191.69 to 9918453.8) | 160.25(159.9 to 160.6) |
| Neural tube defects | Both | 1993 | 9759616.21(9739034.15 to 9780198.81) | 158.22(157.88 to 158.57) |
| Neural tube defects | Both | 1994 | 9600631.86(9580113.54 to 9621150.64) | 156.14(155.79 to 156.48) |
| Neural tube defects | Both | 1995 | 9438418.95(9417969.33 to 9458868.93) | 154.12(153.78 to 154.46) |
| Neural tube defects | Both | 1996 | 9246635.9(9226292.49 to 9266979.55) | 151.44(151.1 to 151.78) |
| Neural tube defects | Both | 1997 | 9050637.38(9030406.85 to 9070868) | 148.51(148.17 to 148.84) |
| Neural tube defects | Both | 1998 | 8853803.64(8833694.22 to 8873913.05) | 145.45(145.11 to 145.78) |
| Neural tube defects | Both | 1999 | 8676829.73(8656811.56 to 8696847.9) | 142.6(142.27 to 142.93) |
| Neural tube defects | Both | 2000 | 8498425.92(8478514.82 to 8518337.02) | 139.6(139.28 to 139.93) |
| Neural tube defects | Both | 2001 | 8312162.93(8292372.49 to 8331953.36) | 136.33(136.01 to 136.65) |
| Neural tube defects | Both | 2002 | 8111858.99(8092212.47 to 8131505.51) | 132.63(132.31 to 132.95) |
| Neural tube defects | Both | 2003 | 7939871.1(7920328.96 to 7959413.25) | 129.21(128.89 to 129.52) |
| Neural tube defects | Both | 2004 | 7801966.62(7782494.98 to 7821438.25) | 126.2(125.89 to 126.51) |
| Neural tube defects | Both | 2005 | 7673776.96(7654356.24 to 7693197.68) | 123.23(122.93 to 123.54) |
| Neural tube defects | Both | 2006 | 7570685.47(7551307.66 to 7590063.28) | 120.52(120.22 to 120.82) |
| Neural tube defects | Both | 2007 | 7465613.33(7446282.29 to 7484944.37) | 117.64(117.34 to 117.94) |
| Neural tube defects | Both | 2008 | 7362736.23(7343441.5 to 7382030.97) | 114.72(114.43 to 115.02) |
| Neural tube defects | Both | 2009 | 7263323.54(7244054.57 to 7282592.5) | 111.89(111.6 to 112.18) |
| Neural tube defects | Both | 2010 | 7133250.43(7114046.07 to 7152454.79) | 108.84(108.56 to 109.13) |
| Neural tube defects | Both | 2011 | 7017931.72(6998794.3 to 7037069.13) | 106.36(106.08 to 106.64) |
| Neural tube defects | Both | 2012 | 6931156.5(6912080.76 to 6950232.24) | 104.42(104.14 to 104.7) |
| Neural tube defects | Both | 2013 | 6857925.36(6838894.35 to 6876956.38) | 102.71(102.44 to 102.99) |
| Neural tube defects | Both | 2014 | 6758944.29(6739980.46 to 6777908.13) | 100.68(100.41 to 100.95) |
| Neural tube defects | Both | 2015 | 6659070.86(6640162.76 to 6677978.96) | 98.54(98.27 to 98.81) |
| Neural tube defects | Both | 2016 | 6520839.62(6501999.02 to 6539680.22) | 95.76(95.49 to 96.03) |
| Neural tube defects | Both | 2017 | 6302772.32(6284075.57 to 6321469.07) | 92.1(91.84 to 92.36) |
| Neural tube defects | Both | 2018 | 6060460.76(6041946.54 to 6078974.97) | 88.55(88.29 to 88.8) |
| Neural tube defects | Both | 2019 | 5833679.31(5815333.24 to 5852025.37) | 85.63(85.38 to 85.89) |
| Neural tube defects | Both | 2020 | 5525968.72(5507893.46 to 5544043.97) | 81.93(81.68 to 82.18) |
| Neural tube defects | Both | 2021 | 5294517.66(5276610.95 to 5312424.36) | 79.66(79.41 to 79.91) |
| Neural tube defects | Both | 2022 | 5172933.37(4864213.83 to 5481652.9) | 76.51(71.94 to 81.08) |
| Neural tube defects | Both | 2023 | 4974951.84(4620651.32 to 5329252.37) | 73.63(68.37 to 78.89) |
| Neural tube defects | Both | 2024 | 4781857.99(4367703.16 to 5196012.82) | 70.87(64.71 to 77.03) |
| Neural tube defects | Both | 2025 | 4593369.18(4109865.28 to 5076873.08) | 68.22(61 to 75.43) |
| Neural tube defects | Both | 2026 | 4410425.25(3851791.53 to 4969058.96) | 65.68(57.31 to 74.04) |
| Neural tube defects | Both | 2027 | 4233596.49(3596715.97 to 4870477) | 63.24(53.67 to 72.81) |
| Neural tube defects | Both | 2028 | 4063558.14(3347187.49 to 4779928.8) | 60.9(50.1 to 71.71) |
| Neural tube defects | Both | 2029 | 3900929.06(3105010.49 to 4696847.62) | 58.67(46.61 to 70.73) |
| Neural tube defects | Both | 2030 | 3746065.51(2871265.99 to 4620865.03) | 56.54(43.23 to 69.84) |
| Neural tube defects | Both | 2031 | 3598953.02(2646388.4 to 4551517.64) | 54.5(39.96 to 69.04) |
| Neural tube defects | Both | 2032 | 3459170.1(2430295.31 to 4488044.89) | 52.55(36.79 to 68.32) |
| Neural tube defects | Both | 2033 | 3326368.76(2222890.75 to 4429846.77) | 50.7(33.73 to 67.67) |
| Neural tube defects | Both | 2034 | 3200340.92(2024032.2 to 4376649.63) | 48.93(30.77 to 67.08) |
| Neural tube defects | Both | 2035 | 3080943.66(1833510.56 to 4328376.77) | 47.24(27.92 to 66.55) |
| Neural tube defects | Both | 2036 | 2967843.8(1650917.53 to 4284770.07) | 45.63(25.17 to 66.09) |
| Neural tube defects | Both | 2037 | 2860720.64(1475813.42 to 4245627.86) | 44.1(22.52 to 65.68) |
| Neural tube defects | Both | 2038 | 2759148.87(1307721.39 to 4210576.36) | 42.65(19.96 to 65.33) |
| Neural tube defects | Both | 2039 | 2662656.29(1146129.42 to 4179183.16) | 41.27(17.49 to 65.04) |
| Neural tube defects | Both | 2040 | 2570987.29(990614.91 to 4151359.67) | 39.95(15.1 to 64.81) |
| Neural tube defects | Female | 1990 | 5348592.39(5337983.51 to 5359201.27) | 179.22(179.06 to 179.37) |
| Neural tube defects | Female | 1991 | 5265115.2(5254561.48 to 5275668.91) | 176.04(175.89 to 176.19) |
| Neural tube defects | Female | 1992 | 5190738.79(5180210.12 to 5201267.47) | 173.56(173.41 to 173.71) |
| Neural tube defects | Female | 1993 | 5113866.68(5103365.7 to 5124367.67) | 171.32(171.17 to 171.47) |
| Neural tube defects | Female | 1994 | 5020183.47(5009720.11 to 5030646.83) | 168.8(168.65 to 168.94) |
| Neural tube defects | Female | 1995 | 4925816.63(4915395.75 to 4936237.51) | 166.37(166.22 to 166.51) |
| Neural tube defects | Female | 1996 | 4808564.33(4798212.38 to 4818916.28) | 162.95(162.8 to 163.09) |
| Neural tube defects | Female | 1997 | 4691444.49(4681162.61 to 4701726.37) | 159.32(159.17 to 159.46) |
| Neural tube defects | Female | 1998 | 4579771.77(4569560.88 to 4589982.66) | 155.73(155.59 to 155.87) |
| Neural tube defects | Female | 1999 | 4481391.73(4471228.39 to 4491555.07) | 152.46(152.32 to 152.6) |
| Neural tube defects | Female | 2000 | 4380071.81(4369973.57 to 4390170.05) | 148.95(148.81 to 149.09) |
| Neural tube defects | Female | 2001 | 4278412.39(4268379.33 to 4288445.44) | 145.27(145.13 to 145.4) |
| Neural tube defects | Female | 2002 | 4188154.41(4178182.81 to 4198126) | 141.77(141.63 to 141.9) |
| Neural tube defects | Female | 2003 | 4106492.37(4096565.41 to 4116419.33) | 138.34(138.21 to 138.48) |
| Neural tube defects | Female | 2004 | 4038018.04(4028123.93 to 4047912.15) | 135.21(135.08 to 135.34) |
| Neural tube defects | Female | 2005 | 3970707.89(3960841.02 to 3980574.75) | 131.99(131.86 to 132.12) |
| Neural tube defects | Female | 2006 | 3917772.29(3907928.79 to 3927615.8) | 129.07(128.95 to 129.2) |
| Neural tube defects | Female | 2007 | 3863302.46(3853486.4 to 3873118.53) | 125.97(125.84 to 126.09) |
| Neural tube defects | Female | 2008 | 3808351.35(3798555.47 to 3818147.24) | 122.77(122.64 to 122.89) |
| Neural tube defects | Female | 2009 | 3760233.12(3750445.61 to 3770020.63) | 119.81(119.69 to 119.93) |
| Neural tube defects | Female | 2010 | 3696811.87(3687052.29 to 3706571.45) | 116.65(116.53 to 116.77) |
| Neural tube defects | Female | 2011 | 3640466.91(3630738.18 to 3650195.64) | 114.05(113.94 to 114.17) |
| Neural tube defects | Female | 2012 | 3606013.99(3596305.3 to 3615722.67) | 112.28(112.16 to 112.39) |
| Neural tube defects | Female | 2013 | 3587154.35(3577445.21 to 3596863.49) | 111.03(110.91 to 111.14) |
| Neural tube defects | Female | 2014 | 3548398.4(3538704.45 to 3558092.36) | 109.23(109.11 to 109.34) |
| Neural tube defects | Female | 2015 | 3500304.69(3490631.76 to 3509977.62) | 107.05(106.94 to 107.16) |
| Neural tube defects | Female | 2016 | 3428842.29(3419196.42 to 3438488.17) | 104.08(103.97 to 104.19) |
| Neural tube defects | Female | 2017 | 3307097.73(3297527.67 to 3316667.79) | 99.9(99.8 to 100.01) |
| Neural tube defects | Female | 2018 | 3171661.72(3162189.22 to 3181134.23) | 95.81(95.7 to 95.91) |
| Neural tube defects | Female | 2019 | 3041346.57(3031970.7 to 3050722.44) | 92.3(92.19 to 92.4) |
| Neural tube defects | Female | 2020 | 2847531.11(2838323.55 to 2856738.67) | 87.26(87.16 to 87.36) |
| Neural tube defects | Female | 2021 | 2715997.95(2706888.3 to 2725107.6) | 84.42(84.32 to 84.52) |
| Neural tube defects | Female | 2022 | 2653236.52(2495875.21 to 2810597.83) | 81.04(76.57 to 85.52) |
| Neural tube defects | Female | 2023 | 2540800.91(2360998.25 to 2720603.56) | 77.62(72.43 to 82.81) |
| Neural tube defects | Female | 2024 | 2431659.62(2222465.01 to 2640854.23) | 74.34(68.23 to 80.46) |
| Neural tube defects | Female | 2025 | 2325654.78(2082603.05 to 2568706.52) | 71.2(64.02 to 78.37) |
| Neural tube defects | Female | 2026 | 2223264.43(1943801.58 to 2502727.27) | 68.18(59.86 to 76.5) |
| Neural tube defects | Female | 2027 | 2124759.12(1807689.25 to 2441828.98) | 65.28(55.78 to 74.79) |
| Neural tube defects | Female | 2028 | 2030425.9(1675500.44 to 2385351.35) | 62.51(51.82 to 73.2) |
| Neural tube defects | Female | 2029 | 1940518.79(1548082.59 to 2332954.98) | 59.85(47.99 to 71.72) |
| Neural tube defects | Female | 2030 | 1855154.48(1425911.16 to 2284397.81) | 57.31(44.3 to 70.32) |
| Neural tube defects | Female | 2031 | 1774286.66(1309154.18 to 2239419.15) | 54.86(40.74 to 68.99) |
| Neural tube defects | Female | 2032 | 1697662.89(1197716.82 to 2197608.96) | 52.53(37.34 to 67.71) |
| Neural tube defects | Female | 2033 | 1625057.52(1091489 to 2158626.05) | 50.28(34.08 to 66.49) |
| Neural tube defects | Female | 2034 | 1556319.39(990342.6 to 2122296.17) | 48.14(30.97 to 65.31) |
| Neural tube defects | Female | 2035 | 1491326.47(894116.94 to 2088535.99) | 46.08(28 to 64.16) |
| Neural tube defects | Female | 2036 | 1429875.38(802565.36 to 2057185.4) | 44.11(25.18 to 63.05) |
| Neural tube defects | Female | 2037 | 1371779.55(715434 to 2028125.11) | 42.23(22.5 to 61.95) |
| Neural tube defects | Female | 2038 | 1316803.57(632452.41 to 2001154.72) | 40.42(19.96 to 60.88) |
| Neural tube defects | Female | 2039 | 1264688.85(553333.99 to 1976043.72) | 38.69(17.55 to 59.83) |
| Neural tube defects | Female | 2040 | 1215277.09(477836.03 to 1952718.15) | 37.03(15.28 to 58.79) |
| Neural tube defects | Male | 1990 | 4856630.62(4846456.67 to 4866805.27) | 153.23(153.09 to 153.36) |
| Neural tube defects | Male | 1991 | 4778395.49(4768263.52 to 4788528.11) | 150.2(150.07 to 150.34) |
| Neural tube defects | Male | 1992 | 4707083.66(4696981.58 to 4717186.33) | 147.77(147.64 to 147.9) |
| Neural tube defects | Male | 1993 | 4645749.53(4635668.45 to 4655831.14) | 145.95(145.82 to 146.08) |
| Neural tube defects | Male | 1994 | 4580448.39(4570393.43 to 4590503.81) | 144.28(144.15 to 144.41) |
| Neural tube defects | Male | 1995 | 4512602.32(4502573.57 to 4522631.42) | 142.67(142.54 to 142.8) |
| Neural tube defects | Male | 1996 | 4438071.58(4428080.11 to 4448063.27) | 140.68(140.55 to 140.82) |
| Neural tube defects | Male | 1997 | 4359192.89(4349244.24 to 4369141.63) | 138.41(138.28 to 138.54) |
| Neural tube defects | Male | 1998 | 4274031.87(4264133.34 to 4283930.4) | 135.84(135.71 to 135.97) |
| Neural tube defects | Male | 1999 | 4195437.99(4185583.16 to 4205292.82) | 133.39(133.27 to 133.52) |
| Neural tube defects | Male | 2000 | 4118354.11(4108541.25 to 4128166.98) | 130.87(130.75 to 131) |
| Neural tube defects | Male | 2001 | 4033750.54(4023993.16 to 4043507.91) | 127.98(127.85 to 128.1) |
| Neural tube defects | Male | 2002 | 3923704.59(3914029.66 to 3933379.51) | 124.1(123.98 to 124.22) |
| Neural tube defects | Male | 2003 | 3833378.73(3823763.54 to 3842993.92) | 120.67(120.55 to 120.79) |
| Neural tube defects | Male | 2004 | 3763948.58(3754371.05 to 3773526.11) | 117.78(117.66 to 117.9) |
| Neural tube defects | Male | 2005 | 3703069.07(3693515.22 to 3712622.92) | 115.06(114.94 to 115.17) |
| Neural tube defects | Male | 2006 | 3652913.18(3643378.87 to 3662447.48) | 112.53(112.41 to 112.64) |
| Neural tube defects | Male | 2007 | 3602310.87(3592795.89 to 3611825.85) | 109.86(109.75 to 109.97) |
| Neural tube defects | Male | 2008 | 3554384.88(3544886.03 to 3563883.73) | 107.2(107.09 to 107.31) |
| Neural tube defects | Male | 2009 | 3503090.42(3493608.96 to 3512571.87) | 104.47(104.36 to 104.58) |
| Neural tube defects | Male | 2010 | 3436438.56(3426993.78 to 3445883.34) | 101.54(101.44 to 101.65) |
| Neural tube defects | Male | 2011 | 3377464.81(3368056.12 to 3386873.49) | 99.15(99.04 to 99.26) |
| Neural tube defects | Male | 2012 | 3325142.52(3315775.46 to 3334509.57) | 97.05(96.95 to 97.16) |
| Neural tube defects | Male | 2013 | 3270771.01(3261449.14 to 3280092.88) | 94.92(94.82 to 95.03) |
| Neural tube defects | Male | 2014 | 3210545.89(3201276 to 3219815.77) | 92.67(92.57 to 92.77) |
| Neural tube defects | Male | 2015 | 3158766.17(3149531 to 3168001.34) | 90.57(90.47 to 90.67) |
| Neural tube defects | Male | 2016 | 3091997.33(3082802.61 to 3101192.05) | 87.97(87.87 to 88.06) |
| Neural tube defects | Male | 2017 | 2995674.59(2986547.9 to 3004801.28) | 84.79(84.69 to 84.88) |
| Neural tube defects | Male | 2018 | 2888799.03(2879757.33 to 2897840.74) | 81.75(81.65 to 81.84) |
| Neural tube defects | Male | 2019 | 2792332.74(2783362.54 to 2801302.93) | 79.39(79.3 to 79.48) |
| Neural tube defects | Male | 2020 | 2678437.61(2669569.91 to 2687305.31) | 76.94(76.85 to 77.03) |
| Neural tube defects | Male | 2021 | 2578519.7(2569722.65 to 2587316.76) | 75.18(75.09 to 75.28) |
| Neural tube defects | Male | 2022 | 2519696.85(2368338.63 to 2671055.07) | 72.19(68.16 to 76.22) |
| Neural tube defects | Male | 2023 | 2434150.94(2259653.06 to 2608648.81) | 69.8(65.08 to 74.51) |
| Neural tube defects | Male | 2024 | 2350198.37(2145238.15 to 2555158.59) | 67.47(61.86 to 73.09) |
| Neural tube defects | Male | 2025 | 2267714.4(2027262.23 to 2508166.56) | 65.23(58.57 to 71.89) |
| Neural tube defects | Male | 2026 | 2187160.82(1907989.95 to 2466331.69) | 63.05(55.25 to 70.85) |
| Neural tube defects | Male | 2027 | 2108837.37(1789026.72 to 2428648.02) | 60.94(51.95 to 69.94) |
| Neural tube defects | Male | 2028 | 2033132.25(1671687.05 to 2394577.45) | 58.9(48.68 to 69.12) |
| Neural tube defects | Male | 2029 | 1960410.27(1556927.89 to 2363892.64) | 56.93(45.48 to 68.39) |
| Neural tube defects | Male | 2030 | 1890911.03(1445354.83 to 2336467.23) | 55.02(42.33 to 67.71) |
| Neural tube defects | Male | 2031 | 1824666.36(1337234.22 to 2312098.49) | 53.18(39.27 to 67.09) |
| Neural tube defects | Male | 2032 | 1761507.21(1232578.49 to 2290435.93) | 51.39(36.28 to 66.5) |
| Neural tube defects | Male | 2033 | 1701311.24(1131401.75 to 2271220.73) | 49.66(33.39 to 65.94) |
| Neural tube defects | Male | 2034 | 1644021.53(1033689.6 to 2254353.46) | 47.99(30.58 to 65.4) |
| Neural tube defects | Male | 2035 | 1589617.2(939393.62 to 2239840.77) | 46.38(27.86 to 64.89) |
| Neural tube defects | Male | 2036 | 1537968.42(848352.17 to 2227584.67) | 44.81(25.24 to 64.39) |
| Neural tube defects | Male | 2037 | 1488941.09(760379.42 to 2217502.75) | 43.3(22.71 to 63.89) |
| Neural tube defects | Male | 2038 | 1442345.31(675268.97 to 2209421.64) | 41.84(20.28 to 63.41) |
| Neural tube defects | Male | 2039 | 1397967.44(592795.43 to 2203139.44) | 40.43(17.93 to 62.92) |
| Neural tube defects | Male | 2040 | 1355710.2(512778.88 to 2198641.52) | 39.06(15.69 to 62.44) |
| Digestive congenital anomalies | Both | 1990 | 6835865.63(6820962.27 to 6850769.96) | 110.96(110.71 to 111.22) |
| Digestive congenital anomalies | Both | 1991 | 6750036.21(6735162.94 to 6764910.46) | 109.28(109.03 to 109.54) |
| Digestive congenital anomalies | Both | 1992 | 6654744.39(6639891.99 to 6669597.75) | 107.72(107.47 to 107.97) |
| Digestive congenital anomalies | Both | 1993 | 6554982.58(6540161.02 to 6569805.08) | 106.29(106.04 to 106.54) |
| Digestive congenital anomalies | Both | 1994 | 6444954.73(6430177.41 to 6459732.98) | 104.89(104.64 to 105.14) |
| Digestive congenital anomalies | Both | 1995 | 6338779.08(6324049.89 to 6353509.19) | 103.64(103.39 to 103.88) |
| Digestive congenital anomalies | Both | 1996 | 6217371.14(6202713.55 to 6232029.62) | 102(101.76 to 102.25) |
| Digestive congenital anomalies | Both | 1997 | 6106276.28(6091682.41 to 6120871.02) | 100.4(100.16 to 100.65) |
| Digestive congenital anomalies | Both | 1998 | 6004762.46(5990224.68 to 6019301.06) | 98.88(98.64 to 99.12) |
| Digestive congenital anomalies | Both | 1999 | 5921976.8(5907471.63 to 5936482.72) | 97.59(97.35 to 97.83) |
| Digestive congenital anomalies | Both | 2000 | 5855500.51(5841019.74 to 5869981.98) | 96.47(96.23 to 96.71) |
| Digestive congenital anomalies | Both | 2001 | 5781644.69(5767198.38 to 5796091.63) | 95.12(94.88 to 95.36) |
| Digestive congenital anomalies | Both | 2002 | 5747141.09(5732694.6 to 5761588.15) | 94.27(94.03 to 94.51) |
| Digestive congenital anomalies | Both | 2003 | 5713287.58(5698833.1 to 5727742.56) | 93.28(93.04 to 93.51) |
| Digestive congenital anomalies | Both | 2004 | 5691725.6(5677255.98 to 5706195.64) | 92.36(92.13 to 92.6) |
| Digestive congenital anomalies | Both | 2005 | 5673611.75(5659116.14 to 5688107.7) | 91.4(91.17 to 91.63) |
| Digestive congenital anomalies | Both | 2006 | 5675626.52(5661102.4 to 5690150.89) | 90.63(90.4 to 90.86) |
| Digestive congenital anomalies | Both | 2007 | 5667064.95(5652522.88 to 5681607.19) | 89.56(89.33 to 89.79) |
| Digestive congenital anomalies | Both | 2008 | 5661951.38(5647378.72 to 5676524.11) | 88.46(88.23 to 88.68) |
| Digestive congenital anomalies | Both | 2009 | 5643050.48(5628460.95 to 5657640.01) | 87.15(86.93 to 87.37) |
| Digestive congenital anomalies | Both | 2010 | 5592667.95(5578089.45 to 5607246.45) | 85.54(85.32 to 85.76) |
| Digestive congenital anomalies | Both | 2011 | 5555273.85(5540700.16 to 5569847.55) | 84.4(84.18 to 84.61) |
| Digestive congenital anomalies | Both | 2012 | 5562796.2(5548192.51 to 5577399.89) | 84.01(83.8 to 84.23) |
| Digestive congenital anomalies | Both | 2013 | 5562446.49(5547815.44 to 5577077.55) | 83.53(83.31 to 83.74) |
| Digestive congenital anomalies | Both | 2014 | 5537440.04(5522804.93 to 5552075.15) | 82.71(82.5 to 82.92) |
| Digestive congenital anomalies | Both | 2015 | 5511297.06(5496655.85 to 5525938.27) | 81.79(81.58 to 82) |
| Digestive congenital anomalies | Both | 2016 | 5437447.14(5422834.32 to 5452059.95) | 80.08(79.88 to 80.29) |
| Digestive congenital anomalies | Both | 2017 | 5284320.56(5269818.87 to 5298822.25) | 77.46(77.26 to 77.67) |
| Digestive congenital anomalies | Both | 2018 | 5105037.23(5090679.95 to 5119394.51) | 74.87(74.66 to 75.07) |
| Digestive congenital anomalies | Both | 2019 | 4920377.64(4906164.47 to 4934590.81) | 72.56(72.36 to 72.76) |
| Digestive congenital anomalies | Both | 2020 | 4637721.71(4623749.33 to 4651694.1) | 69.16(68.97 to 69.36) |
| Digestive congenital anomalies | Both | 2021 | 4430717.32(4416923.08 to 4444511.56) | 67.16(66.96 to 67.35) |
| Digestive congenital anomalies | Both | 2022 | 4411858.89(4160930.93 to 4662786.85) | 65.7(61.96 to 69.43) |
| Digestive congenital anomalies | Both | 2023 | 4261270.81(3975216.31 to 4547325.32) | 63.53(59.27 to 67.8) |
| Digestive congenital anomalies | Both | 2024 | 4113276.8(3780964.85 to 4445588.74) | 61.44(56.47 to 66.41) |
| Digestive congenital anomalies | Both | 2025 | 3967676.53(3581372.31 to 4353980.76) | 59.43(53.64 to 65.23) |
| Digestive congenital anomalies | Both | 2026 | 3825409.02(3380139.46 to 4270678.59) | 57.49(50.79 to 64.2) |
| Digestive congenital anomalies | Both | 2027 | 3687067.94(3179901.35 to 4194234.54) | 55.62(47.96 to 63.29) |
| Digestive congenital anomalies | Both | 2028 | 3553252.91(2982691.63 to 4123814.2) | 53.83(45.16 to 62.49) |
| Digestive congenital anomalies | Both | 2029 | 3424612.19(2790090.96 to 4059133.42) | 52.1(42.42 to 61.78) |
| Digestive congenital anomalies | Both | 2030 | 3301589.34(2603141.41 to 4000037.28) | 50.44(39.74 to 61.14) |
| Digestive congenital anomalies | Both | 2031 | 3184274.06(2422336.26 to 3946211.87) | 48.84(37.12 to 60.57) |
| Digestive congenital anomalies | Both | 2032 | 3072380.34(2247711.42 to 3897049.26) | 47.31(34.57 to 60.06) |
| Digestive congenital anomalies | Both | 2033 | 2965534.78(2079151.72 to 3851917.84) | 45.85(32.09 to 59.6) |
| Digestive congenital anomalies | Both | 2034 | 2863668.74(1916677.99 to 3810659.49) | 44.44(29.69 to 59.19) |
| Digestive congenital anomalies | Both | 2035 | 2766758.85(1760258.26 to 3773259.44) | 43.09(27.35 to 58.83) |
| Digestive congenital anomalies | Both | 2036 | 2674586.7(1609668.44 to 3739504.96) | 41.8(25.09 to 58.51) |
| Digestive congenital anomalies | Both | 2037 | 2586941.87(1464655.14 to 3709228.61) | 40.56(22.89 to 58.23) |
| Digestive congenital anomalies | Both | 2038 | 2503435.21(1324850.22 to 3682020.19) | 39.38(20.76 to 58) |
| Digestive congenital anomalies | Both | 2039 | 2423667.36(1189884.23 to 3657450.48) | 38.25(18.69 to 57.81) |
| Digestive congenital anomalies | Both | 2040 | 2347464.16(1059505.67 to 3635422.65) | 37.17(16.68 to 57.66) |
| Digestive congenital anomalies | Female | 1990 | 3342789.87(3335416.76 to 3350162.98) | 111.92(111.8 to 112.04) |
| Digestive congenital anomalies | Female | 1991 | 3302477.03(3295117.24 to 3309836.82) | 110.37(110.25 to 110.49) |
| Digestive congenital anomalies | Female | 1992 | 3255398.79(3248047.17 to 3262750.42) | 108.85(108.73 to 108.97) |
| Digestive congenital anomalies | Female | 1993 | 3199730.96(3192400.47 to 3207061.44) | 107.25(107.13 to 107.36) |
| Digestive congenital anomalies | Female | 1994 | 3133951.03(3126650.39 to 3141251.67) | 105.48(105.36 to 105.59) |
| Digestive congenital anomalies | Female | 1995 | 3074592.17(3067320.2 to 3081864.14) | 103.99(103.88 to 104.11) |
| Digestive congenital anomalies | Female | 1996 | 3005336.8(2998105.9 to 3012567.7) | 102.04(101.92 to 102.15) |
| Digestive congenital anomalies | Female | 1997 | 2943783.93(2936586.5 to 2950981.37) | 100.2(100.08 to 100.31) |
| Digestive congenital anomalies | Female | 1998 | 2889705.5(2882536.94 to 2896874.05) | 98.52(98.4 to 98.63) |
| Digestive congenital anomalies | Female | 1999 | 2846638.42(2839486.66 to 2853790.19) | 97.13(97.02 to 97.24) |
| Digestive congenital anomalies | Female | 2000 | 2809414.89(2802278.3 to 2816551.48) | 95.84(95.73 to 95.95) |
| Digestive congenital anomalies | Female | 2001 | 2768518.98(2761404.31 to 2775633.65) | 94.31(94.2 to 94.42) |
| Digestive congenital anomalies | Female | 2002 | 2753777.79(2746661.97 to 2760893.62) | 93.53(93.42 to 93.64) |
| Digestive congenital anomalies | Female | 2003 | 2733285.84(2726171.49 to 2740400.19) | 92.4(92.29 to 92.51) |
| Digestive congenital anomalies | Female | 2004 | 2724004.94(2716884.01 to 2731125.88) | 91.52(91.42 to 91.63) |
| Digestive congenital anomalies | Female | 2005 | 2717067.2(2709933.78 to 2724200.63) | 90.62(90.51 to 90.73) |
| Digestive congenital anomalies | Female | 2006 | 2719353.23(2712206.21 to 2726500.25) | 89.88(89.78 to 89.99) |
| Digestive congenital anomalies | Female | 2007 | 2715491.54(2708336.97 to 2722646.12) | 88.82(88.71 to 88.92) |
| Digestive congenital anomalies | Female | 2008 | 2717309.15(2710135.99 to 2724482.3) | 87.85(87.75 to 87.96) |
| Digestive congenital anomalies | Female | 2009 | 2716416.29(2709226.58 to 2723606) | 86.79(86.69 to 86.9) |
| Digestive congenital anomalies | Female | 2010 | 2702623.82(2695429.75 to 2709817.89) | 85.51(85.41 to 85.61) |
| Digestive congenital anomalies | Female | 2011 | 2693160.86(2685962.01 to 2700359.72) | 84.61(84.51 to 84.71) |
| Digestive congenital anomalies | Female | 2012 | 2707327.32(2700105.71 to 2714548.93) | 84.53(84.43 to 84.63) |
| Digestive congenital anomalies | Female | 2013 | 2725413.88(2718162.65 to 2732665.11) | 84.6(84.5 to 84.7) |
| Digestive congenital anomalies | Female | 2014 | 2727479.21(2720212.81 to 2734745.6) | 84.22(84.12 to 84.31) |
| Digestive congenital anomalies | Female | 2015 | 2724107.1(2716828.41 to 2731385.78) | 83.58(83.48 to 83.67) |
| Digestive congenital anomalies | Female | 2016 | 2688606.78(2681341.5 to 2695872.07) | 81.88(81.79 to 81.98) |
| Digestive congenital anomalies | Female | 2017 | 2605493.6(2598291.92 to 2612695.27) | 78.99(78.9 to 79.09) |
| Digestive congenital anomalies | Female | 2018 | 2510246.92(2503122.74 to 2517371.1) | 76.15(76.05 to 76.24) |
| Digestive congenital anomalies | Female | 2019 | 2411424.29(2404378.51 to 2418470.08) | 73.56(73.46 to 73.65) |
| Digestive congenital anomalies | Female | 2020 | 2247882.43(2240965.8 to 2254799.06) | 69.32(69.23 to 69.41) |
| Digestive congenital anomalies | Female | 2021 | 2139387.86(2132578.44 to 2146197.28) | 67.04(66.95 to 67.13) |
| Digestive congenital anomalies | Female | 2022 | 2146840.75(2009681.41 to 2284000.08) | 66.05(61.97 to 70.14) |
| Digestive congenital anomalies | Female | 2023 | 2069699.13(1915922.16 to 2223476.11) | 63.73(59.12 to 68.34) |
| Digestive congenital anomalies | Female | 2024 | 1994042.64(1818468.65 to 2169616.64) | 61.49(56.18 to 66.79) |
| Digestive congenital anomalies | Female | 2025 | 1919790.65(1718772.92 to 2120808.37) | 59.32(53.21 to 65.43) |
| Digestive congenital anomalies | Female | 2026 | 1847409.89(1618595.78 to 2076224.01) | 57.23(50.23 to 64.23) |
| Digestive congenital anomalies | Female | 2027 | 1777195.91(1519220.16 to 2035171.67) | 55.21(47.28 to 63.14) |
| Digestive congenital anomalies | Female | 2028 | 1709420.54(1421625.08 to 1997216) | 53.26(44.37 to 62.15) |
| Digestive congenital anomalies | Female | 2029 | 1644378.15(1326569.2 to 1962187.1) | 51.38(41.52 to 61.24) |
| Digestive congenital anomalies | Female | 2030 | 1582258.27(1234542.77 to 1929973.78) | 49.57(38.75 to 60.39) |
| Digestive congenital anomalies | Female | 2031 | 1523086.6(1145774.15 to 1900399.04) | 47.82(36.05 to 59.59) |
| Digestive congenital anomalies | Female | 2032 | 1466711.65(1060272.6 to 1873150.7) | 46.13(33.43 to 58.83) |
| Digestive congenital anomalies | Female | 2033 | 1412937.34(977970.25 to 1847904.43) | 44.5(30.89 to 58.1) |
| Digestive congenital anomalies | Female | 2034 | 1361718.41(898864.76 to 1824572.07) | 42.93(28.45 to 57.41) |
| Digestive congenital anomalies | Female | 2035 | 1313027.62(822924.54 to 1803130.71) | 41.41(26.09 to 56.73) |
| Digestive congenital anomalies | Female | 2036 | 1266742.43(750025.83 to 1783459.03) | 39.95(23.81 to 56.08) |
| Digestive congenital anomalies | Female | 2037 | 1222759(680040.69 to 1765477.32) | 38.53(21.63 to 55.44) |
| Digestive congenital anomalies | Female | 2038 | 1180888.03(612785.67 to 1748990.39) | 37.17(19.53 to 54.81) |
| Digestive congenital anomalies | Female | 2039 | 1140937.65(548077.58 to 1733797.71) | 35.86(17.52 to 54.19) |
| Digestive congenital anomalies | Female | 2040 | 1102816.25(485783.89 to 1719848.6) | 34.59(15.6 to 53.58) |
| Digestive congenital anomalies | Male | 1990 | 3493075.76(3485545.51 to 3500606.99) | 110.06(109.95 to 110.18) |
| Digestive congenital anomalies | Male | 1991 | 3447559.18(3440045.7 to 3455073.64) | 108.27(108.15 to 108.38) |
| Digestive congenital anomalies | Male | 1992 | 3399345.6(3391844.82 to 3406847.34) | 106.66(106.54 to 106.77) |
| Digestive congenital anomalies | Male | 1993 | 3355251.62(3347760.55 to 3362743.64) | 105.4(105.29 to 105.51) |
| Digestive congenital anomalies | Male | 1994 | 3311003.7(3303527.03 to 3318481.3) | 104.34(104.23 to 104.46) |
| Digestive congenital anomalies | Male | 1995 | 3264186.92(3256729.69 to 3271645.05) | 103.3(103.19 to 103.41) |
| Digestive congenital anomalies | Male | 1996 | 3212034.34(3204607.64 to 3219461.92) | 101.97(101.86 to 102.08) |
| Digestive congenital anomalies | Male | 1997 | 3162492.35(3155095.91 to 3169889.65) | 100.6(100.49 to 100.71) |
| Digestive congenital anomalies | Male | 1998 | 3115056.96(3107687.74 to 3122427) | 99.22(99.11 to 99.33) |
| Digestive congenital anomalies | Male | 1999 | 3075338.38(3067984.98 to 3082692.54) | 98.03(97.92 to 98.13) |
| Digestive congenital anomalies | Male | 2000 | 3046085.62(3038741.44 to 3053430.5) | 97.07(96.96 to 97.17) |
| Digestive congenital anomalies | Male | 2001 | 3013125.7(3005794.07 to 3020457.97) | 95.88(95.77 to 95.98) |
| Digestive congenital anomalies | Male | 2002 | 2993363.3(2986032.63 to 3000694.53) | 94.96(94.85 to 95.07) |
| Digestive congenital anomalies | Male | 2003 | 2980001.74(2972661.61 to 2987342.36) | 94.1(93.99 to 94.2) |
| Digestive congenital anomalies | Male | 2004 | 2967720.66(2960371.97 to 2975069.76) | 93.15(93.04 to 93.25) |
| Digestive congenital anomalies | Male | 2005 | 2956544.55(2949182.36 to 2963907.07) | 92.14(92.03 to 92.24) |
| Digestive congenital anomalies | Male | 2006 | 2956273.29(2948896.2 to 2963650.65) | 91.33(91.22 to 91.43) |
| Digestive congenital anomalies | Male | 2007 | 2951573.41(2944185.92 to 2958961.07) | 90.25(90.15 to 90.36) |
| Digestive congenital anomalies | Male | 2008 | 2944642.23(2937242.72 to 2952041.81) | 89.03(88.93 to 89.13) |
| Digestive congenital anomalies | Male | 2009 | 2926634.19(2919234.38 to 2934034.01) | 87.48(87.38 to 87.58) |
| Digestive congenital anomalies | Male | 2010 | 2890044.13(2882659.7 to 2897428.56) | 85.58(85.48 to 85.68) |
| Digestive congenital anomalies | Male | 2011 | 2862112.99(2854738.15 to 2869487.84) | 84.2(84.1 to 84.3) |
| Digestive congenital anomalies | Male | 2012 | 2855468.88(2848086.8 to 2862850.96) | 83.53(83.43 to 83.63) |
| Digestive congenital anomalies | Male | 2013 | 2837032.61(2829652.78 to 2844412.44) | 82.53(82.43 to 82.62) |
| Digestive congenital anomalies | Male | 2014 | 2809960.83(2802592.12 to 2817329.55) | 81.31(81.21 to 81.4) |
| Digestive congenital anomalies | Male | 2015 | 2787189.96(2779827.44 to 2794552.49) | 80.12(80.02 to 80.21) |
| Digestive congenital anomalies | Male | 2016 | 2748840.35(2741492.82 to 2756187.88) | 78.4(78.31 to 78.49) |
| Digestive congenital anomalies | Male | 2017 | 2678826.96(2671526.95 to 2686126.97) | 76.03(75.94 to 76.12) |
| Digestive congenital anomalies | Male | 2018 | 2594790.32(2587557.22 to 2602023.41) | 73.67(73.58 to 73.76) |
| Digestive congenital anomalies | Male | 2019 | 2508953.35(2501785.96 to 2516120.73) | 71.63(71.54 to 71.72) |
| Digestive congenital anomalies | Male | 2020 | 2389839.29(2382783.53 to 2396895.04) | 69.02(68.93 to 69.1) |
| Digestive congenital anomalies | Male | 2021 | 2291329.46(2284344.64 to 2298314.28) | 67.27(67.18 to 67.36) |
| Digestive congenital anomalies | Male | 2022 | 2265018.15(2151249.52 to 2378786.77) | 65.31(62.15 to 68.48) |
| Digestive congenital anomalies | Male | 2023 | 2191571.68(2059294.15 to 2323849.21) | 63.28(59.56 to 67) |
| Digestive congenital anomalies | Male | 2024 | 2119234.15(1962496.2 to 2275972.1) | 61.31(56.87 to 65.75) |
| Digestive congenital anomalies | Male | 2025 | 2047885.89(1862599.38 to 2233172.4) | 59.4(54.11 to 64.68) |
| Digestive congenital anomalies | Male | 2026 | 1977999.13(1761543.68 to 2194454.58) | 57.55(51.33 to 63.76) |
| Digestive congenital anomalies | Male | 2027 | 1909872.03(1660681.19 to 2159062.87) | 55.75(48.56 to 62.94) |
| Digestive congenital anomalies | Male | 2028 | 1843832.38(1561066.55 to 2126598.2) | 54.01(45.81 to 62.21) |
| Digestive congenital anomalies | Male | 2029 | 1780234.04(1463521.75 to 2096946.32) | 52.32(43.11 to 61.54) |
| Digestive congenital anomalies | Male | 2030 | 1719331.07(1368598.65 to 2070063.5) | 50.69(40.45 to 60.93) |
| Digestive congenital anomalies | Male | 2031 | 1661187.47(1276562.11 to 2045812.83) | 49.11(37.85 to 60.37) |
| Digestive congenital anomalies | Male | 2032 | 1605668.69(1187438.82 to 2023898.56) | 47.57(35.31 to 59.84) |
| Digestive congenital anomalies | Male | 2033 | 1552597.44(1101181.47 to 2004013.42) | 46.09(32.84 to 59.34) |
| Digestive congenital anomalies | Male | 2034 | 1501950.33(1017813.23 to 1986087.43) | 44.65(30.43 to 58.86) |
| Digestive congenital anomalies | Male | 2035 | 1453731.23(937333.73 to 1970128.73) | 43.25(28.1 to 58.41) |
| Digestive congenital anomalies | Male | 2036 | 1407844.27(859642.61 to 1956045.92) | 41.9(25.84 to 57.97) |
| Digestive congenital anomalies | Male | 2037 | 1364182.87(784614.45 to 1943751.29) | 40.59(23.65 to 57.54) |
| Digestive congenital anomalies | Male | 2038 | 1322547.17(712064.54 to 1933029.8) | 39.32(21.53 to 57.12) |
| Digestive congenital anomalies | Male | 2039 | 1282729.71(641806.66 to 1923652.77) | 38.09(19.48 to 56.71) |
| Digestive congenital anomalies | Male | 2040 | 1244647.91(573721.78 to 1915574.05) | 36.9(17.51 to 56.3) |
| Down syndrome | Both | 1990 | 2803886.61(2789669.61 to 2818103.61) | 46.13(45.88 to 46.38) |
| Down syndrome | Both | 1991 | 2765817.16(2751606.99 to 2780027.33) | 45.33(45.08 to 45.58) |
| Down syndrome | Both | 1992 | 2724522.88(2710298.09 to 2738747.68) | 44.58(44.33 to 44.83) |
| Down syndrome | Both | 1993 | 2687893.49(2673630.34 to 2702156.63) | 43.98(43.74 to 44.23) |
| Down syndrome | Both | 1994 | 2647676.88(2633397.31 to 2661956.45) | 43.4(43.15 to 43.64) |
| Down syndrome | Both | 1995 | 2620090.31(2605764.5 to 2634416.11) | 43.05(42.8 to 43.29) |
| Down syndrome | Both | 1996 | 2587242.33(2572888.13 to 2601596.54) | 42.57(42.32 to 42.81) |
| Down syndrome | Both | 1997 | 2557338.52(2542941.17 to 2571735.87) | 42.09(41.85 to 42.33) |
| Down syndrome | Both | 1998 | 2533909.21(2519450.81 to 2548367.62) | 41.68(41.44 to 41.92) |
| Down syndrome | Both | 1999 | 2534581.66(2520001.03 to 2549162.29) | 41.65(41.41 to 41.89) |
| Down syndrome | Both | 2000 | 2526007.97(2511323.84 to 2540692.1) | 41.43(41.19 to 41.67) |
| Down syndrome | Both | 2001 | 2501051.26(2486295.29 to 2515807.24) | 40.89(40.65 to 41.13) |
| Down syndrome | Both | 2002 | 2482369.67(2467553.78 to 2497185.57) | 40.41(40.17 to 40.64) |
| Down syndrome | Both | 2003 | 2457729.97(2442840.02 to 2472619.91) | 39.76(39.52 to 40) |
| Down syndrome | Both | 2004 | 2435307.02(2420394.16 to 2450219.87) | 39.11(38.88 to 39.35) |
| Down syndrome | Both | 2005 | 2432153.94(2417160.2 to 2447147.68) | 38.74(38.51 to 38.98) |
| Down syndrome | Both | 2006 | 2439684.73(2424625.95 to 2454743.51) | 38.49(38.26 to 38.73) |
| Down syndrome | Both | 2007 | 2448343.09(2433210.43 to 2463475.75) | 38.22(37.99 to 38.45) |
| Down syndrome | Both | 2008 | 2469410.02(2454158.4 to 2484661.65) | 38.1(37.87 to 38.33) |
| Down syndrome | Both | 2009 | 2481959.7(2466585.42 to 2497333.99) | 37.83(37.61 to 38.06) |
| Down syndrome | Both | 2010 | 2478122.59(2462676.3 to 2493568.89) | 37.39(37.16 to 37.62) |
| Down syndrome | Both | 2011 | 2483412.18(2467870.34 to 2498954.02) | 37.18(36.95 to 37.4) |
| Down syndrome | Both | 2012 | 2511668.49(2495998.17 to 2527338.8) | 37.33(37.11 to 37.56) |
| Down syndrome | Both | 2013 | 2546880.49(2531039.16 to 2562721.82) | 37.58(37.36 to 37.81) |
| Down syndrome | Both | 2014 | 2580438.64(2564427.41 to 2596449.87) | 37.82(37.59 to 38.04) |
| Down syndrome | Both | 2015 | 2611783.91(2595593.99 to 2627973.82) | 37.97(37.75 to 38.2) |
| Down syndrome | Both | 2016 | 2645067.59(2628670.82 to 2661464.36) | 38.12(37.89 to 38.34) |
| Down syndrome | Both | 2017 | 2639026.56(2622529.68 to 2655523.43) | 37.78(37.56 to 38) |
| Down syndrome | Both | 2018 | 2596514.97(2580023.63 to 2613006.31) | 37.07(36.85 to 37.29) |
| Down syndrome | Both | 2019 | 2547963.19(2531492.72 to 2564433.65) | 36.43(36.21 to 36.65) |
| Down syndrome | Both | 2020 | 2426083.47(2409783.08 to 2442383.87) | 34.86(34.64 to 35.08) |
| Down syndrome | Both | 2021 | 2353125.76(2336905.72 to 2369345.8) | 34.13(33.91 to 34.34) |
| Down syndrome | Both | 2022 | 2339317.12(2154068.62 to 2524565.62) | 33.3(30.65 to 35.96) |
| Down syndrome | Both | 2023 | 2279203.76(2065213.03 to 2493194.48) | 32.37(29.3 to 35.44) |
| Down syndrome | Both | 2024 | 2219948.68(1967121.23 to 2472776.13) | 31.47(27.83 to 35.1) |
| Down syndrome | Both | 2025 | 2161505.3(1862332.78 to 2460677.81) | 30.6(26.29 to 34.9) |
| Down syndrome | Both | 2026 | 2104398.1(1753489.26 to 2455306.94) | 29.76(24.71 to 34.81) |
| Down syndrome | Both | 2027 | 2048977.79(1642501.31 to 2455454.27) | 28.95(23.09 to 34.81) |
| Down syndrome | Both | 2028 | 1995626.46(1530921.85 to 2460331.07) | 28.18(21.48 to 34.89) |
| Down syndrome | Both | 2029 | 1944550.78(1419693.02 to 2469408.54) | 27.44(19.87 to 35.02) |
| Down syndrome | Both | 2030 | 1895892.68(1309371.74 to 2482413.62) | 26.74(18.27 to 35.21) |
| Down syndrome | Both | 2031 | 1849766.58(1200257.81 to 2499275.35) | 26.07(16.68 to 35.46) |
| Down syndrome | Both | 2032 | 1806109.01(1092388.84 to 2519829.18) | 25.43(15.11 to 35.75) |
| Down syndrome | Both | 2033 | 1764822.97(985774.59 to 2543871.35) | 24.83(13.56 to 36.1) |
| Down syndrome | Both | 2034 | 1725922.47(880367.56 to 2571477.37) | 24.26(12.03 to 36.49) |
| Down syndrome | Both | 2035 | 1689407.21(775995.06 to 2602819.35) | 23.72(10.51 to 36.94) |
| Down syndrome | Both | 2036 | 1655243.58(672380.05 to 2638107.1) | 23.22(9 to 37.44) |
| Down syndrome | Both | 2037 | 1623376.33(569174.38 to 2677578.28) | 22.75(7.49 to 38) |
| Down syndrome | Both | 2038 | 1593709.58(466042.87 to 2721376.29) | 22.3(5.99 to 38.62) |
| Down syndrome | Both | 2039 | 1566096.98(362571.26 to 2769622.7) | 21.9(4.48 to 39.31) |
| Down syndrome | Both | 2040 | 1540474.46(263345.42 to 2822648.83) | 21.52(3.04 to 40.08) |
| Down syndrome | Female | 1990 | 1479675.24(1472402.91 to 1486947.56) | 50.12(50.04 to 50.2) |
| Down syndrome | Female | 1991 | 1459225.85(1451961.49 to 1466490.21) | 49.27(49.19 to 49.35) |
| Down syndrome | Female | 1992 | 1437631.94(1430358.31 to 1444905.57) | 48.49(48.41 to 48.57) |
| Down syndrome | Female | 1993 | 1417019.41(1409728.61 to 1424310.21) | 47.82(47.75 to 47.9) |
| Down syndrome | Female | 1994 | 1393754.44(1386455.49 to 1401053.4) | 47.14(47.06 to 47.21) |
| Down syndrome | Female | 1995 | 1376063.68(1368746.15 to 1383381.21) | 46.66(46.59 to 46.74) |
| Down syndrome | Female | 1996 | 1352903.11(1345582.29 to 1360223.94) | 45.95(45.87 to 46.03) |
| Down syndrome | Female | 1997 | 1336862.18(1329519.81 to 1344204.55) | 45.43(45.35 to 45.5) |
| Down syndrome | Female | 1998 | 1332318.44(1324932.16 to 1339704.72) | 45.25(45.18 to 45.33) |
| Down syndrome | Female | 1999 | 1334093.81(1326641.06 to 1341546.56) | 45.26(45.19 to 45.34) |
| Down syndrome | Female | 2000 | 1325712.55(1318215.42 to 1333209.68) | 44.89(44.81 to 44.97) |
| Down syndrome | Female | 2001 | 1312326.06(1304801.36 to 1319850.77) | 44.3(44.22 to 44.37) |
| Down syndrome | Female | 2002 | 1299136.63(1291594.18 to 1306679.07) | 43.65(43.58 to 43.73) |
| Down syndrome | Female | 2003 | 1285060.82(1277489.78 to 1292631.85) | 42.91(42.84 to 42.99) |
| Down syndrome | Female | 2004 | 1272758.35(1265184.57 to 1280332.13) | 42.19(42.12 to 42.26) |
| Down syndrome | Female | 2005 | 1270062.08(1262453.91 to 1277670.26) | 41.75(41.68 to 41.82) |
| Down syndrome | Female | 2006 | 1274829.51(1267195.59 to 1282463.44) | 41.51(41.44 to 41.58) |
| Down syndrome | Female | 2007 | 1281680.04(1274006.88 to 1289353.21) | 41.28(41.21 to 41.35) |
| Down syndrome | Female | 2008 | 1289387.52(1281658.06 to 1297116.98) | 41.03(40.96 to 41.11) |
| Down syndrome | Female | 2009 | 1294660.15(1286870.11 to 1302450.19) | 40.7(40.63 to 40.77) |
| Down syndrome | Female | 2010 | 1293962.88(1286136.28 to 1301789.48) | 40.25(40.19 to 40.32) |
| Down syndrome | Female | 2011 | 1298511.53(1290635.79 to 1306387.26) | 40.07(40 to 40.14) |
| Down syndrome | Female | 2012 | 1313141.61(1305206.1 to 1321077.13) | 40.23(40.16 to 40.3) |
| Down syndrome | Female | 2013 | 1334721.12(1326688.57 to 1342753.66) | 40.59(40.52 to 40.66) |
| Down syndrome | Female | 2014 | 1353502.34(1345381.04 to 1361623.64) | 40.88(40.81 to 40.95) |
| Down syndrome | Female | 2015 | 1368139.14(1359929.26 to 1376349.01) | 40.99(40.92 to 41.06) |
| Down syndrome | Female | 2016 | 1384483.7(1376162.19 to 1392805.21) | 41.12(41.05 to 41.19) |
| Down syndrome | Female | 2017 | 1377488.8(1369123.74 to 1385853.86) | 40.65(40.58 to 40.72) |
| Down syndrome | Female | 2018 | 1350018.95(1341665.64 to 1358372.27) | 39.74(39.67 to 39.8) |
| Down syndrome | Female | 2019 | 1319635.45(1311307.04 to 1327963.86) | 38.9(38.84 to 38.97) |
| Down syndrome | Female | 2020 | 1247215.47(1238986.56 to 1255444.37) | 36.94(36.87 to 37) |
| Down syndrome | Female | 2021 | 1207532.8(1199334.18 to 1215731.42) | 36.08(36.01 to 36.14) |
| Down syndrome | Female | 2022 | 1200644.31(1099129.88 to 1302158.75) | 35.19(32.73 to 37.64) |
| Down syndrome | Female | 2023 | 1167291.45(1050569.8 to 1284013.09) | 34.1(31.17 to 37.03) |
| Down syndrome | Female | 2024 | 1134531.7(997285.37 to 1271778.02) | 33.05(29.49 to 36.61) |
| Down syndrome | Female | 2025 | 1102374.87(940673.12 to 1264076.62) | 32.03(27.74 to 36.32) |
| Down syndrome | Female | 2026 | 1071117.14(882160.68 to 1260073.6) | 31.04(25.95 to 36.12) |
| Down syndrome | Female | 2027 | 1040934.19(822757.34 to 1259111.03) | 30.07(24.16 to 35.99) |
| Down syndrome | Female | 2028 | 1012024.07(763281.81 to 1260766.34) | 29.14(22.37 to 35.91) |
| Down syndrome | Female | 2029 | 984462.22(704199.33 to 1264725.12) | 28.23(20.6 to 35.87) |
| Down syndrome | Female | 2030 | 958297.58(645774.47 to 1270820.7) | 27.35(18.85 to 35.86) |
| Down syndrome | Female | 2031 | 933573.95(588139.91 to 1279007.99) | 26.5(17.14 to 35.86) |
| Down syndrome | Female | 2032 | 910225.74(531278.57 to 1289172.91) | 25.67(15.47 to 35.87) |
| Down syndrome | Female | 2033 | 888213.81(475191.19 to 1301236.44) | 24.87(13.84 to 35.9) |
| Down syndrome | Female | 2034 | 867545.16(419834.46 to 1315255.87) | 24.09(12.26 to 35.93) |
| Down syndrome | Female | 2035 | 848202.63(365085.99 to 1331319.27) | 23.34(10.73 to 35.95) |
| Down syndrome | Female | 2036 | 830152.27(310769.02 to 1349535.52) | 22.61(9.24 to 35.98) |
| Down syndrome | Female | 2037 | 813346.6(256667.89 to 1370025.31) | 21.9(7.8 to 36) |
| Down syndrome | Female | 2038 | 797740.94(202587.23 to 1392894.65) | 21.22(6.42 to 36.02) |
| Down syndrome | Female | 2039 | 783276.7(148291.62 to 1418261.77) | 20.55(5.08 to 36.02) |
| Down syndrome | Female | 2040 | 769921.53(98552.26 to 1446336.15) | 19.91(3.79 to 36.02) |
| Down syndrome | Male | 1990 | 1324211.38(1317266.7 to 1331156.06) | 42.38(42.3 to 42.45) |
| Down syndrome | Male | 1991 | 1306591.31(1299645.5 to 1313537.11) | 41.63(41.55 to 41.7) |
| Down syndrome | Male | 1992 | 1286890.94(1279939.79 to 1293842.1) | 40.91(40.83 to 40.98) |
| Down syndrome | Male | 1993 | 1270874.08(1263901.74 to 1277846.43) | 40.38(40.31 to 40.45) |
| Down syndrome | Male | 1994 | 1253922.44(1246941.82 to 1260903.05) | 39.89(39.82 to 39.96) |
| Down syndrome | Male | 1995 | 1244026.63(1237018.36 to 1251034.9) | 39.66(39.6 to 39.73) |
| Down syndrome | Male | 1996 | 1234339.22(1227305.84 to 1241372.6) | 39.4(39.33 to 39.47) |
| Down syndrome | Male | 1997 | 1220476.34(1213421.36 to 1227531.32) | 38.97(38.9 to 39.04) |
| Down syndrome | Male | 1998 | 1201590.77(1194518.65 to 1208662.89) | 38.35(38.28 to 38.41) |
| Down syndrome | Male | 1999 | 1200487.85(1193359.97 to 1207615.73) | 38.27(38.2 to 38.34) |
| Down syndrome | Male | 2000 | 1200295.42(1193108.42 to 1207482.42) | 38.19(38.12 to 38.26) |
| Down syndrome | Male | 2001 | 1188725.2(1181493.93 to 1195956.47) | 37.71(37.64 to 37.78) |
| Down syndrome | Male | 2002 | 1183233.05(1175959.6 to 1190506.5) | 37.37(37.3 to 37.44) |
| Down syndrome | Male | 2003 | 1172669.15(1165350.24 to 1179988.06) | 36.82(36.75 to 36.88) |
| Down syndrome | Male | 2004 | 1162548.67(1155209.59 to 1169887.75) | 36.24(36.17 to 36.3) |
| Down syndrome | Male | 2005 | 1162091.85(1154706.29 to 1169477.42) | 35.93(35.87 to 36) |
| Down syndrome | Male | 2006 | 1164855.22(1157430.36 to 1172280.08) | 35.68(35.62 to 35.75) |
| Down syndrome | Male | 2007 | 1166663.05(1159203.56 to 1174122.54) | 35.36(35.29 to 35.42) |
| Down syndrome | Male | 2008 | 1180022.5(1172500.34 to 1187544.67) | 35.35(35.29 to 35.42) |
| Down syndrome | Male | 2009 | 1187299.55(1179715.31 to 1194883.8) | 35.15(35.09 to 35.22) |
| Down syndrome | Male | 2010 | 1184159.71(1176540.01 to 1191779.41) | 34.71(34.65 to 34.77) |
| Down syndrome | Male | 2011 | 1184900.65(1177234.55 to 1192566.76) | 34.47(34.41 to 34.53) |
| Down syndrome | Male | 2012 | 1198526.87(1190792.07 to 1206261.67) | 34.63(34.56 to 34.69) |
| Down syndrome | Male | 2013 | 1212159.38(1204350.59 to 1219968.16) | 34.77(34.71 to 34.84) |
| Down syndrome | Male | 2014 | 1226936.3(1219046.37 to 1234826.23) | 34.96(34.9 to 35.02) |
| Down syndrome | Male | 2015 | 1243644.77(1235664.74 to 1251624.81) | 35.15(35.09 to 35.21) |
| Down syndrome | Male | 2016 | 1260583.89(1252508.63 to 1268659.15) | 35.31(35.25 to 35.37) |
| Down syndrome | Male | 2017 | 1261537.76(1253405.94 to 1269669.57) | 35.1(35.04 to 35.16) |
| Down syndrome | Male | 2018 | 1246496.01(1238357.98 to 1254634.05) | 34.58(34.52 to 34.65) |
| Down syndrome | Male | 2019 | 1228327.74(1220185.68 to 1236469.79) | 34.12(34.06 to 34.18) |
| Down syndrome | Male | 2020 | 1178868.01(1170796.52 to 1186939.5) | 32.92(32.86 to 32.98) |
| Down syndrome | Male | 2021 | 1145592.96(1137571.54 to 1153614.38) | 32.31(32.25 to 32.37) |
| Down syndrome | Male | 2022 | 1138672.81(1054938.74 to 1222406.87) | 31.5(29.61 to 33.38) |
| Down syndrome | Male | 2023 | 1111912.31(1014643.23 to 1209181.39) | 30.68(28.39 to 32.97) |
| Down syndrome | Male | 2024 | 1085416.98(969835.85 to 1200998.11) | 29.88(27.07 to 32.7) |
| Down syndrome | Male | 2025 | 1059130.43(921659.66 to 1196601.19) | 29.11(25.67 to 32.54) |
| Down syndrome | Male | 2026 | 1033280.96(871328.58 to 1195233.34) | 28.34(24.23 to 32.46) |
| Down syndrome | Male | 2027 | 1008043.6(819743.97 to 1196343.24) | 27.6(22.77 to 32.44) |
| Down syndrome | Male | 2028 | 983602.39(767640.05 to 1199564.73) | 26.88(21.3 to 32.46) |
| Down syndrome | Male | 2029 | 960088.56(715493.69 to 1204683.42) | 26.17(19.83 to 32.51) |
| Down syndrome | Male | 2030 | 937595.1(663597.28 to 1211592.92) | 25.48(18.38 to 32.59) |
| Down syndrome | Male | 2031 | 916192.63(612117.9 to 1220267.36) | 24.81(16.94 to 32.69) |
| Down syndrome | Male | 2032 | 895883.27(561110.27 to 1230656.27) | 24.16(15.52 to 32.8) |
| Down syndrome | Male | 2033 | 876609.16(510583.4 to 1242634.91) | 23.52(14.12 to 32.92) |
| Down syndrome | Male | 2034 | 858377.3(460533.11 to 1256221.5) | 22.9(12.75 to 33.05) |
| Down syndrome | Male | 2035 | 841204.58(410909.07 to 1271500.09) | 22.29(11.4 to 33.19) |
| Down syndrome | Male | 2036 | 825091.3(361611.03 to 1288571.58) | 21.7(10.09 to 33.32) |
| Down syndrome | Male | 2037 | 810029.74(312506.5 to 1307552.97) | 21.13(8.8 to 33.46) |
| Down syndrome | Male | 2038 | 795968.64(263455.65 to 1328481.63) | 20.57(7.55 to 33.58) |
| Down syndrome | Male | 2039 | 782820.28(214279.64 to 1351360.93) | 20.02(6.33 to 33.71) |
| Down syndrome | Male | 2040 | 770552.92(164793.17 to 1376312.68) | 19.49(5.15 to 33.83) |
